# Long intergenic non-coding RNA DIO3OS promotes osteosarcoma metastasis via activation of the TGF-β signaling pathway: a potential diagnostic and immunotherapeutic target for osteosarcoma

**DOI:** 10.1186/s12935-023-03076-5

**Published:** 2023-09-26

**Authors:** Jinghong Yuan, Jingyu Jia, Tianlong Wu, Zhi Du, Qi Chen, Jian Zhang, Zhiwen Wu, Zhao Yuan, Xiaokun Zhao, Jiahao Liu, Jia Guo, Xigao Cheng

**Affiliations:** 1 Department of Orthopaedics, The Second Affiliated Hospital of Nanchang University, Nanchang, 1 Minde Road, Donghu, Nanchang, 330006 Jiangxi People’s Republic of China; 2 Institute of Orthopaedics of Jiangxi Province, Nanchang, Jiangxi China; 3 Institute of Minimally Invasive Orthopaedics of Nanchang University, Nanchang University, Nanchang, Jiangxi China; 4 Clinical Research Center, The Second Affiliated Hospital of Nanchang University, Nanchang, Jiangxi China; 5 Department of Orthopaedics, Jiangxi Provincial People’s Hospital Affiliated to Nanchang University, Nanchang, Jiangxi China

**Keywords:** Osteosarcoma, DIO3OS, TGF-β signaling pathway, Immunotherapy, Diagnosis

## Abstract

**Background:**

The aim of this study was to determine the underlying potential mechanisms and function of DIO3OS, a lincRNA in osteosarcoma and clarify that DIO3OS can be used as a potential diagnostic biomarker and immunotherapeutic target.

**Methods:**

The expression matrix data and clinical information were obtained from XENA platform of UCSC and GEO database as the test cohorts. The external validation cohort was collected from our hospital. Bioinformatics analysis was used to annotate the biological function of DIO3OS. Immune infiltration and immune checkpoint analysis were applied to evaluate whether DIO3OS can be used as an immunotherapeutic target. ROC curves and AUC were established to assess the diagnostic value of DIO3OS for differentiating patients from other subtypes sarcoma. The expression analysis was detected by qRT-PCR, western blot, and immunohistochemical. Wound healing assay and Transwell assay were applied to determine the migration and invasion function of DIO3OS in osteosarcoma cell lines. The tail vein injection osteosarcoma cells metastases model was used in this research.

**Results:**

High expression of DIO3OS was identified as a risk lincRNA for predicting overall survival of osteosarcoma in test cohort. The outcomes of experiments in vitro and in vivo showed that low expression of DIO3OS limited osteosarcoma tumor metastasis with inhibiting TGF-β signaling pathway. Immune checkpoint genes (CD200 and TNFRSF25) expressions were inhibited in the low DIO3OS expression group. The DIO3OS expression can be applied to reliably distinguish osteosarcoma from lipomatous neoplasms, myomatous neoplasms, nerve sheath tumors, and synovial-like neoplasms. This result was further validated in the validation cohort.

**Conclusions:**

In conclusion, our outcomes indicated that DIO3OS is a potential diagnostic and prognostic biomarker of osteosarcoma, emphasizing its potential as a target of immunotherapy to improve the treatment of osteosarcoma through TGF-β signaling pathway.

*Trial registration number:* The present retrospectively study was approved by the Ethics Committee of The Second Affiliated Hospital of Nanchang University [Review (2020) No. (115)].

**Supplementary Information:**

The online version contains supplementary material available at 10.1186/s12935-023-03076-5.

## Background

Osteosarcoma is a common primary solid malignant tumor in the bones of young adults, adolescents, and children [1]. The common therapy for osteosarcoma was the operation combined with before and after surgery chemotherapy and the overall survival of 5 years rate reached 70% [2]. However, after 30 years of research development, the 5-year overall survival rate for patients with metastatic and recurrent osteosarcoma is only 20% [3]. Despite previous studies that have identified genomic instability and high frequency of chromosomal fragmentation, osteosarcoma rarely has recurrent targeted mutations, and the results of targeted drug trials have not been satisfactory [2, 4–6]. Therefore, it is necessary to seek potential targets for improving the outcomes of osteosarcoma therapy.

In previous studies, long non-coding RNAs (lncRNAs), a type of RNA with more than 200 nucleotides with a long non-coding domain are involved in a series of biological processes, including invitation, promoting, and migration of cancers [7–9]. In addition, most studies suggested that lncRNAs were determined as the biomarkers of tumor suppressors or promoters in cancer diagnosis, development, metastasis, and prognosis [10–12]. LncRNAs also play a crucial role in the development of osteosarcoma [13, 14]. The research of Chen et al. reported that the upregulated expression of NR_027471 as a sponging of miR-8055 inhibited the development, migration, and induced cell cycle arrest group in G1 phase by increasing the TP53INP1 expression in vivo and vitro [13]. The experiments of Yao et al. revealed that YY1 accelerated the TNK2-AS1 mRNA expression to increase the expression of WDR1 through inhibiting miR-4319 in osteosarcoma cells [14]. In some previous studies, DIO3OS was indicated as a crucial lncRNA to promote or suppress disease progression in non-small cell lung cancer, esophageal squamous cell carcinoma, thyroid cancer, and cervical carcinoma [15–18]. However, the potation mechanism of DIO3OS in osteosarcoma is unclear.

Activation of the TGF-β signaling pathway promotes the occurrence and development of diseases by promoting cell proliferation, cell cycle progression, recovery of immune responses, invasion, and tumorigenesis [19]. A growing number of preclinical and clinical studies have been already using drugs targeting the TGF-β signaling pathway, including anti-ligand antisense oligonucleotides, antibodies targeting ligands or receptors, and targeting TGF-β-receptor kinase drugs, especially in end-stage cancers [20–24].

Here, for the first time, high expression of DIO3OS was associated with poor prognosis of osteosarcoma and can be a potential diagnostic biomarker to distinguish osteosarcoma from other variety subtypes of sarcoma reported. Further experiments in vitro and in vivo were applied to understand the potential molecular mechanisms of DIO3OS/TGF-β pathway in the treatment of osteosarcoma. In the current study, the outcomes indicated that DIO3OS played a crucial role in osteosarcoma and may serve as a potential diagnostic biomarker and immunotherapeutic target in osteosarcoma.

## Methods

### Data collection and analysis

An easy-to-use pre-compiled data of the XENA platform were downloaded from UCSC (https://xenabrowser.net/datapages/). A total of 88 osteosarcoma cases expression matrix of GDC TARGET osteosarcoma project from the data were included in the present research. The expression matrix of the 58,387 genes, based on the log2(FPKM + 1), was collected in the GDC TARGET osteosarcoma project on July 23, 2019.

The clinical information was downloaded from the XENA platform provided by UCSC on September 13, 2018. Patients surviving less than 30 days were excluded from this study, overall survival data from 85 patient samples were included in the study. Using survival (3.2–10 version) and surviminer (0.4.9 version) packages, these overall survival-related genes were identified.

In addition, a microarray included 37 osteosarcoma cases, and related clinical information data were collected from the GSE39055 of the open Gene Expression Omnibus (GEO; http://www.ncbi.nlm.nih.gov/geo/) database. Based on survival (3.2–10 version) and surviminer (0.4.9 version) packages, these overall survival-related genes were demonstrated. Based on the R software (3.6.3 version), the crucial overall-survival-related genes and lncRNAs were selected and visualized by ggplot2 package (3.3.3 version). To further identify the expression of lncRNAs between osteosarcoma and control samples, GSE12865, which included 2 control osteoblast cell lines and 12 osteosarcoma cell lines, was downloaded from the GEO database (Additional file 1).

Tissues and clinical information of 62 patients (32 osteosarcoma, 12 lipomatous, 7 myomatous neoplasms, 6 nerve sheath tumors, and 5 synovial-like neoplasms) were provided by the Second Affiliated Hospital of Nanchang University from January 2012 to December 2016 as an external validation cohort. Survival distributions of clinical data were estimated using the log-rank test. Before collecting pathological tissue by puncture, no patient had received chemotherapy or radiotherapy. The metastatic status of the patients was evaluated at the first time of diagnosis. The clinical information of patients was shown in Table 1. The present study was approved by the Ethics Committee of The Second Affiliated Hospital of Nanchang University [Review (2020) No. (115)]. Written informed consent was obtained from all participants. All tissue samples were preserved in liquid nitrogen until RNA extraction.

**Table 1:** Baseline clinical characteristics of validation cohort

Characteristic	Levels	Overall
n		62
Group, n (%)	Lipomatous neoplasms	12 (19.4%)
	Myomatous neoplasms	7 (11.3%)
	Nerve sheath tumors	6 (9.7%)
	Osteosarcoma	32 (51.6%)
	Synovial-like neoplasms	5 (8.1%)
Gender, n (%)	Female	23 (37.1%)
	Male	39 (62.9%)
M/NM, n (%)	M	30 (48.4%)
	NM	32 (51.6%)
Survival status, n (%)	Alive	32 (51.6%)
	Dead	30 (48.4%)
Age (Years), median (IQR)		24.5 (16.25, 34)
Survival time (Days), median (IQR)		1613.5 (443, 2351)

### Survival analysis

To evaluate the accuracy of genes expression in predicting overall survival time (OS) and progression-free survival time (PFS), Kaplan–Meier (KM) survival analyses of the TARGET database and GSE39055 were assessed using the survival (3.2–10 version) and surviminer (0.4.9 version) packages. In addition, the receiver operating characteristics (ROC) curves and the area under the ROC curve (AUC) of 1-, 3-, and 5-year survival prognosis of genes were identified using timeROC package (0.4 version) and visualized by ggplot2 (3.3.3 version).

### DIO3OS-related expression analysis

Based on the TARGET database, samples with DIO3OS expression value of 0, and the expression matrix of 80 osteosarcoma cases was used for further analysis. According to the median value of DIO3OS expression as the cutoff, the 80 cases were divided into low (n = 40) and high (n = 40) DIO3OS expression groups. Between low and high DIO3OS expression groups, significant differentially expressed genes (DEGs) were determined with the limma package (3.14 version) in R (|FC|> 1.5 and p < 0.05). Volcano plots were constructed using ggplot2 package (3.3.3 version), and heat maps were generated with pheatmap package (1.0.12 version) in R.

A pan-cancer HTSeq-TPM database of TCGA and GTEx, processed uniformly by Toil, was downloaded from the XENA platform in USCS. These differential expressions of DIO3OS between tumor and control tissues were constructed in R and visualized with ggplot2 package.

### Functional enrichment analysis and Gene set enrichment analysis (GSEA)

On Xiantao Academic online (https://www.xiantaozi.com/), Gene Ontology (GO) and Kyoto Encyclopedia of Genes and Genomes (KEGG) functional enrichment analyses were performed on DEGs between high and low DIO3OS expression groups in the TARGET cohort by using clusterProfiler package (3.14.3 version), org.Hs.eg.db package (3.10.0 version) and GOplot package (1.0.2 version). These outcomes were visualized in the histogram, bubble plot, circle diagram, chord diagram, and functional enrichment network by ggplot2 package on Xiantao Academic online.

According to Pearson's correlation analysis, DIO3OS-related genes were screened by using psych package from TARGET database. Based on h.all.v7.2.symbols.gmt [hallmarks] as reference gene set, using clusterProfiler package, significant differences in pathways and function between high/low DIO3OS expression groups were identified and visualized in ggplot2 package.

### TGF-β signaling pathway analysis

A protein–protein interaction (PPI) network of DEGs in the TGF-β signaling pathway was constructed using Cytoscape (3.7.2 version). Based on Xiantao Academic online, DIO3OS co-expression heatmap and expression correlation scatter plots were calculated between DIO3OS expression in the TARGET cohort with the DEGs in the TGF-β related PPI network. These results of correlation analysis were performed in R and the outcomes were visualized by ggplot2 package.

### Immune infiltration and immune checkpoint analysis

In the present research, the CIBERSORT package was applied to evaluate the infiltration abundance of 22 types of immune cells between the high/low DIO3OS expression groups. Furthermore, the correlation between the expression distribution of 22 cells in the two groups was demonstrated and plotted a heat map using corrplot package (0.84 version) in R. Moreover, the expression levels of immune checkpoint genes were evaluated and visualized using ggpubr (0.4.0 version) and ggplot2 packages in R.

### Differential diagnostic analysis

DIO3OS expression of osteosarcoma and other subtypes of sarcoma (fibromatous neoplasms, lipomatous neoplasms, myomatous neoplasms, nerve sheath tumors, soft tissue tumors and sarcomas, and synovial-like neoplasms) were collected from the pan-cancer HTSeq-TPM database of TCGA and GTEx, processed uniformly by Toil, in the XENA platform on USCS. Differences in the expression of DIO3OS were visualized by Hiplot, an online tool (https://hiplot.com.cn/). ROC curves and AUC were established to evaluate the diagnostic value of DIO3OS for differentiating patients from other subtypes of sarcoma.

### Cell culture, transfection, and treatment

Four human osteosarcoma cell lines (SaoS-2, U2OS, MG-63, and HOS) and a control cell line (hFOB 1.19) were from American Tissue Culture Collection (ATCC). The hFOB 1.19 cell line was incubated in complete Dulbecco’s modified Eagle’s medium (DMEM) culture medium (Procell, Cat. No PM150210) supplemented with 1% penicillin/streptomycin (NCM Biotech, Cat. No c125c5) and 10% fetal bovine serum (FBS, WISENT, Cat. No 085-060) at 37 ℃ with 5% CO2. The HOS cell line was incubated in modified Eagle’s medium (MEM) culture medium (Procell, Cat. No PM150410) supplemented with 1% penicillin/streptomycin (NCM Biotech, Cat. No c125c5) and 10% fetal bovine serum (FBS, WISENT, Cat. No 085-060) at 37 ℃ with 5% CO2. MG-63, SaoS-2, and U2OS cell lines were incubated in RPMI-1640 (Procell, Cat. No PM150110) supplemented with 1% penicillin/streptomycin (NCM Biotech, Cat. No c125c5) and 10% fetal bovine serum (FBS, WISENT, Cat. No 085–060) at 37 ℃ with 5% CO2.

DIO3OS smart silencer (Cat. No lnc3210428014954) and lncRNA smart silencer NC (Cat. No lnc3N0000001-1-5) were purchased from RiboBio (Guangzhou). Transient transfections were conducted with Lipofectamine 2000 regent (ThermoFisher Scientific, Cat. No 11668019), according to manufacturer’s guidelines. All sequences are provided in Additional file 3: Table S1. To determine the role of the TGF-β signaling pathway in cells of osteosarcoma, after 36 h of SaoS-2 and U2OS transfection, cells were treated with TGF-β1 (MedChemExpress, Cat. No HY-P7117) at a concentration of 10 ng/ml in complete medium 12 h.

### qRT-PCR

Total RNA was isolated from cells or human tissues using Trizol reagent (Invitrogen, Carlsbad, CA). The cDNA was generated from each 2 ug RNA sample using All-in-One™ First-Strand cDNA Synthesis Kit (GeneCopoeia, Cat. No QP006). The temperature protocol for reverse transcription was: 28 ºC for 2 min, 42 ºC for 30 min, and 85 ºC for 5 min. cDNA was subjected to initial denaturation at 94˚C for 2 min, followed by 40 cycles at 95˚C for 15 s and 60–68 ºC for 30 s, after the end, it starts from 65˚C to 95 ºC, rising by 0.5 ºC per second, using the BeyoFastTM SYBR Green qPCR Mix (Bio-Rad, Cat. No 1708882AP). The primers used in qRT-PCR are shown in Additional file 3: Table S2. The method was used for relative quantification and the quantifications were normalized by taking GAPDH as an internal reference. All experiments were repeated in three times.

### Nuclear and cytoplasmic RNA fraction isolations

According to the manufacturer’s fractions, the Cytoplasmic & Nuclear RNA Purification Kit (Norgen, Cat. No 21000, Canada) was used to prepare the nuclear and Cytoplasmic RNA fraction from 1 × 10^6^. After using the manufacturer’s instructions to isolate RNAs, RNA concentrations of nuclear and cytoplasmic were measured at OD260, and their purity was calculated with OD260/OD280. Following the manufacturer’s guidelines, a All-in-One™ First-Strand cDNA Synthesis Kit (GeneCopoeia, Cat. No QP006) and BeyoFastTM SYBR Green qPCR Mix (Bio-Rad, Cat. No 1708882AP) were applied to determine the expression level of DIO3OS in the nucleus (U6 as an internal reference) and cytoplasm (GAPDH as an internal reference). The primer sequences are performed in Additional file 3: Table S2.

### Transwell migration and Matrigel invasion assays

According to the manufacturer’s protocol, transwell migration and matrigel invasion assays were determined with a modified Boyden chamber (Corning, Cat. No 3422) or matrigel Invasion chambers (Brand BD BioCoatTM, Cat. No 354234). After being cultured with serum-free medium for 24 h, cells were resuspended and diluted in serum-free medium with 0.1% BSA (Saiguo Biotech, Cat. No 9045–46-8). 200 ul of serum-free medium containing 4 × 10^5^ treated cells for migration assay and invasion assay were added to the upper chambers, and 500 ul of complete medium was added to the lower chambers, and then the cells were incubated for 30 h (for the migration assay) and 48 h (for the invasion assay). After that, 10% formaldehyde solution was added to each well and fixed at 28 ℃ for 30 s, and then the adhering cells were washed once time with 1 × PBS. Then 0.5% crystal violet was added to 1 ml/ well for 20 min and washed 3 times with 1 × PBS. After dried, stained cells were evaluated in three independent areas under an inverted microscope (CHONGQING OPTEC INSTRUMENT, Cat. No BDS300).

### Wound healing assay

SaoS-2 and U2OS cells (5 × 10^5^/2 ml/well) were seeded into 6-well plates and cultured overnight. After discarding the culture medium, the cells were scratched by a 200 μl plastic pipette tip, and the cells scratched were washed three times by PBS and cultured in a new serum-free medium at 37 ℃. The wound was photographed by using an inverted microscope (Olympus; Cat. No CKX41) after 0, 24, and 48 h. The results were analyzed by Image J software (1.62 version; National Institute of Health, Bethesda, MD, USA).

### Western blotting analysis

In this study, western blotting was performed in cultured cells as indicated or tissue samples. The cells and tissue were collected and total proteins were extracted with RIPA lysis buffer (Beyotime, Cat. No P0013) and quantified using the BCA protein quantification kit (Beyotime, Cat. No P0010S) Each lane was loaded with 15 μg cells protein or 25 ug tissue protein. Samples were then separated via 10% SDS-PAGE and separated proteins were transferred to PVDF membranes. After blocking in 5% nonfat dry milk in TBS-T (0.1% Tween 20) for 1 h at 37 ºC, the membranes were incubated with anti-E-cad (4 ºC, 8 h; 1:1000, Proteintech, Cat. No 20874-1-AP), anti-Vimentin (4 ºC, 8 h; 1:1000, Proteintech, Cat. No 10366–1-AP), anti-p-SMAD2 (4 ºC, 8 h; 1:1000, CST, Cat. No 18338T), anti-SMAD2 (4 ºC, 8 h; 1:3000, Proteintech, Cat. No 12570-1-AP), anti-GAPDH-Rabbit (4 ºC, 8 h; 1:6000, Proteintech, Cat. No 10494–1-AP), and anti-GAPDH-Mouse (4 ºC, 8 h; 1:6000, Proteintech, Cat. No 60004–1-Ig) primary antibodies respectively, and then with the secondary Anti-mouse IgG (37 ºC, 1 h, 1:10,000, CST, Cat. No 14709) and Anti-rabbit IgG (37 ºC, 1 h, 1:10,000, CST, Cat. No 14708), the detection was performed using chemiluminescence (NCM Biotech, Cat. No P10100). The densities of protein blots were normalized to GAPDH. ImageJ software (1.46 version; National Institutes of Health) was used to quantify protein band intensity.

### Animal model of osteosarcoma metastasis

BALB/C nude mice (male, 5–6-week-old) were purchased from Hunan SJA Laboratory Animal Co., Ltd. According to the Guidelines for the Care and Use of Laboratory Animals, all experiments on animals were approved by the Animal Ethics Committee of The Second Affiliated Hospital of Nanchang University (Nanchang, China). The lung metastases model was established through tail vein injection of si-NC/si-DIO3OS SaoS-2 cells (5 × 106) into nude mice and si-NC/si-DIO3OS once every 4 days. A total of 12 mice were used in this study, involving six mice in the si-NC group and six mice in the si-DIO3OS group. After six weeks, the nodule number in the lung was counted in each group. In addition, isolated tumor tissues were used to further experiments. The expression levels of DIO3OS in the two groups were identified using qRT-PCR, and SMAD2 and p-SMAD2 protein expression were detected using western blotting analysis. Some tissues were used for immunohistochemical analysis.

### Immunohistochemical (IHC)

Tissue samples were either fixed in 4% formalin for 48 h followed by paraffin embedding and cut into 4-μm-thick sections. Slides were placed for 20 min in 10 nM sodium citrate buffer, pH 6.5 heat at 97˚C in order to unmask antigens. Tissue sections were incubated with antibodies against E-cad (1:1000, Proteintech, Cat. No 20874-1-AP) and Vimentin (1:5000, Proteintech, Cat. No 10366–1-AP). Furthermore, stained the slides with 3,3’-diaminobenzidine (DAB, Beyotime, Cat. No P0202) and were counterstained with hematoxylin following the manufacturer’s protocol. Three independent areas under an inverted microscope (CHONGQING OPTEC INSTRUMENT, Cat. No BDS300).

### Statistics analysis

In this research, the statistical analyses were identified using R statistical software (3.6.3 version) and Xiantao Academic online (https://www.xiantaozi.com/). The statistics of multiple comparisons were used one-way ANOVA. The one-on-one comparisons statistics analysis used Student’s t-test. The statistical analysis for categorical variables used chi-square test. Log-rank test was used for statistics of KM survival analysis. P-value of less than 0.05 is considered statistically significant (ns, p > 0.05; *, p < 0.05; **, p < 0.01; ***, p < 0.001; ****, p < 0.0001)).

## Results

### DIO3OS is a risk lincRNA in osteosarcoma

**Fig. 1:**
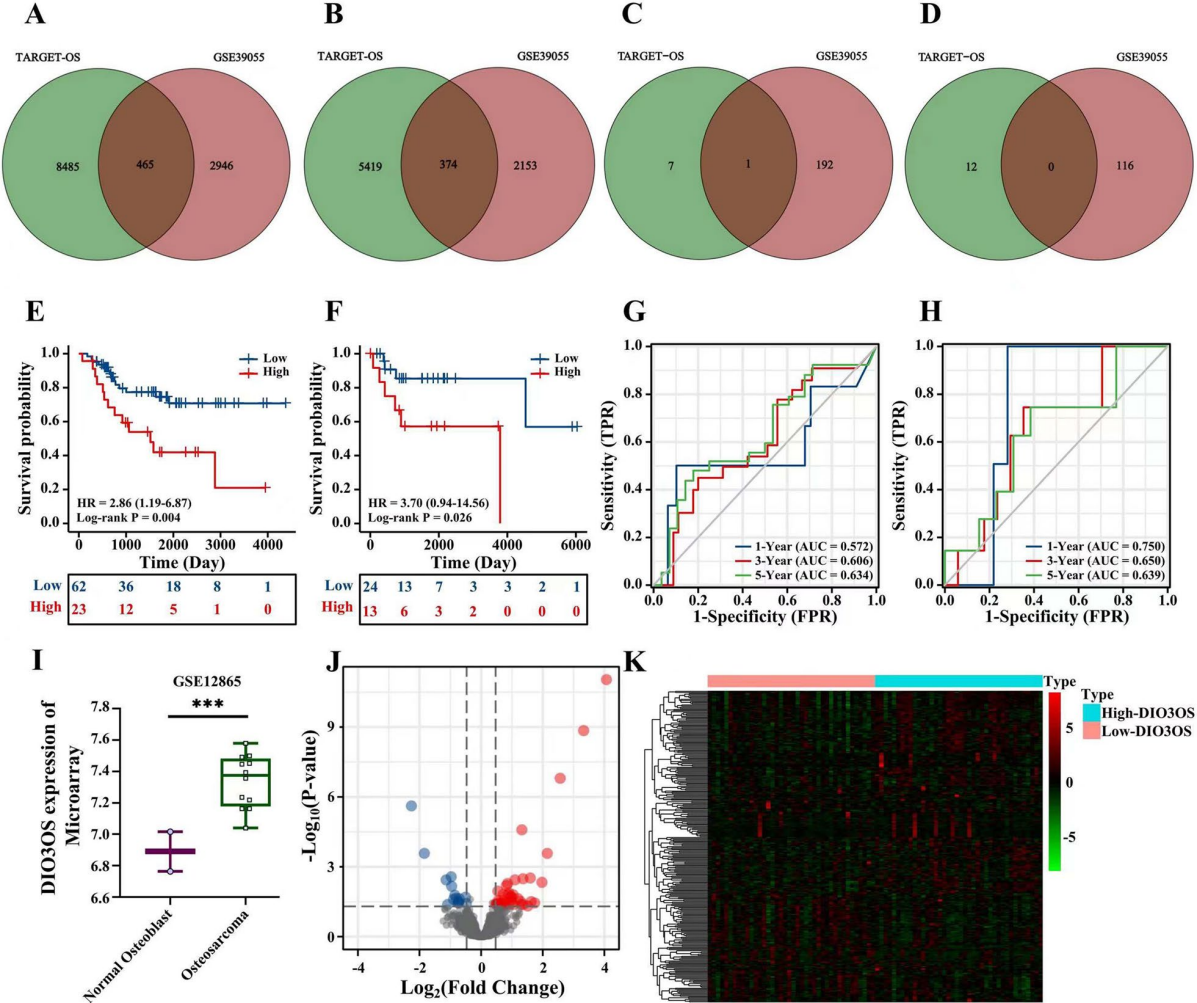
High expression of DIO3OS was a risk factor of overall survival and the validation and expression of DIO3OS in osteosarcoma cell lines. **A** 465 overlap overall survival risk genes between TARGET datasets and GSE39055. **B** 374 overlap overall survival protective genes between TARGET datasets and GSE39055. **C** 1 overlap overall survival risk lincRNAs between TARGET datasets and GSE39055. **D** 0 overlap overall survival protective lincRNAs between TARGET datasets and GSE39055. **E** The effect of high DIO3OS expression on the prognosis of osteosarcoma overall survival (OS) is statistically significant in TARGET cohort. **F** The effect of high DIO3OS expression on the prognosis of osteosarcoma overall survival (OS) is statistically significant in GSE39055. **G** Time-dependent ROC curves, 1-Year (AUC = 0.572), 3-Year (AUC = 0.606), 5-Year (AUC = 0.634). **H** Time-dependent ROC curves, 1-Year (AUC = 0.750), 3-Year (AUC = 0.650), 5-Year (AUC = 0.639). **I** Compared with normal osteoblast, DIO3OS is significantly higher expressed in osteosarcoma. **J** In the TARGET cohort, DIO3OS high and low expression groups showed significant differential genes between the two groups in the form of a volcano map. **K** The TARGET data set showed the significant differential genes between the two groups in the form of a heat map. (ns, p > 0.05; *, p < 0.05; **, p < 0.01; ***, p < 0.001; ****, p < 0.0001)

According to these results of KM survival analysis in 85 osteosarcoma patients from the TARGET database, 14,743 overall survival-related genes (8,950 risk gene, HR > 1, p < 0.05; 5,793 protective genes, HR < 1, p < 0.05) and 20 overall survival related lincRNAs (8 risk lincRNAs, HR > 1, p < 0.05; 12 protective lincRNAs, HR > 1, p < 0.05) were selected. Based on these outcomes of KM survival analysis in 37 osteosarcoma patients from GSE39055, 5,938 overall survival-related genes (3,411 risk gene, HR > 1, p < 0.05; 2,527 protective genes, HR < 1, p < 0.05) and 309 overall survival related lincRNAs (193 risk lincRNAs, HR > 1, p < 0.05; 116 protective lincRNAs, HR > 1, p < 0.05) were screened. To further determine the crucial overall survival-related genes and lincRNAs, 465 overlap risk genes, 374 overlap protective genes, 1 risk lincRNA (DIO3OS), and 0 protective lincRNA were identified according to these Venn plots (Fig. 1A–D).

Based on KM survival analysis results, DIO3OS is a risk lincRNA for predicting the overall survival of osteosarcoma in the TARGET database and GSE39055 (Fig. 1E, F). In addition, using the timeROC package, the time-dependent ROC curves (1-year, 3-year, and 5-year) were established, and the 1-year/3-year/5-year of AUC were 0.572, 0.60,6 and 0.634 in TARGET cohort and 0.750, 0.650 and 0.639 in GSE39055 (Fig. 1G, H). Based on these results, high expression of DIO3OS was a potential risk factor for poor prognosis in patients with osteosarcoma.

### Different expression analyses of DIO3OS

First of all, to clarify the expression of DIO3OS in other cancer, DIO3OS expression in pan-cancer were evaluated. These outcomes showed that DIO3OS were up-regulated in DLBC, PAAD, and THYM and down-regulated in ACC, BLCA, BRCA, CESC, COAD, ESCA, GBM, HNSC, KICH, KIRC, CIRP, LAML, LGG, LIHC, LUAD, LUSC, OV, PCPG, PRAD, SKCM, STAD, TGCT, THCA, UBEC, and UCS (Additional file 2: Fig. S1). Therefore, DIO3OS were reduced in most cancer, it may be used as a specific molecule for the diagnosis of osteosarcoma.

Furthermore, GSE12865 was used to determine the expression of DIO3OS between control and osteosarcoma. The outcome revealed that DIO3OS expression was up-regulated in osteosarcoma (Fig. 1I). To further explore the potential role of DIO3OS in osteosarcoma, based on the TARGET database, the differential expression analysis of DIO3OS results indicated that 248 DEGs (157 up-regulated and 91 down-regulated) were identified and visualized to a volcano and a heatmap (Fig. 1J, K).

### The TGF-β signaling pathway was activated in the high expression of DIO3OS group

**Fig. 2:**
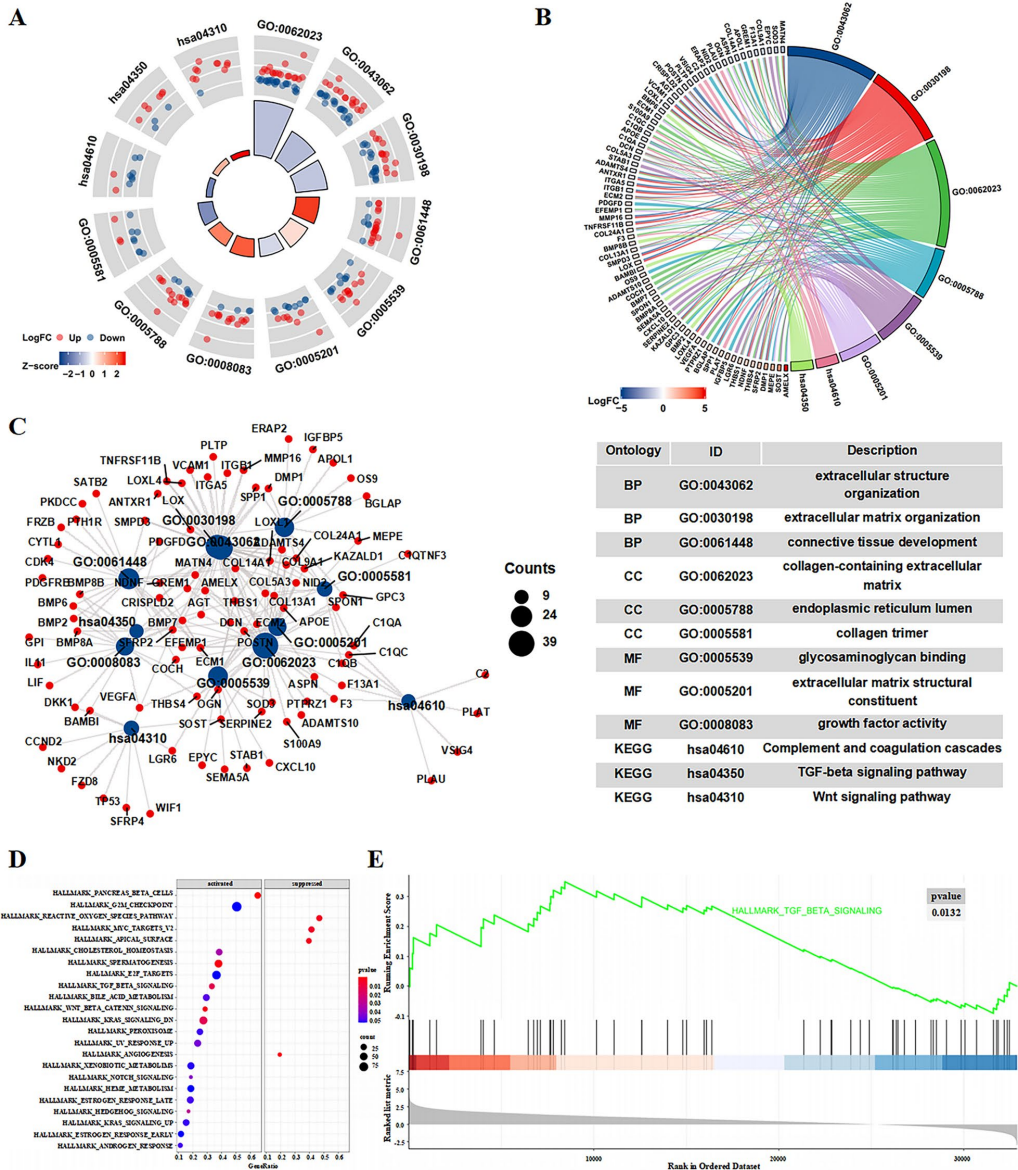
Functional enrichment analysis in TARGET cohort. **A** A circle graph showing the results of GO and KEGG enrichment analysis. Red nodes: Up-regulated in high expression of DIO3OS groups; Blue nodes: Down-regulated in high expression of DIO3OS groups. The blue or red color of the Z-score represents the activated or suppressed pathways in functional enrichment analysis. Red: Activated pathway; Blue: Suppressed pathway. **B** A chord plot showing the results of GO and KEGG enrichment analysis. The blue or red color of the LogFC represents gene expression between samples. Red: Up-regulated; Blue: Down-regulated. **C** A function enrichment network. Red nodes: genes; Blue Nodes: functional terms. **D** A bubble plot of GSEA enrichment analysis in TARGET cohort. p < 0.05. **E** TGF-β signaling pathway was significant enrichment using GSEA enrichment analysis in TARGET cohort

The results of GO and KEGG enrichment analysis for these DEGs included that “extracellular structure organization”, “extracellular matrix organization”, “endoplasmic reticulum lumen”, “growth factor activity”, and “TGF-β signaling pathway”. These outcomes were visualized as a circle graph, a chord graph, and a network graph (Fig. 2A, Top 3 in each part of biological process, molecular function, cellular component, and KEGG, p < 0.05; Fig. 2B, Top 2 in each part of biological process, molecular function, cellular component, and KEGG, p < 0.05; Fig. 2C, Top 3 in the part of biological process, molecular function, cellular component, and KEGG, p < 0.05).

The h.all.v7.2.symbols.gmt [hallmarks] was selected as the reference gene set in the present study GSEA. DEGs related to DIO3OS were involved that up-regulated of DIO3OS activated “TGF-β signaling pathway”, “G2M checkpoint”, “Pancreas β cells” et al. and suppressed “Reactive oxygen species pathway”, “Angiogenesis”, “MYC targets v2” et al. (Fig. 2D, E). These results enhanced that DIO3OS expression inhibited may suppress the activity of the TGF-β signaling pathway.

### DIO3OS may be involved in osteosarcoma progression through the TGF-β signaling pathway

**Fig. 3:**
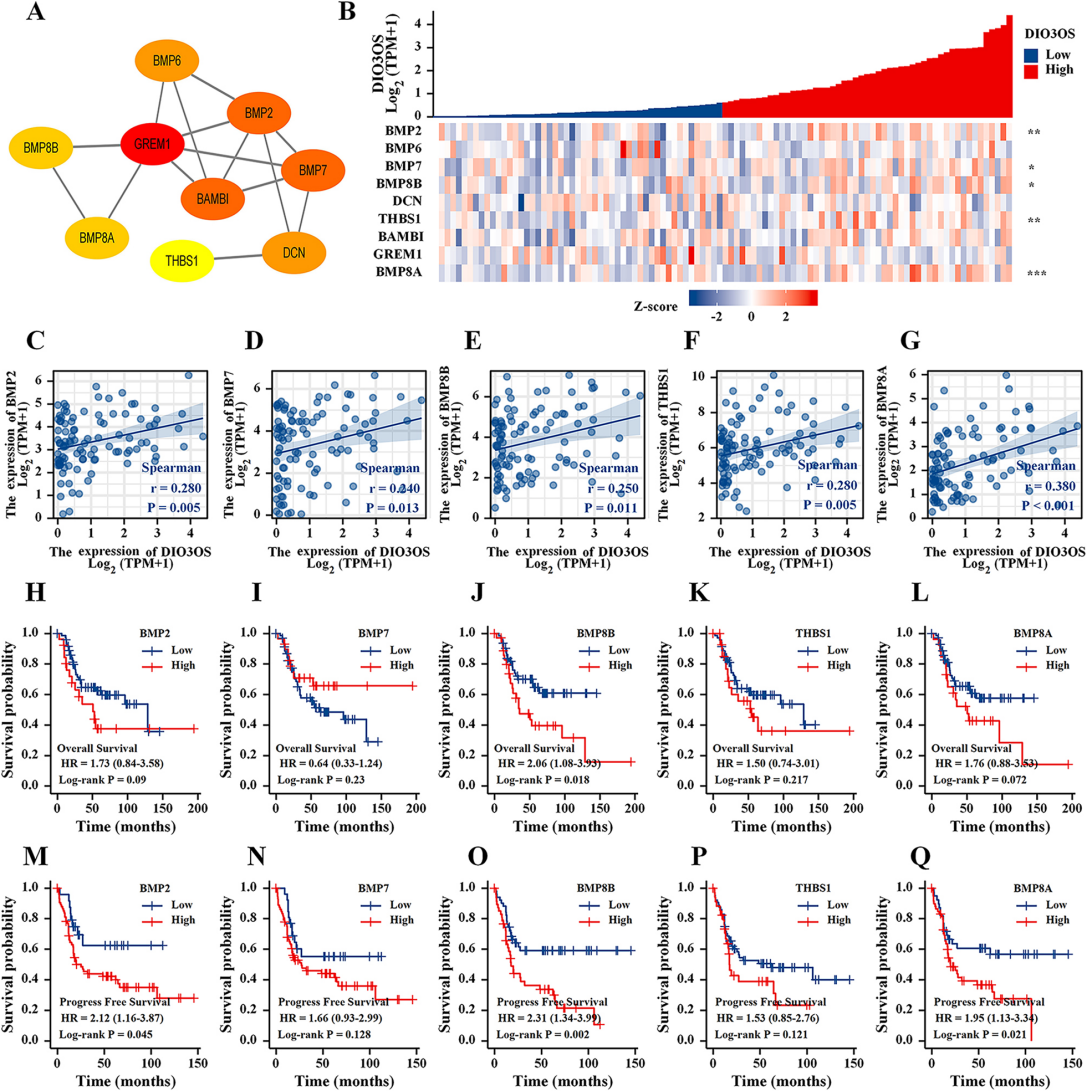
TGF-β signaling pathway analysis. **A** A PPI network of TGF-β signaling pathway in DEGs from TARGET cohort. **B** A gene expression related heat map of the 9 DEGs of TGF-β signaling pathway from TARGET cohort. **C**–**G** Scatter plots of the expression correlation between BMP2, BMP7, BMP8B, THBS1, and BMP8A and DIO3OS. **H–L** The effect of BMP2, BMP7, BMP8B, THBS1, and BMP8A expression on the prognosis of osteosarcoma overall survival (OS). **M**–**Q** The effect of BMP2, BMP7, BMP8B, THBS1, and BMP8A expression on the prognosis of osteosarcoma progress free survival (PFS). P-value of less than 0.05 is considered statistically significant (ns, p > 0.05; *, p < 0.05; **, p < 0.01; ***, p < 0.001)

Based on the DEGs related to DIO3OS in the TARGET cohort, the enrichment analysis determined that DIO3OS may mainly promote osteosarcoma progression by the TGF-β signaling pathway. A PPI network, including GREM1, BMP2, BAMBI, BMP7, BMP6, DCN, BMP8A, BMP8B, and THBS1, was established to identify these crucial DEGs-related DIO3OS in TGF -β signaling pathway (Fig. 3A). A gene co-expression heat map was constructed to show that expression levels of BMP2, BMP7, BMP8B, BAMBI, and BMP8A were positively correlated with DIO3OS expression in the TARGET database (Fig. 3B). Then, five scatterplots of correlation between these five genes and DIO3OS expression were generated (Fig. 3C–G). These results indicated that the expression of BMP2, BMP7, BMP8B, BAMBI, and BMP8A was suppressed when DIO3OS was down-regulated.

These outcomes of the KM analysis determined that OS and PFS significantly differed for these five DEGs from the TGF-β signaling pathway (Fig. 3H–Q). Therefore, the inhibition of osteosarcoma migration and invasion by silencing DIO3OS expression, possibly through inhibiting the TGF-β signaling pathway.

### Correlation between DIO3OS expression and immune analysis

**Fig. 4:**
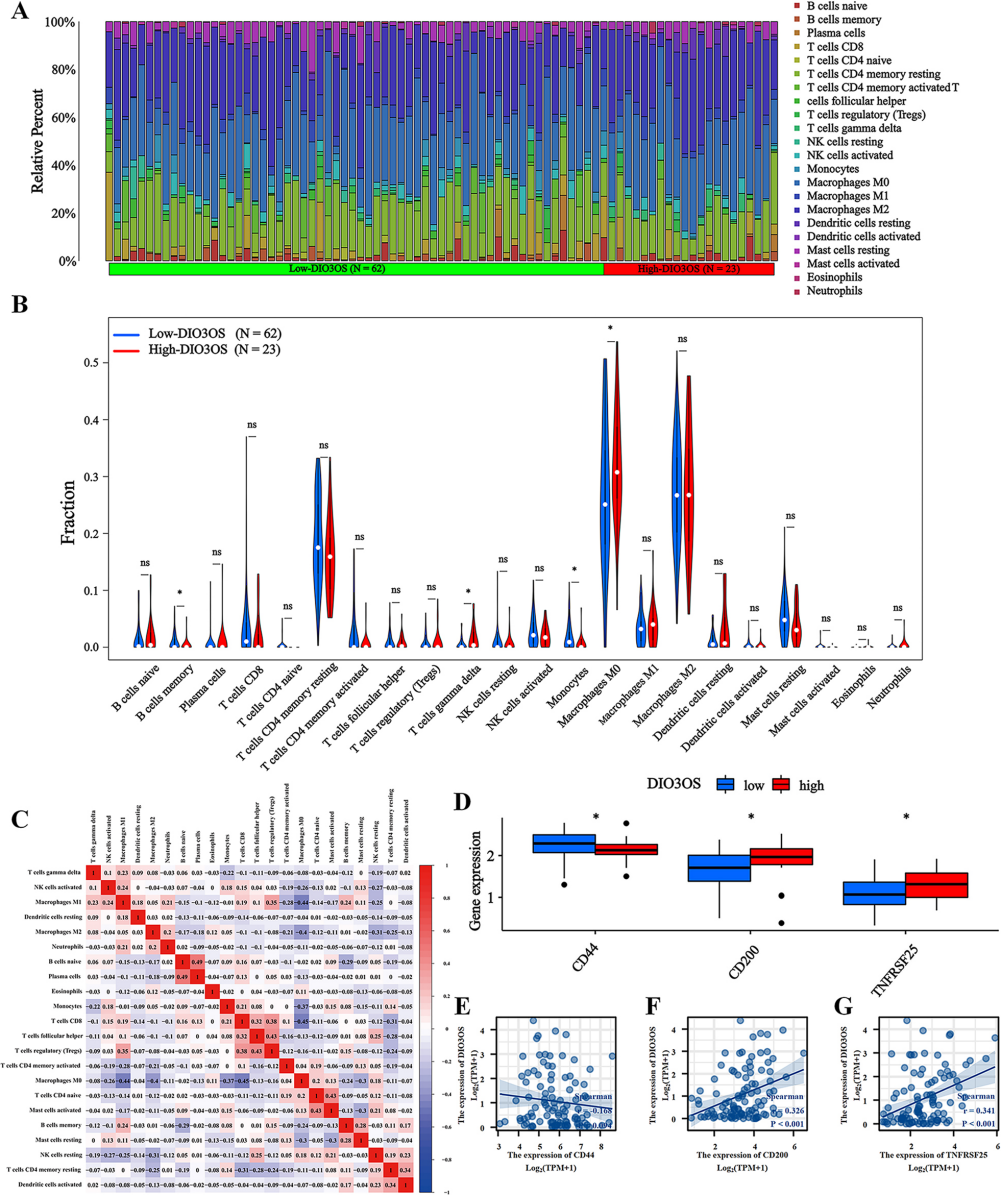
Immune related analysis. **A** The mean proportion of 22 immune cells in the Target database. **B** Violin diagram of the difference in expression of 22 immune cells infiltration between the high and low expression groups of DIO3OS in 85 osteosarcoma samples in the TARGET set. Blue represents the low expression group, red represents the high expression group. **C** Correlation heat map of 22 immune cells in the TARGET set. **D** In low expression of DIO3OS group, CD200 and TNFRSF25 expression were inhibited and CD44 expression were increased. **E–G** Scatter plots of the expression correlation between CD44, CD200, and TNFRSF25 and DIO3OS. P-value of less than 0.05 is considered statistically significant (ns, p > 0.05; *, p < 0.05; **, p < 0.01; ***, p < 0.001; ****, p < 0.0001)

To further evaluate immune infiltration, the CIBERSORT package and LM22 algorithm were used to calculate the infiltrating abundance of 22 immune cells between high/low DIO3OS expression groups in the TARGET database. These results demonstrated that in high expression of the DIO3OS group, the proportion ofT cells gamma delta, and Macrophages M0 were up-regulated, and B cells memory and Monocytes were reduced. The proportion of immune cells in each sample was visualized (Fig. 4A). To indicate the differences in the proportion of immune cells between the two groups, these results were visualized as a violin map and a correlation heat map (Fig. 4B, C). Thence, these results suggest that DI3OOS may be involved in the regulation of immune cell infiltration in osteosarcoma.

The assessment of immune checkpoint genes expression suggested that in low expression of the DIO3OS group, CD200 and TNFRSF25 expression were inhibited and CD44 expression was increased (Fig. 4D). These scatterplots of correlated expression between the three genes and DIO3OS indicated that the expression of CD40 was up-regulated in the low DIO3OS expression group (r = -0.168, p = 0.094 > 0.05) (Fig. 4E). However, CD200 and TNFRSF expression were inhibited in the low DI3OS expression group (r = 0.326, p < 0.001; r = 0.342, p < 0.001) (Fig. 4F, G). Therefore, silencing DIO3OS expression may improve the efficacy of osteosarcoma immunotherapy by inhibiting immune checkpoints (CD200 and TNFRSF25) [25–27].

### Diagnostic value of DIO3OS

**Fig. 5:**
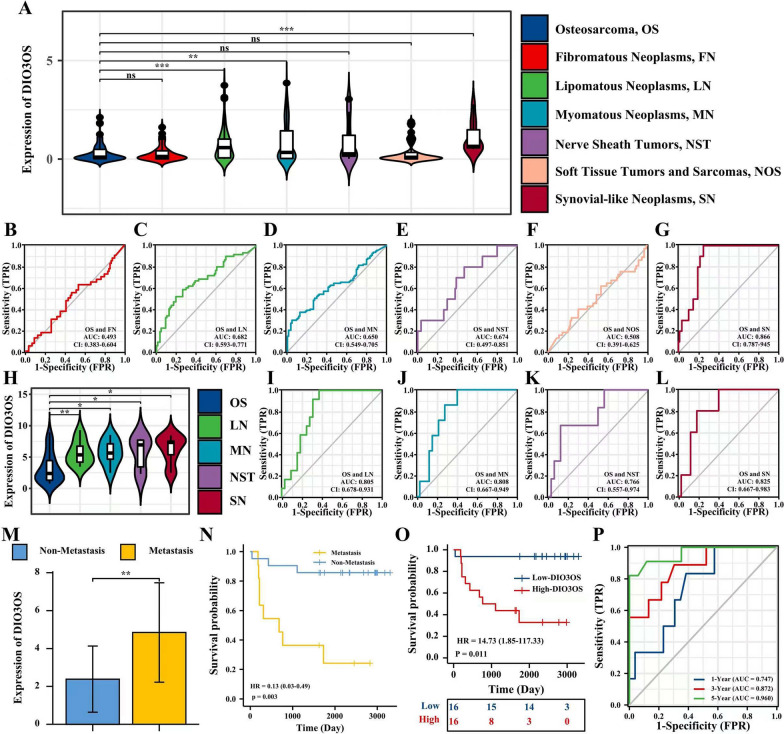
Testing and validating DIO3OS as a biomarker for the diagnosis of osteosarcoma. **A** DIO3OS expression in osteosarcoma and various subtypes of sarcoma (FN, LN, MN, NST, NOS, and SN) in test cohort. **B**–**G** The area under the ROC curve (AUC) in the TARGET and TCGA sets assesses the differential diagnostic performance of DIO3OS between osteosarcoma and other subtypes of sarcoma. **H** DIO3OS expression in osteosarcoma and various subtypes of sarcoma (LN, MN, NST, and SN) in validate cohort. **I**–**L** The area under the ROC curve (AUC) in the validate cohort assesses the differential diagnostic performance of DIO3OS between osteosarcoma and other subtypes of sarcoma. **M** In validate cohort, the expression level of DIO3OS between metastasis and non-metastasis groups. **N** The effect of metastasis on the prognosis of osteosarcoma overall survival (OS) is statistically significant in validate cohort. **O** The effect of high DIO3OS expression on the prognosis of osteosarcoma overall survival (OS) is statistically significant in validate cohort. **P** Time-dependent ROC curves, 1-Year (AUC = 0.747), 3-Year (AUC = 0.872), 5-Year (AUC = 0.960) in validate cohort. P-value of less than 0.05 is considered statistically significant (ns, p > 0.05; *, p < 0.05; **, p < 0.01; ***, p < 0.001; ****, p < 0.0001)

To evaluate the diagnostic accuracy of DIO3OS in distinguishing one subtype of sarcoma from another, the DIO3OS expression in osteosarcoma, fibromatous neoplasms, lipomatous neoplasms, myomatous neoplasms, nerve sheath tumors, soft tissue tumors and sarcomas, and synovial-like neoplasms were collected to assess. These outcomes suggested that the expression levels of DIO3OS in osteosarcoma were lower than lipomatous neoplasms (p < 0.001), myomatous neoplasms (p < 0.01), and synovial-like neoplasms (p < 0.001) (Fig. 5A).

The receiver operating characteristic (ROC) curves were drawn using the pROC package to analyze whether the expression level of DIO3OS could distinguish osteosarcoma from other subtypes of sarcoma. These results determined that the DIO3OS expression can be applied to reliably distinguish osteosarcoma from lipomatous neoplasms (AUC: 0.682, CI: 0.593–0.682), myomatous neoplasms (AUC: 0.650, CI: 0.549–0.705), nerve sheath tumors (AUC: 0.674, CI: 0.497–0.851), and synovial-like neoplasms (AUC: 0.866, CI: 0.787–0.945) (Fig. 5B–G). These outcomes indicated that the DIO3OS expression was an excellent diagnostic biomarker for osteosarcoma diagnosis.

### Validation of the diagnostic and prognostic value of DIO3OS

A validation cohort, including 62 patients (32 osteosarcoma, 12 lipomatous, 7 myomatous neoplasms, 6 nerve sheath tumors, and 5 synovial-like neoplasms), was applied to evaluate the diagnostic and prognostic reliability of DIO3OS. The expression levels of DIO3OS in the 62 patients were identified using qRT-PCR (Fig. 5H). These outcomes further indicated that the expression of DIO3OS can be used to distinguish osteosarcoma from lipomatous neoplasms (AUC: 0.805, CI: 0.678–0.931), myomatous neoplasms (AUC: 0.808, CI: 0.667–0.949), nerve sheath tumors (AUC: 0.766, CI: 0.557–0.974), and synovial-like neoplasms (AUC: 0.825, CI: 0.667–0.983) (Fig. 5I–L).

Furthermore, the patients with osteosarcoma in the validation cohort were used to further analysis (Additional file 3: Table S3). The expression levels of DIO3OS in the metastatic group were higher than those in the non-metastatic group (Fig. 5M), and patients with osteosarcoma in the metastatic group have a poor overall survival prognosis (Fig. 5N). Based on KM survival analysis, the result suggested that osteosarcoma patients with high expression of DIO3OS have a poor overall survival prognosis (Fig. 5O). The outcome of time-dependent ROC curves analysis showed that AUCs of 1-/3-/5-year were 0.747, 0.872, and 0.960 (Fig. 5P). It was further verified that the expression level of DIO3OS was a reliable diagnostic and prognostic biomarker for patients with osteosarcoma.

### Silencing DIO3OS expression inhibited cell migration and invasion in vitro

**Fig. 6:**
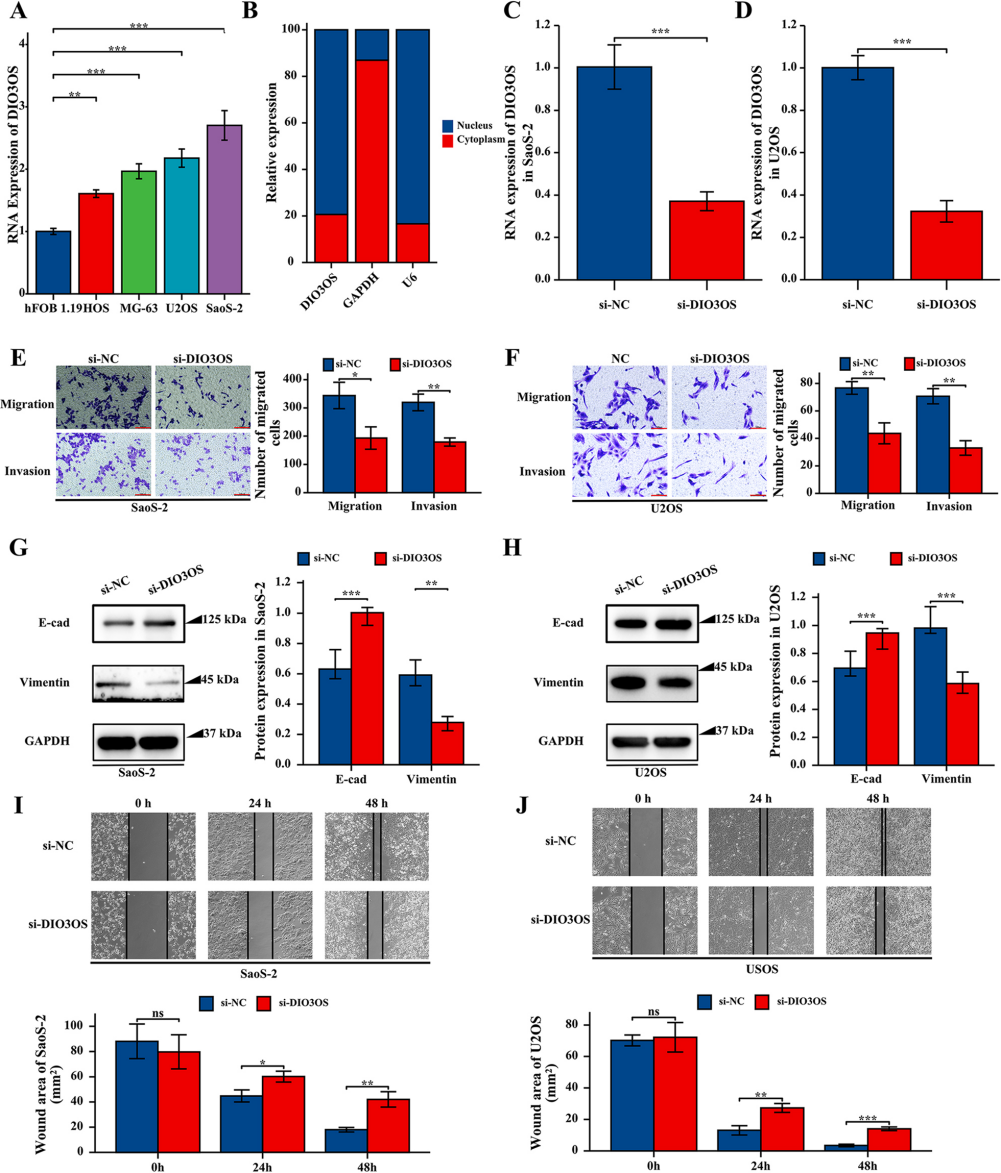
Knockdown of DIO3OS inhibited cellular migration, invasion of osteosarcoma cells in vitro. **A** Expression level of DIO3OS in osteosarcoma cell lines and healthy cell line detected by qRT-PCR. **B** The location of DIO3OS in SaoS-2 cell line was determined by qRT-PCR of nuclear and cytoplasmic RNA fraction isolations. **C** DIO3OS expression was inhibited in SaoS-2 cells after si-DIO3OS transfection. **D** DIO3OS expression was inhibited in U2OS cells after si-DIO3OS transfection. **E** Cell migration and invasion was inhibited in SaoS-2 cells transfected with si-DIO3OS. The scale bar were marked in red, 100 μm. **F** Cell migration and invasion was inhibited in U2OS cells transfected with si-DIO3OS. The scale bar were marked in red, 100 μm. **G** The expression levels of E-cad protein were increasd and Vimentin protein expression were inhibited in SaoS-2 cells after si-DIO3OS transfection. **H** The expression levels of E-cad were up-regulated and Vimentin protein were inhibited in U2OS cells after si-DIO3OS transfection. **I** DIO3OS knockdown suppressed cell migration capacity in SaoS-2 cells, as determined by wound healing assay. **J** DIO3OS knockdown suppressed cell migration capacity in U2OS cells, as determined by wound-healing assay. P-value of less than 0.05 is considered statistically significant (ns, p > 0.05; *, p < 0.05; **, p < 0.01; ***, p < 0.001; ****, p < 0.0001)

Using qRT-PCR, the DIO3OS expression of the control cell line (hFOB 1.19) was less than its expression of osteosarcoma cell lines (HOS, MG-63, U2OS, and SaoS-2) in Fig. 6A. Therefore, these outcomes showed that DIO3OS was up-regulated in osteosarcoma, suggesting high expression of DIO3OS might promote osteosarcoma progression. To further identify the mechanism of DIO3OS in osteosarcoma, the nuclear and cytoplasmic fractions of DIO3OS in the SaoS-2 cell line were determined by qRT-PCR of nuclear and cytoplasmic RNA fraction isolations. The results demonstrated that DIO3OS was significantly enriched in the nuclear fraction, revealing that DIO3OS could involve in the development of osteosarcoma in the nuclear fraction (Fig. 6B). To determine the role of DIO3OS in osteosarcoma cell lines, the specific lncRNA smart silence of DIO3OS (si-DIO3OS) was used to knock down the DIO3OS expression in SaoS-2 and U2OS cells (Fig. 6C, D).

To further determine the function of DIO3OS in migration, a Transwell assay was applied in the present study. The results demonstrated that the migration rate of SaoS-2 and U2OS cells transfected with si-DIO3OS was obviously lower than for control transfected cells (Fig. 6E, F). The invasion ability of DIO3OS silencing in SaoS-2 and U2OS cells was determined by Transwell chamber (with Matrigel) assays. Furthermore, the results indicated that the invasive ability of SaoS-2 and U2OS cells were decreased in the si-DIO3OS-transfected group (Fig. 6E, F). In addition, these epithelial biomarkers, The expression levels of E-cad were increased and Vimentin expression were down-regulated using western blotting (Fig. 6G, H). These results showed that DIO3OS silencing inhibited the invasion of SaoS-2 and U2OS cells.

Finally, the wound healing assay was applied to further identify the effect of DIO3OS on the migration of SaoS-2 and U2OS cells. These outcomes of the wound-healing assay revealed that the wound-healing rates of SaoS-2 and U2OS cells were significantly reduced in the downregulated expression of the DIO3OS group (Fig. 6I, J). These results showed that the downregulated expression of DIO3OS inhibited the migration ability of SaoS-2 and U2OS cells in vitro.

### Inhibited DIO3OS inactivated TGF-β signaling pathway

**Fig.7:**
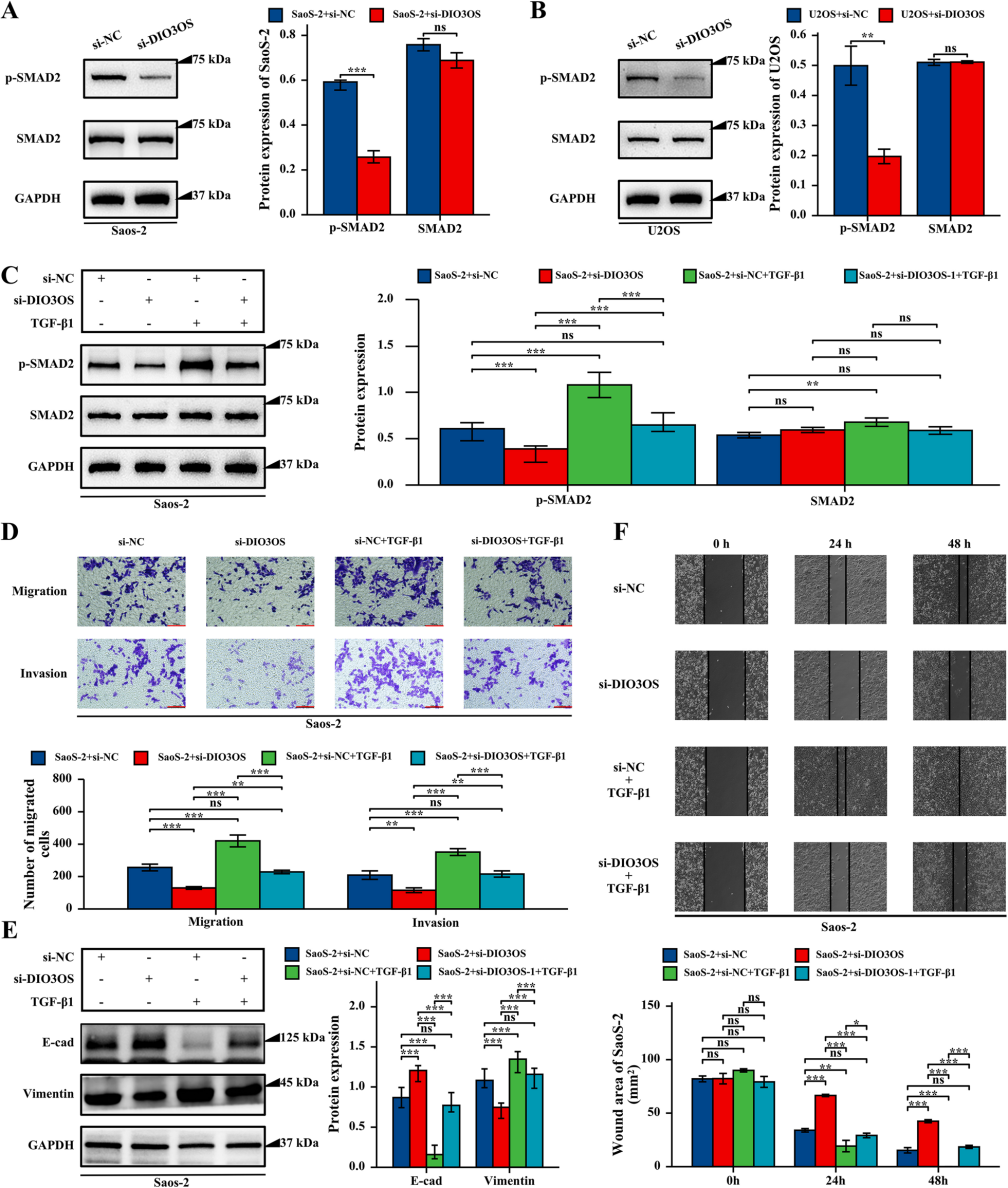
Activating TGF-β signaling pathway reversed si-DIO3OS-induced the attenuation of cell migration, invasion in osteosarcoma cells. **A** Inhibited DIO3OS expression repressed the activation of TGF-β signaling pathway in SaoS-2 cells. **B** Inhibited DIO3OS expression repressed the activation of TGF-β signaling pathway in U2OS cells. **C** TGF-β1 reversed si-DIO3OS-induced the attenuation of TGF-β signaling pathway in SaoS-2 cells. **D** Activating TGF-β signaling pathway reversed si-DIO3OS-induced the attenuation of cell migration, invasion in SaoS-2 cells using Transwell assays. The scale bar were marked in red, 100 μm. **E** TGF-β1 revised si-DIO3OS-induced the promoted expression of E-cad and inhibited expression of vimentin in SaoS-2 cells. **F** Activating TGF-β signaling pathway reversed si-DIO3OS-induced the attenuation of cell migration in SaoS-2 cells using wound healing assay.P-value of less than 0.05 is considered statistically significant (ns, p > 0.05; *, p < 0.05; **, p < 0.01; ***, p < 0.001; ****, p < 0.0001)

To further study the underlying mechanism of DIO3OS in osteosarcoma cells, the protein levels of SMAD2 and p-SMAD2 were compared between si-DIO3OS and control groups by using western blot analysis. The results showed that the phosphorylation of SMAD2 was downregulated in osteosarcoma cells transfected with si-DIO3OS and the total SMAD2 expression remained unchanged (Fig. 7A, B). Therefore, these results indicated that the inactivation of the TGF-β signaling pathway was correlated with DIO3OS down-regulated in osteosarcoma cells.

### *DIO3OS silencing suppressed migration and invasion of osteosarcoma *via* the TGF-β signaling pathway*

To determine the above mechanism, a rescue experiment was designed by using TGF-β1, and activation of the TGF-β signaling pathway significantly promoted the migration and invasion, and expression of E-cadherin were up-regulated and vimentin expression were decreased in osteosarcoma cells and rescued the effects of DIO3OS-knockdown (Fig. 7C–F). Therefore, these results suggested that the downregulation of DIO3OS may act by inhibiting the TGF-β signaling pathway in osteosarcoma cells.

### *Silencing of DIO3OS inhibited osteosarcoma metastasis *in vivo

**Fig. 8:**
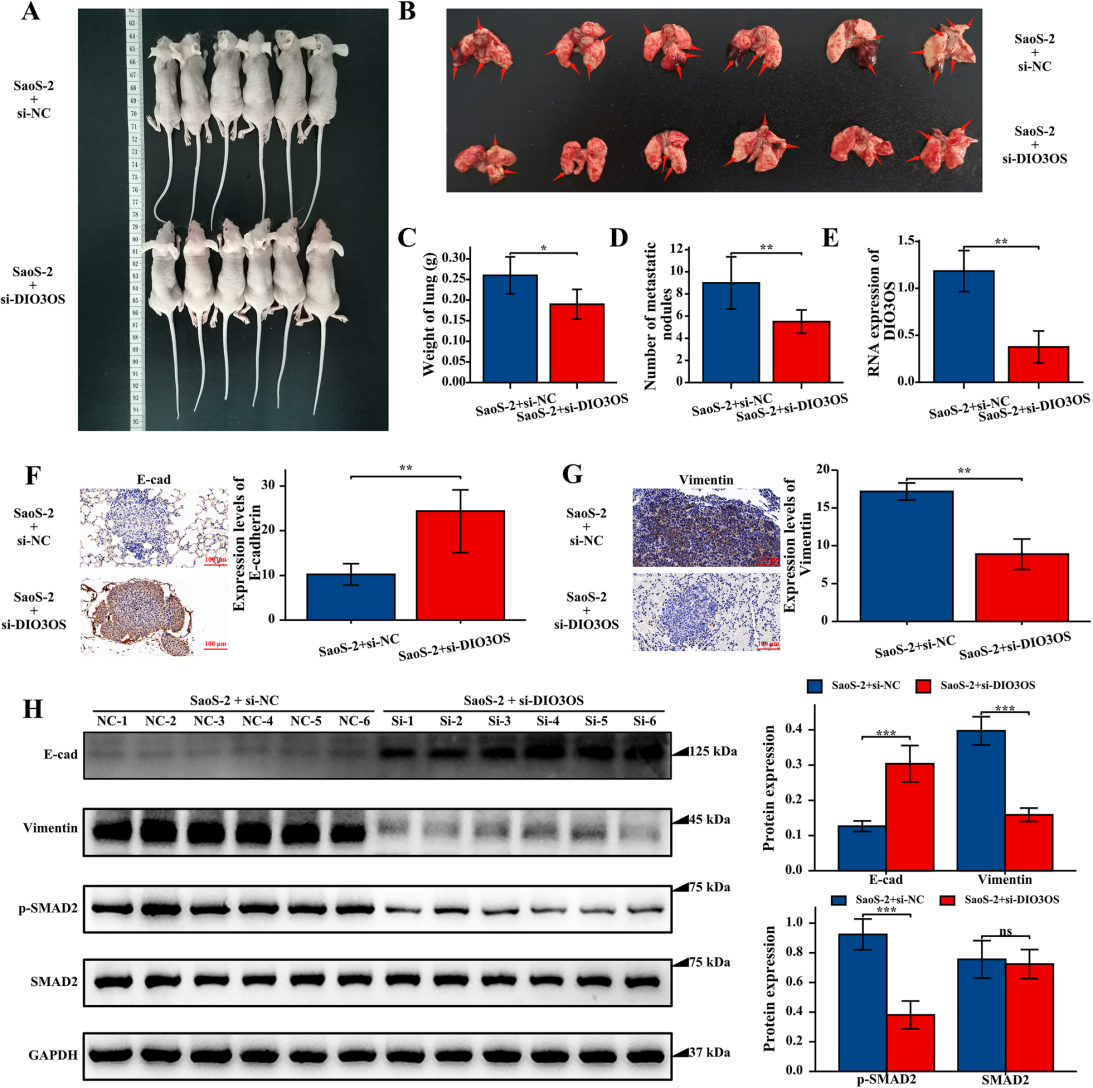
The DIO3OS silence inhibited osteosarcoma cell metastasis in vivo. **A** Images of BALB/C nude mice injected SaoS-2 cell treated with si-NC and si-DIO3OS via tail vein. **B** Images of lung tissue from BALB/C nude mice injected SaoS-2 cell treated with si-NC and si-DIO3OS via tail vein. **C** Weight of lung from si-DIO3OS group was significant lower than si-NC group. **D** Number of metastatic nodules in lung from si-DIO3OS was significant lower than si-NC group. **E** The expression of DIO3OS in two groups of mice using qRT-PCR. **F** The expression levels of E-cad were up-regulated in si-DI3OS group. **G** The expression levels of Vimentin were suppressed in si-DI3OS group. **H** Western blotting analysis of E-cad, Vimentin, p-SMAD2 and SMAD2 in two groups of mice. P-value of less than 0.05 is considered statistically significant (ns, p > 0.05; *, p < 0.05; **, p < 0.01; ***, p < 0.001; ****, p < 0.0001)

Based on the results of bioinformatics analysis and experiments in vitro, the silencing of DIO3OS plays a crucial role in inhibiting osteosarcoma metastasis. To further determine the role of DIO3OS in osteosarcoma in vivo, a lung metastasis model was established in nude mice by injecting SaoS-2 cells transfected with si-DIO3OS and si-NC into the tail veins of mice (Fig. 8A). As far as the tumor nodule number and weight in the lungs were concerned, DIO3OS knockdown inhibited lung metastasis of SaoS-2 cells in vivo (Fig. 8B–D). The results of qRT-PCR showed that DIO3OS was knocked down (Fig. 8E), and the IHC analysis and western blotting analysis results showed that expression levels of E-cad were increased and Vimentin were down-regulated in the si-DIO3OS group (Fig. 8F–H). Furthermore, the western blot analysis outcomes revealed that the expression of p-SMAD2 was down-regulated in the si-DIO3OS group and SMAD2 expression remained unchanged (Fig. 8H). These results suggested that DIO3OS downregulation inhibited osteosarcoma metastasis via the inactivation of the TGF-β signaling pathway.

## Discussion

Previous studies have suggested that tumor metastasis is the most crucial cause of death in patients with osteosarcoma, especially lung metastasis [1–3]. Most research focused on the underlying mechanism of osteosarcoma metastasis to improve the survival of osteosarcoma [1, 3]. However, no more effective treatment has been proposed to effectively improve the survival of osteosarcoma over the past three decades [1, 2]. Therefore, to further determining the molecular mechanisms of osteosarcoma progression and metastasis would reveal effective diagnostic targeted therapy to improve survival.

Nowadays, more and more studies have reported that lncRNAs played a crucial role in the proliferation, progression, metastasis, invasion, diagnosis, and prognosis of osteosarcoma [2–4]. The research of Wang et al. reported that high expression levels of HOTAIR promoted growth and metastasis by increasing the expression of MMP-2 and MMP-9 in osteosarcoma [28]. Qian et al. suggested that P50-associated COX-2 extragenic RNA (PACER) expression was higher in clinical osteosarcoma tissues and osteosarcoma cell lines, and the overexpression of PACER promoted the proliferation and metastasis of osteosarcoma cells by activating the COX-2 gene in an NF-κB-dependent manner [29]. The experiment of Sun et al. revealed that FGFR3-AS1 promoted osteosarcoma growth by regulating FGFR3, a natural antisense transcription product of FGFR3-AS1 [30]. In previous research, some researchers have explored the biological function of DIO3OS in non-small cell lung cancer, esophageal squamous cell carcinoma, thyroid cancer, and cervical carcinoma [15–18]. However, the role of DIO3OS in the potential mechanism of osteosarcoma has not been clarified. Here, we explored the clinical significance and potential molecular mechanism of DIO3OS in osteosarcoma.

Based on the TARGET database and GSE39055, the results indicated that the DIO3OS expression was up-regulated in osteosarcoma tissues and high expression of DIO3OS was significantly associated with poor prognosis for patients with osteosarcoma. The pan-cancer analysis result showed DIO3OS were up-regulated in OS, DLBC, PAAD, and THYM, distinguishing them from other types of cancer. The expression of DIO3OS up-regulated in osteosarcoma was validated between osteoblast and osteosarcoma in GSE12865 and further indicated between hFOB 1.19 cell line and osteosarcoma cell lines. According to the quantitative detection of DIO3OS in cell lines HOS, MG-63, U2OS, and SaoS-2, we selected two typical cell lines (U2OS and SaoS-2) with high DIO3OS expression for further experiments. The outcomes showed that knockdown DIO3OS decreased the ability of migration and invasion for cell lines U2OS and SaoS-2 in vitro. Therefore, DIO3OS may be a novel potential therapeutic target for osteosarcoma.

Furthermore, the potential role of DIO3OS in osteosarcoma was explored using functional enrichment analysis, and these outcomes included “extracellular structure organization”, “endoplasmic reticulum lumen”, “growth factor activity”, and “TGF-β signaling pathway”. Activation of the TGF-β signaling pathway plays an essential role in disease initiation and development, including proliferation, cell cycle progression, restoration of the immune response, invasion, and tumorigenesis [19]. In addition, several strategies targeting TGF-β signaling have been used in preclinical or clinical applications, particularly in end-stage cancer, including anti-ligand antisense oligonucleotides, antibodies targeting ligands or receptors, and targeting TGF-β-receptor kinase drugs [20–24]. In the present investigation, a PPI network was constructed to further investigate the relationship between DIO3OS and TGF-β signaling pathway. This co-expression analysis and KM analysis outcomes further indicated the inhibition of osteosarcoma migration and invasion by silencing DIO3OS expression, possibly through inhibiting the TGF-β signaling pathway. Then, downregulated DIO3OS expression significantly inhibited the phosphorylation of SMAD2 in osteosarcoma cell lines. Furthermore, after the combined use of DIO3OS silencing and TGF-β signaling pathway activator (TGF-β1), the results revealed that acting the TGF-β signaling pathway restored the affection of DIO3OS silencing on inhibiting the metastasis, migration, and invasion of osteosarcoma in vivo and in vitro.

Evaluating the comprehensive landscape of the osteosarcoma microenvironment may help clarify osteosarcoma response to immunotherapy, and newer immune-based treatments, such as inhibitors of immune checkpoint, may significantly improve the outcomes of patients with osteosarcoma [2, 31–33]. These results demonstrated that in high expression of DIO3OS group, the proportion of B cells memory, T cells gamma delta, and Macrophages M0 were up-regulated, and Monocytes were reduced. It was determined that DIO3OS may play a crucial role to regulated immune cell infiltration in osteosarcoma. The analysis of immune checkpoint gene expression constructed that CD200 and TNFRSF25 expression were suppressed in low DIO3OS expression group. Inhibiting immunosuppressive factor CD200 may suppress cancer stem cells and evade the immune system by inhibiting tolerogenic response [19, 20]. Therefore, knockout DIO3OS may be a novel immunotherapy for patients with osteosarcoma by inhibiting immune checkpoints (CD200 and TNFRSF25) [19–21].

As our results showed that DIO3OS is highly expressed in osteosarcoma cell lines and tissues compared to controls, these results indicated that DIO3OS can be used as a diagnostic biomarker for osteosarcoma. However, in the clinic, sarcoma diagnoses can be difficult to make in terms of distinguishing one subtype from another, not distinguishing normal tissue. Therefore, it is of greater significance to identify biomarkers that are subtype-specific. These outcomes of the TCGA-SARC cohort indicated that the DIO3OS expression was an excellent diagnostic biomarker for distinguishing osteosarcoma from lipomatous neoplasms, myomatous neoplasms, nerve sheath tumors, and synovial-like neoplasms. In addition, these outcomes of diagnosis and prognosis analysis in the validation cohort further indicated that expression levels of DIO3OS were an excellent diagnostic and prognostic biomarker for osteosarcoma. It is worth mentioning that DIO3OS is the first diagnostic biomarker identified to differentiate osteosarcoma from multiple subtypes of sarcoma.

There are some limitations in this research. First, the sample size of the control was smaller than the diseases in the present study, so in future research, we should keep the balance of the sample size. Second, prospective studies should be conducted in future studies to avoid analysis bias due to the retrospective of this research. Finally, we should further elucidate that the mechanism of DIO3OS accurately regulates the activity of the TFG-β signaling pathway for osteosarcoma metastasis and immune infiltration.

## Conclusions

These outcomes of the present study indicated that inhibiting the expression of DIO3OS sed the ability of metastasis, migration, and invasion of osteosarcoma cell lines and animal models as a novel regulator of TFG-β signaling pathway. Therefore, our experiments provided a novel insight into the molecular mechanism of metastasis in osteosarcoma. In addition, DIO3OS may be a novel immunotherapeutic target for osteosarcoma by suppressing immune checkpoints (CD200 and TNFRSF25) and is a novel diagnostic biomarker to distinguish osteosarcoma from multiple other subtypes of sarcoma. This research indicated DIO3OS as a potential diagnostic and prognostic biomarker of osteosarcoma, emphasizing its potential as a target of immunotherapy to improve the treatment of osteosarcoma.

### Supplementary Information

**Additional file 1.** Raw data.

**Additional file 2: Figure S1.** Differential expression of DIO3OS in pan-cancer.

**Additional file 3: Table S1.** Sequences of Ribo TM h-DIO3OS Smart Silencer. **Table S2.** Primers and sequences used in this study. **Table S3.** Baseline clinical characteristics of patients with osteosarcoma in validation cohort.

## Data Availability

The authors ensure the availability of supporting data and materials. The datasets analyzed for this study can be found in the XENA platform of USCS (http://xena.ucsc.edu/) and Gene Expression Omnibus (GEO, https://www.ncbi.nlm.nih.gov/geo/query/acc.cgi?acc=GSE39055 and https://www.ncbi.nlm.nih.gov/geo/query/acc.cgi?acc=GSE12865).

## Abbreviations

LincRNA: Long intergenic non-coding RNA
DIO3OS: DIO3 Opposite Strand Upstream RNA
UCSC: University California Santa Cruz
GEO: Gene Expression Omnibus
ROC: Reveiver operating characteristics
AUC: Area Under Curve
qRT-PCR: Quantitative real-time PCR
TGF-β: Transforming growth factor beta 1
CD200: CD200 molecule
TNFRSF25: TNF Receptor Superfamily Member
LncRNA: Long non-coding RNA
TP53INP1: Tumor Protein P53 Inducible Nuclear Protein 1
YY1: YIN-YANG-1 transcription factor
TNK2-AS1: Tyrosine Kinase Non Receptor Antisense RNA 1
WDR1: WD repeat domain 1
TARGET: Therapeutically Applicable Research To Generate Effective Treatments
GDC: Genomic data commons
OS: Overall survival
PFS: Progression-free survival
KM: Kaplan–Meier
GSEA: Gene set enrichment analysis
GO: Gene ontology
KEGG: Kyoto Encyclopedia of Genes and Genomes
PPI: Protein protein interaction
DEGs: Diffferentially expressed genes
TCGA: The Cancer Genome Atlas
GTEx: Genotype tissue expression
ATCC: American tissue culture collection
DMEM: Dulbecco’s modified Eagle’s medium
