# Supplementary figures and images for: Long intergenic non-coding RNA DIO3OS promotes osteosarcoma metastasis via activation of the TGF-β signaling pathway: a potential diagnostic and immunotherapeutic target for osteosarcoma (part 2 of 2)
Source: Cancer Cell Int. 2023 Sep 26;23:215. doi: 10.1186/s12935-023-03076-5 (PMC10521498; doi:10.1186/s12935-023-03076-5)

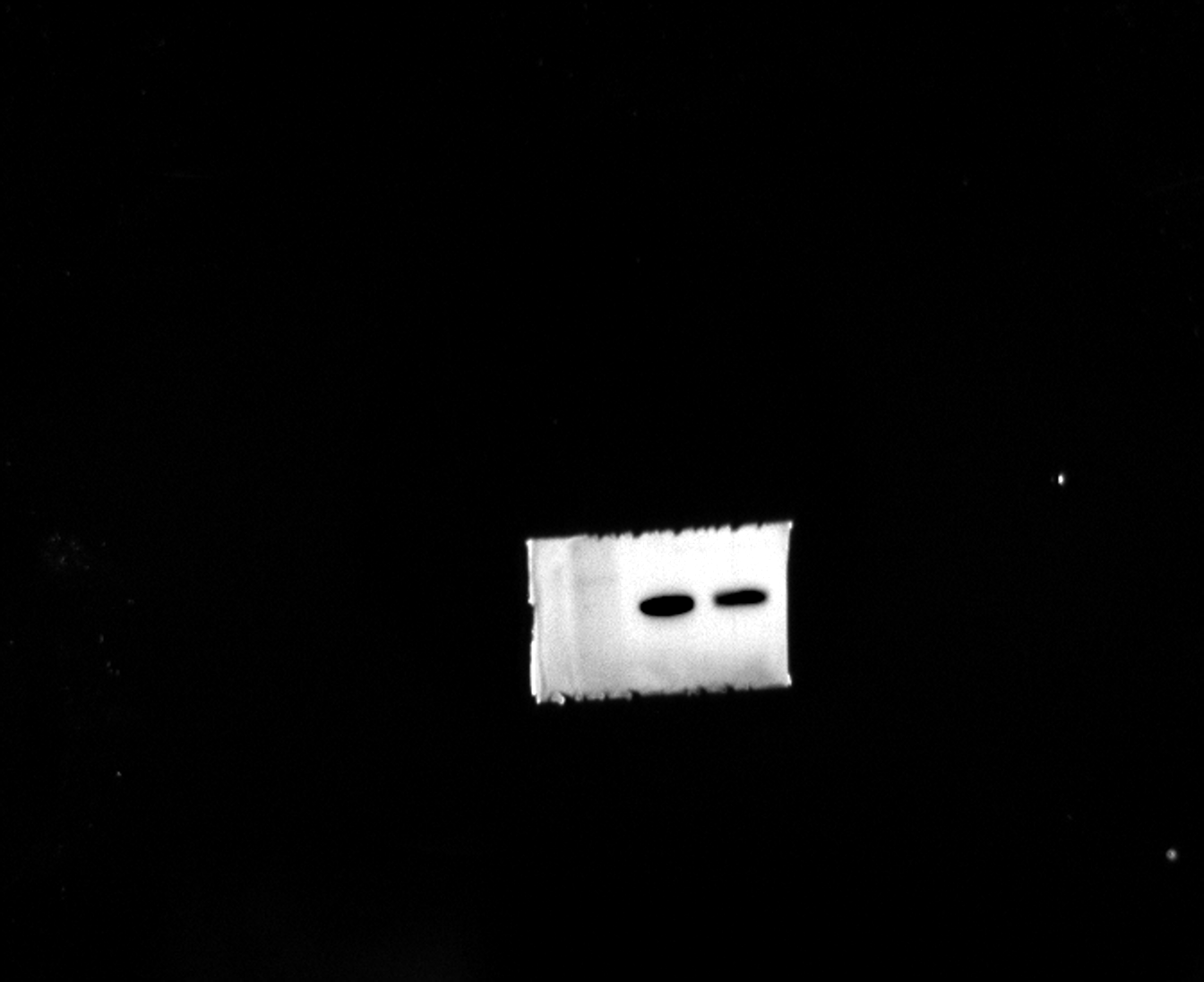

Supplement: Supplementary file 1 — Additional file 1. Raw data. [file 12935_2023_3076_MOESM1_ESM.zip › raw_data/figure6-gh1/Figure.6 Vimentin/raw_H1.Vimentin(τÖ╜σàëΓæáU2OS; ΓæíU2OS+si-DIO3OS).Tif]

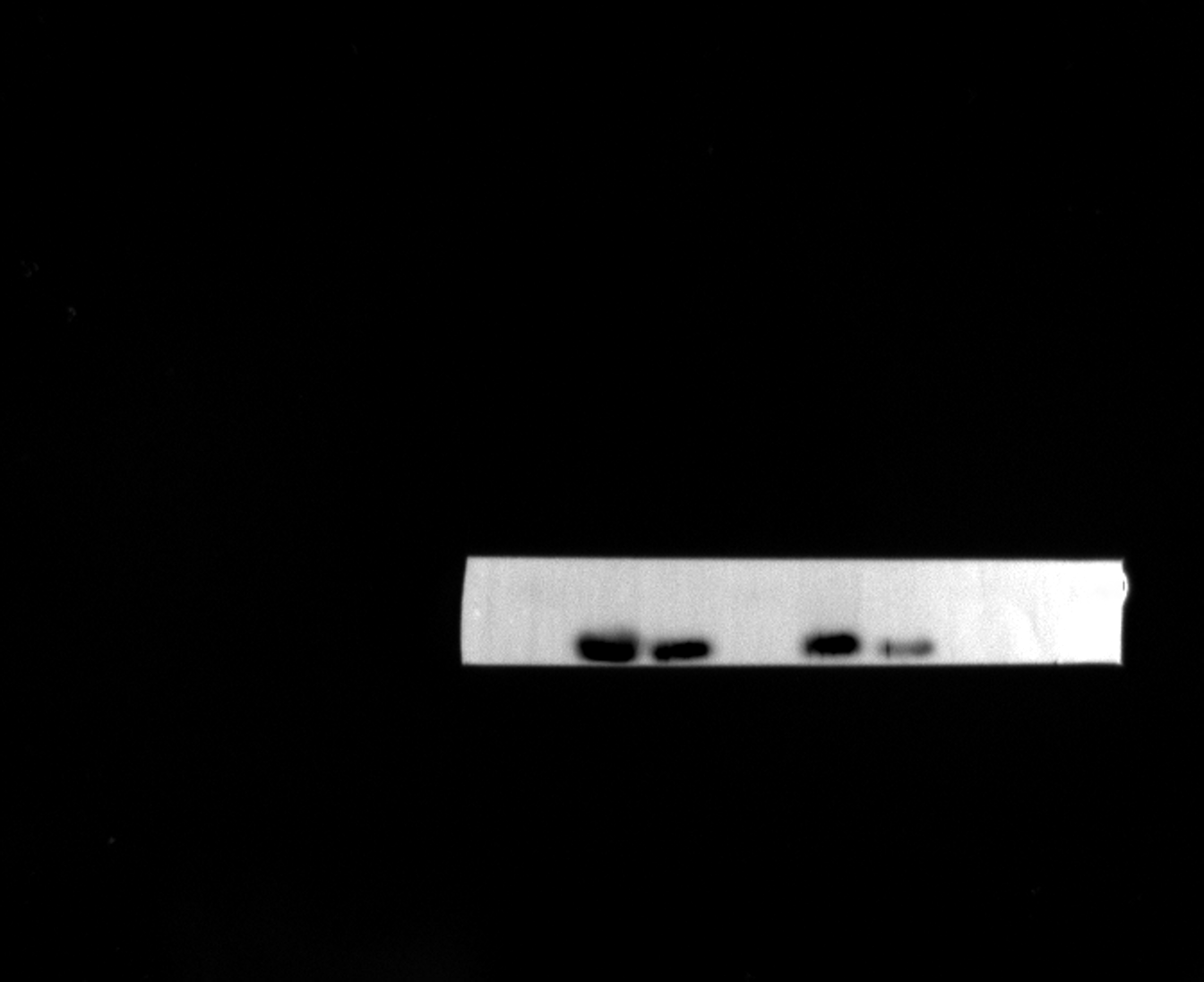

Supplement: Supplementary file 1 — Additional file 1. Raw data. [file 12935_2023_3076_MOESM1_ESM.zip › raw_data/figure6-gh1/Figure.6 Vimentin/raw_H2.Vimentin(τÖ╜σàëΓæáU2OS; ΓæíU2OS+si-DIO3OS).Tif]

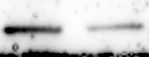

Supplement: Supplementary file 1 — Additional file 1. Raw data. [file 12935_2023_3076_MOESM1_ESM.zip › raw_data/figure6-gh1/Figure.6 Vimentin/final_G Vimentin.tif]

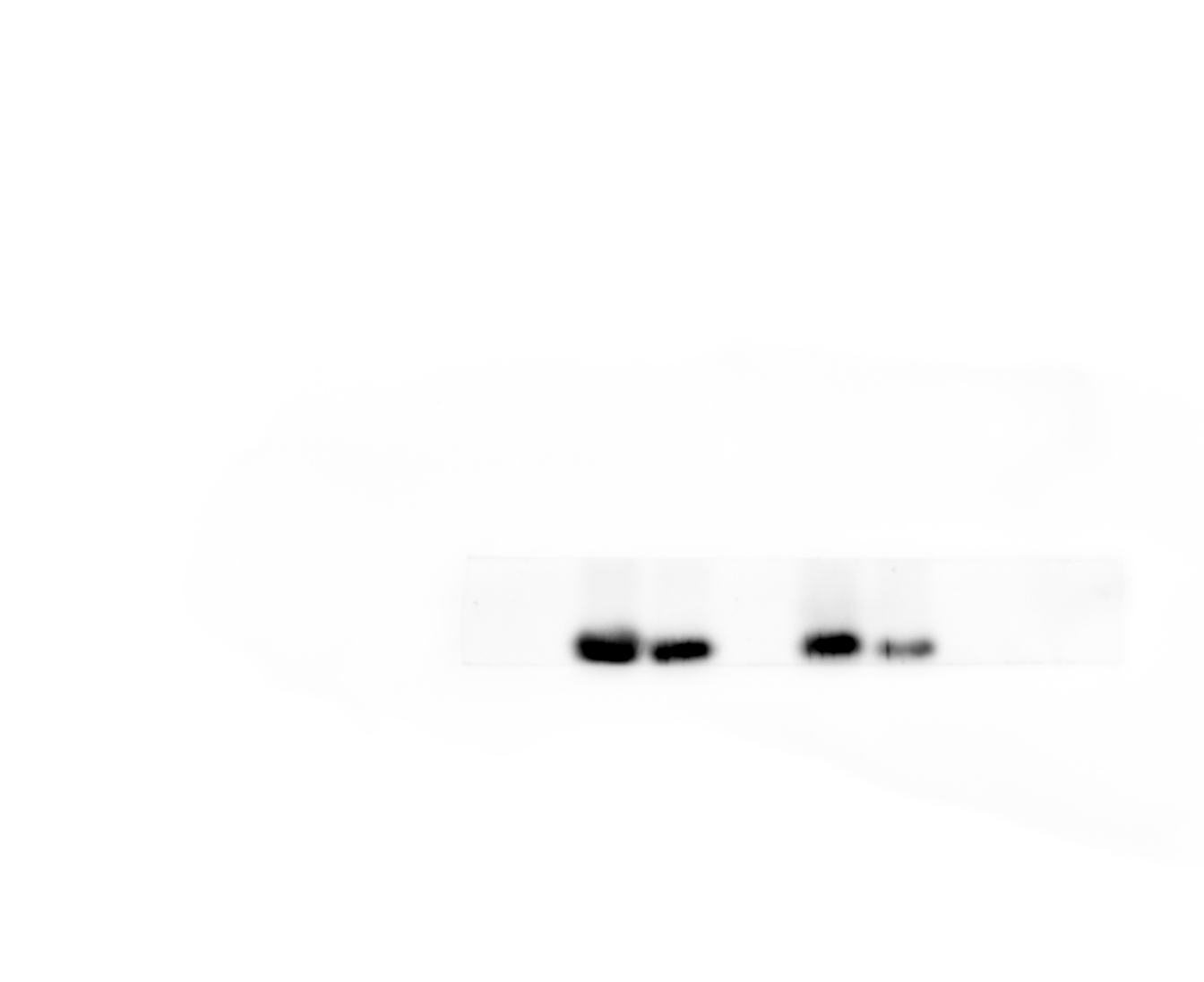

Supplement: Supplementary file 1 — Additional file 1. Raw data. [file 12935_2023_3076_MOESM1_ESM.zip › raw_data/figure6-gh1/Figure.6 Vimentin/raw_H2.Vimentin(ΓæáU2OS; ΓæíU2OS+si-DIO3OS).Tif]

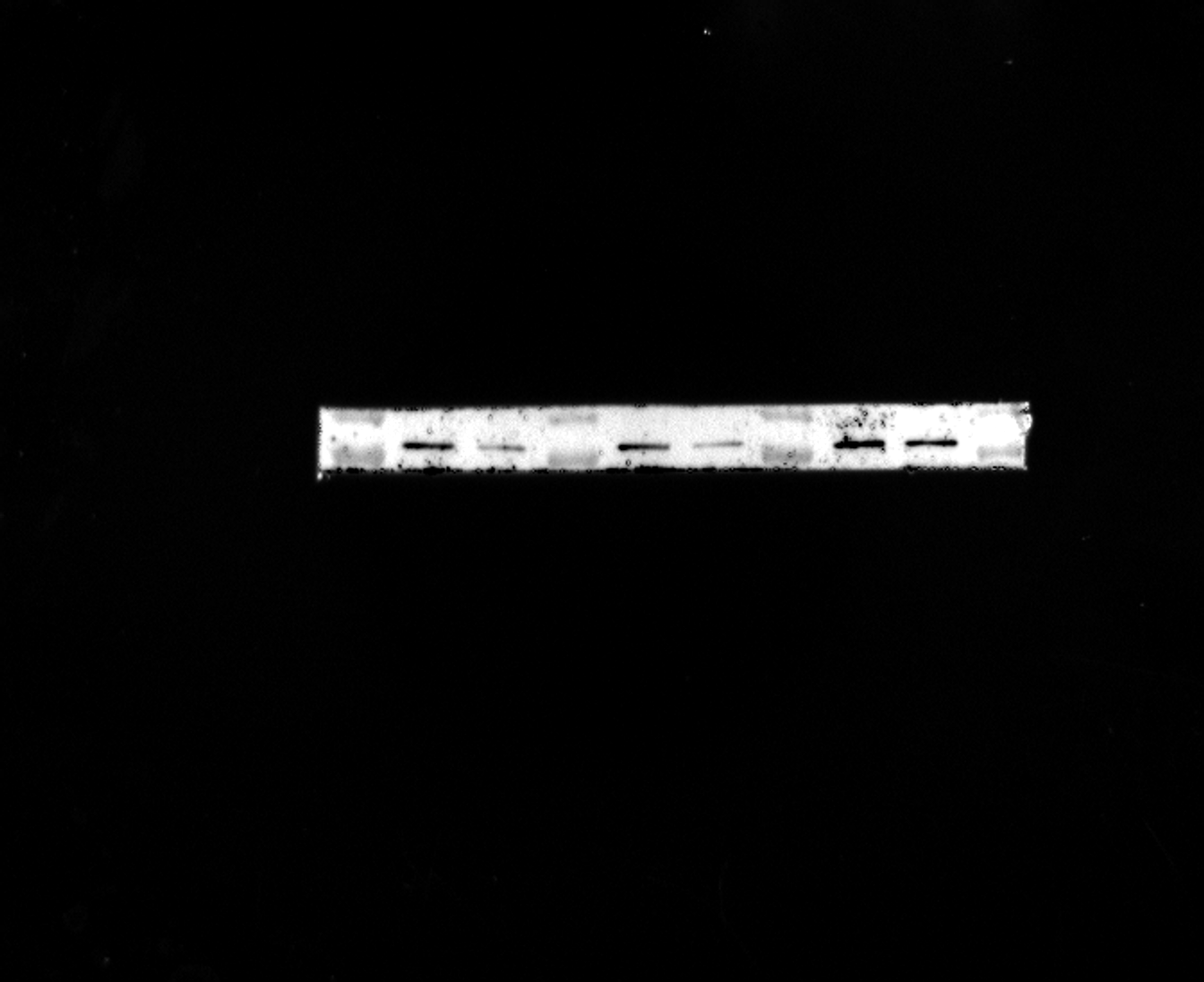

Supplement: Supplementary file 1 — Additional file 1. Raw data. [file 12935_2023_3076_MOESM1_ESM.zip › raw_data/figure6-gh1/Figure.6 Vimentin/raw_G.Vimentin(τÖ╜σàëΓæáSaoS-2+si-NC; ΓæíSaoS-2+si-DIO3OS).Tif]

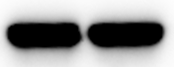

Supplement: Supplementary file 1 — Additional file 1. Raw data. [file 12935_2023_3076_MOESM1_ESM.zip › raw_data/figure6-gh1/Figure.6 GAPDH/final_G GAPDH.tif]

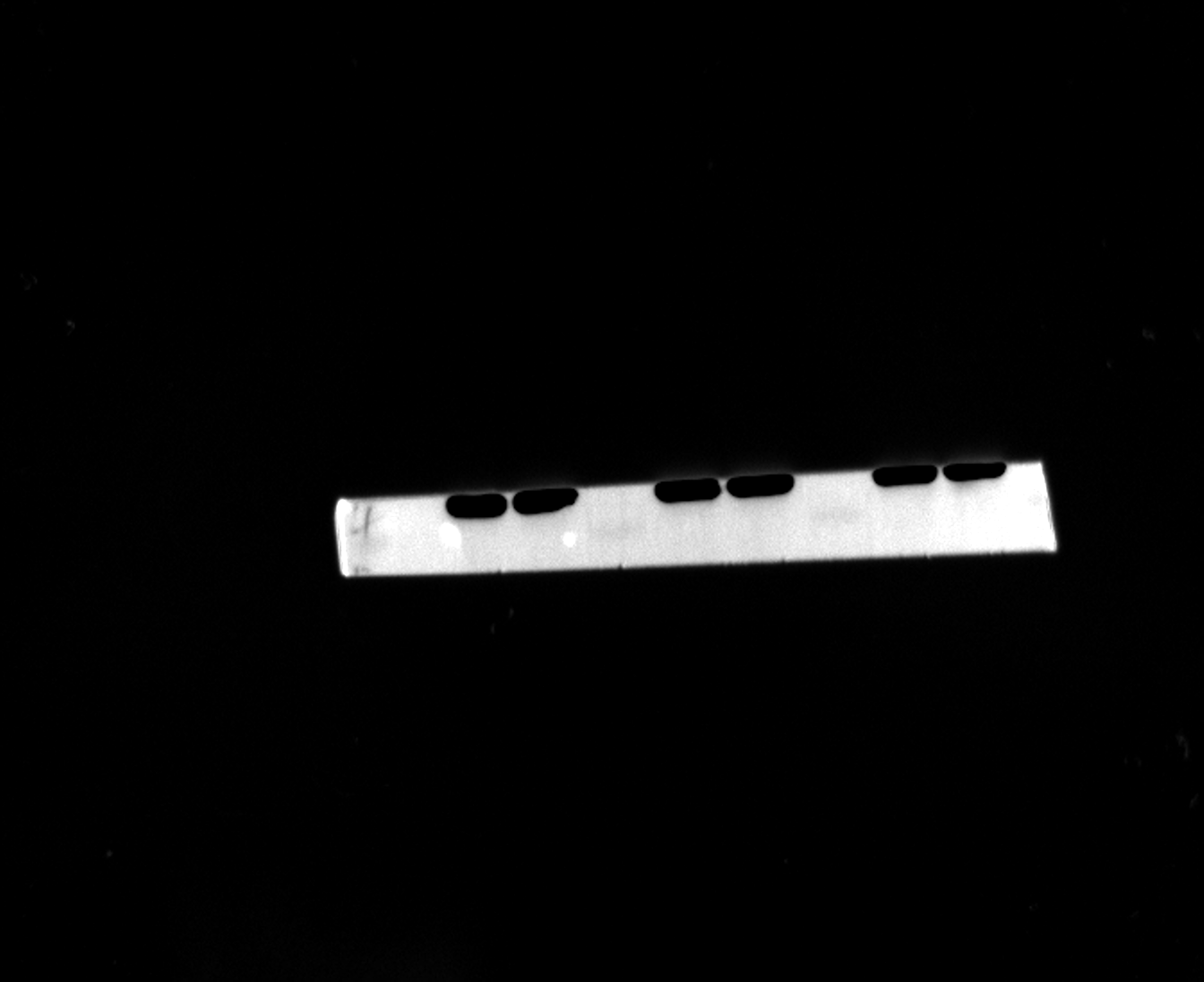

Supplement: Supplementary file 1 — Additional file 1. Raw data. [file 12935_2023_3076_MOESM1_ESM.zip › raw_data/figure6-gh1/Figure.6 GAPDH/rawG.GAPDH(τÖ╜σàëΓæáSaos2; ΓæíSaos2+si-DIO3OS).Tif]

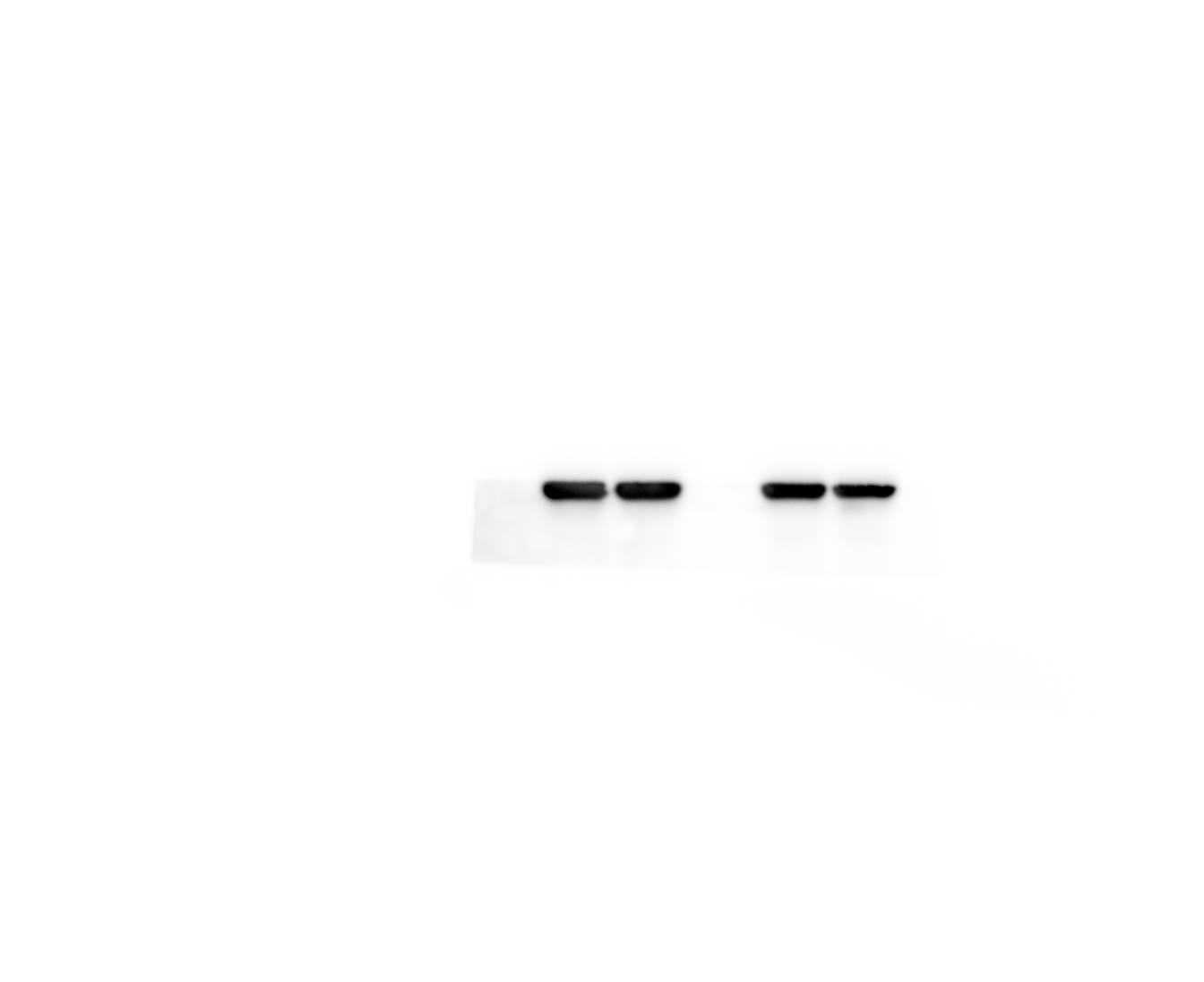

Supplement: Supplementary file 1 — Additional file 1. Raw data. [file 12935_2023_3076_MOESM1_ESM.zip › raw_data/figure6-gh1/Figure.6 GAPDH/raw_H2.GAPDH(ΓæáU2OS; ΓæíU2OS+si-DIO3OS).Tif]

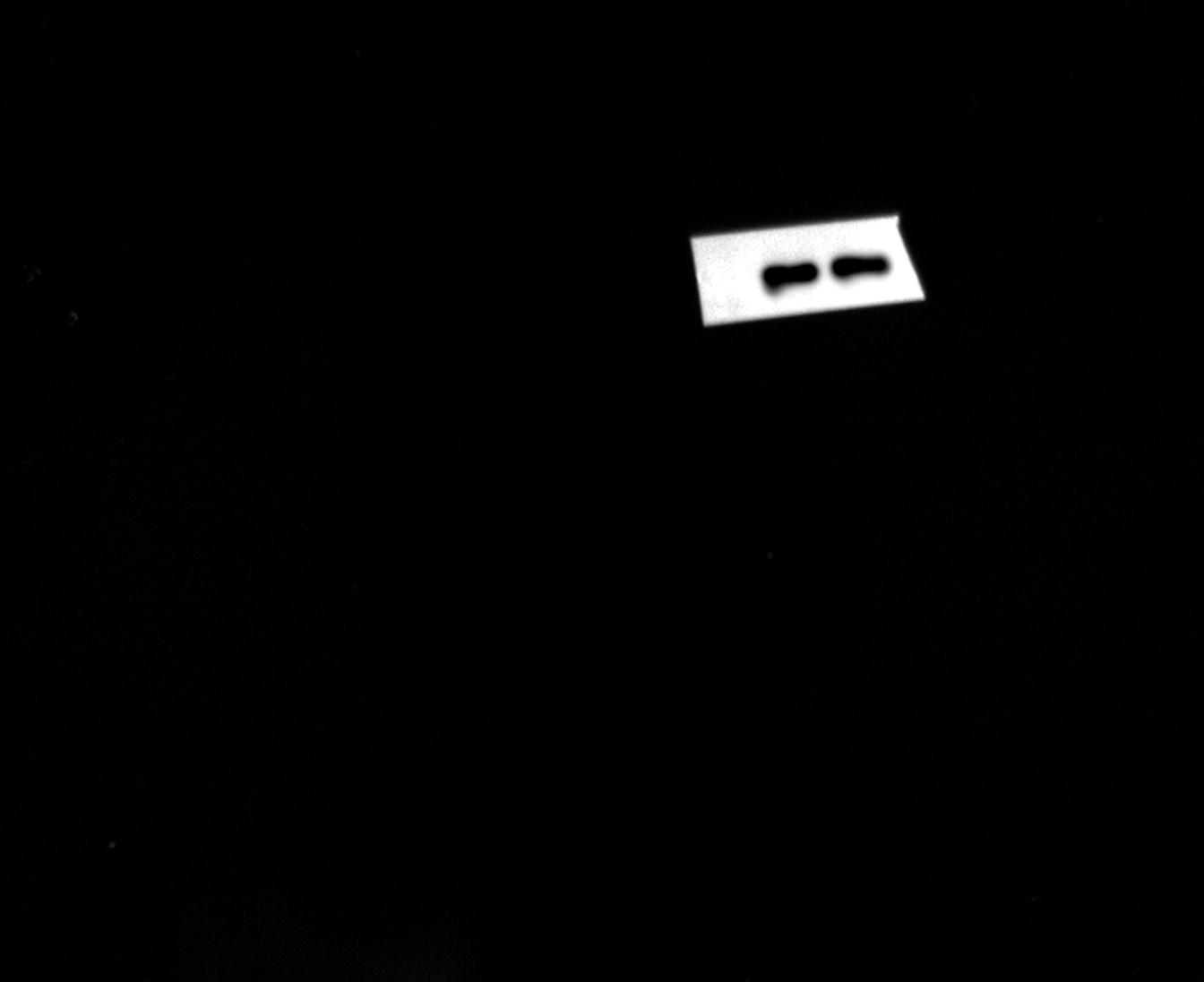

Supplement: Supplementary file 1 — Additional file 1. Raw data. [file 12935_2023_3076_MOESM1_ESM.zip › raw_data/figure6-gh1/Figure.6 GAPDH/raw_H1.GAPDH(τÖ╜σàëΓæáU2OS; ΓæíU2OS+si-DIO3OS).Tif]

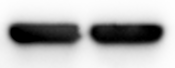

Supplement: Supplementary file 1 — Additional file 1. Raw data. [file 12935_2023_3076_MOESM1_ESM.zip › raw_data/figure6-gh1/Figure.6 GAPDH/final_H GAPDH.tif]

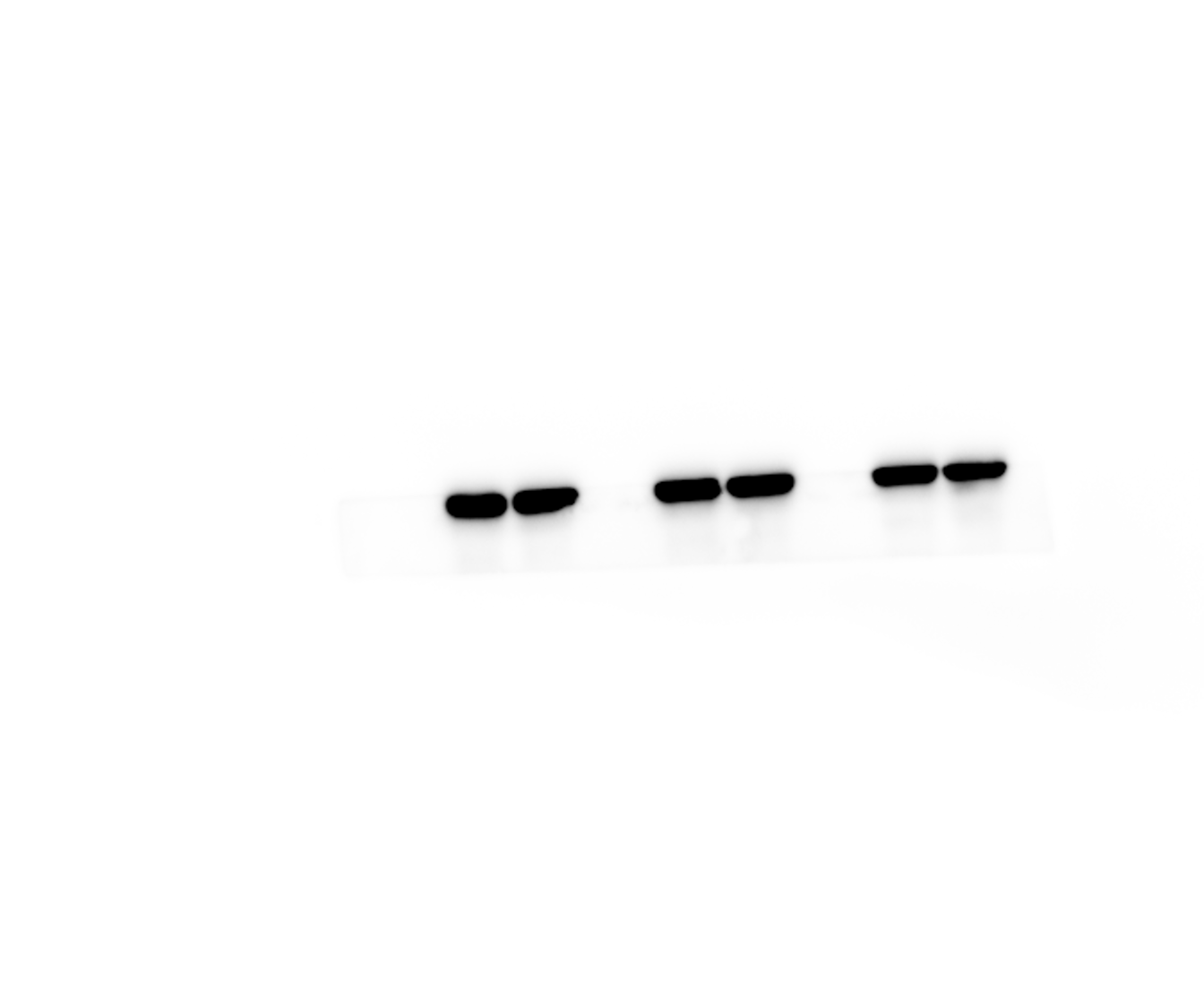

Supplement: Supplementary file 1 — Additional file 1. Raw data. [file 12935_2023_3076_MOESM1_ESM.zip › raw_data/figure6-gh1/Figure.6 GAPDH/raw_G.GAPDH(ΓæáSaos2; ΓæíSaos2+si-DIO3OS).Tif]

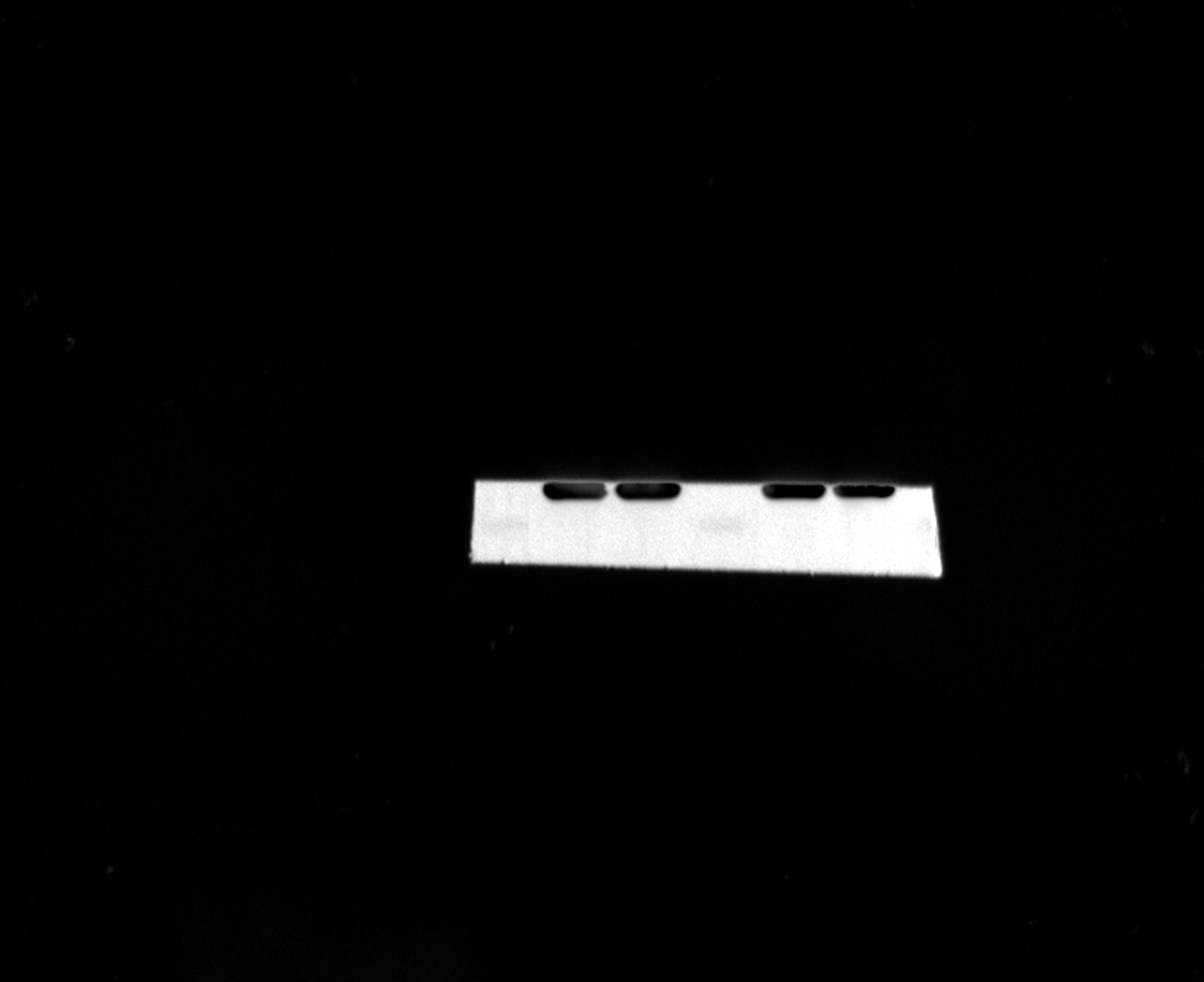

Supplement: Supplementary file 1 — Additional file 1. Raw data. [file 12935_2023_3076_MOESM1_ESM.zip › raw_data/figure6-gh1/Figure.6 GAPDH/raw_H2.GAPDH(τÖ╜σàëΓæáU2OS; ΓæíU2OS+si-DIO3OS).Tif]

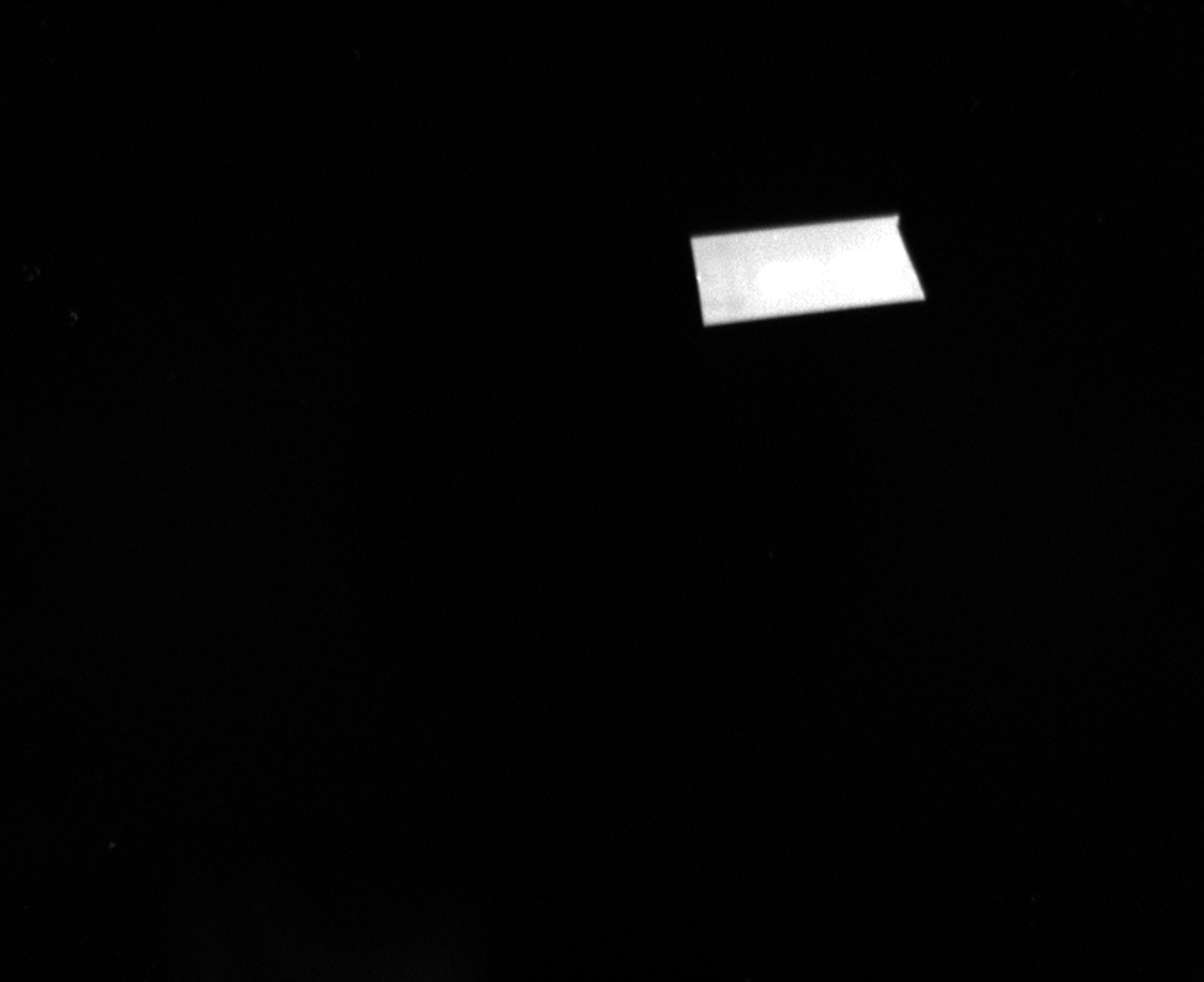

Supplement: Supplementary file 1 — Additional file 1. Raw data. [file 12935_2023_3076_MOESM1_ESM.zip › raw_data/figure6-gh1/Figure.6 GAPDH/raw_H1.GAPDH(ΓæáU2OS; ΓæíU2OS+si-DIO3OS).Tif]

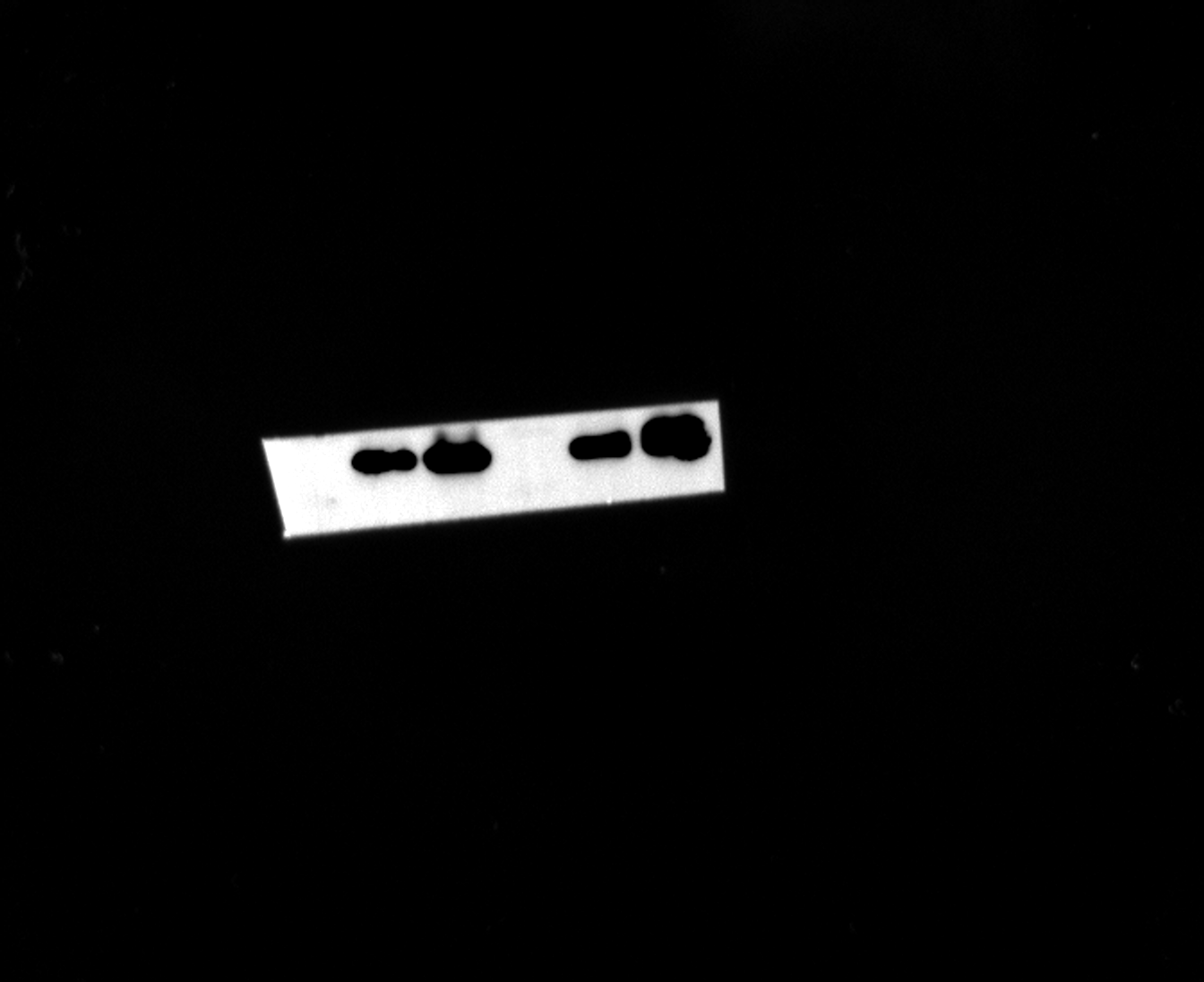

Supplement: Supplementary file 1 — Additional file 1. Raw data. [file 12935_2023_3076_MOESM1_ESM.zip › raw_data/figure6-gh1/Figure.6 E-cad/raw_H2.E-cad(τÖ╜σàëΓæáU2OS; ΓæíU2OS+si-DIO3OS).Tif]

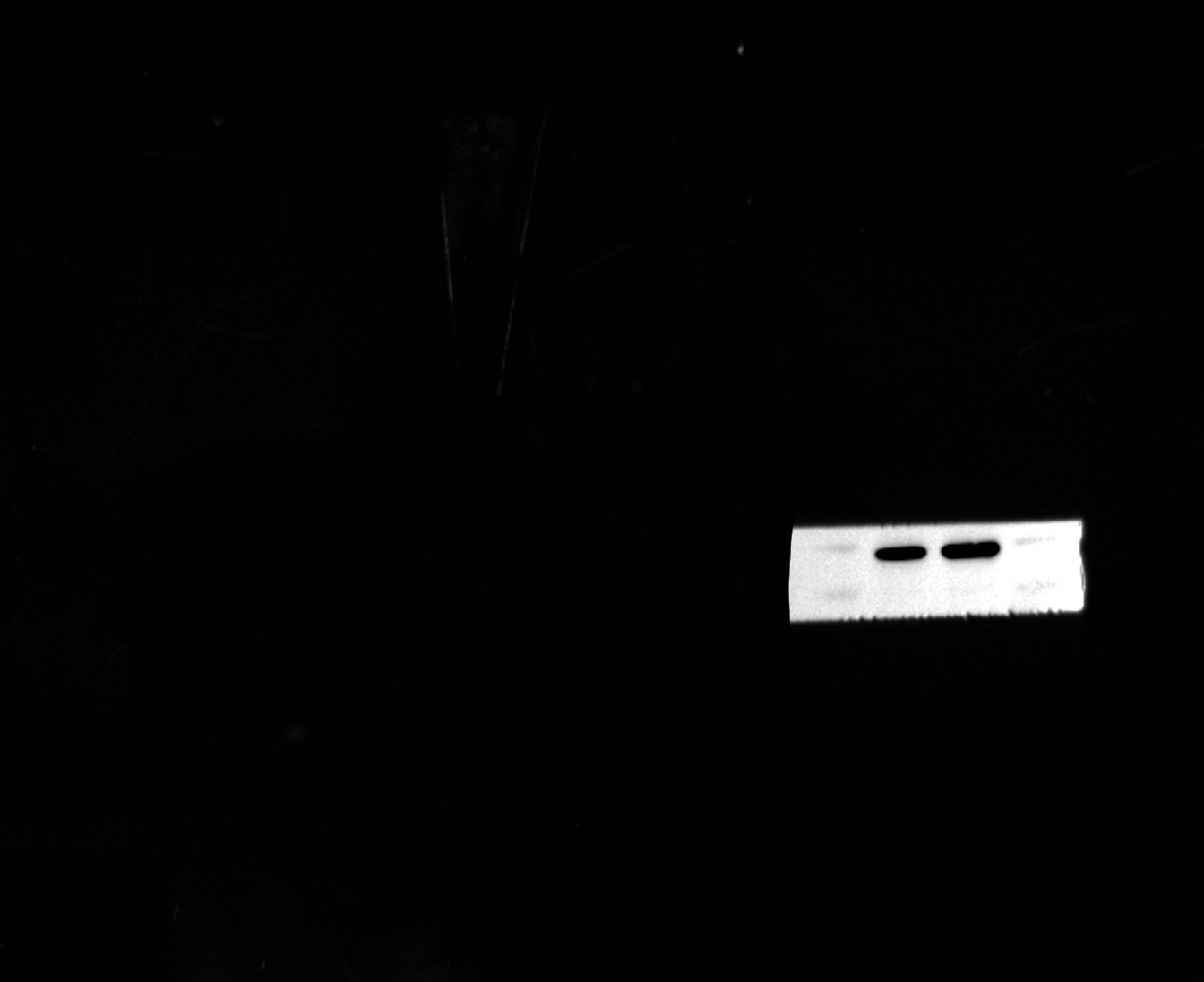

Supplement: Supplementary file 1 — Additional file 1. Raw data. [file 12935_2023_3076_MOESM1_ESM.zip › raw_data/figure6-gh1/Figure.6 E-cad/raw_H1.E-cad(τÖ╜σàëΓæáU2OS; ΓæíU2OS+si-DIO3OS).Tif]

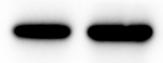

Supplement: Supplementary file 1 — Additional file 1. Raw data. [file 12935_2023_3076_MOESM1_ESM.zip › raw_data/figure6-gh1/Figure.6 E-cad/final_H E-cad.tif]

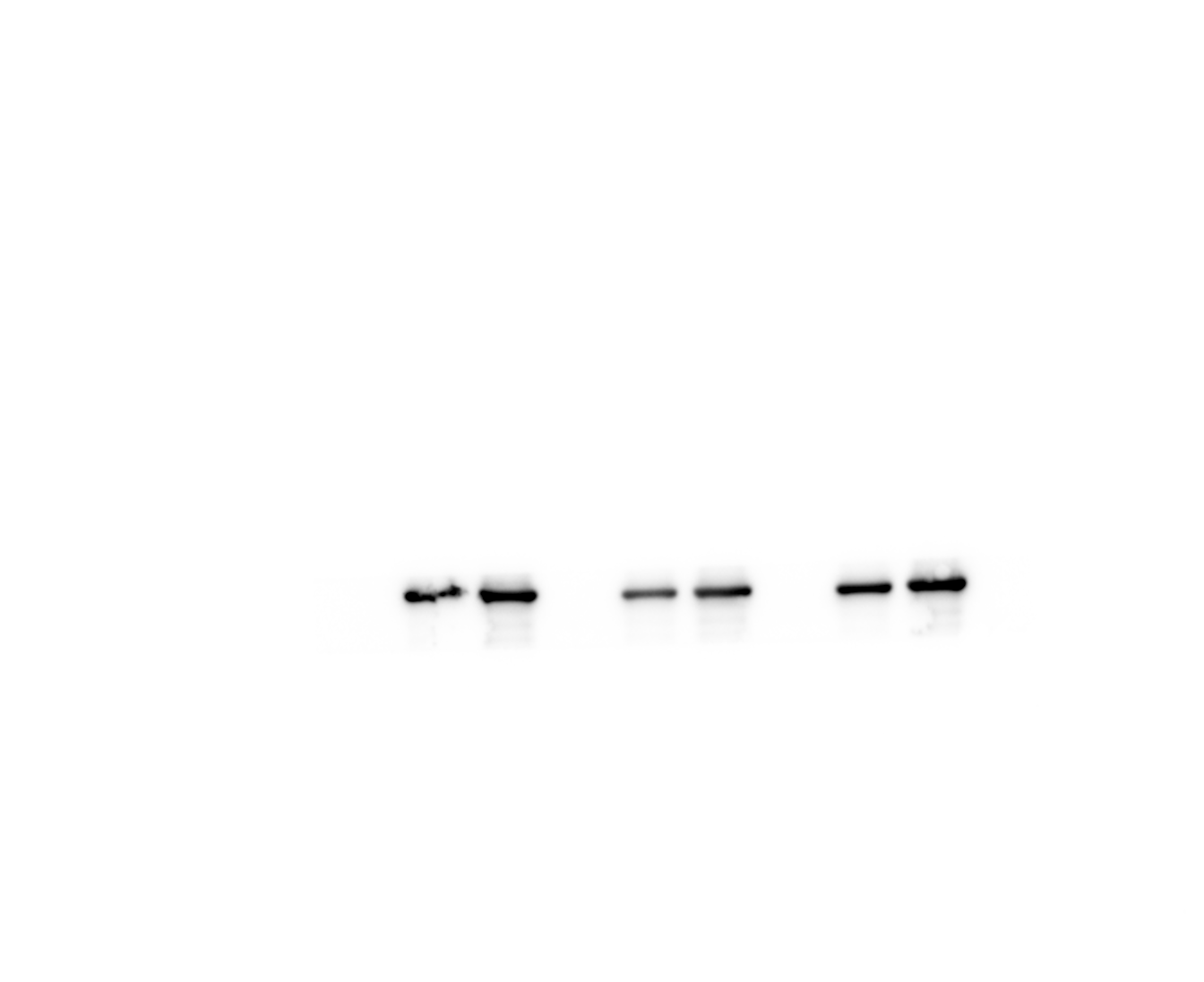

Supplement: Supplementary file 1 — Additional file 1. Raw data. [file 12935_2023_3076_MOESM1_ESM.zip › raw_data/figure6-gh1/Figure.6 E-cad/raw_G.E-cad(ΓæáSaoS-2+si-NC; ΓæíSaoS-2+si-DIO3OS).Tif]

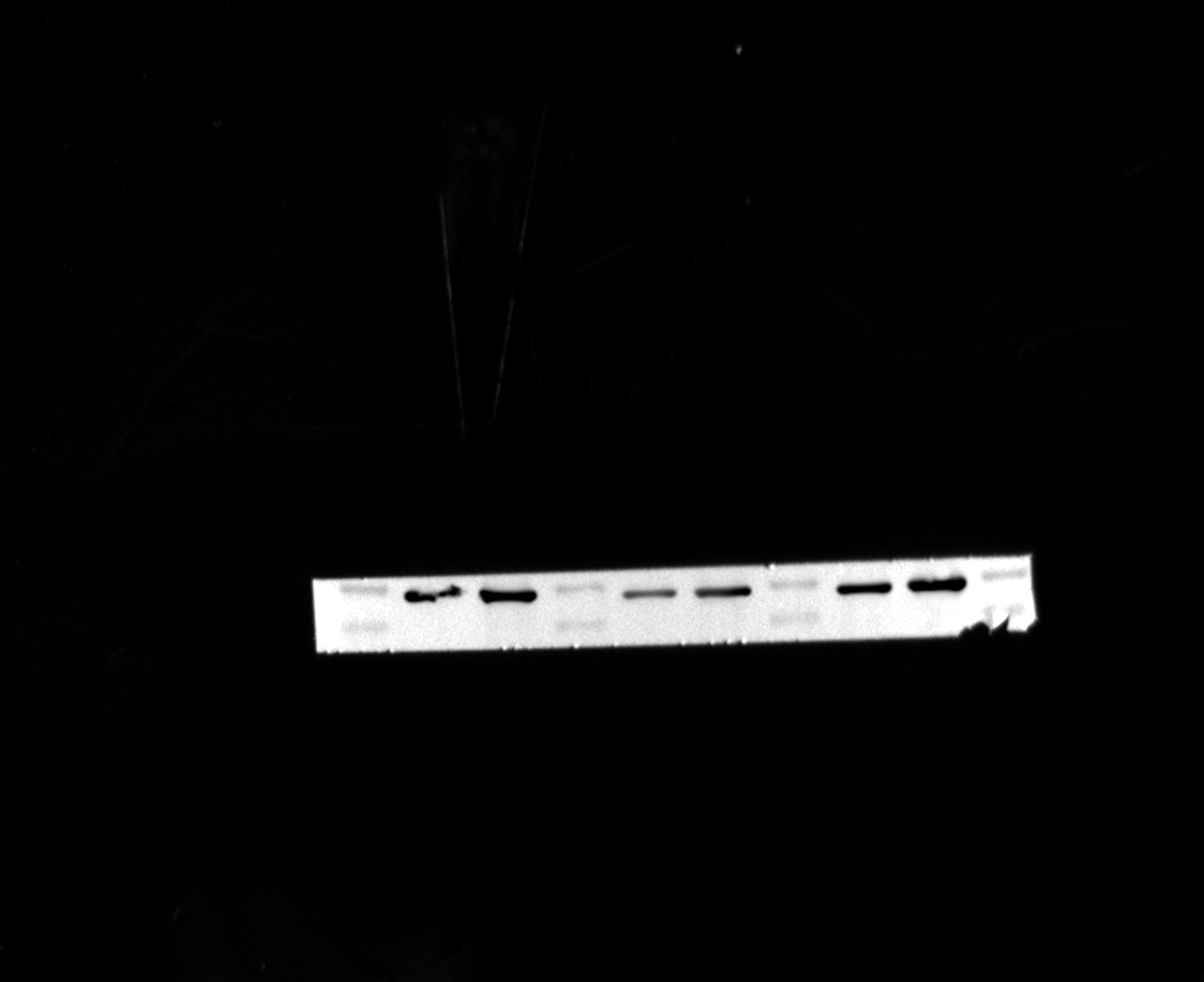

Supplement: Supplementary file 1 — Additional file 1. Raw data. [file 12935_2023_3076_MOESM1_ESM.zip › raw_data/figure6-gh1/Figure.6 E-cad/raw_G.E-cadτÖ╜σàë(ΓæáSaoS-2+si-NC; ΓæíSaoS-2+si-DIO3OS∩╝ë.Tif]

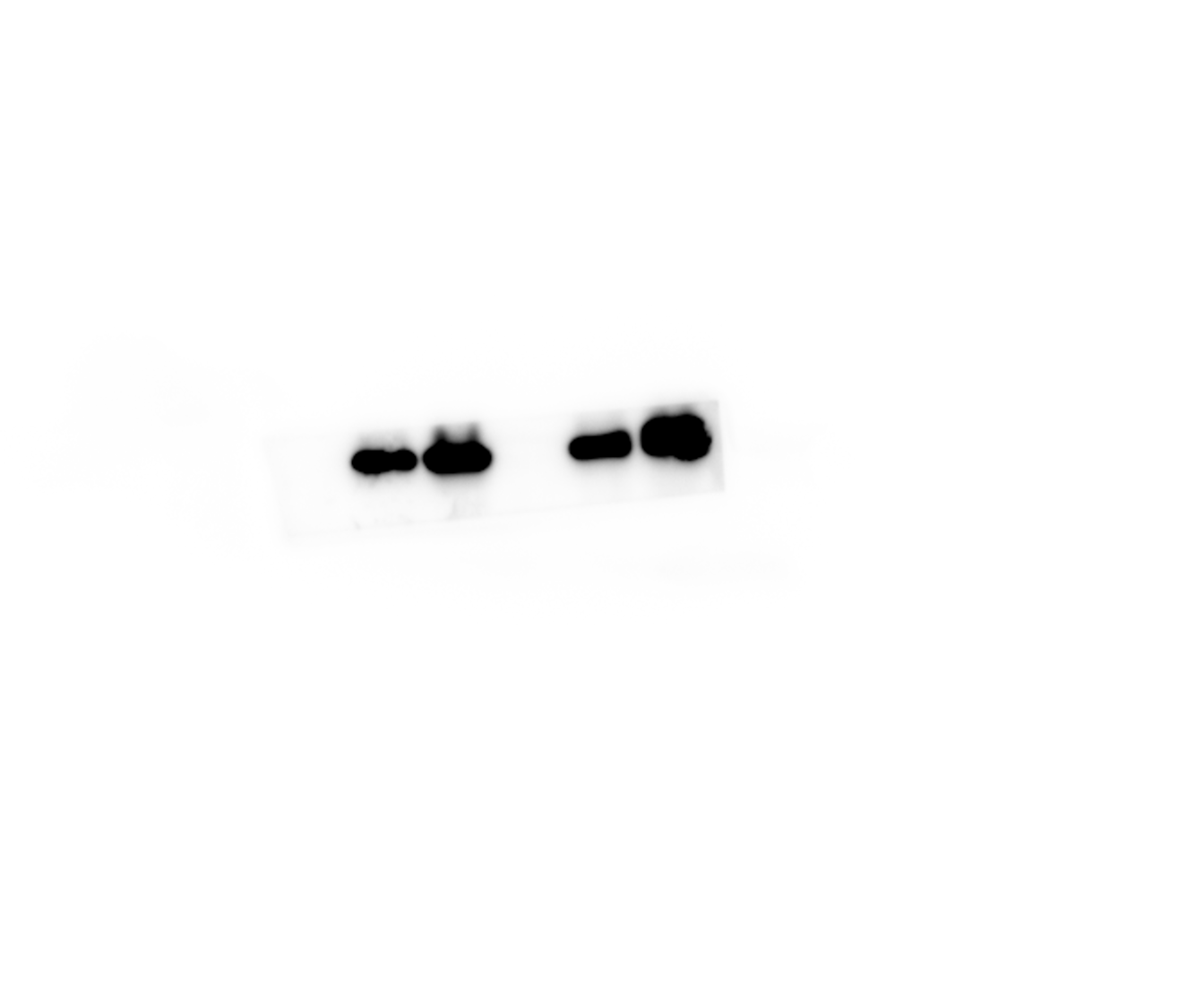

Supplement: Supplementary file 1 — Additional file 1. Raw data. [file 12935_2023_3076_MOESM1_ESM.zip › raw_data/figure6-gh1/Figure.6 E-cad/raw_H2.E-cad(ΓæáU2OS; ΓæíU2OS+si-DIO3OS).Tif]

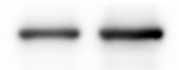

Supplement: Supplementary file 1 — Additional file 1. Raw data. [file 12935_2023_3076_MOESM1_ESM.zip › raw_data/figure6-gh1/Figure.6 E-cad/final_G E-cad.tif]

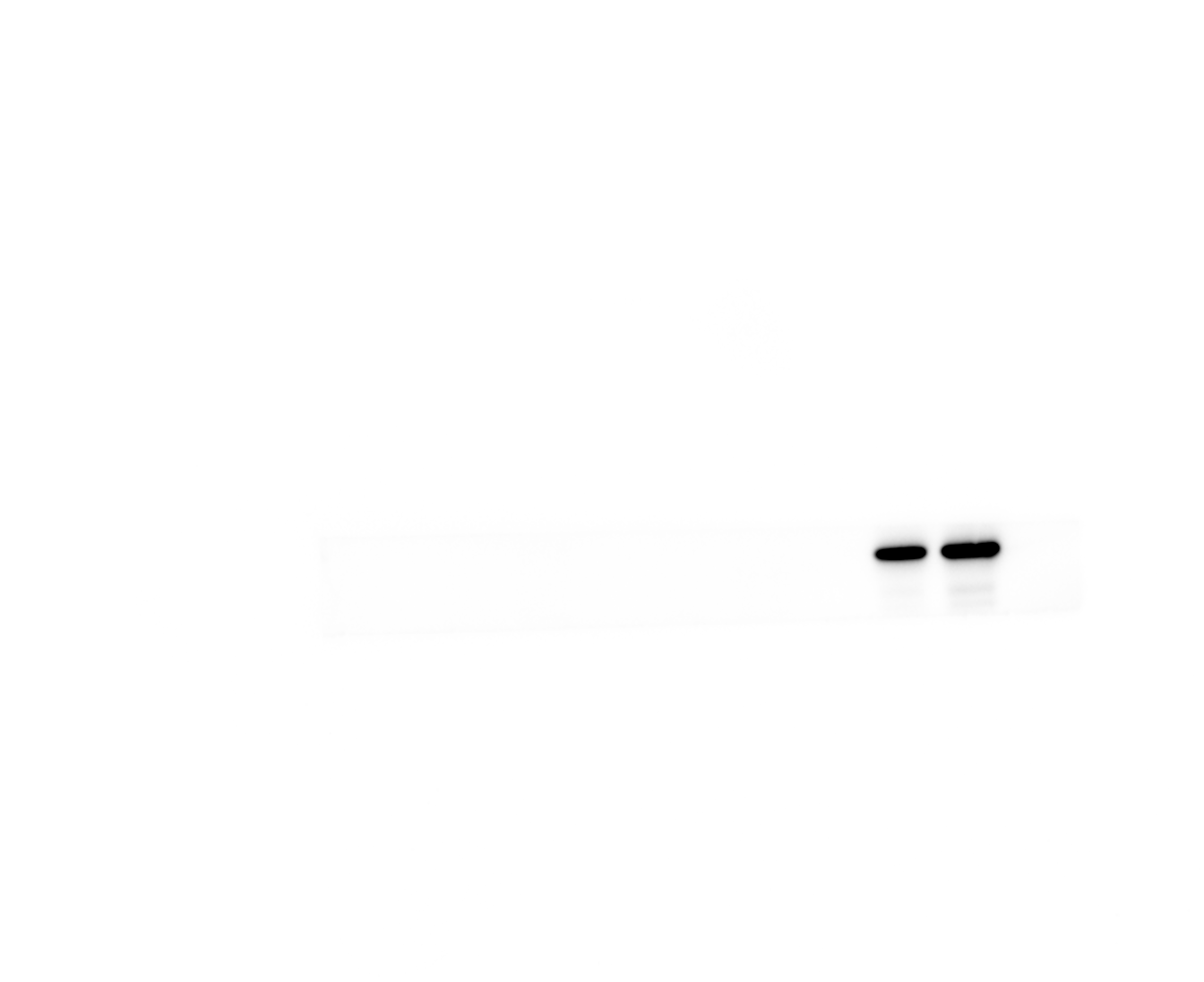

Supplement: Supplementary file 1 — Additional file 1. Raw data. [file 12935_2023_3076_MOESM1_ESM.zip › raw_data/figure6-gh1/Figure.6 E-cad/raw_H1.E-cad(ΓæáU2OS; ΓæíU2OS+si-DIO3OS).Tif]

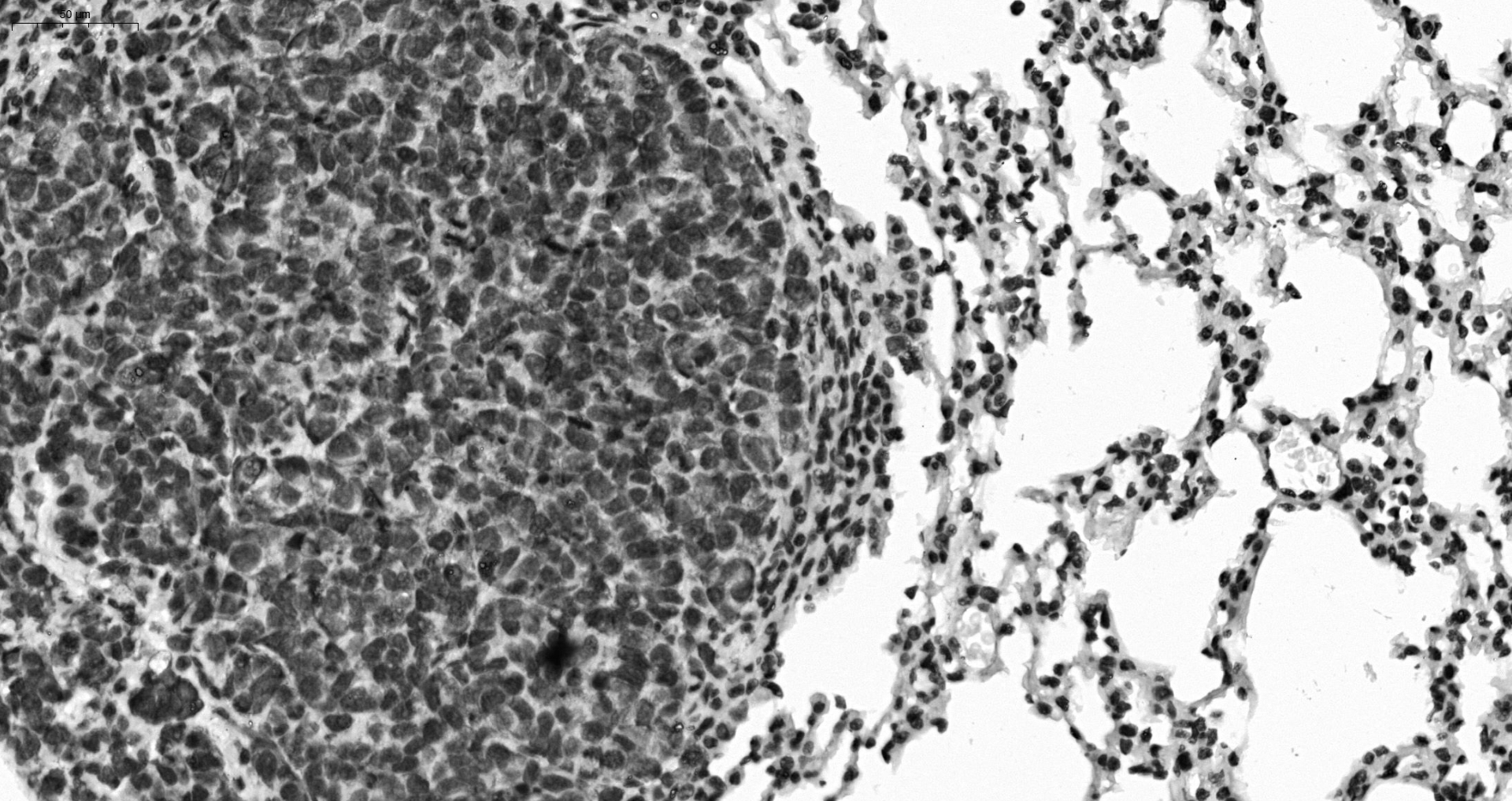

Supplement: Supplementary file 1 — Additional file 1. Raw data. [file 12935_2023_3076_MOESM1_ESM.zip › raw_data/IHC/vimentin/ΓæáSaoS-2+si-NC/1-2 ΓæáSaoS-2+si-NC.tif]

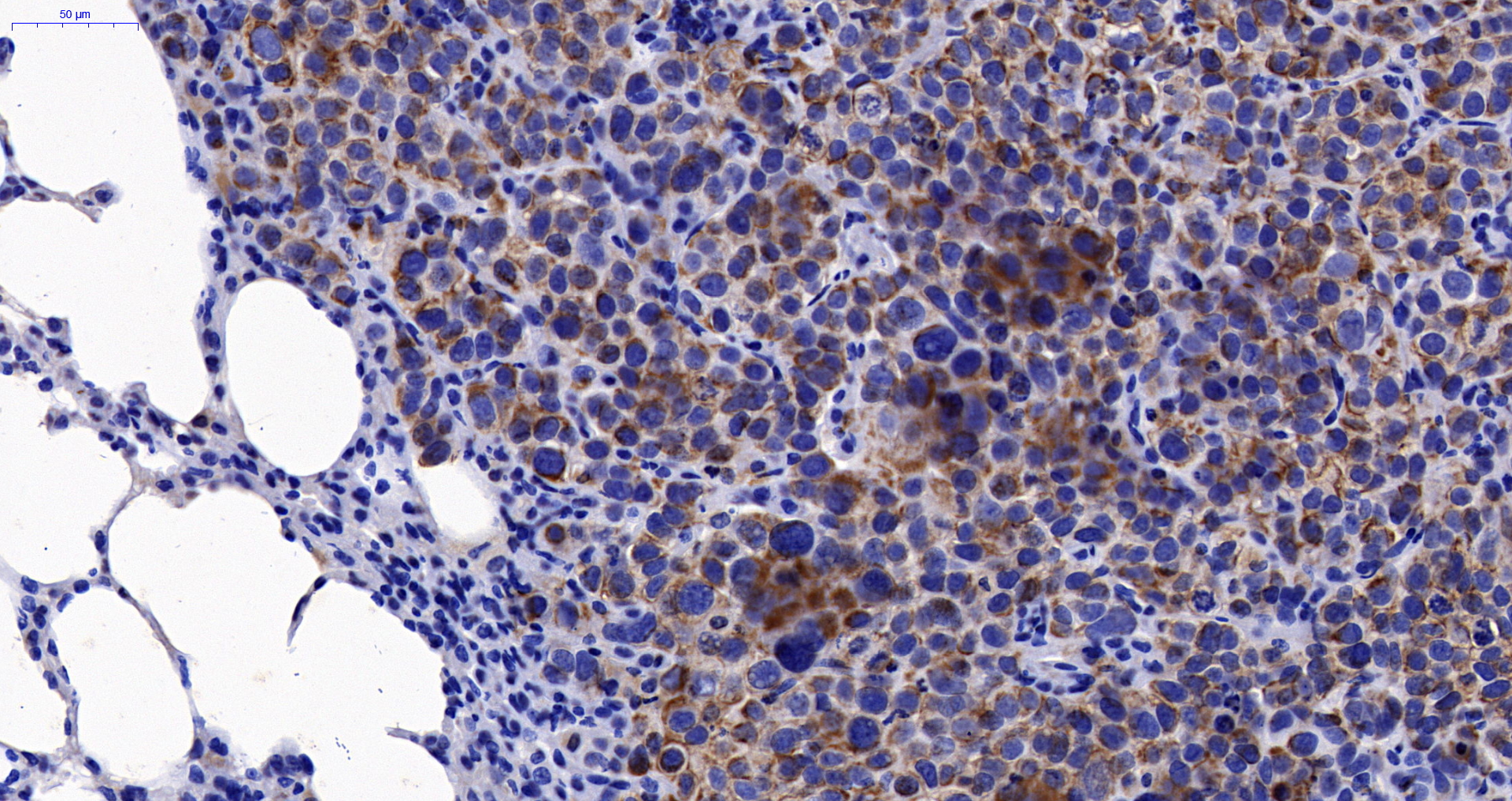

Supplement: Supplementary file 1 — Additional file 1. Raw data. [file 12935_2023_3076_MOESM1_ESM.zip › raw_data/IHC/vimentin/ΓæáSaoS-2+si-NC/1-1 ΓæáSaoS-2+si-NC.jpg]

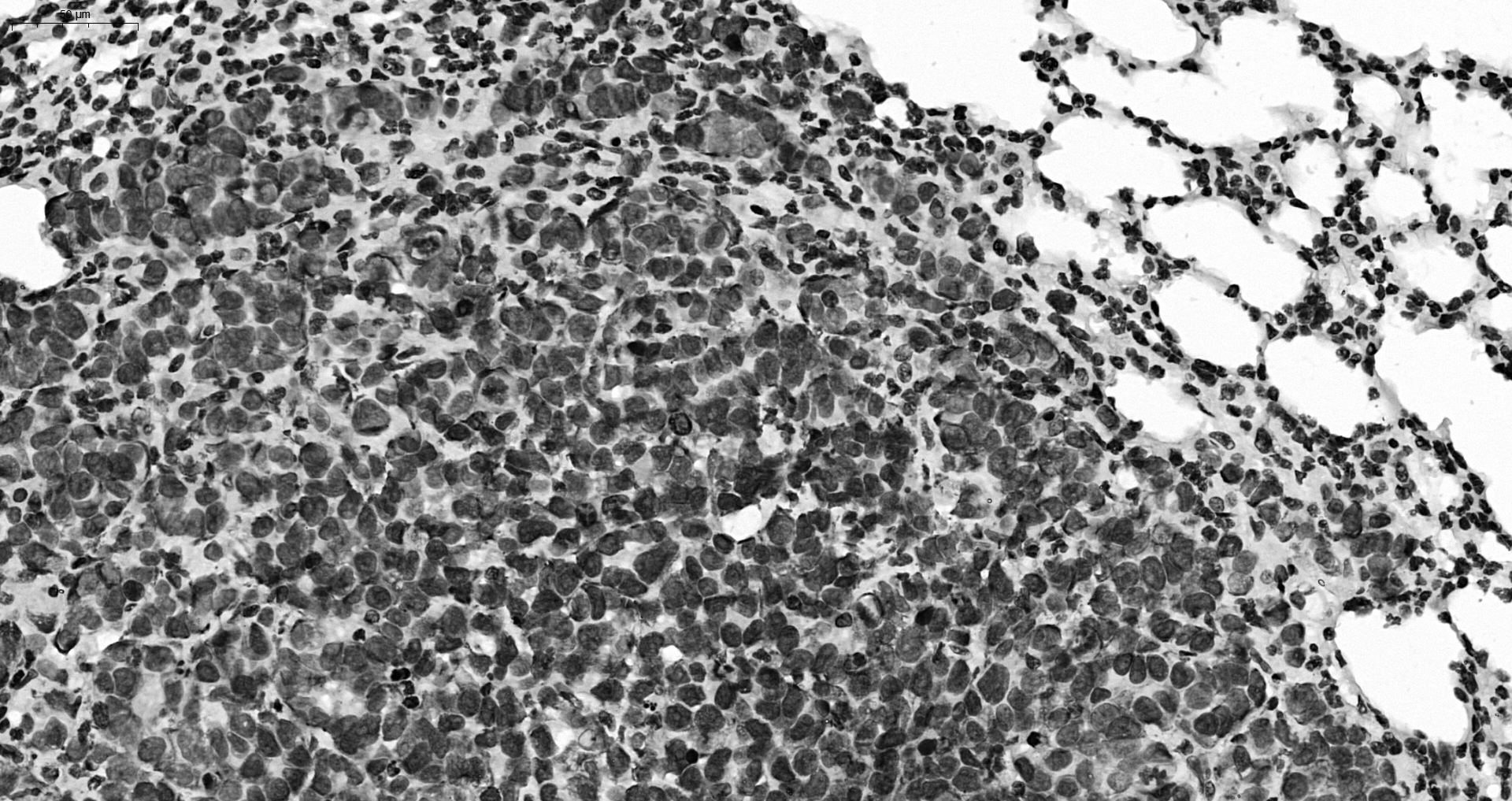

Supplement: Supplementary file 1 — Additional file 1. Raw data. [file 12935_2023_3076_MOESM1_ESM.zip › raw_data/IHC/vimentin/ΓæáSaoS-2+si-NC/3-2 ΓæáSaoS-2+si-NC.tif]

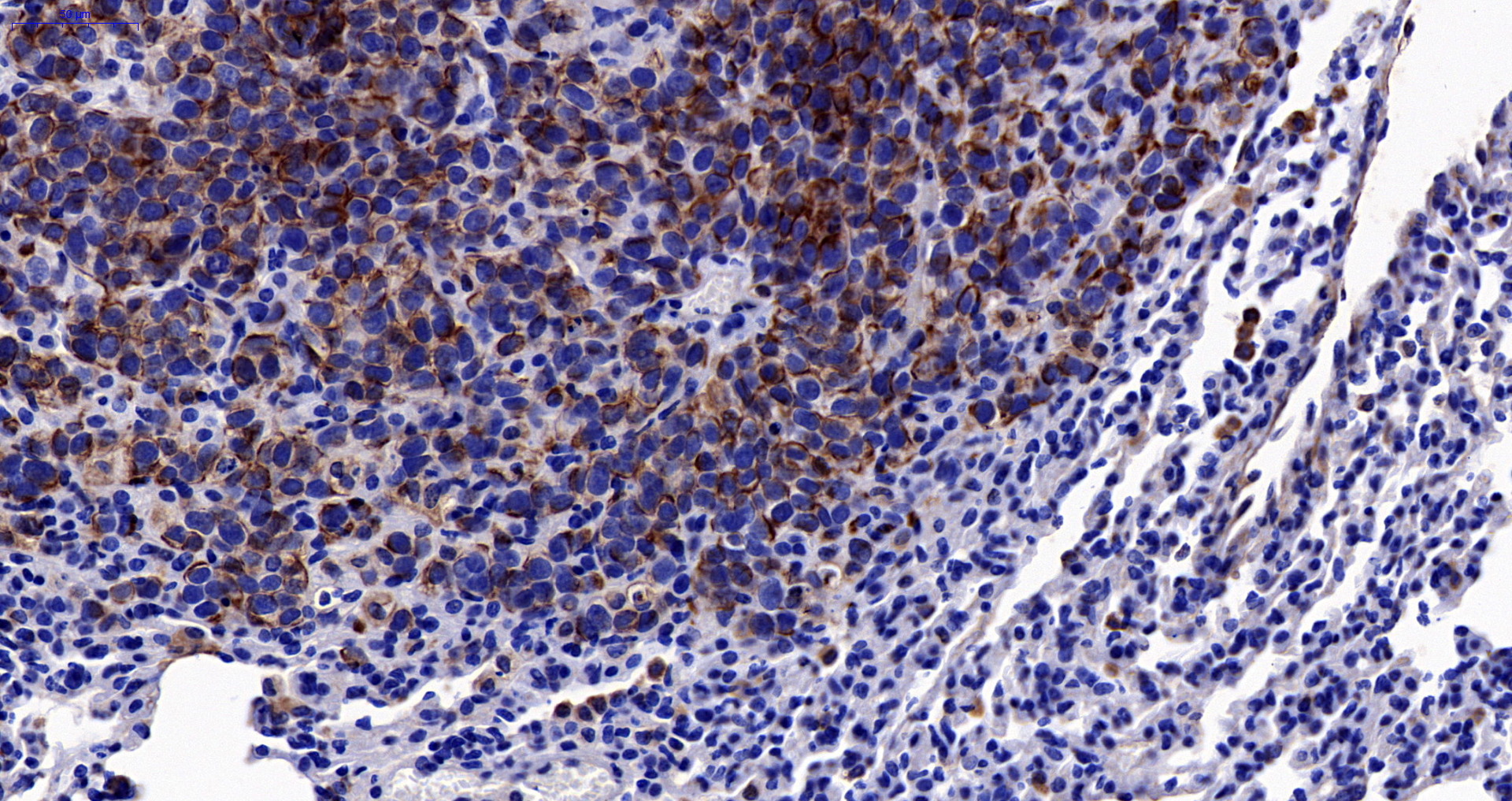

Supplement: Supplementary file 1 — Additional file 1. Raw data. [file 12935_2023_3076_MOESM1_ESM.zip › raw_data/IHC/vimentin/ΓæáSaoS-2+si-NC/2-1 ΓæáSaoS-2+si-NC.jpg]

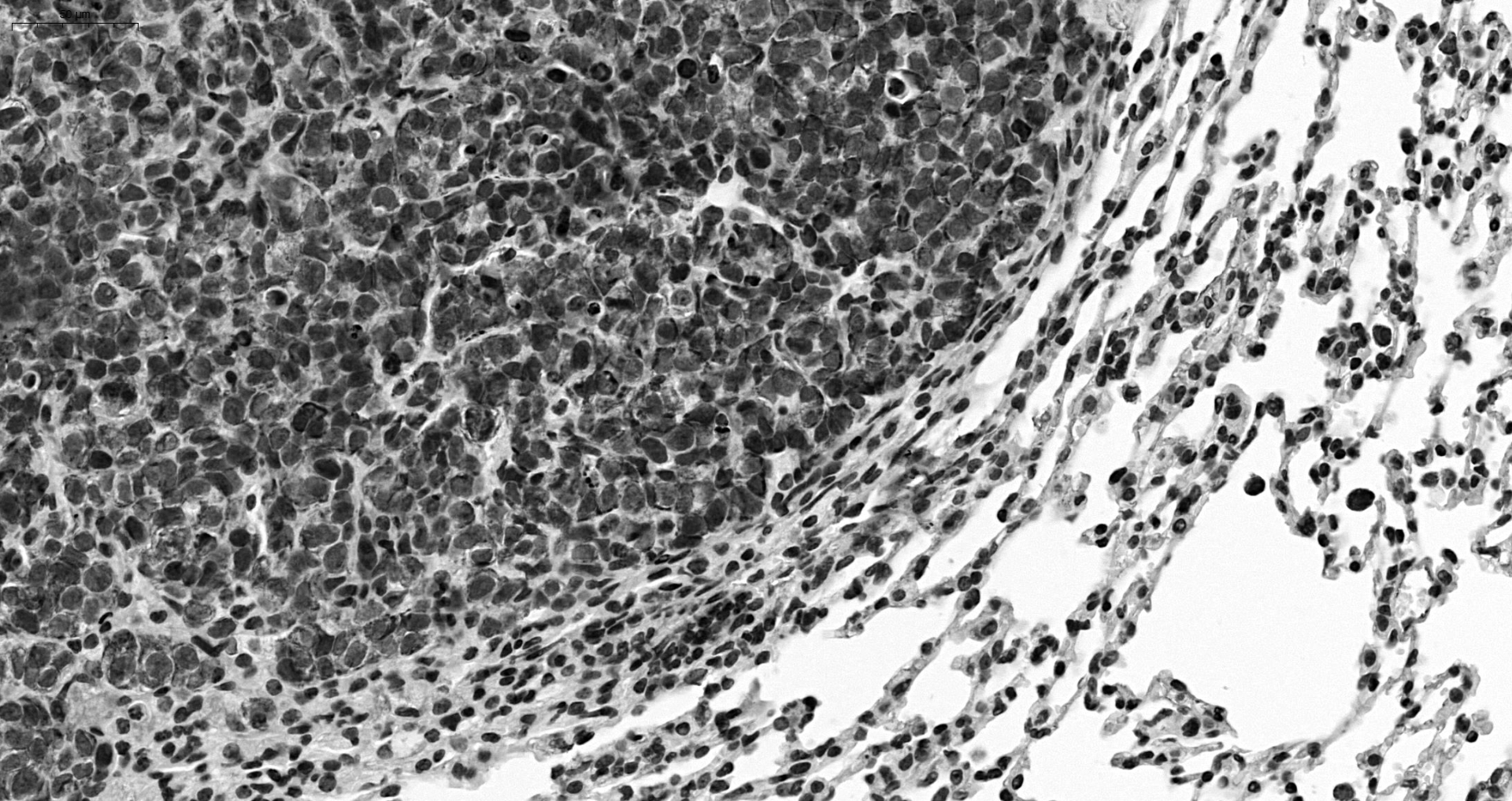

Supplement: Supplementary file 1 — Additional file 1. Raw data. [file 12935_2023_3076_MOESM1_ESM.zip › raw_data/IHC/vimentin/ΓæáSaoS-2+si-NC/2-2 ΓæáSaoS-2+si-NC.tif]

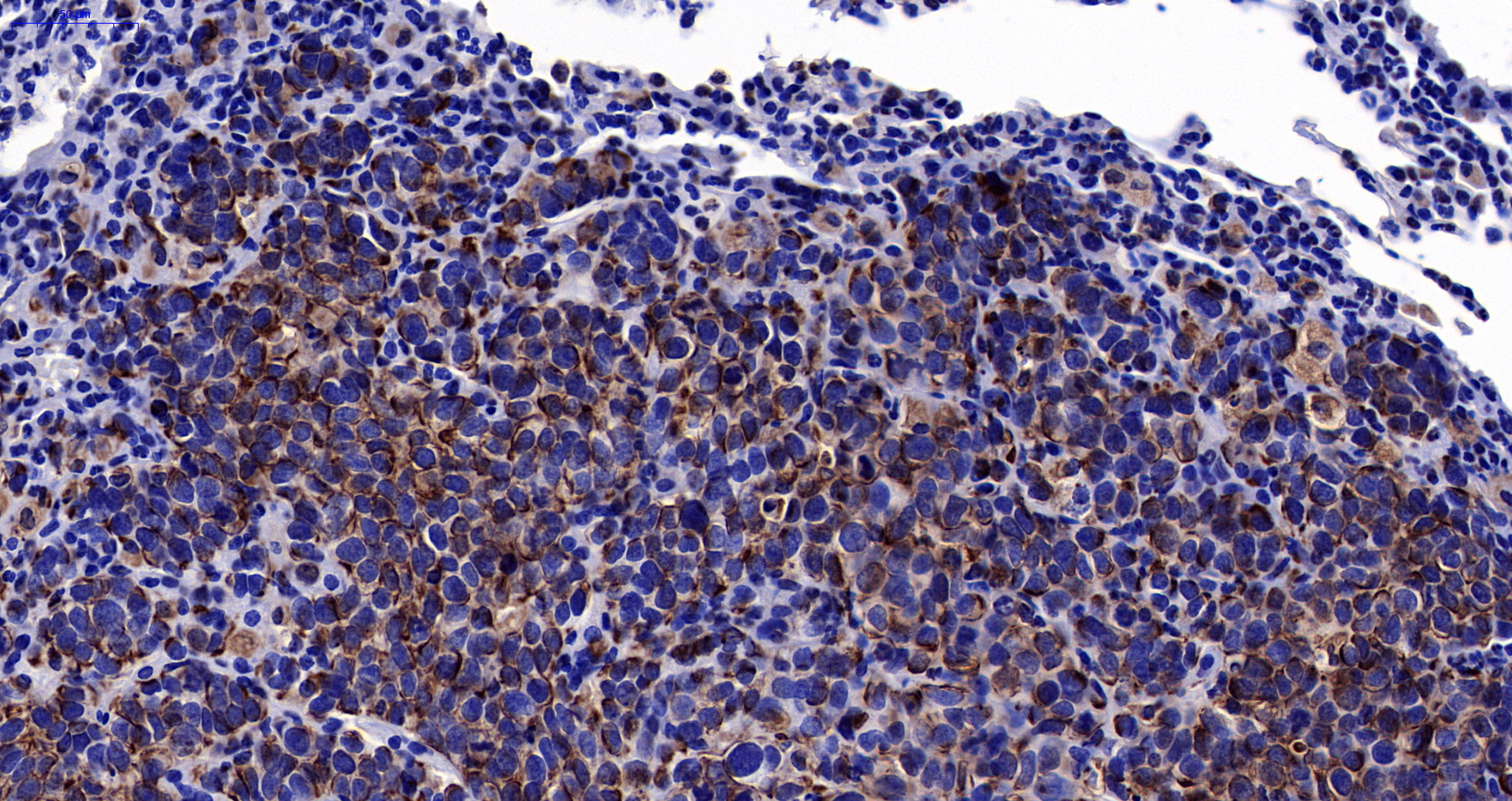

Supplement: Supplementary file 1 — Additional file 1. Raw data. [file 12935_2023_3076_MOESM1_ESM.zip › raw_data/IHC/vimentin/ΓæáSaoS-2+si-NC/3-1 ΓæáSaoS-2+si-NC.jpg]

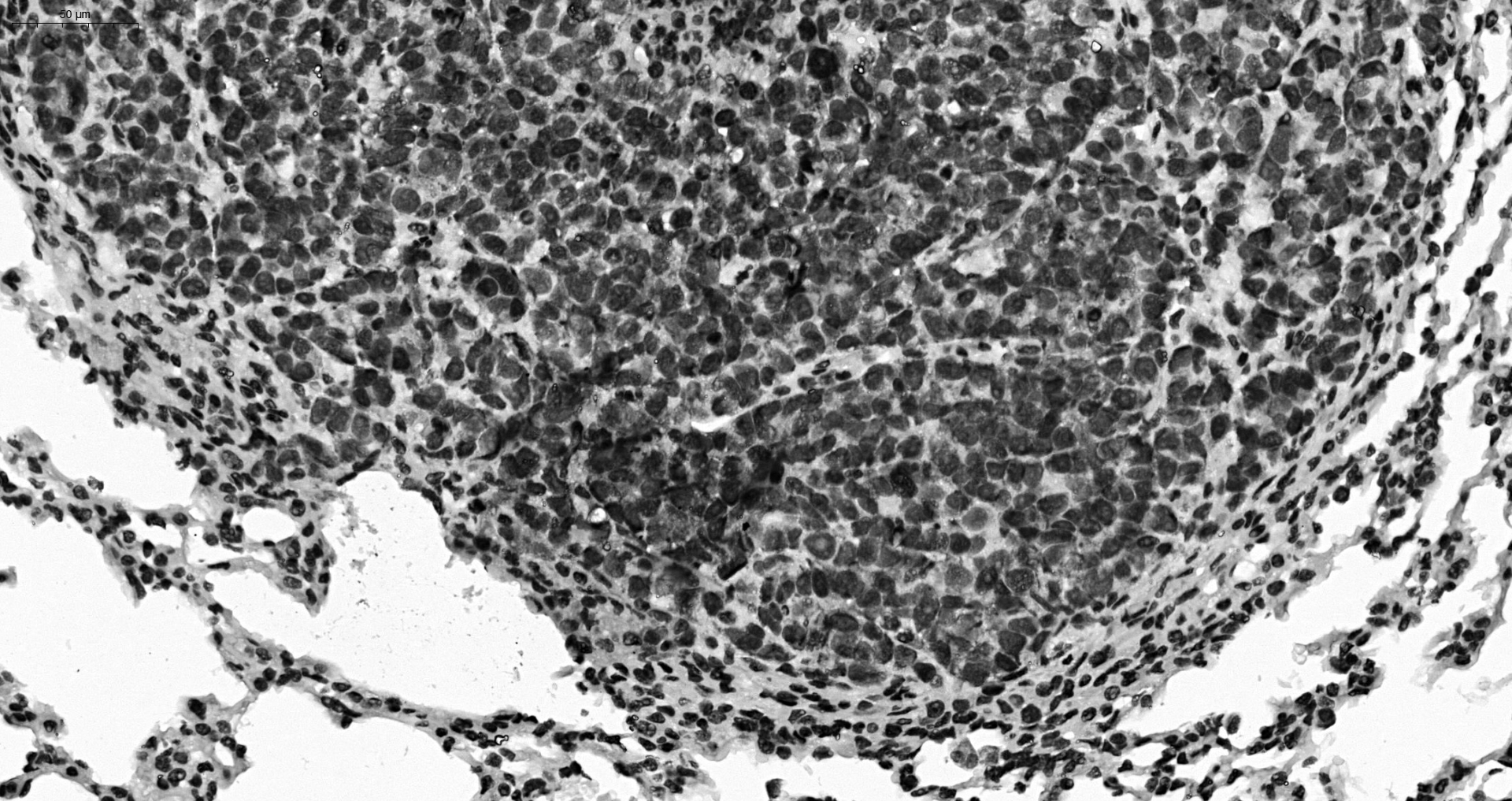

Supplement: Supplementary file 1 — Additional file 1. Raw data. [file 12935_2023_3076_MOESM1_ESM.zip › raw_data/IHC/vimentin/ΓæáSaoS-2+si-NC/1-3 ΓæáSaoS-2+si-NC.tif]

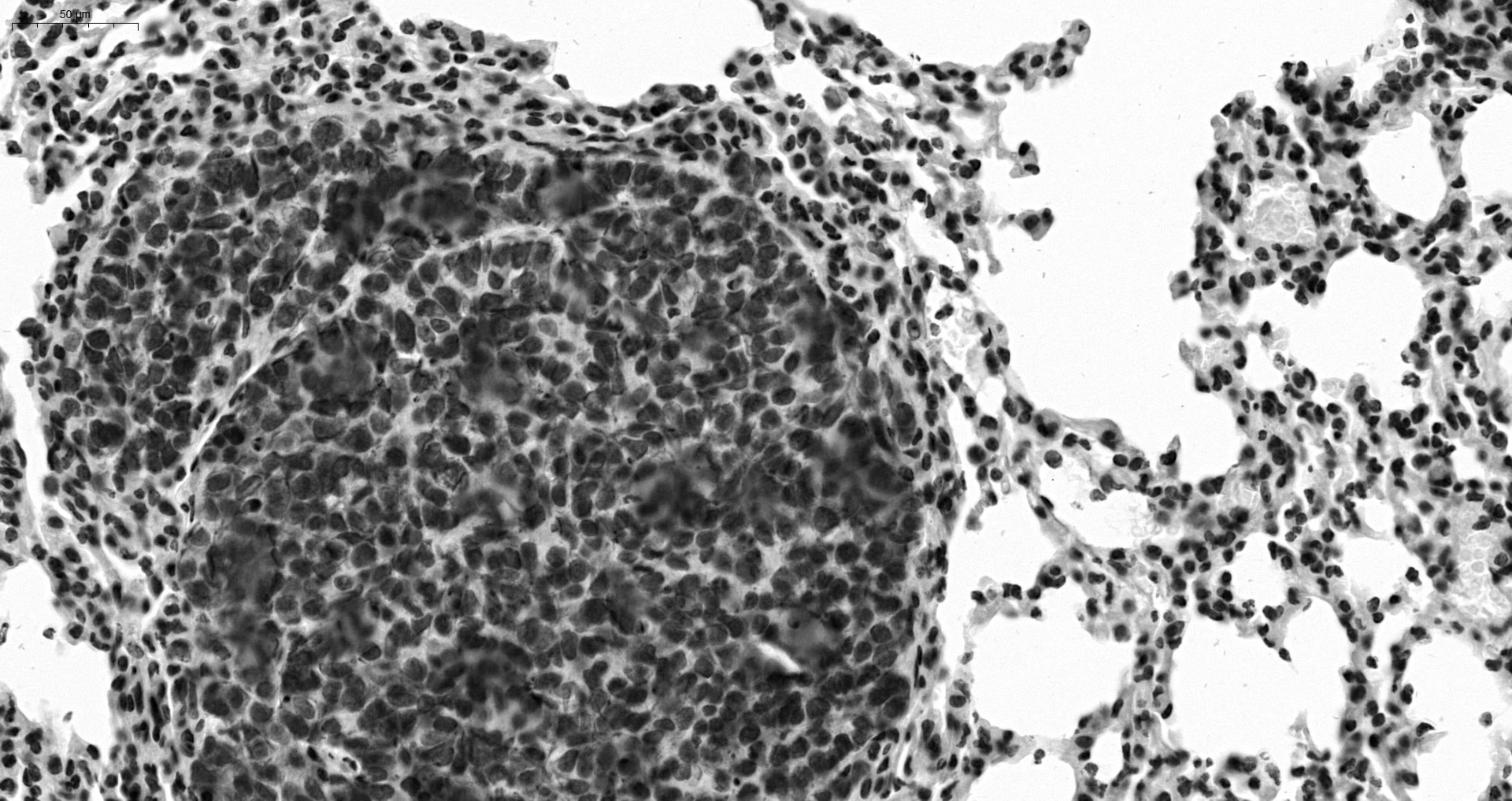

Supplement: Supplementary file 1 — Additional file 1. Raw data. [file 12935_2023_3076_MOESM1_ESM.zip › raw_data/IHC/vimentin/ΓæáSaoS-2+si-NC/2-3 ΓæáSaoS-2+si-NC.tif]

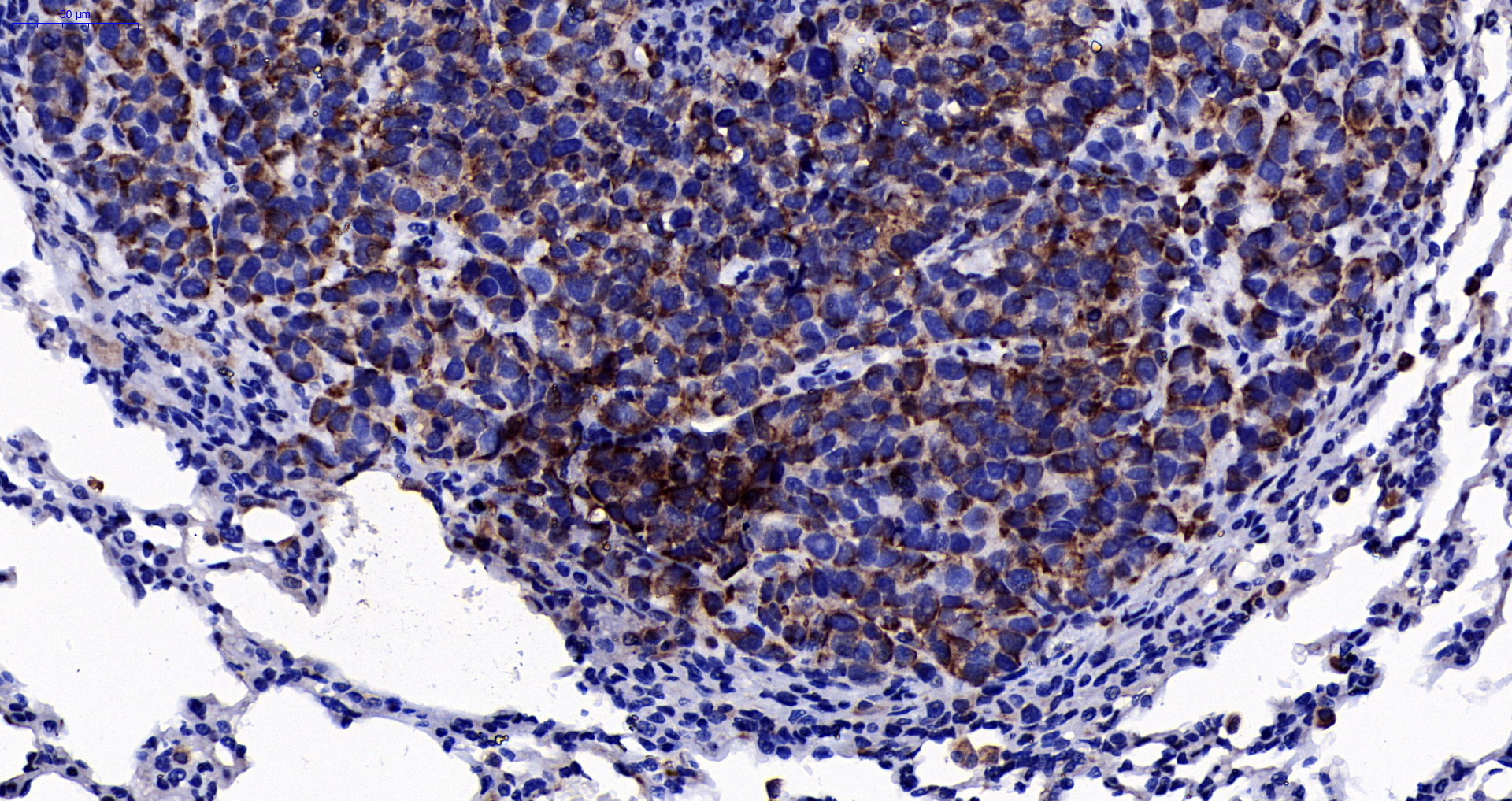

Supplement: Supplementary file 1 — Additional file 1. Raw data. [file 12935_2023_3076_MOESM1_ESM.zip › raw_data/IHC/vimentin/ΓæáSaoS-2+si-NC/1-3 ΓæáSaoS-2+si-NC.jpg]

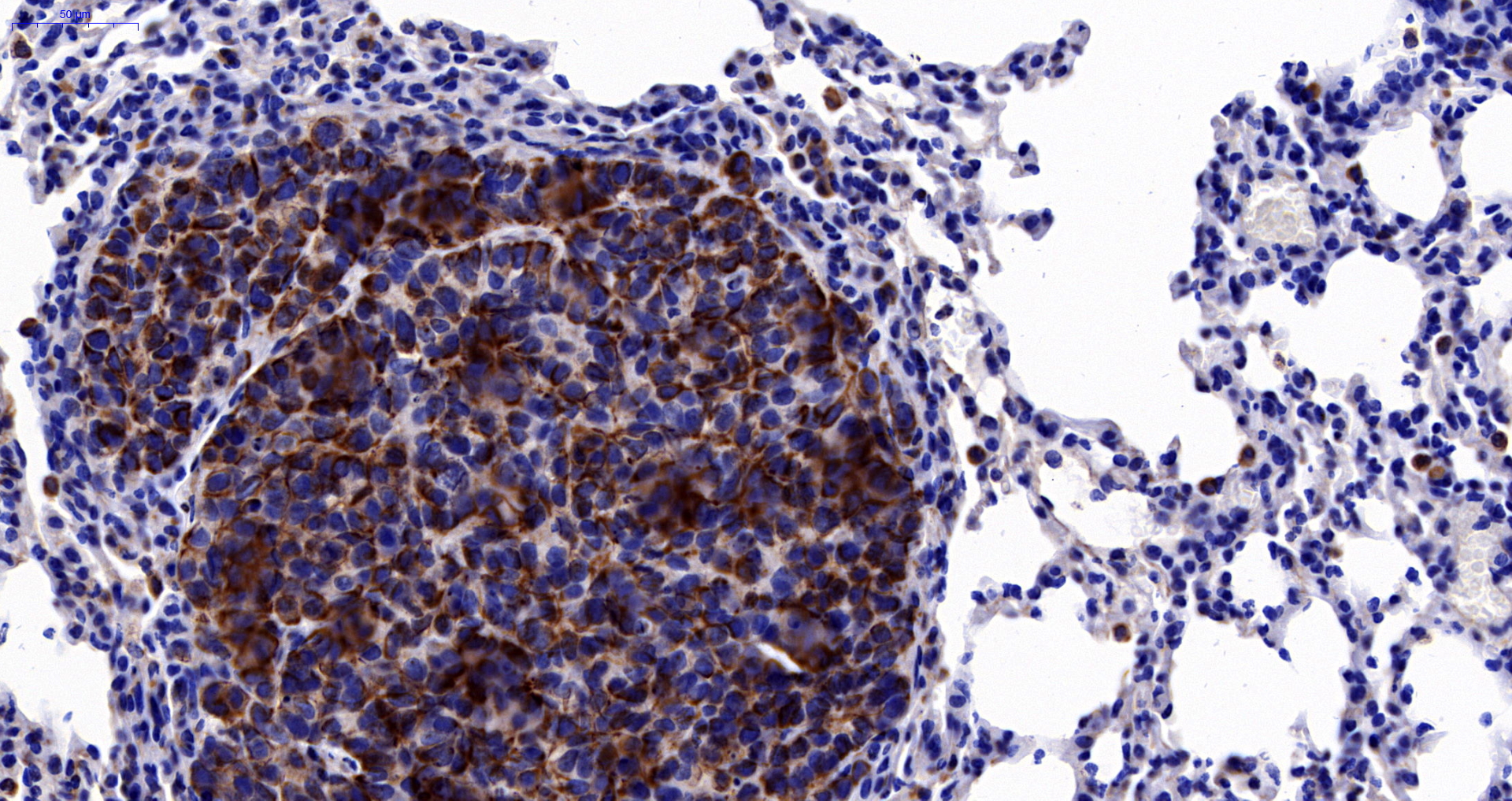

Supplement: Supplementary file 1 — Additional file 1. Raw data. [file 12935_2023_3076_MOESM1_ESM.zip › raw_data/IHC/vimentin/ΓæáSaoS-2+si-NC/2-3 ΓæáSaoS-2+si-NC.jpg]

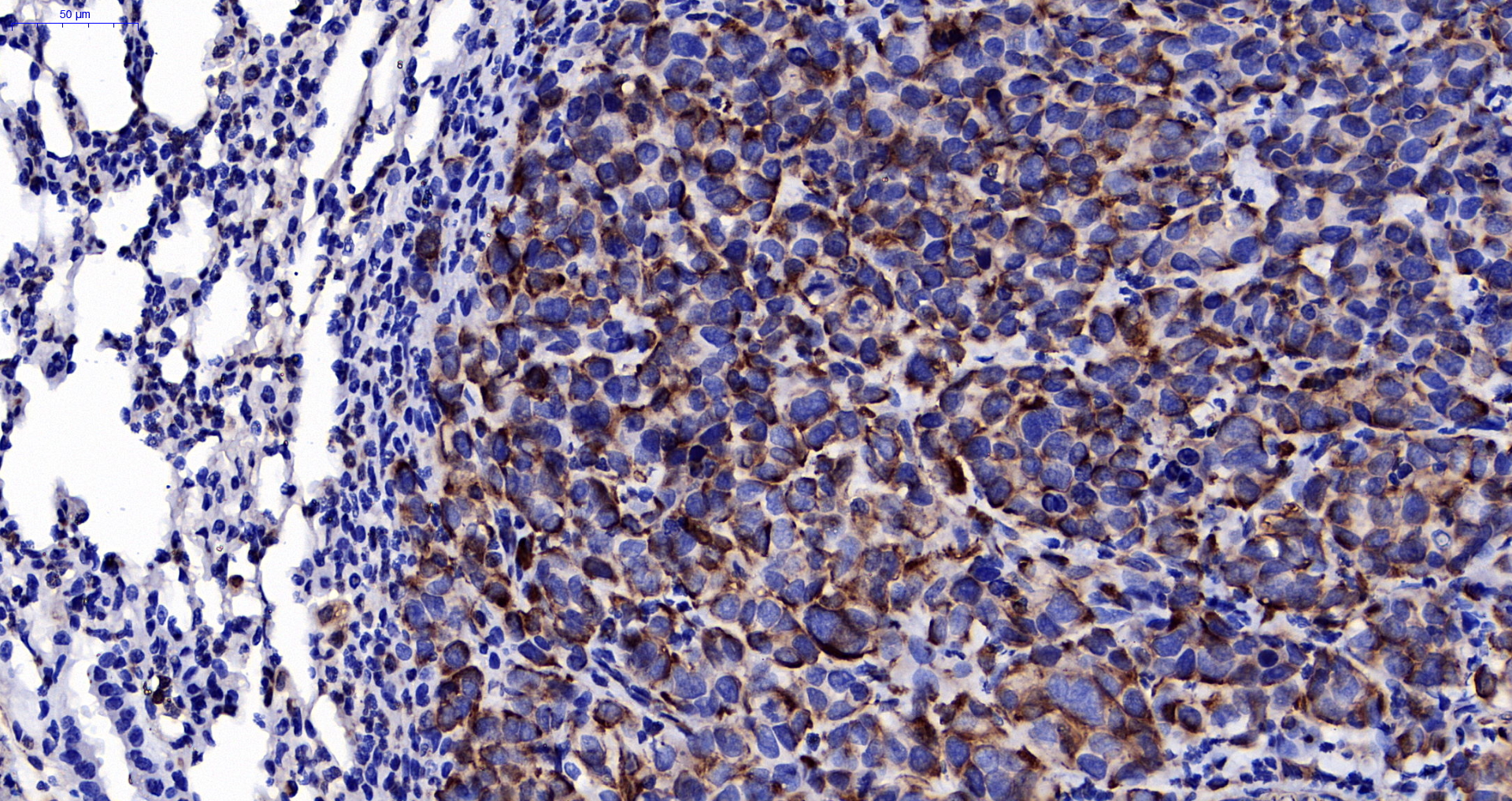

Supplement: Supplementary file 1 — Additional file 1. Raw data. [file 12935_2023_3076_MOESM1_ESM.zip › raw_data/IHC/vimentin/ΓæáSaoS-2+si-NC/3-3 ΓæáSaoS-2+si-NC.jpg]

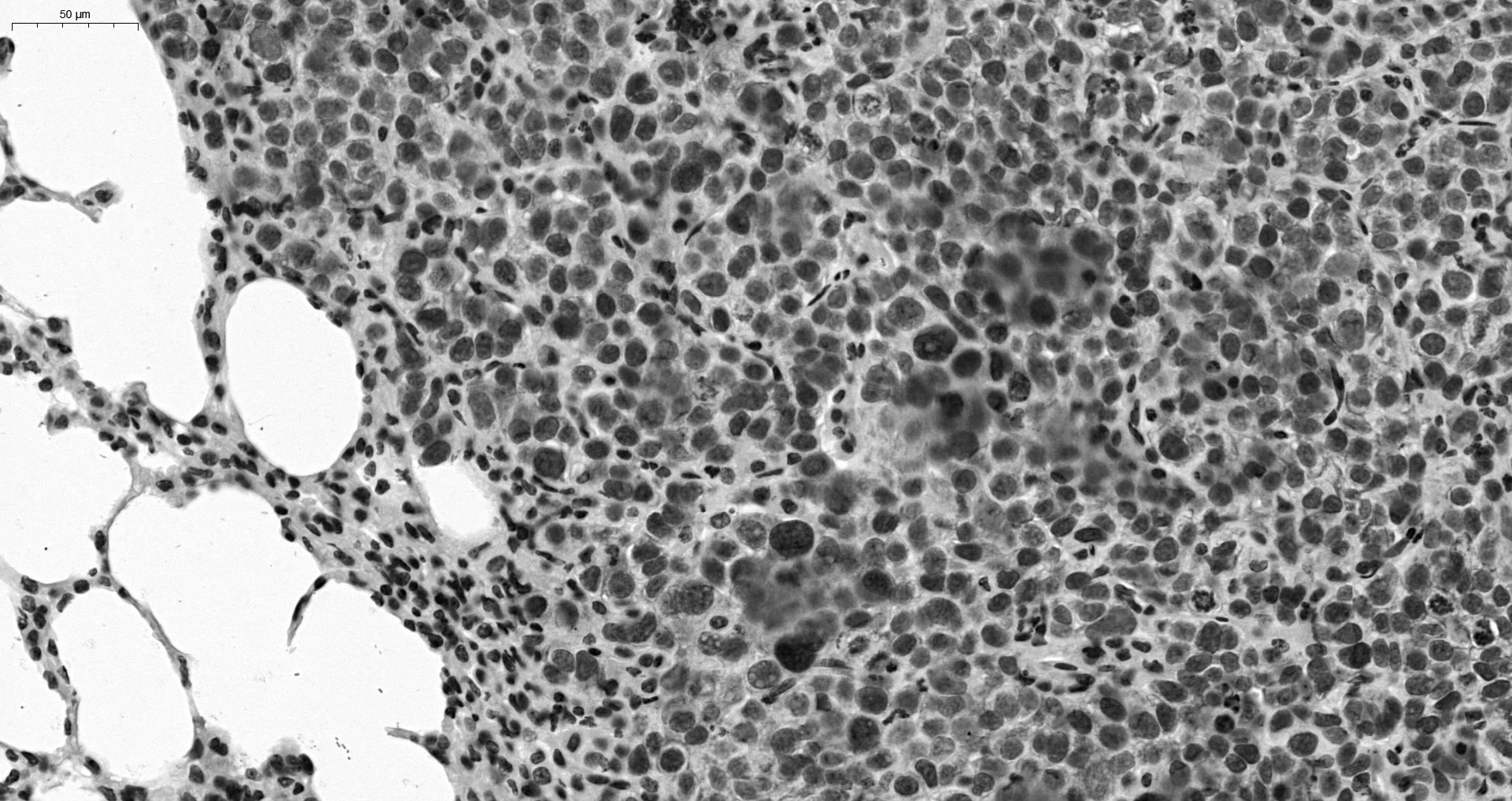

Supplement: Supplementary file 1 — Additional file 1. Raw data. [file 12935_2023_3076_MOESM1_ESM.zip › raw_data/IHC/vimentin/ΓæáSaoS-2+si-NC/1-1 ΓæáSaoS-2+si-NC.tif]

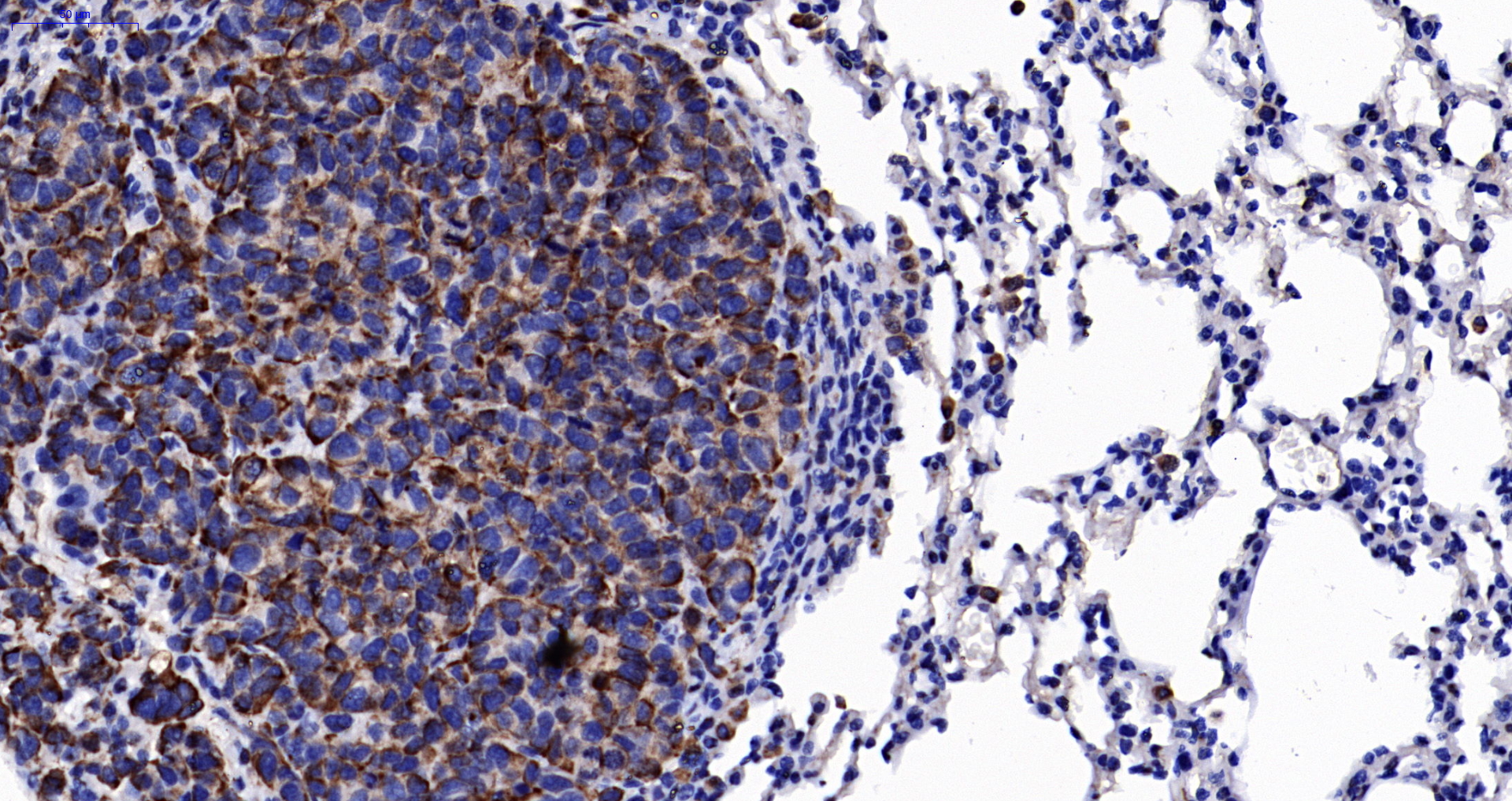

Supplement: Supplementary file 1 — Additional file 1. Raw data. [file 12935_2023_3076_MOESM1_ESM.zip › raw_data/IHC/vimentin/ΓæáSaoS-2+si-NC/1-2 ΓæáSaoS-2+si-NC.jpg]

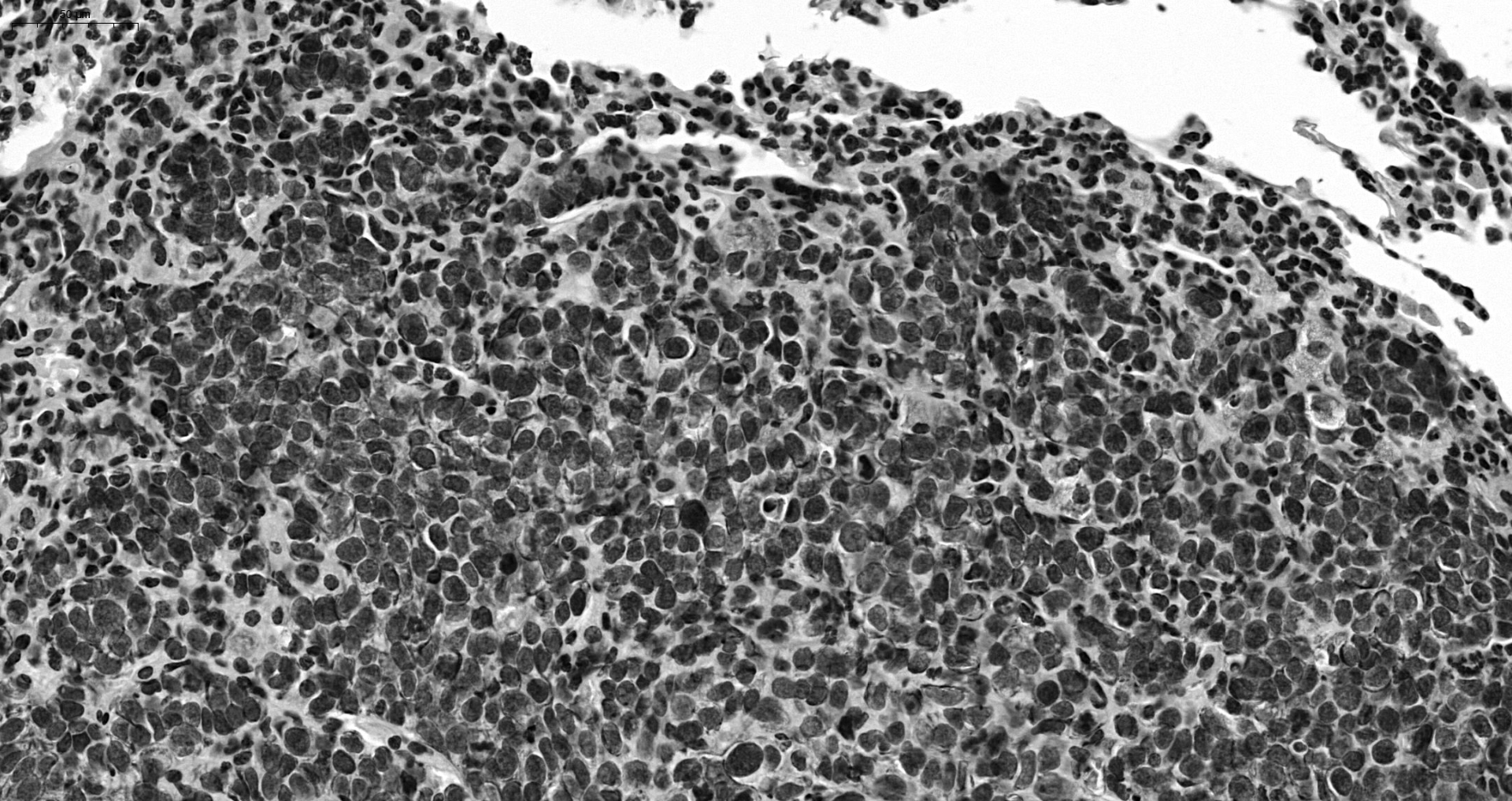

Supplement: Supplementary file 1 — Additional file 1. Raw data. [file 12935_2023_3076_MOESM1_ESM.zip › raw_data/IHC/vimentin/ΓæáSaoS-2+si-NC/3-1 ΓæáSaoS-2+si-NC.tif]

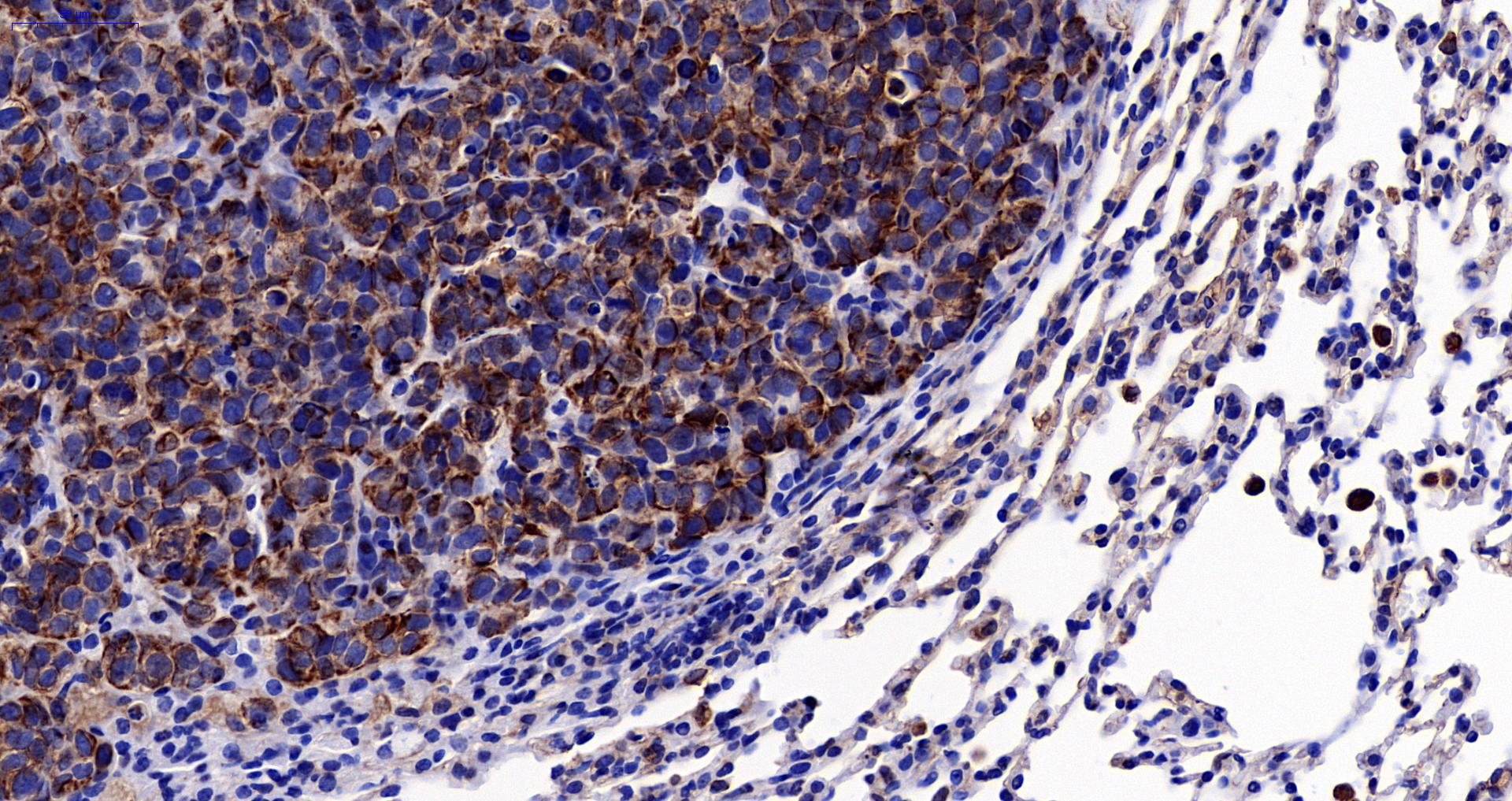

Supplement: Supplementary file 1 — Additional file 1. Raw data. [file 12935_2023_3076_MOESM1_ESM.zip › raw_data/IHC/vimentin/ΓæáSaoS-2+si-NC/2-2 ΓæáSaoS-2+si-NC.jpg]

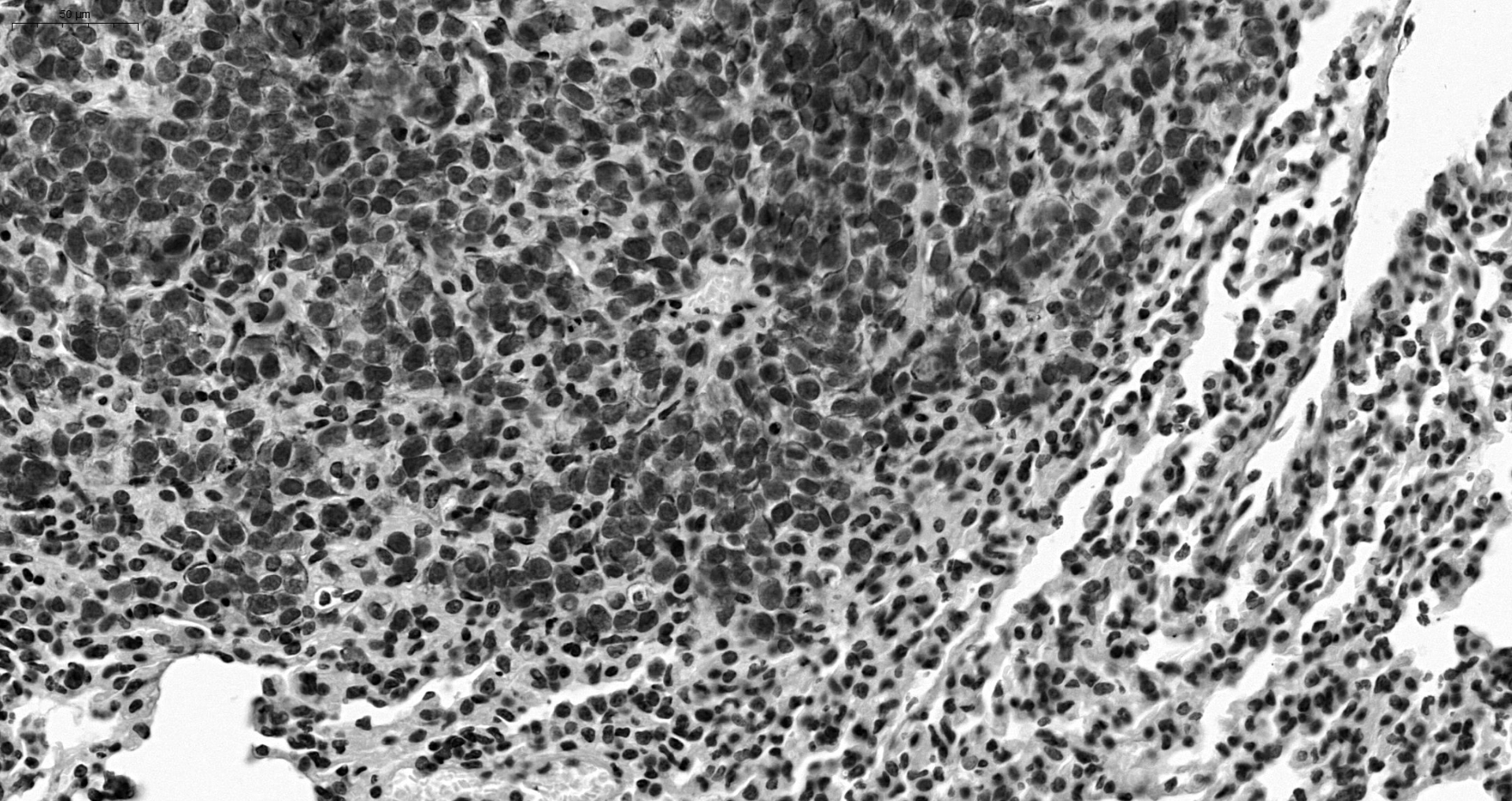

Supplement: Supplementary file 1 — Additional file 1. Raw data. [file 12935_2023_3076_MOESM1_ESM.zip › raw_data/IHC/vimentin/ΓæáSaoS-2+si-NC/2-1 ΓæáSaoS-2+si-NC.tif]

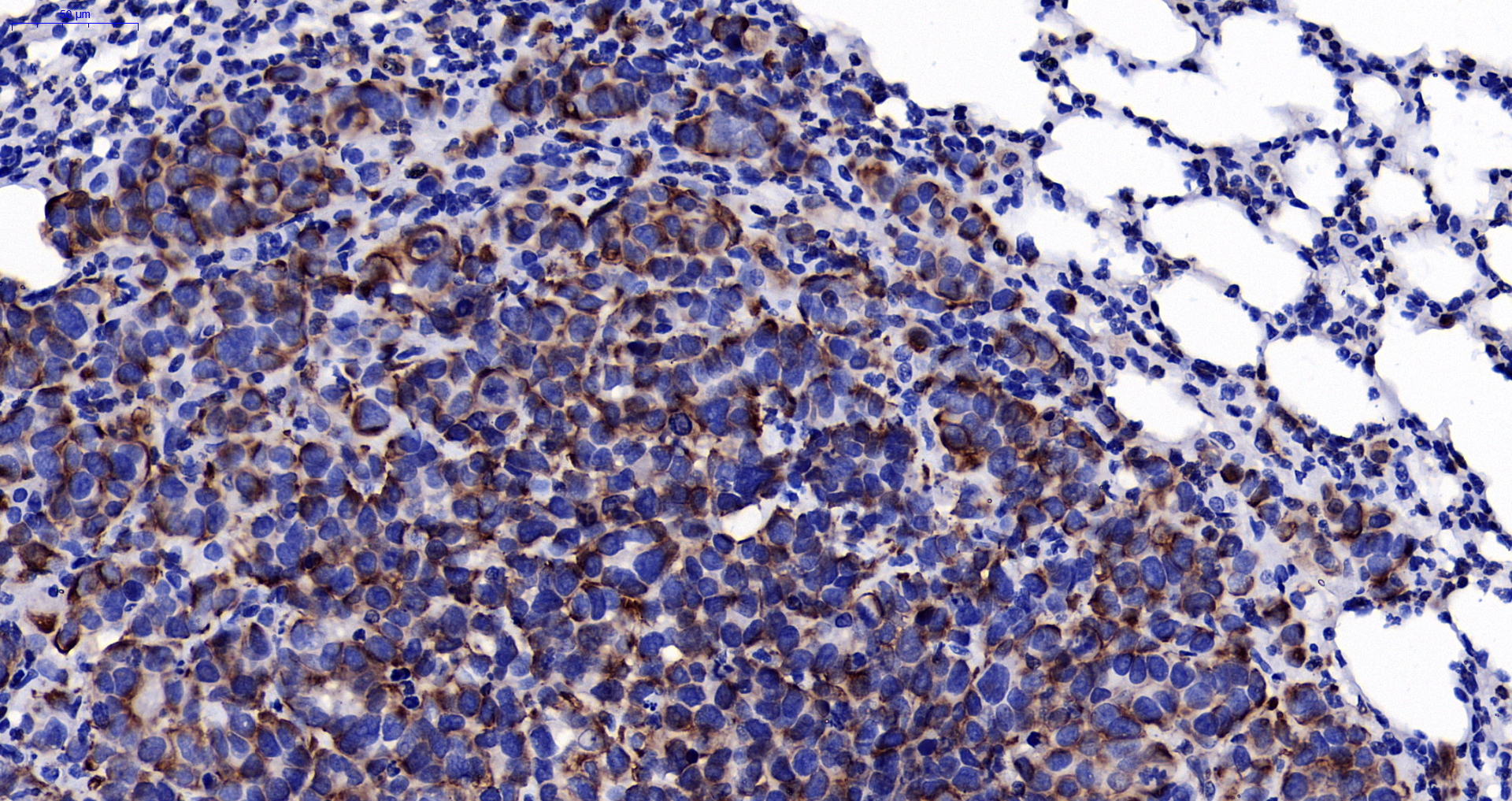

Supplement: Supplementary file 1 — Additional file 1. Raw data. [file 12935_2023_3076_MOESM1_ESM.zip › raw_data/IHC/vimentin/ΓæáSaoS-2+si-NC/3-2 ΓæáSaoS-2+si-NC.jpg]

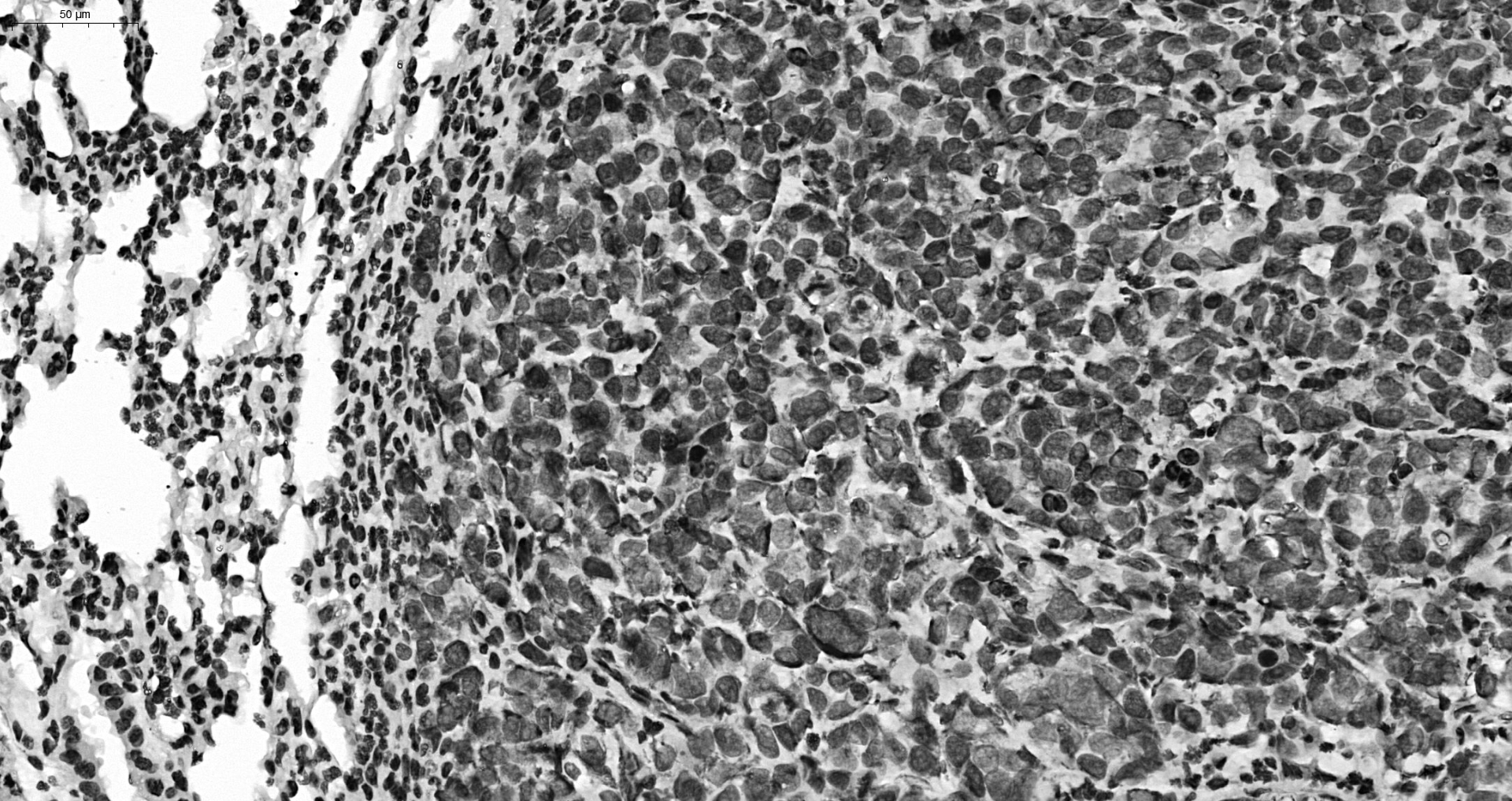

Supplement: Supplementary file 1 — Additional file 1. Raw data. [file 12935_2023_3076_MOESM1_ESM.zip › raw_data/IHC/vimentin/ΓæáSaoS-2+si-NC/3-3 ΓæáSaoS-2+si-NC-1.tif]

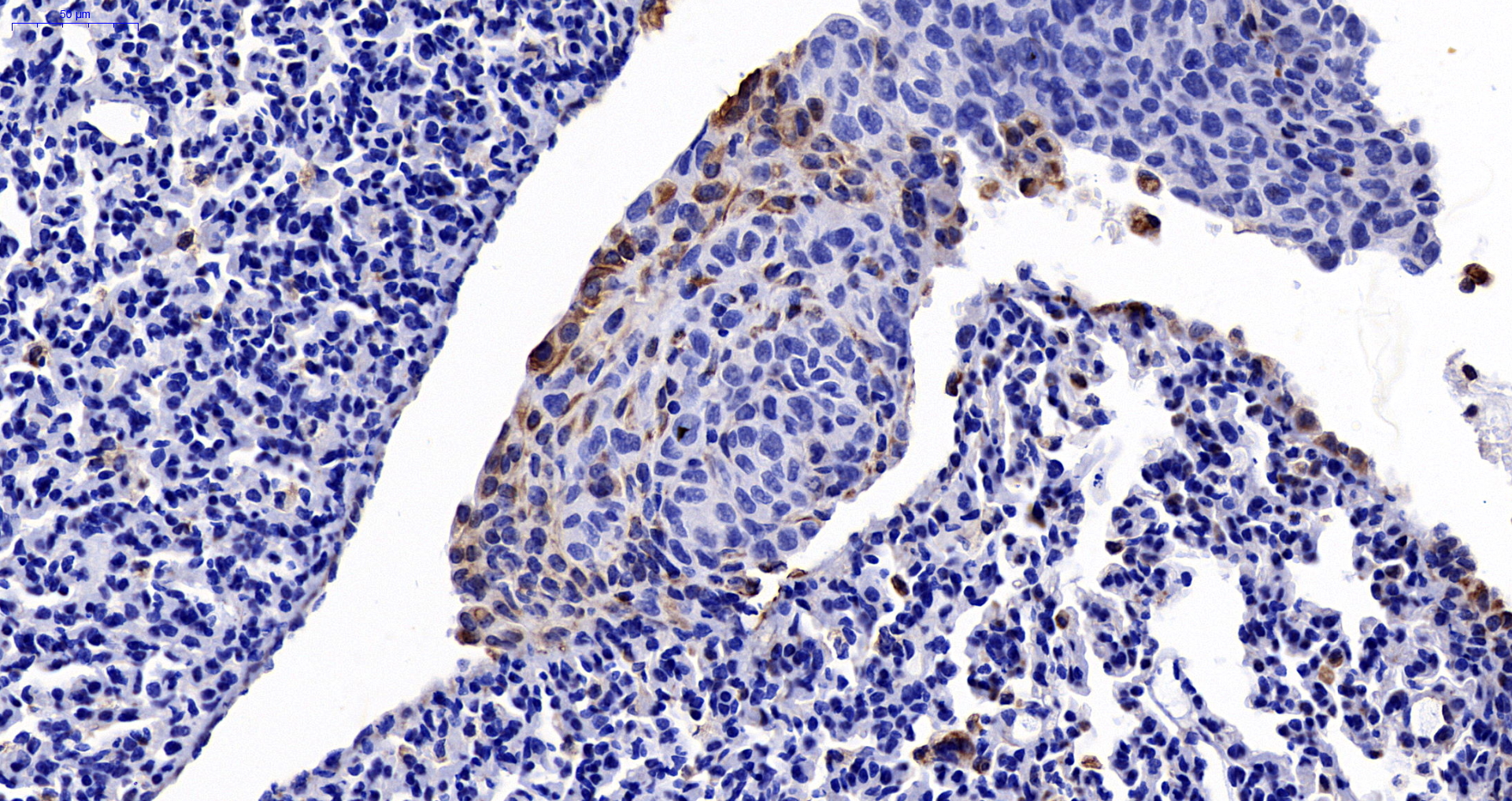

Supplement: Supplementary file 1 — Additional file 1. Raw data. [file 12935_2023_3076_MOESM1_ESM.zip › raw_data/IHC/vimentin/ΓæíSaoS-2+si-DIO3OS/1-2 ΓæíSaoS-2+si-DIO3OS.jpg]

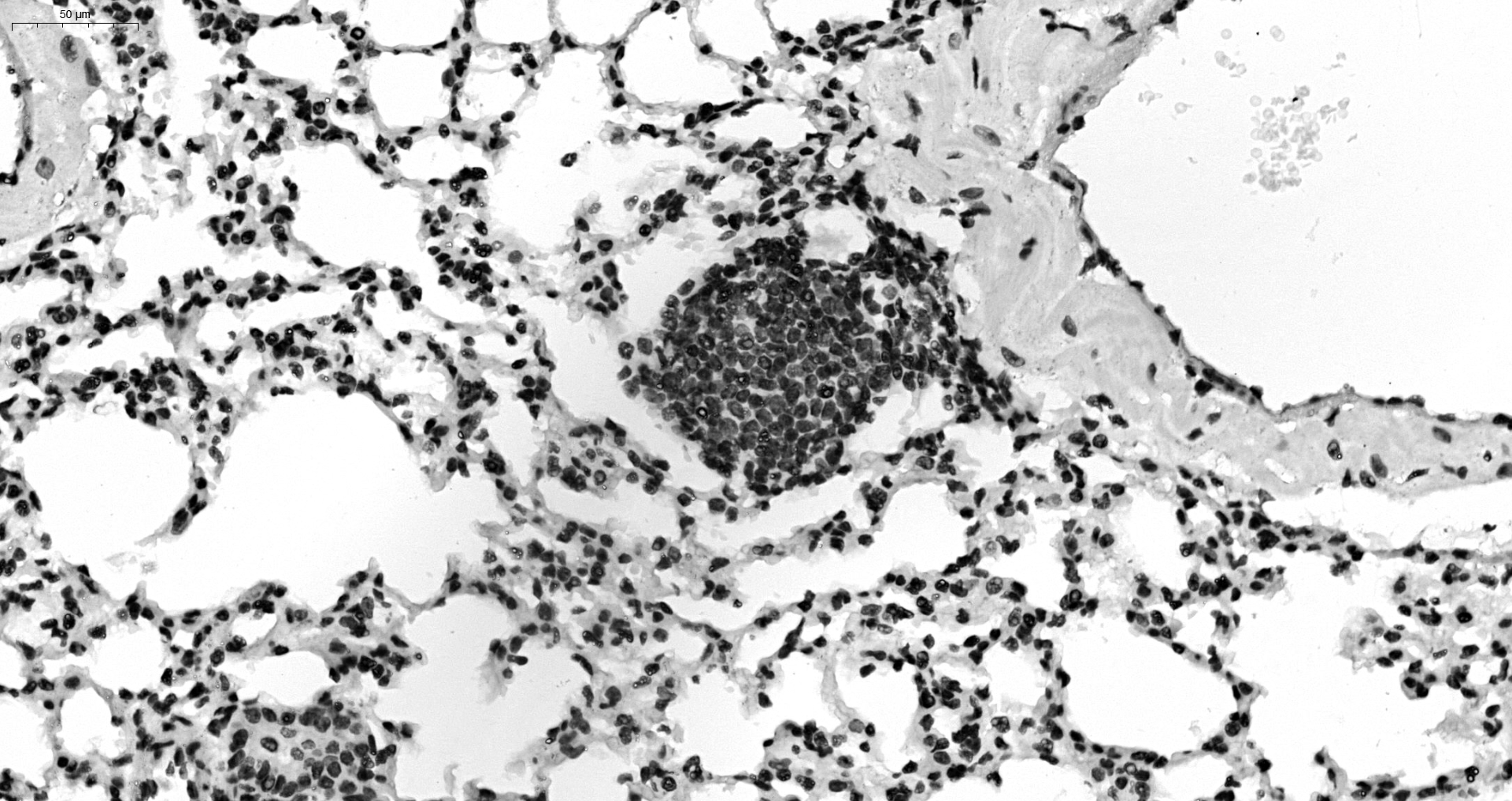

Supplement: Supplementary file 1 — Additional file 1. Raw data. [file 12935_2023_3076_MOESM1_ESM.zip › raw_data/IHC/vimentin/ΓæíSaoS-2+si-DIO3OS/2-2 ΓæíSaoS-2+si-DIO3OS.tif]

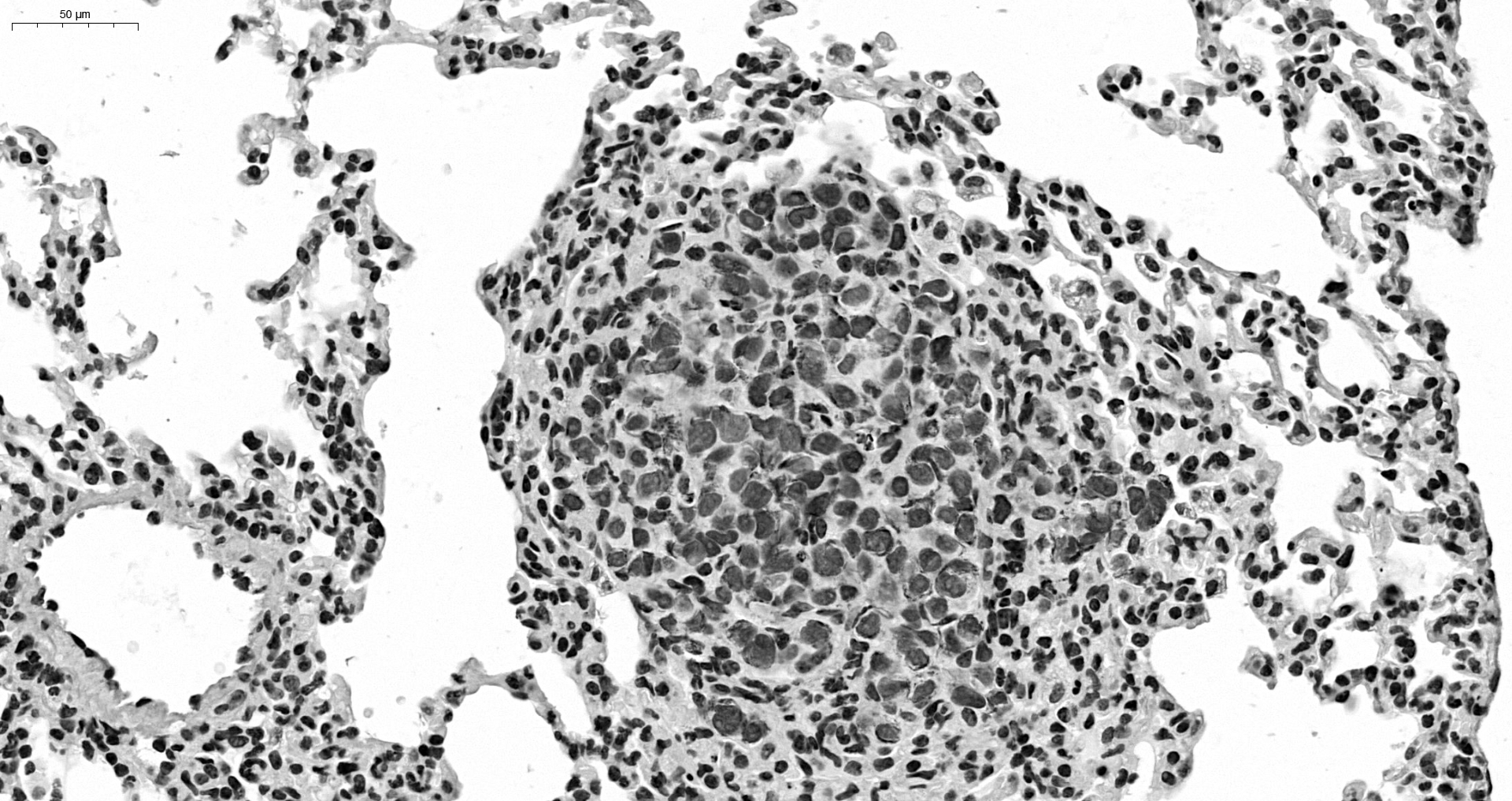

Supplement: Supplementary file 1 — Additional file 1. Raw data. [file 12935_2023_3076_MOESM1_ESM.zip › raw_data/IHC/vimentin/ΓæíSaoS-2+si-DIO3OS/3-1 ΓæíSaoS-2+si-DIO3OS.tif]

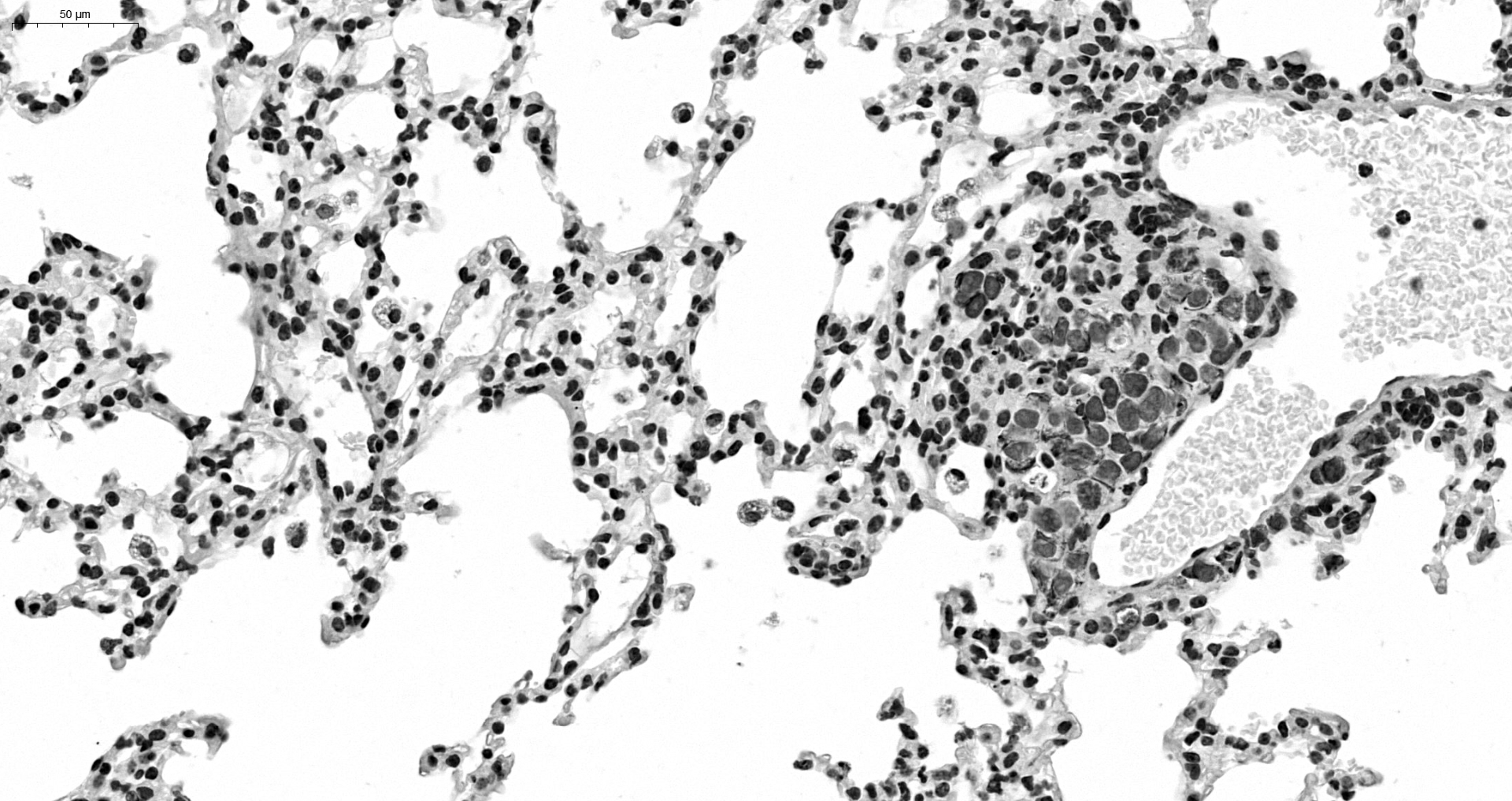

Supplement: Supplementary file 1 — Additional file 1. Raw data. [file 12935_2023_3076_MOESM1_ESM.zip › raw_data/IHC/vimentin/ΓæíSaoS-2+si-DIO3OS/3-3 ΓæíSaoS-2+si-DIO3OS.tif]

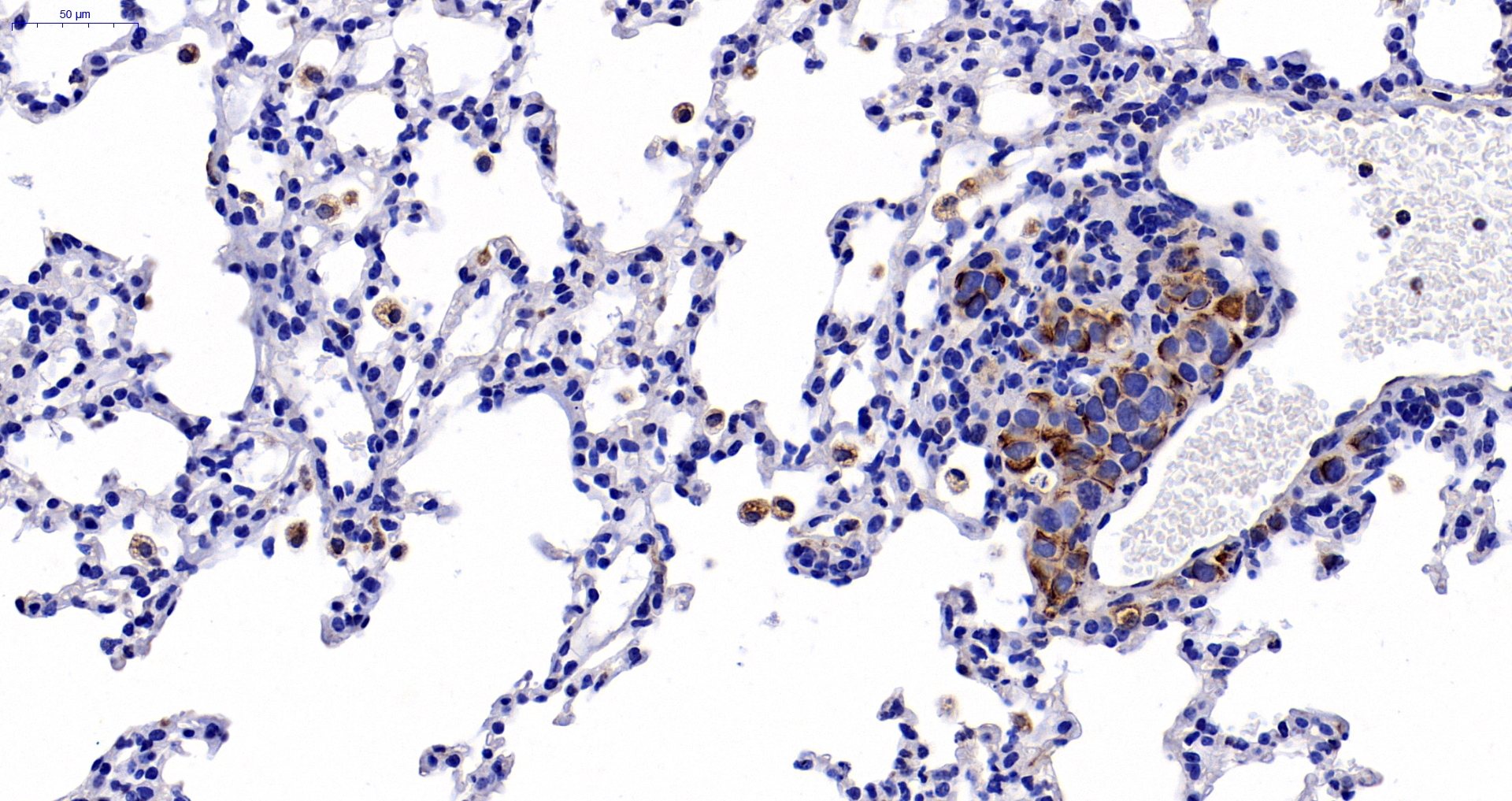

Supplement: Supplementary file 1 — Additional file 1. Raw data. [file 12935_2023_3076_MOESM1_ESM.zip › raw_data/IHC/vimentin/ΓæíSaoS-2+si-DIO3OS/3-3 ΓæíSaoS-2+si-DIO3OS.jpg]

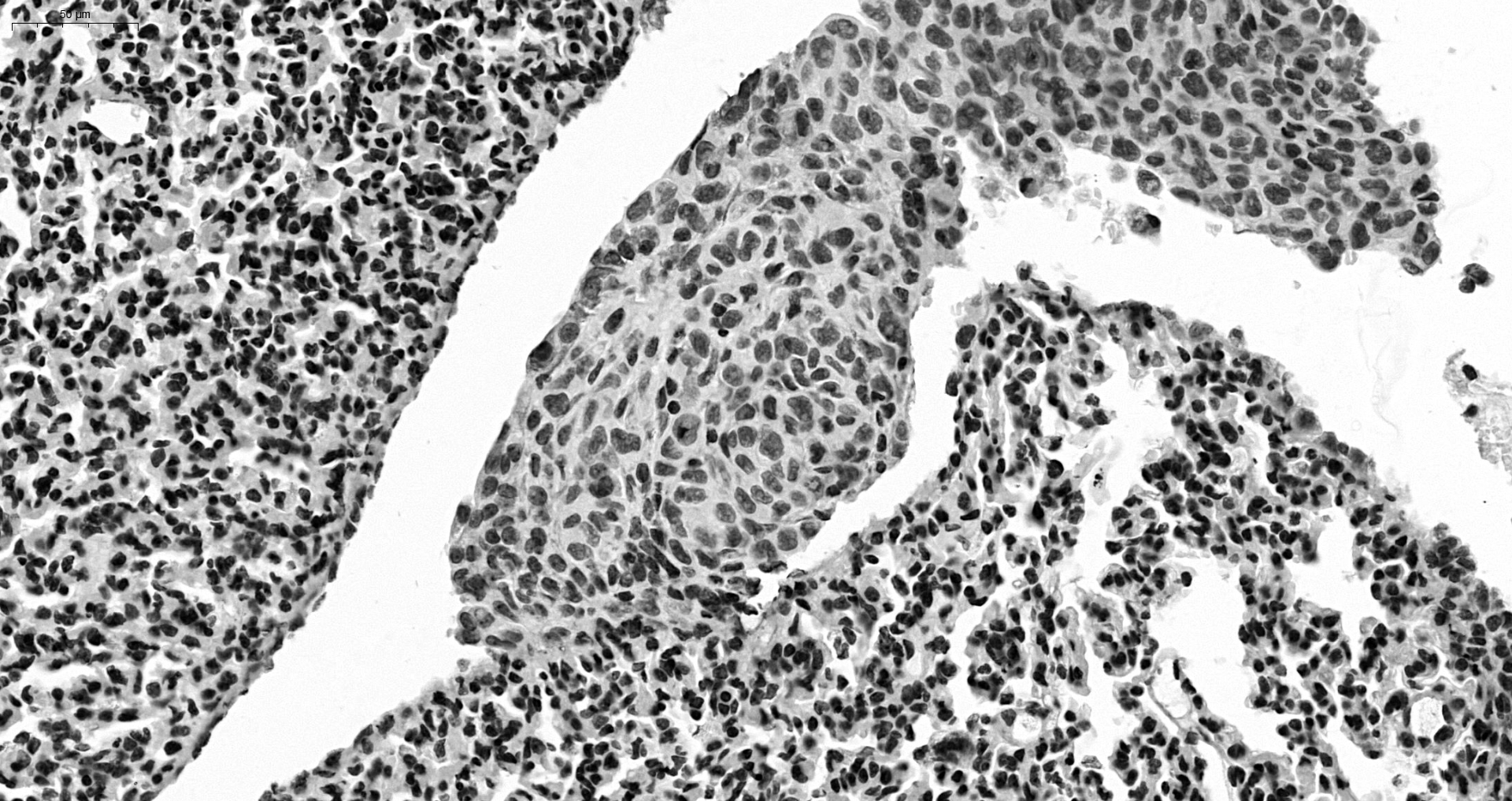

Supplement: Supplementary file 1 — Additional file 1. Raw data. [file 12935_2023_3076_MOESM1_ESM.zip › raw_data/IHC/vimentin/ΓæíSaoS-2+si-DIO3OS/1-2 ΓæíSaoS-2+si-DIO3OS.tif]

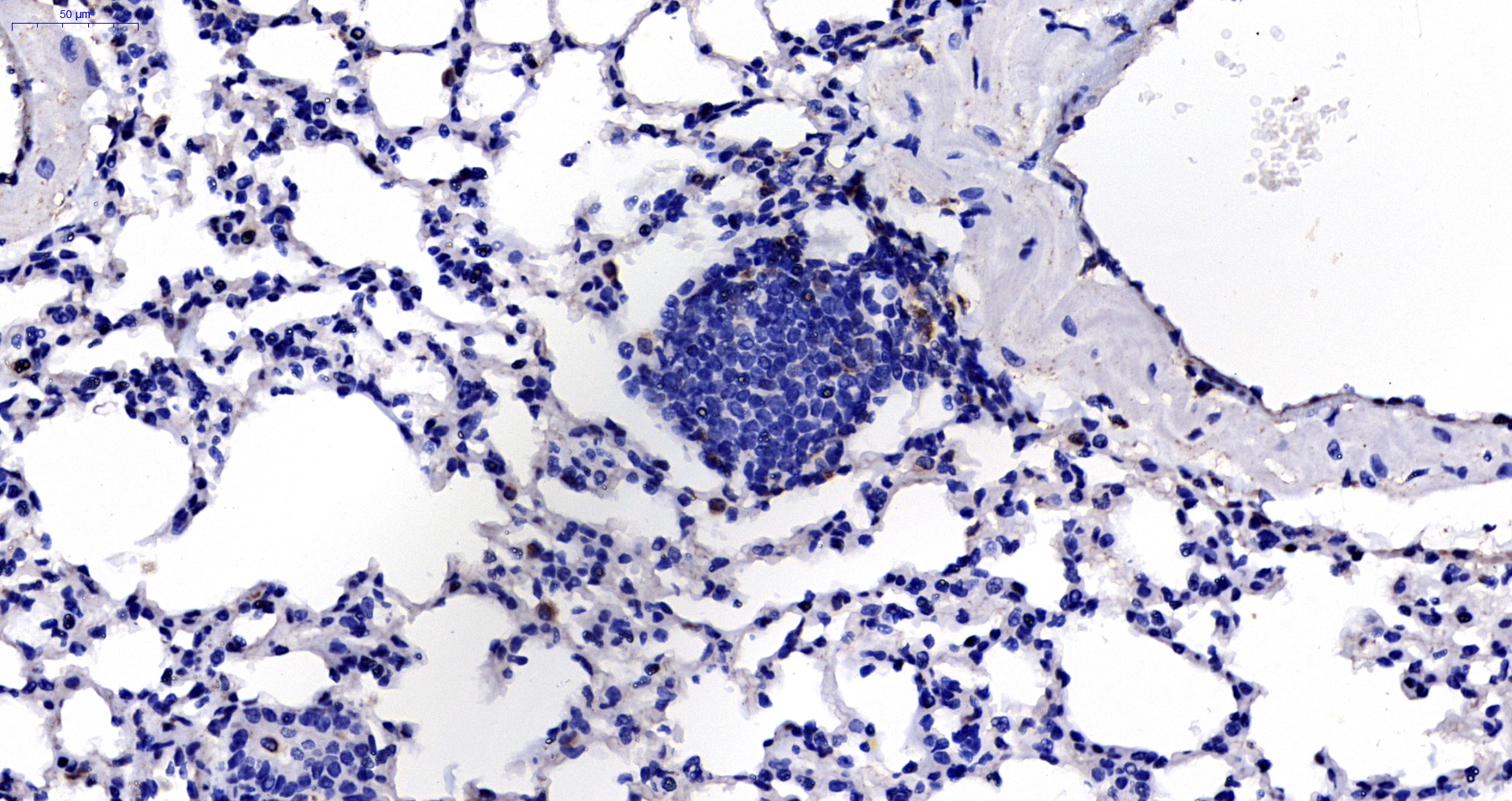

Supplement: Supplementary file 1 — Additional file 1. Raw data. [file 12935_2023_3076_MOESM1_ESM.zip › raw_data/IHC/vimentin/ΓæíSaoS-2+si-DIO3OS/2-2 ΓæíSaoS-2+si-DIO3OS.jpg]

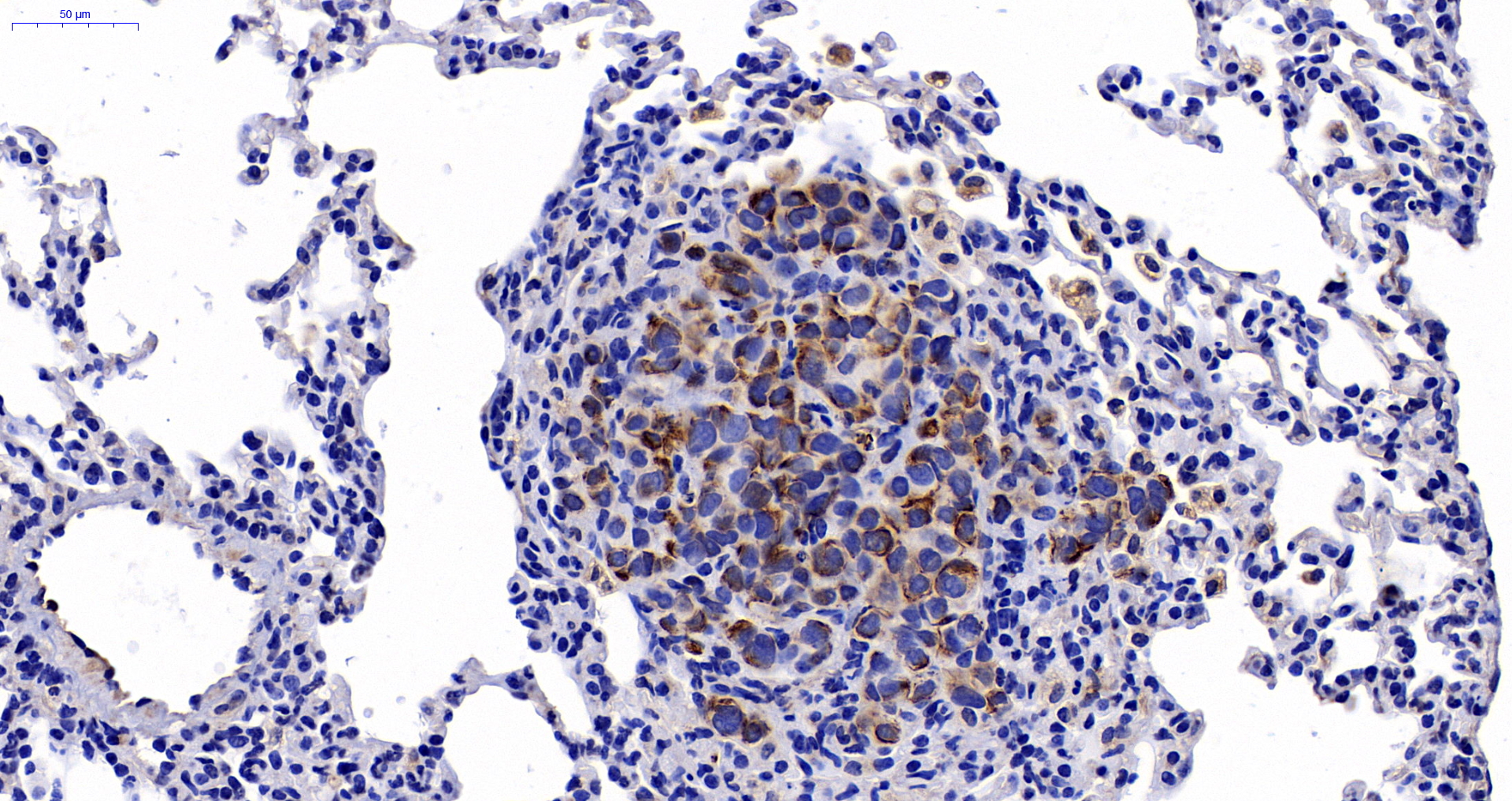

Supplement: Supplementary file 1 — Additional file 1. Raw data. [file 12935_2023_3076_MOESM1_ESM.zip › raw_data/IHC/vimentin/ΓæíSaoS-2+si-DIO3OS/3-1 ΓæíSaoS-2+si-DIO3OS.jpg]

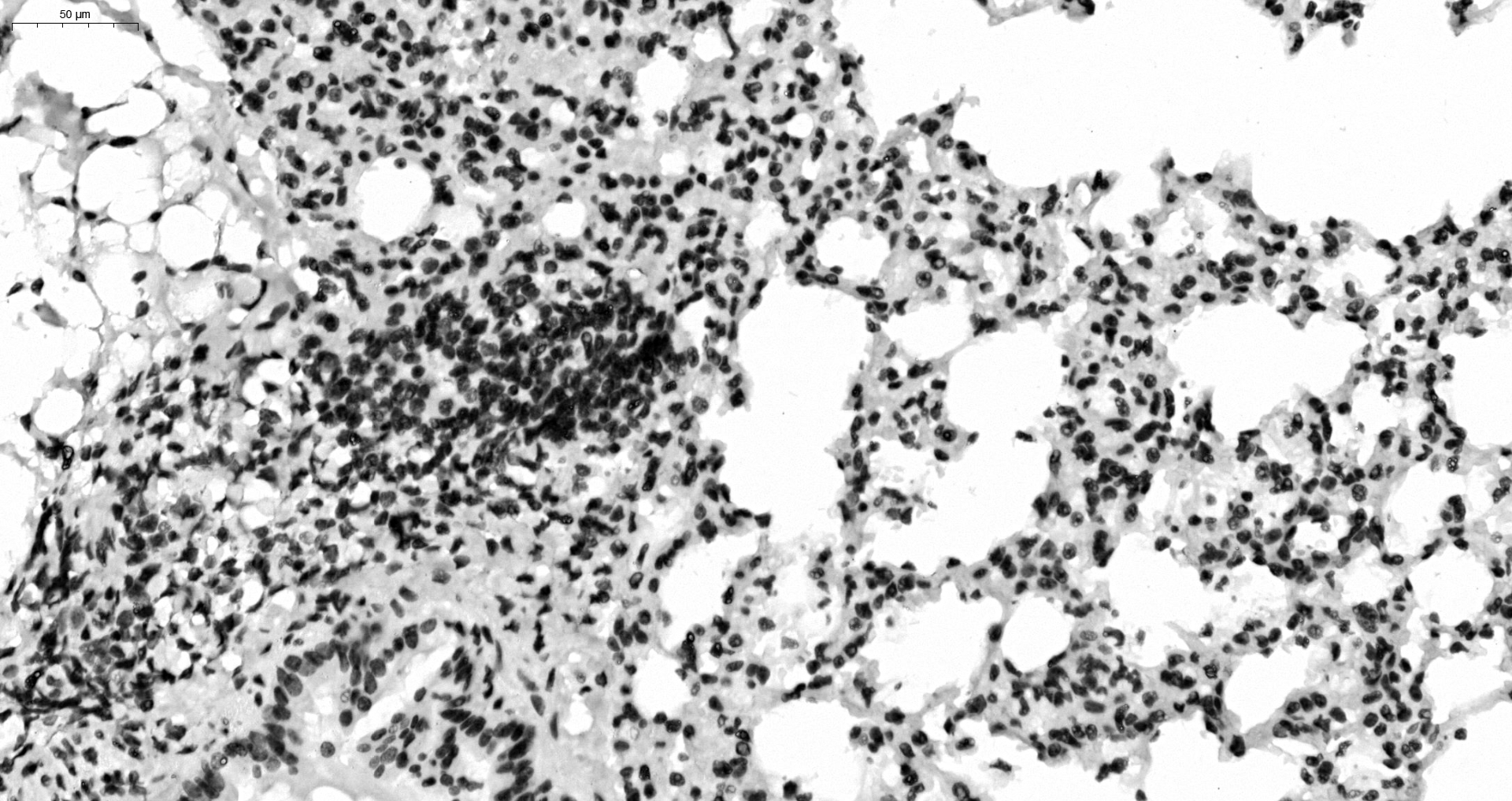

Supplement: Supplementary file 1 — Additional file 1. Raw data. [file 12935_2023_3076_MOESM1_ESM.zip › raw_data/IHC/vimentin/ΓæíSaoS-2+si-DIO3OS/2-3 ΓæíSaoS-2+si-DIO3OS.tif]

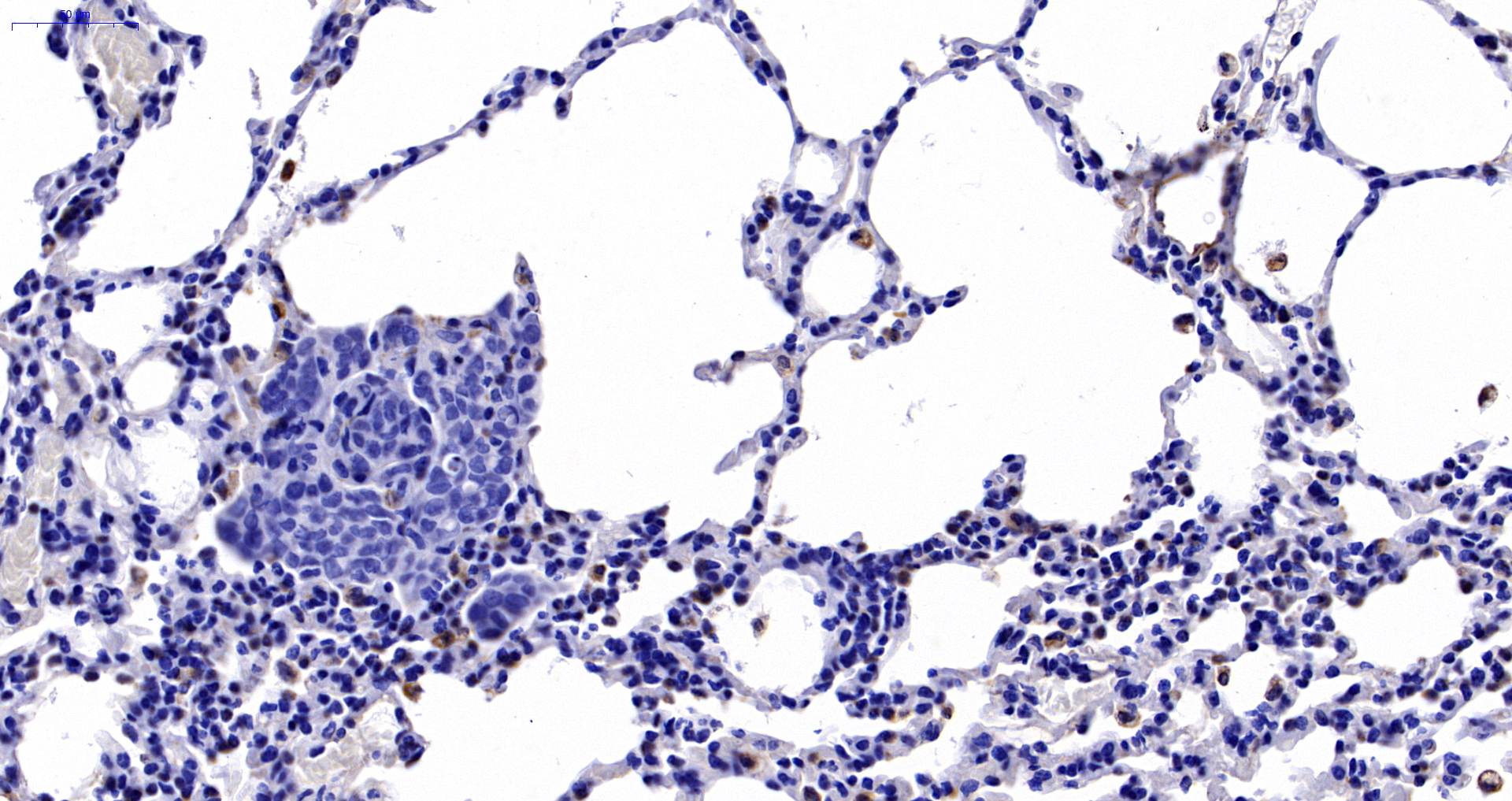

Supplement: Supplementary file 1 — Additional file 1. Raw data. [file 12935_2023_3076_MOESM1_ESM.zip › raw_data/IHC/vimentin/ΓæíSaoS-2+si-DIO3OS/1-3 ΓæíSaoS-2+si-DIO3OS.jpg]

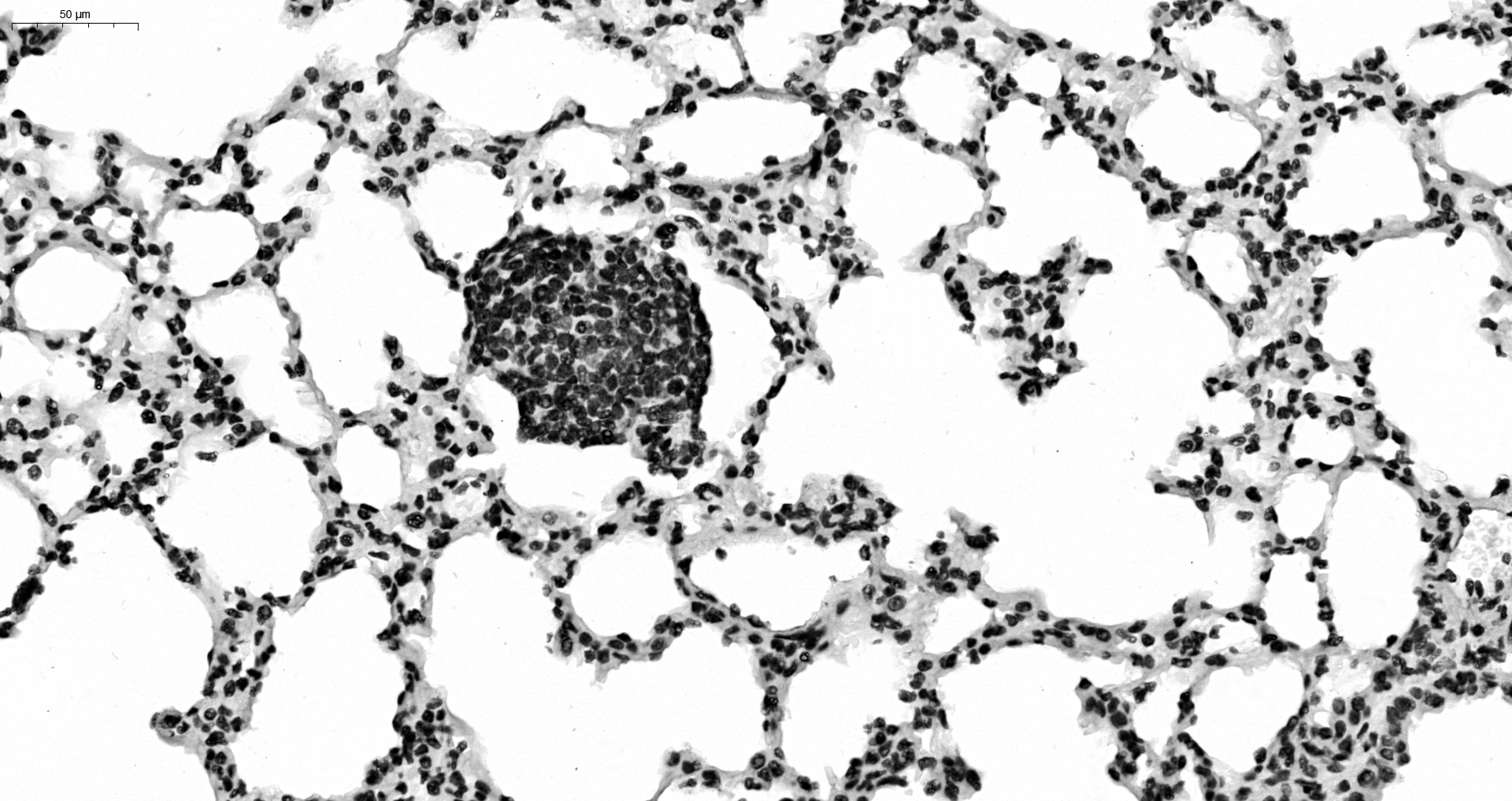

Supplement: Supplementary file 1 — Additional file 1. Raw data. [file 12935_2023_3076_MOESM1_ESM.zip › raw_data/IHC/vimentin/ΓæíSaoS-2+si-DIO3OS/2-1 ΓæíSaoS-2+si-DIO3OS.tif]

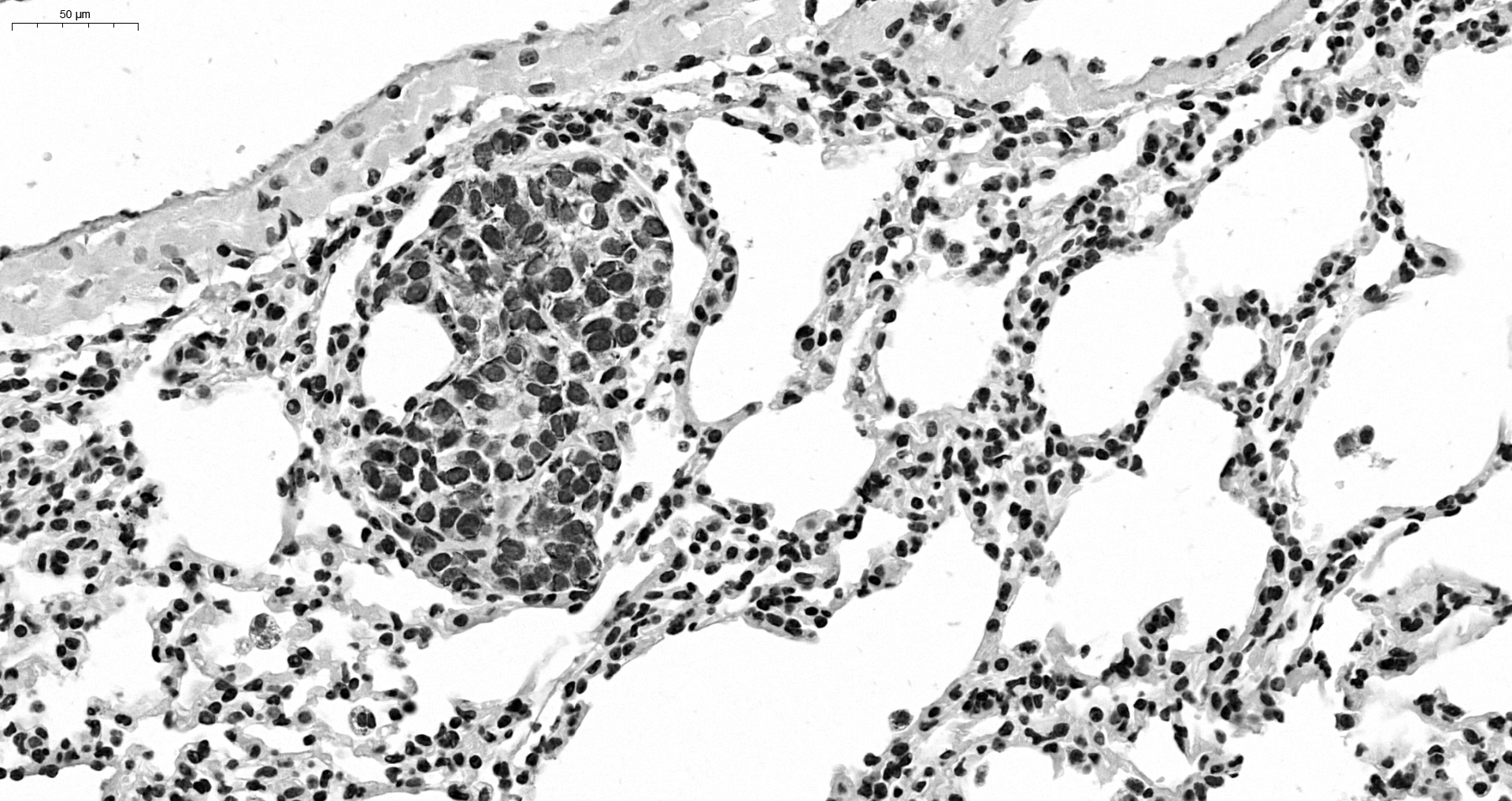

Supplement: Supplementary file 1 — Additional file 1. Raw data. [file 12935_2023_3076_MOESM1_ESM.zip › raw_data/IHC/vimentin/ΓæíSaoS-2+si-DIO3OS/3-2 ΓæíSaoS-2+si-DIO3OS.tif]

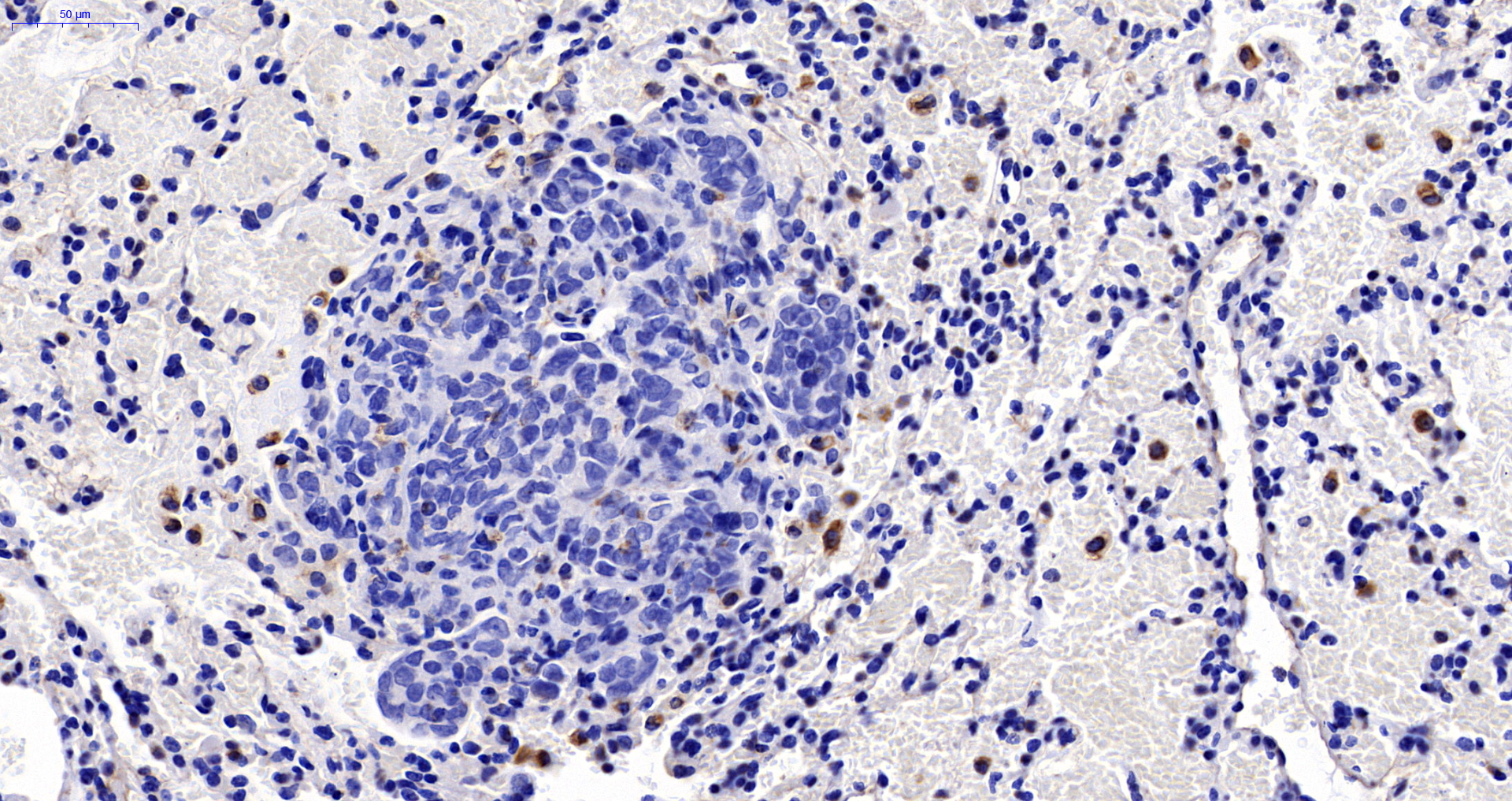

Supplement: Supplementary file 1 — Additional file 1. Raw data. [file 12935_2023_3076_MOESM1_ESM.zip › raw_data/IHC/vimentin/ΓæíSaoS-2+si-DIO3OS/1-1 ΓæíSaoS-2+si-DIO3OS.jpg]

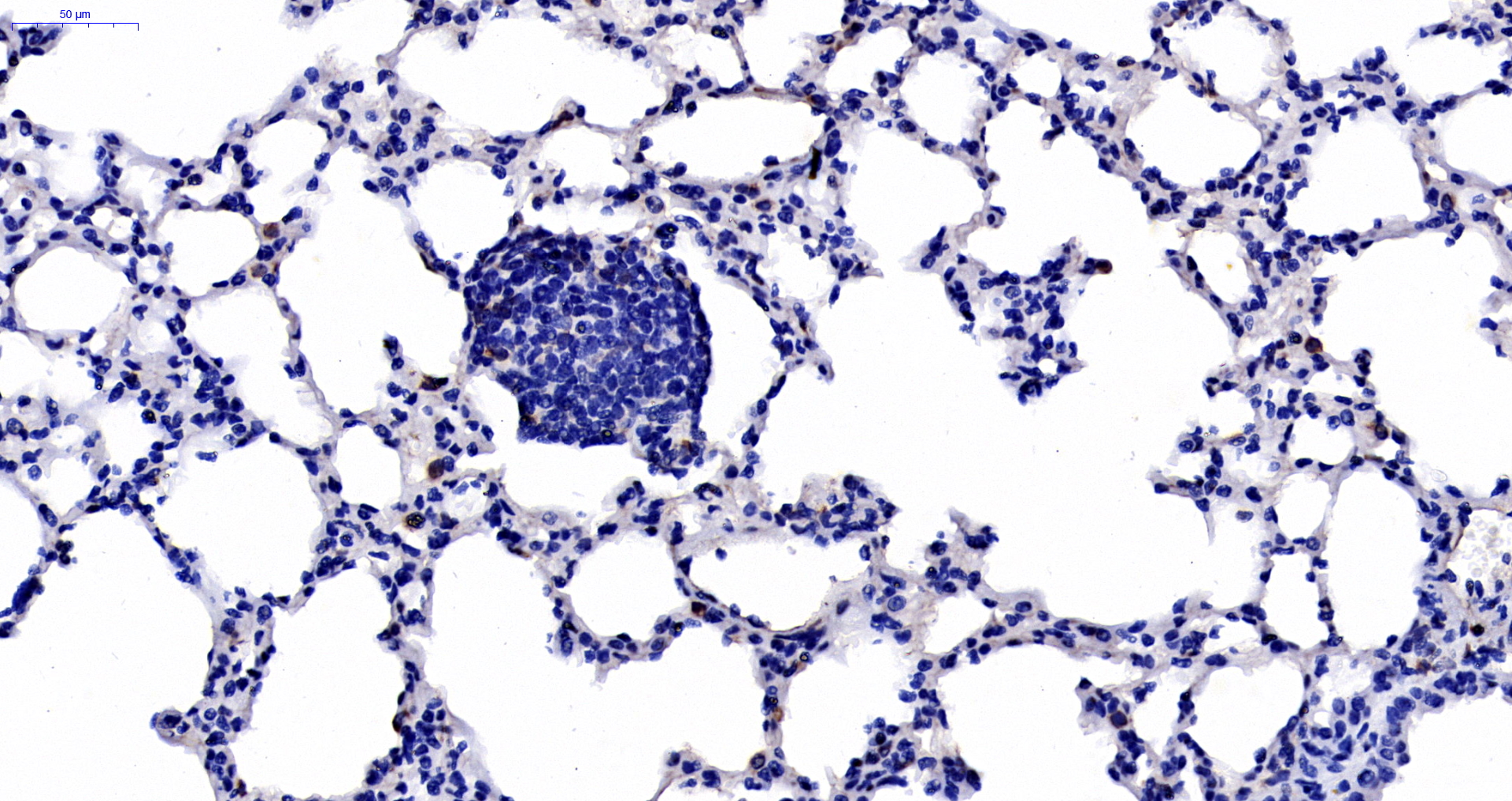

Supplement: Supplementary file 1 — Additional file 1. Raw data. [file 12935_2023_3076_MOESM1_ESM.zip › raw_data/IHC/vimentin/ΓæíSaoS-2+si-DIO3OS/2-1 ΓæíSaoS-2+si-DIO3OS.jpg]

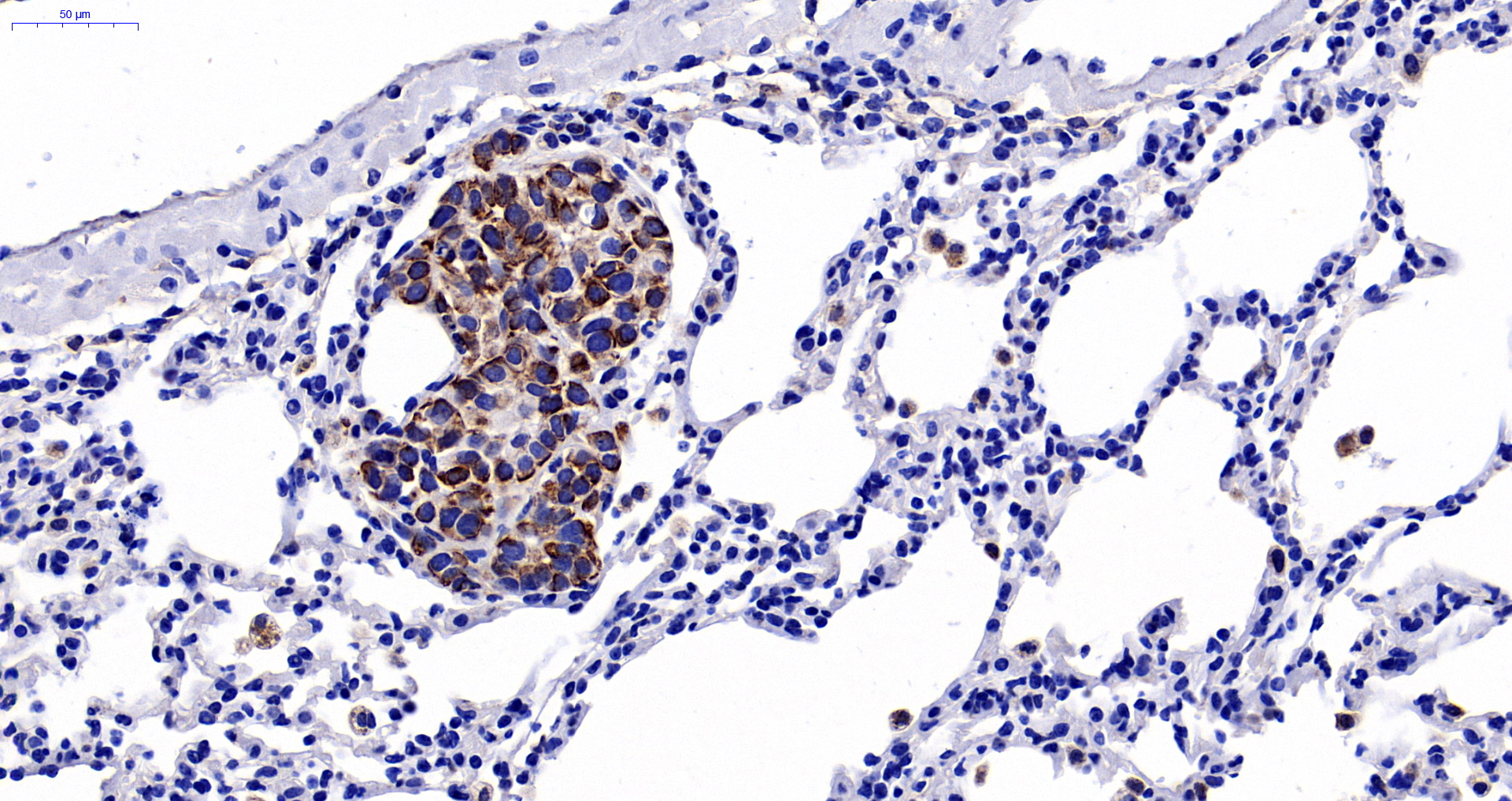

Supplement: Supplementary file 1 — Additional file 1. Raw data. [file 12935_2023_3076_MOESM1_ESM.zip › raw_data/IHC/vimentin/ΓæíSaoS-2+si-DIO3OS/3-2 ΓæíSaoS-2+si-DIO3OS.jpg]

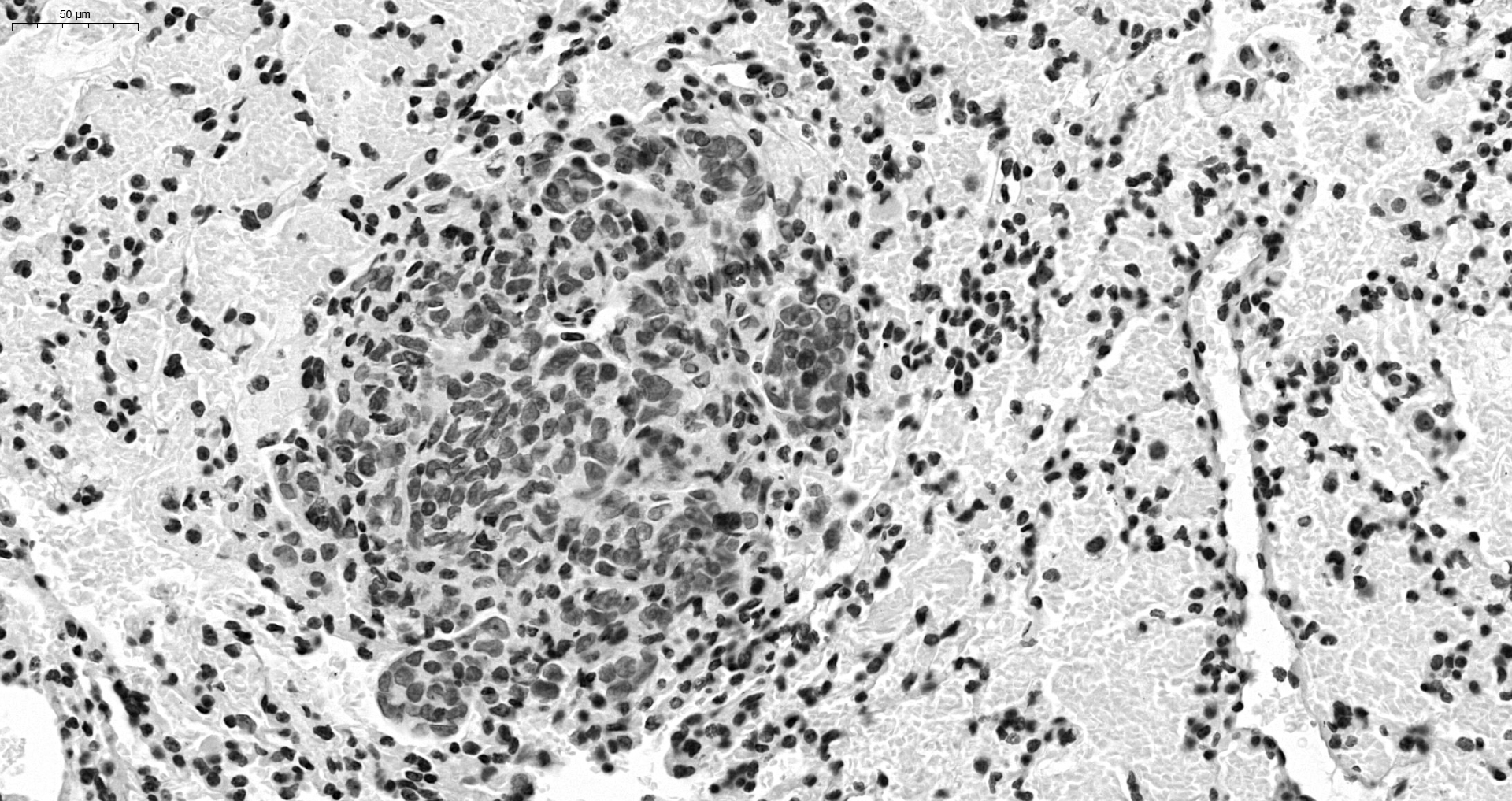

Supplement: Supplementary file 1 — Additional file 1. Raw data. [file 12935_2023_3076_MOESM1_ESM.zip › raw_data/IHC/vimentin/ΓæíSaoS-2+si-DIO3OS/1-1 ΓæíSaoS-2+si-DIO3OS.tif]

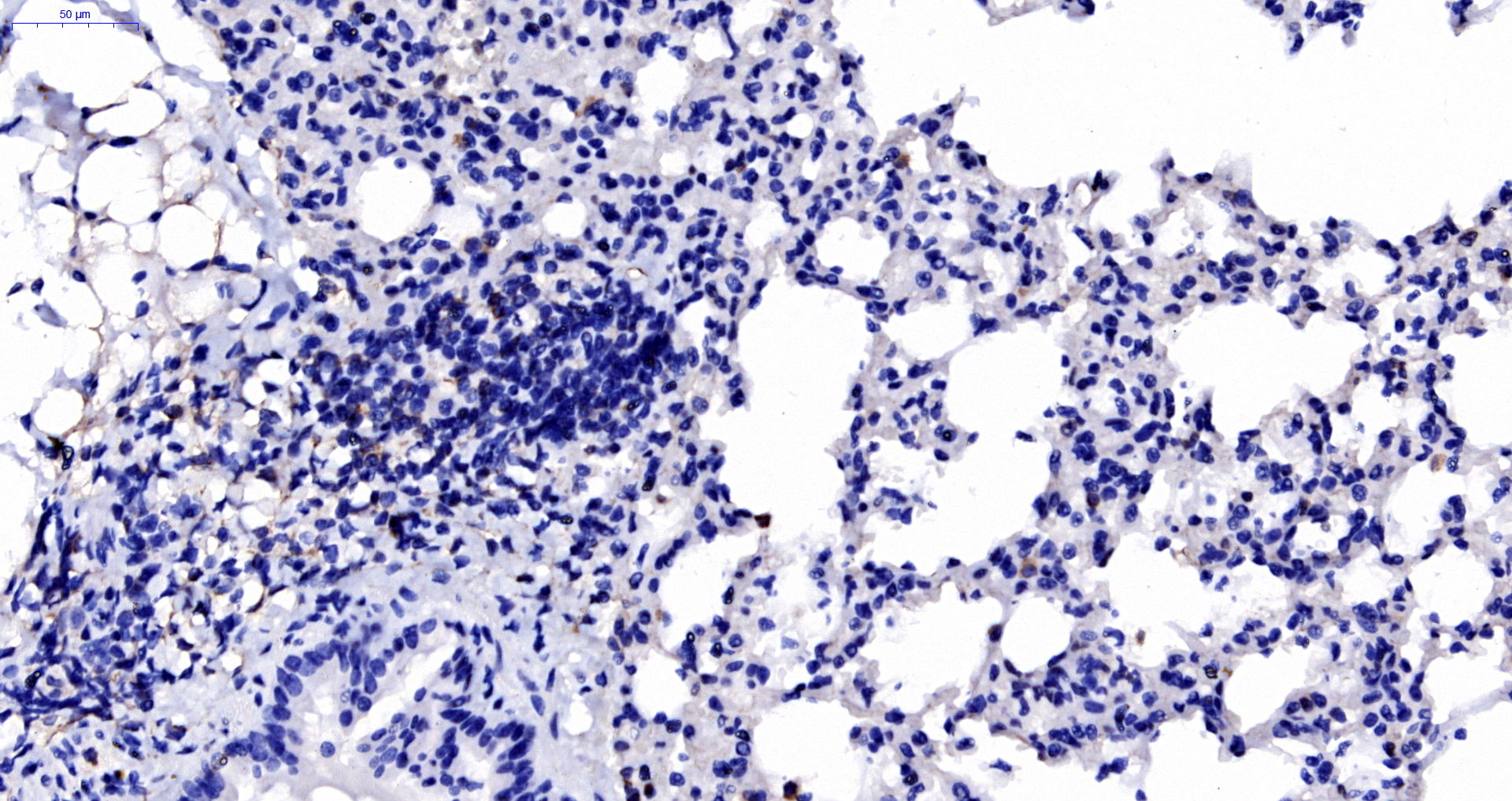

Supplement: Supplementary file 1 — Additional file 1. Raw data. [file 12935_2023_3076_MOESM1_ESM.zip › raw_data/IHC/vimentin/ΓæíSaoS-2+si-DIO3OS/2-3 ΓæíSaoS-2+si-DIO3OS.jpg]

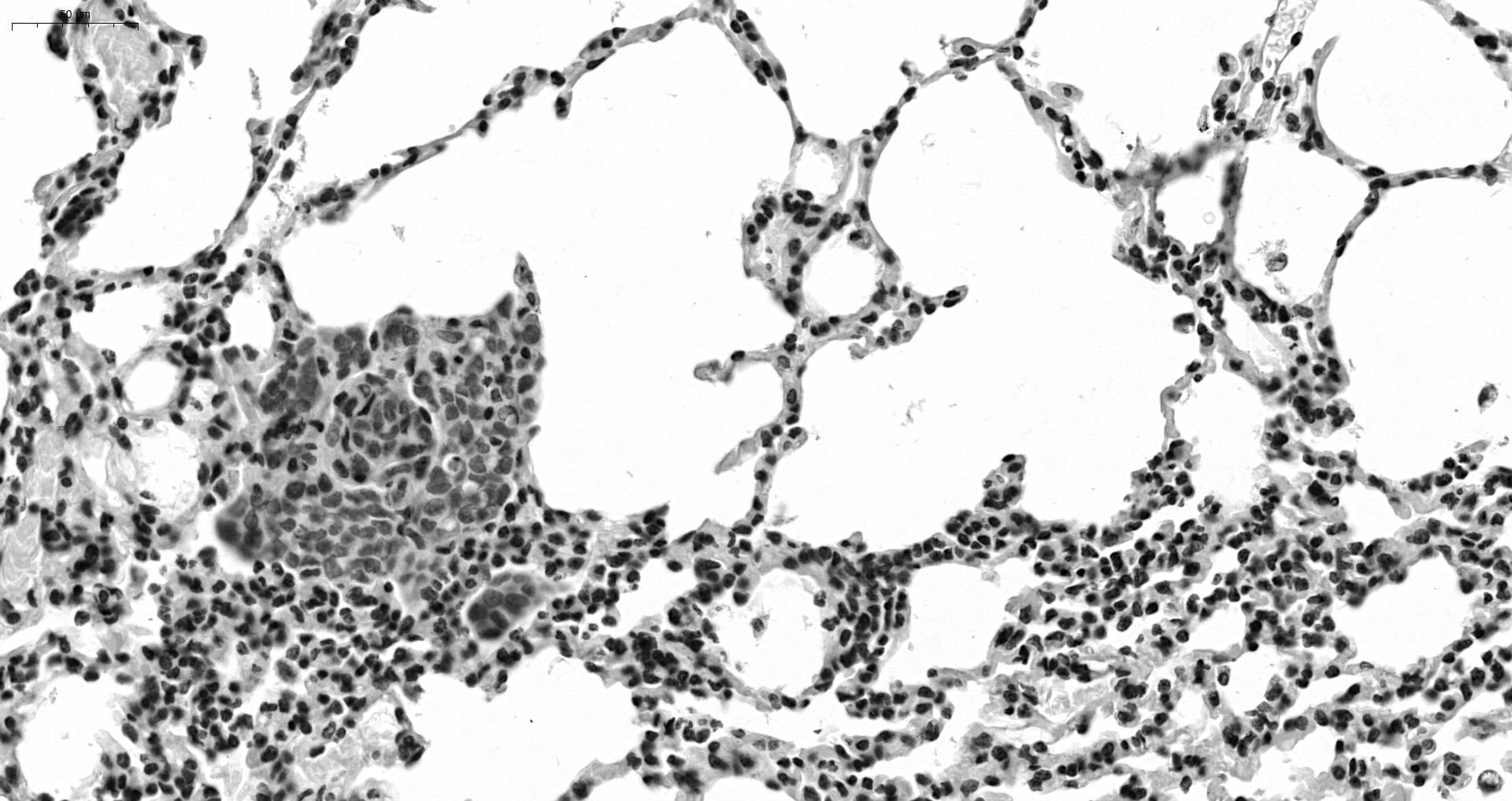

Supplement: Supplementary file 1 — Additional file 1. Raw data. [file 12935_2023_3076_MOESM1_ESM.zip › raw_data/IHC/vimentin/ΓæíSaoS-2+si-DIO3OS/1-3 ΓæíSaoS-2+si-DIO3OS.tif]

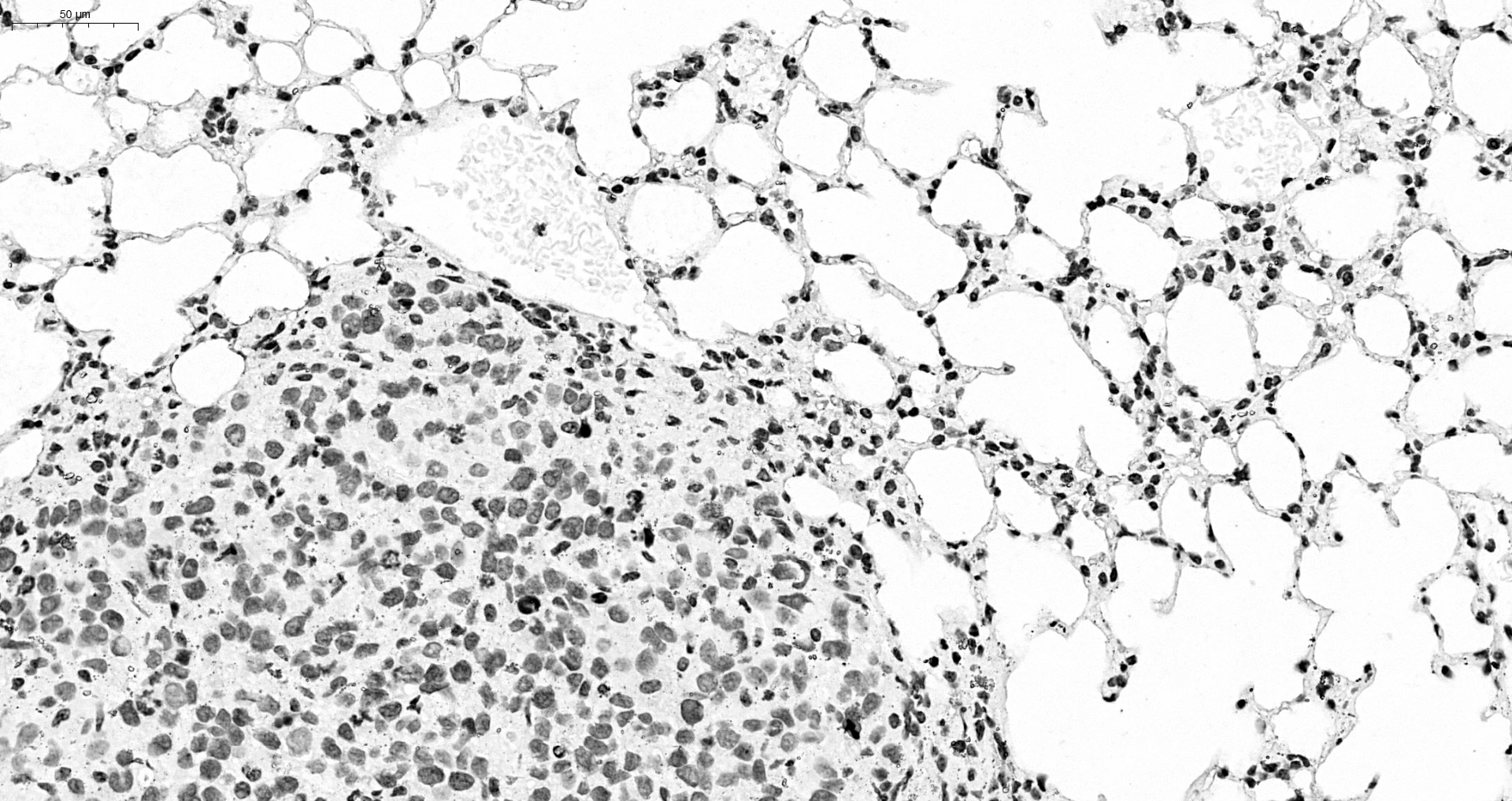

Supplement: Supplementary file 1 — Additional file 1. Raw data. [file 12935_2023_3076_MOESM1_ESM.zip › raw_data/IHC/E-cad/ΓæáSaoS-2+si-NC/1-2 ΓæáSaoS-2+si-NC.tif]

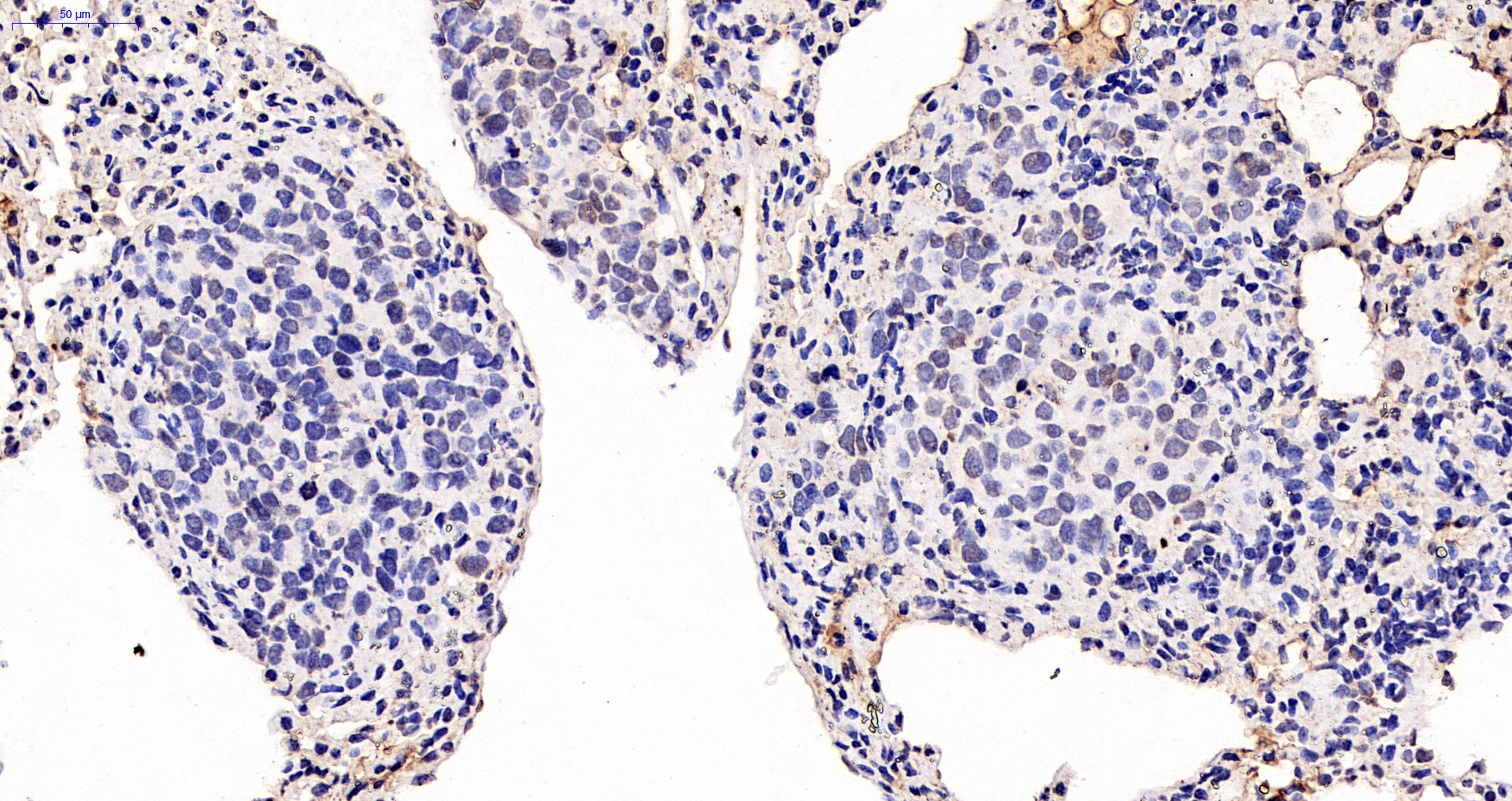

Supplement: Supplementary file 1 — Additional file 1. Raw data. [file 12935_2023_3076_MOESM1_ESM.zip › raw_data/IHC/E-cad/ΓæáSaoS-2+si-NC/1-1 ΓæáSaoS-2+si-NC.jpg]

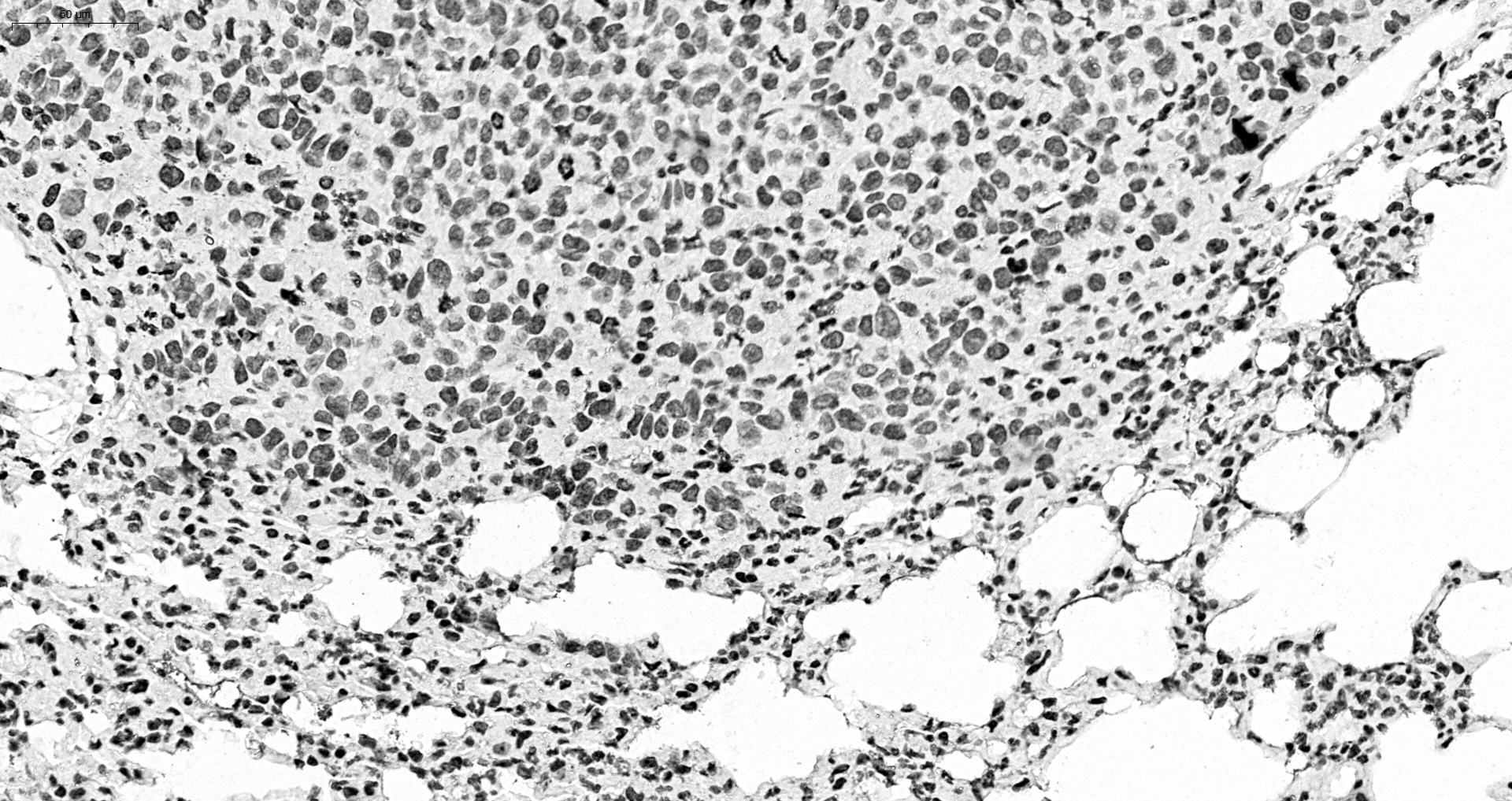

Supplement: Supplementary file 1 — Additional file 1. Raw data. [file 12935_2023_3076_MOESM1_ESM.zip › raw_data/IHC/E-cad/ΓæáSaoS-2+si-NC/3-2 ΓæáSaoS-2+si-NC.tif]

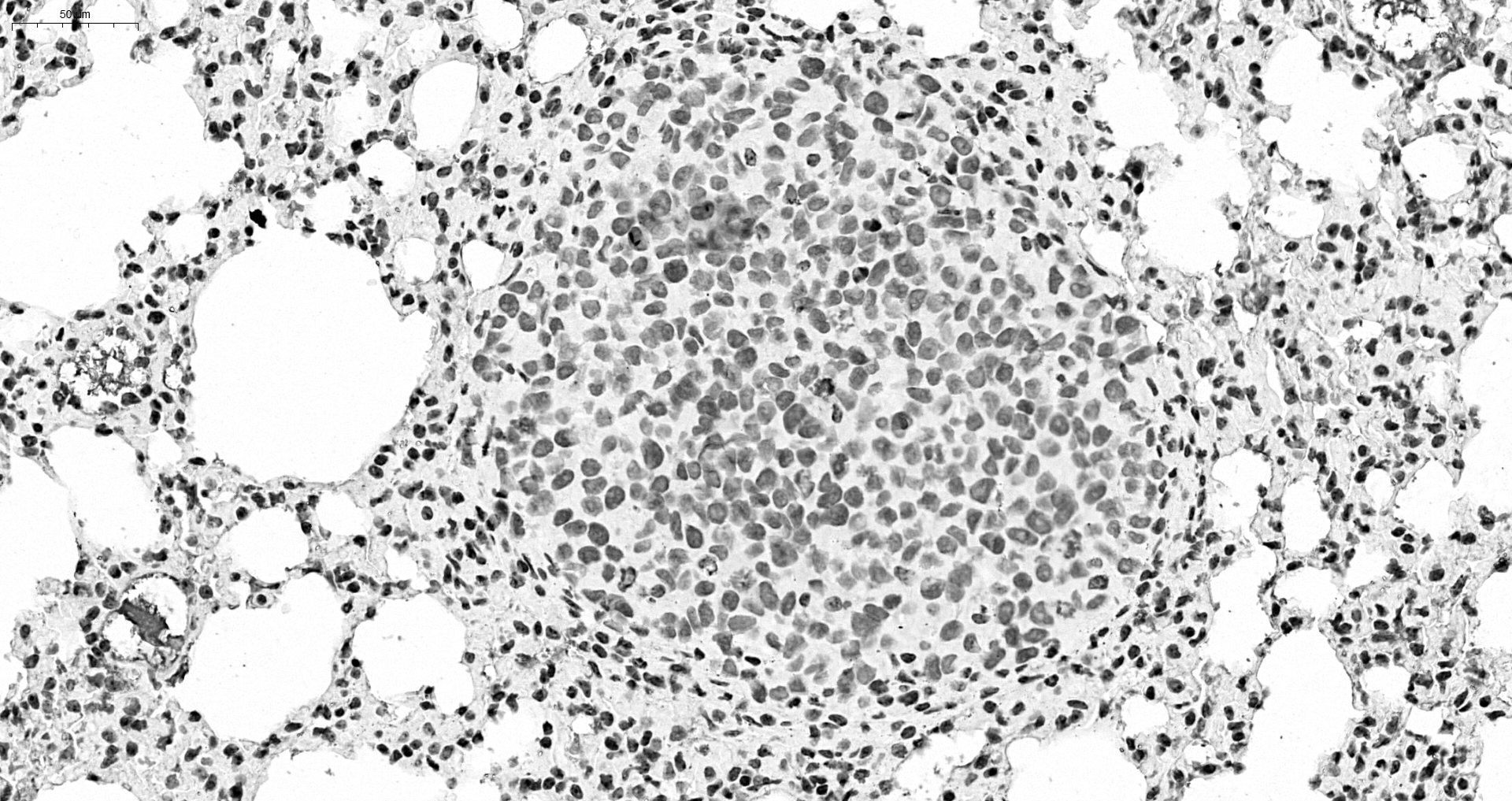

Supplement: Supplementary file 1 — Additional file 1. Raw data. [file 12935_2023_3076_MOESM1_ESM.zip › raw_data/IHC/E-cad/ΓæáSaoS-2+si-NC/2-2 ΓæáSaoS-2+si-NC.tif]

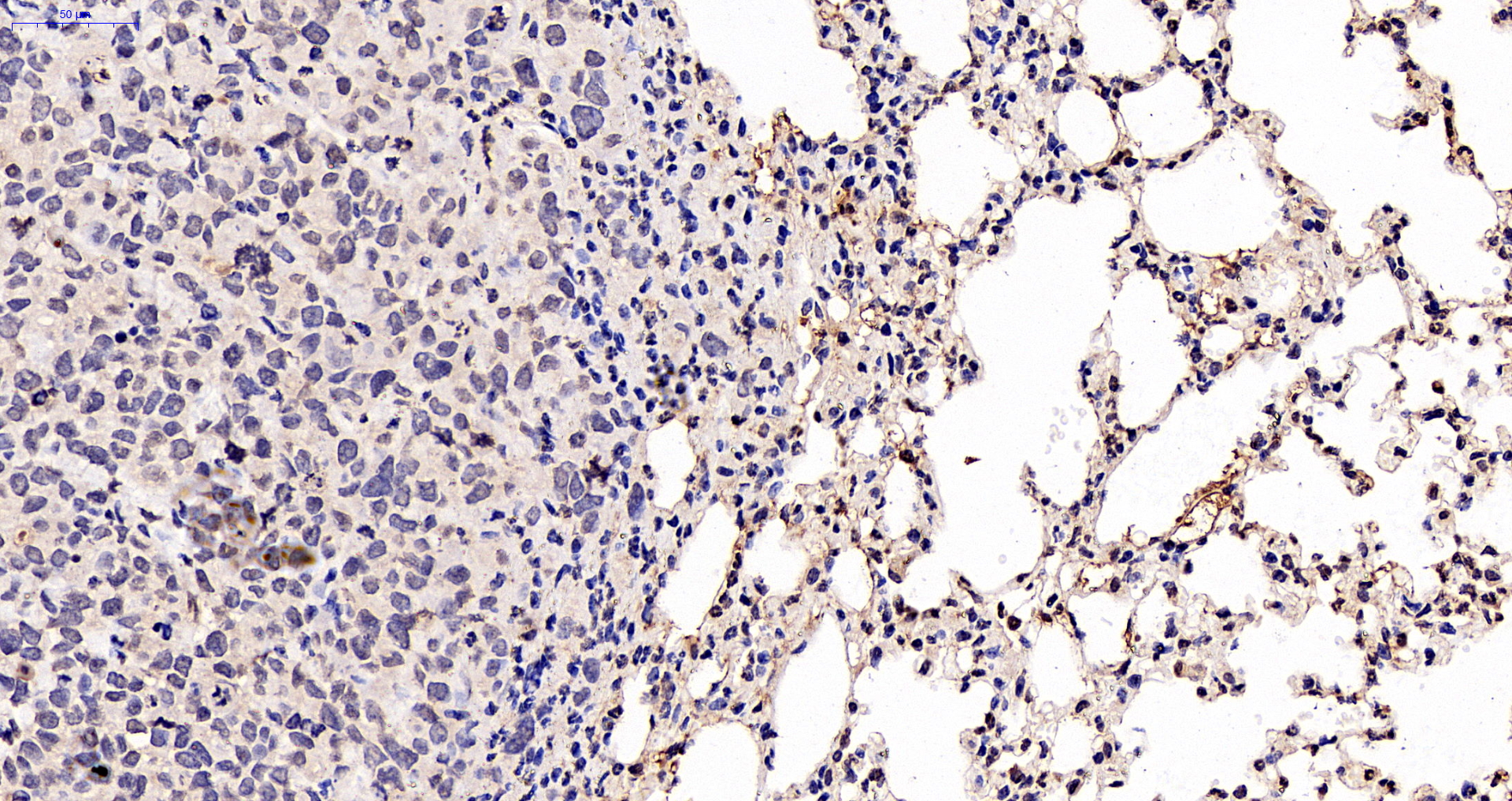

Supplement: Supplementary file 1 — Additional file 1. Raw data. [file 12935_2023_3076_MOESM1_ESM.zip › raw_data/IHC/E-cad/ΓæáSaoS-2+si-NC/3-1 ΓæáSaoS-2+si-NC.jpg]

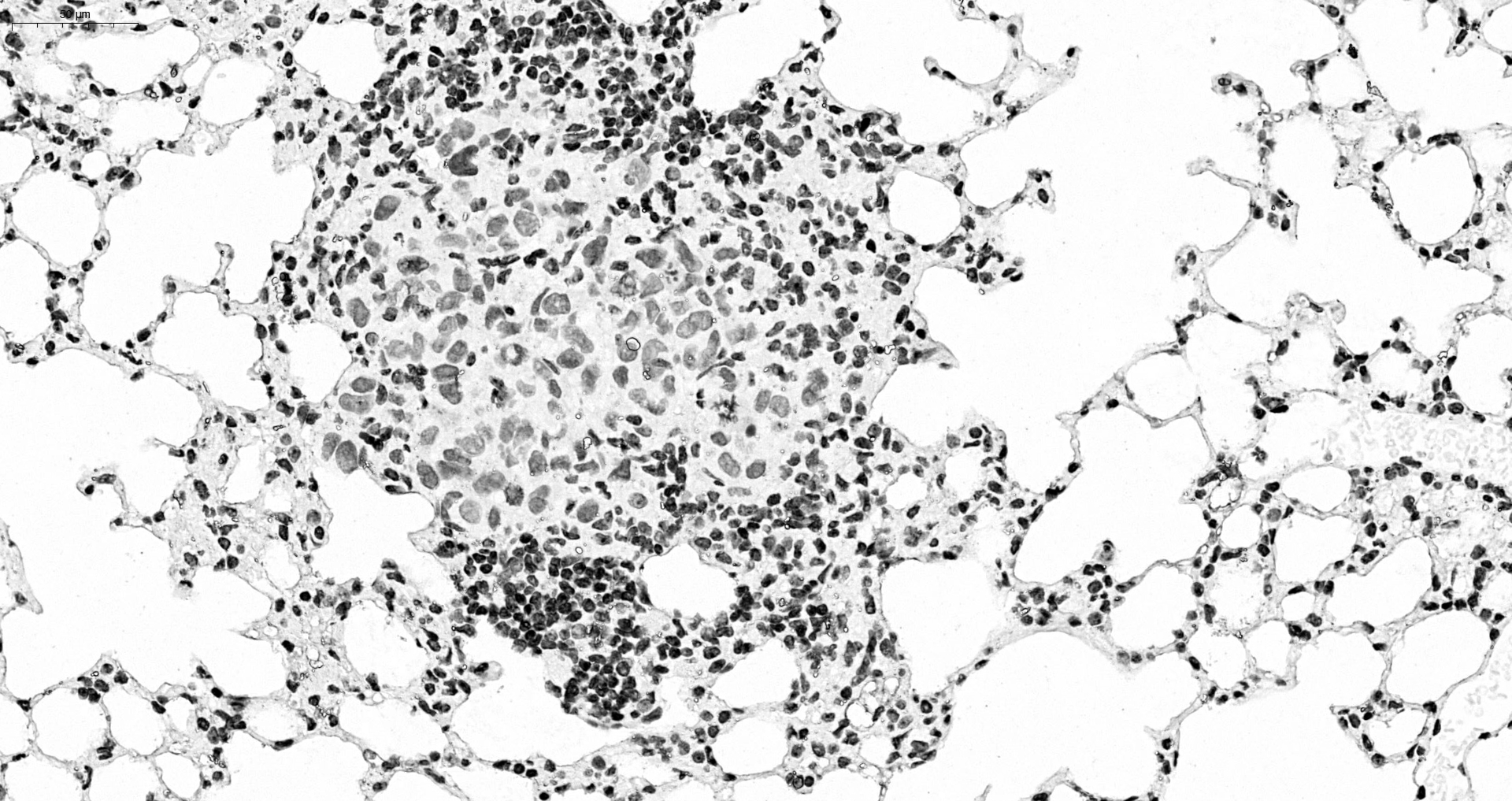

Supplement: Supplementary file 1 — Additional file 1. Raw data. [file 12935_2023_3076_MOESM1_ESM.zip › raw_data/IHC/E-cad/ΓæáSaoS-2+si-NC/1-3 ΓæáSaoS-2+si-NC.tif]

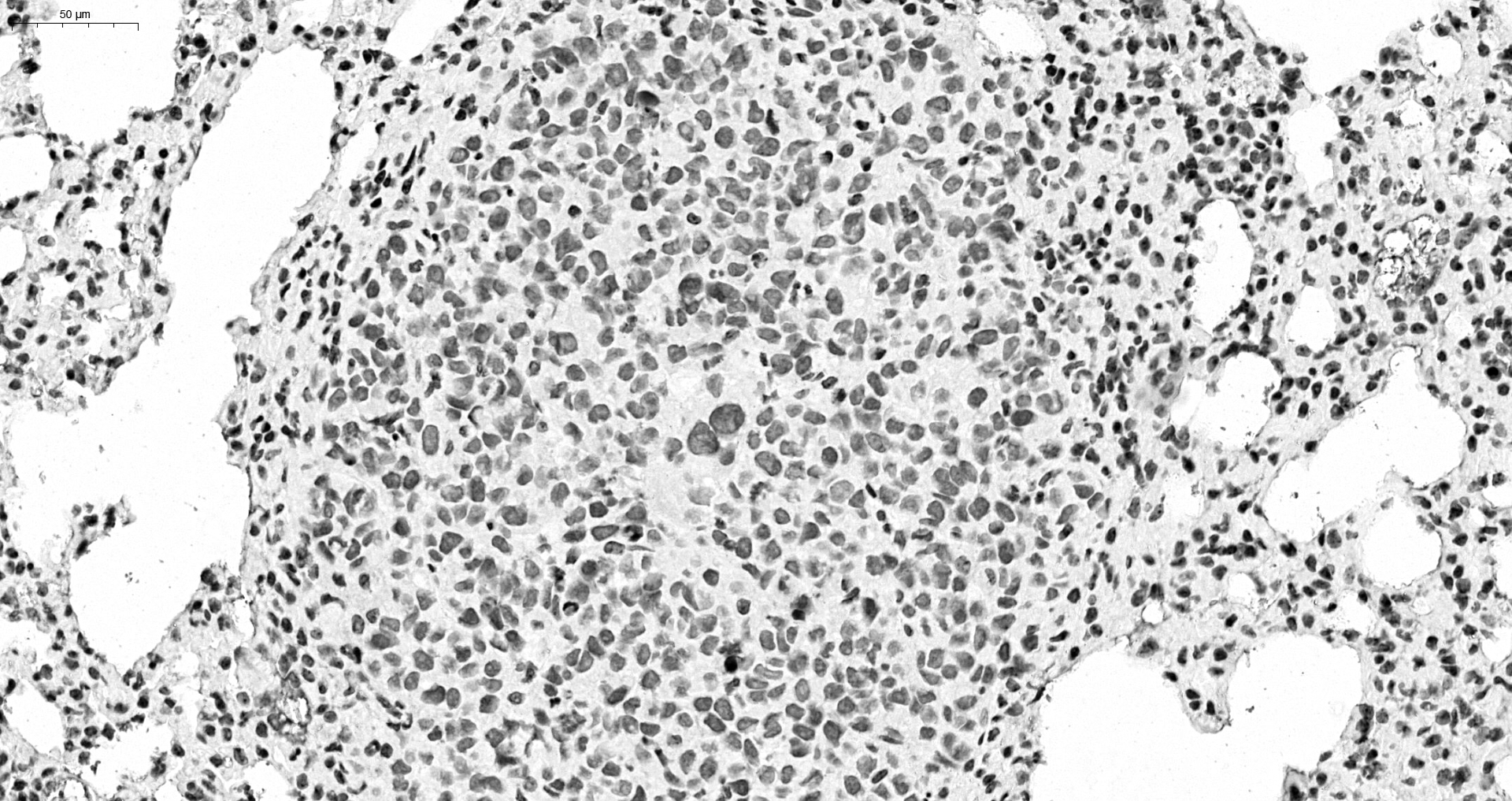

Supplement: Supplementary file 1 — Additional file 1. Raw data. [file 12935_2023_3076_MOESM1_ESM.zip › raw_data/IHC/E-cad/ΓæáSaoS-2+si-NC/2-3 ΓæáSaoS-2+si-NC.tif]

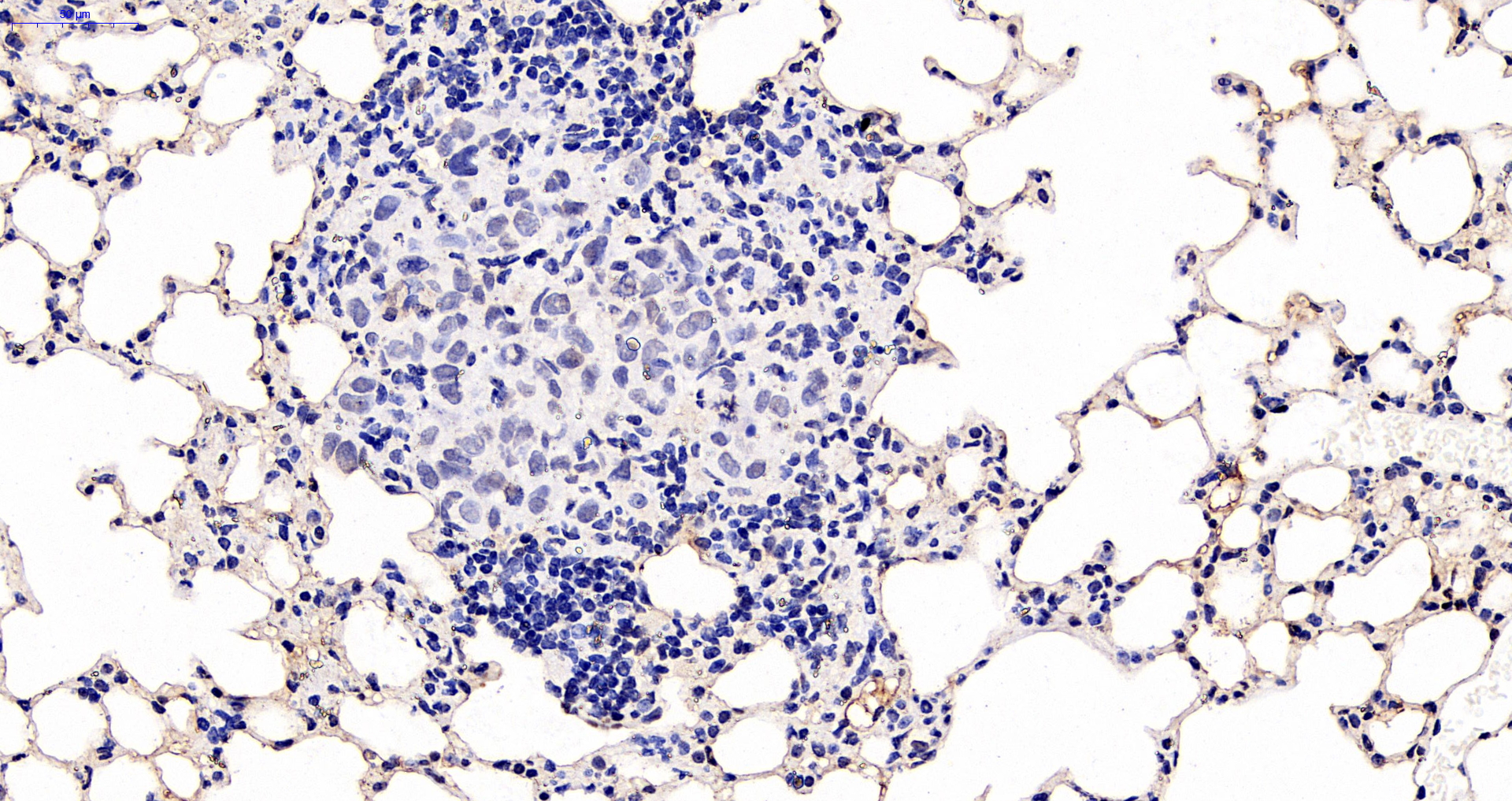

Supplement: Supplementary file 1 — Additional file 1. Raw data. [file 12935_2023_3076_MOESM1_ESM.zip › raw_data/IHC/E-cad/ΓæáSaoS-2+si-NC/1-3 ΓæáSaoS-2+si-NC.jpg]

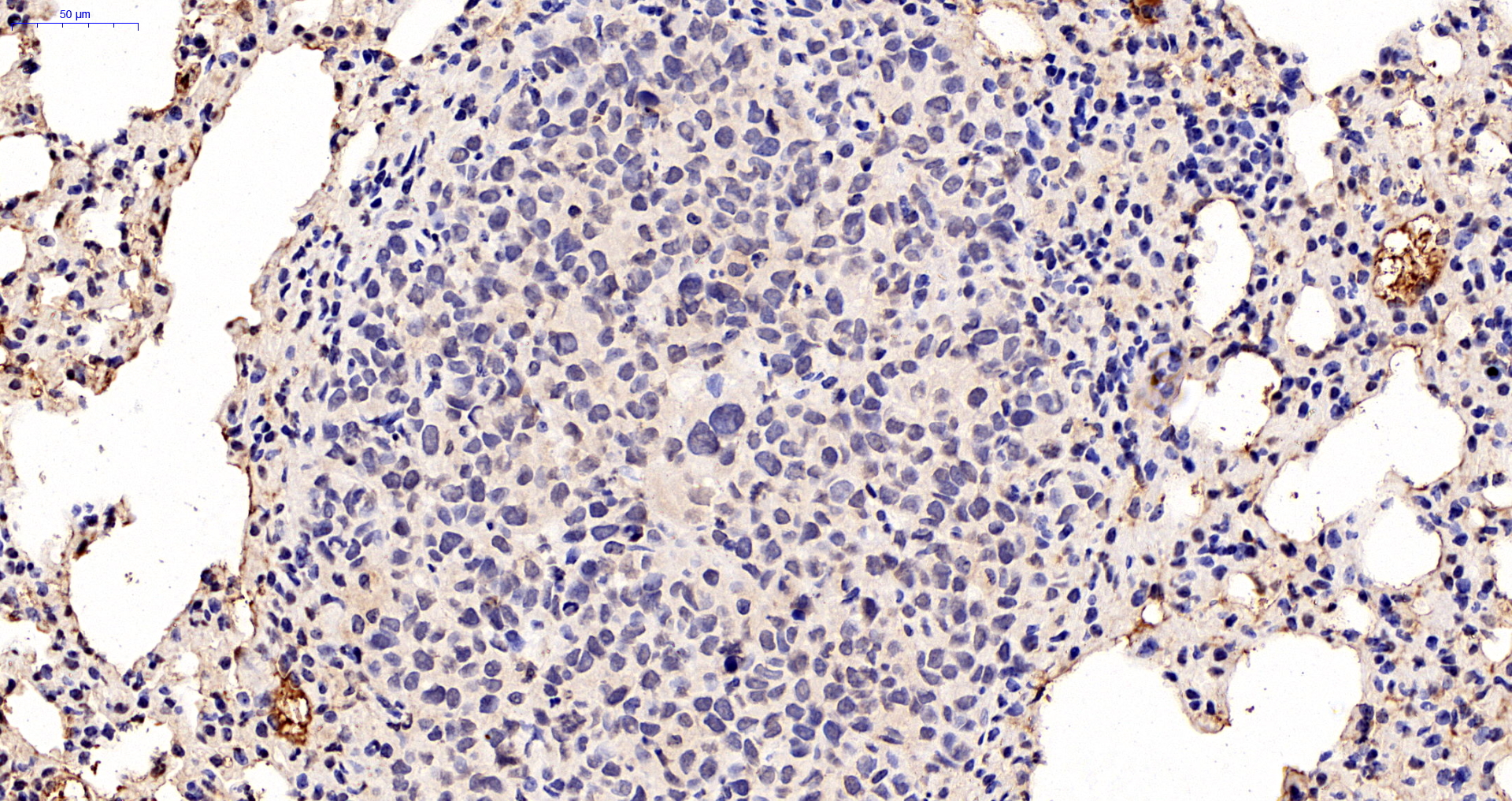

Supplement: Supplementary file 1 — Additional file 1. Raw data. [file 12935_2023_3076_MOESM1_ESM.zip › raw_data/IHC/E-cad/ΓæáSaoS-2+si-NC/2-3 ΓæáSaoS-2+si-NC.jpg]

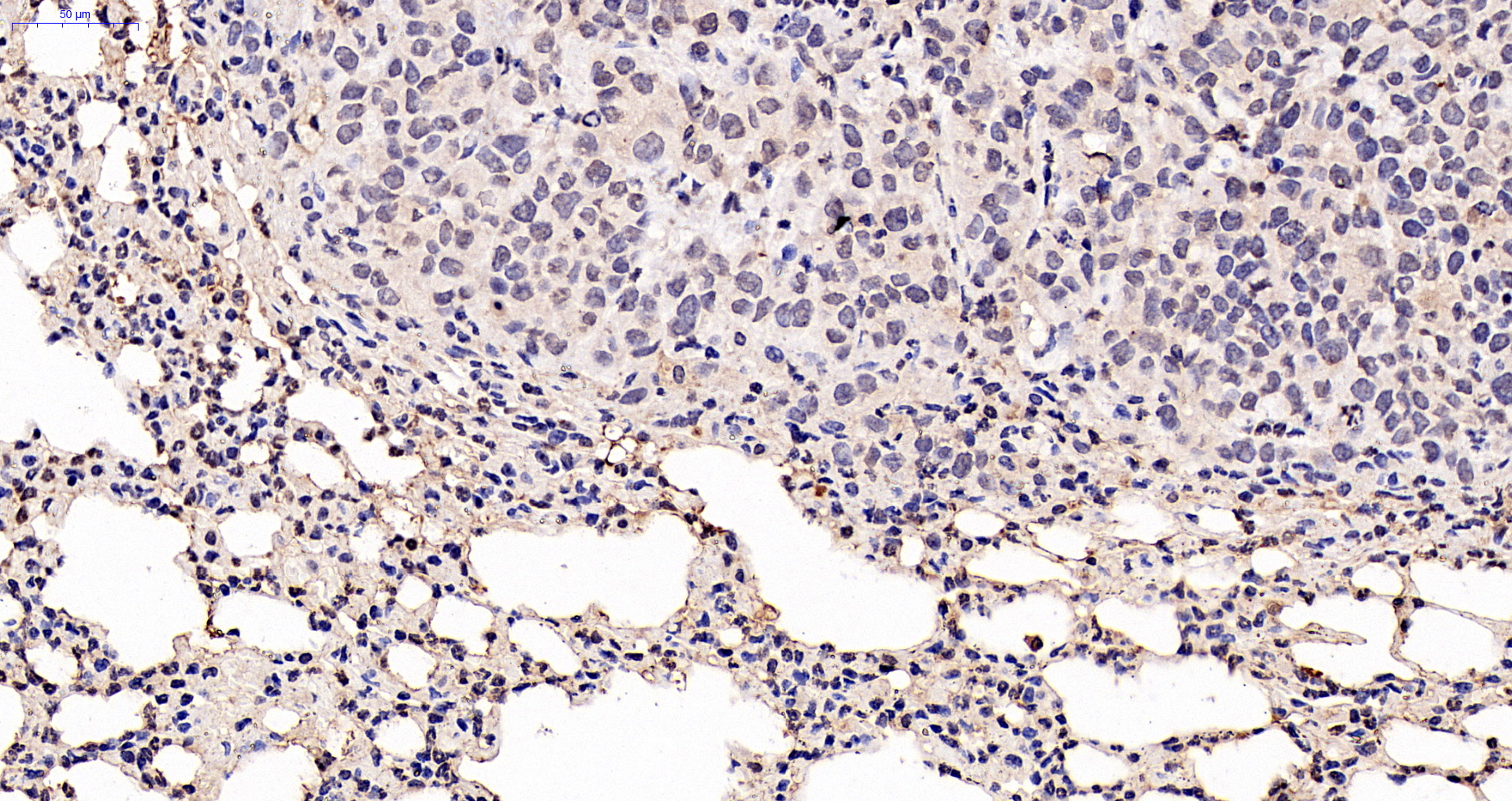

Supplement: Supplementary file 1 — Additional file 1. Raw data. [file 12935_2023_3076_MOESM1_ESM.zip › raw_data/IHC/E-cad/ΓæáSaoS-2+si-NC/3-3 ΓæáSaoS-2+si-NC.jpg]

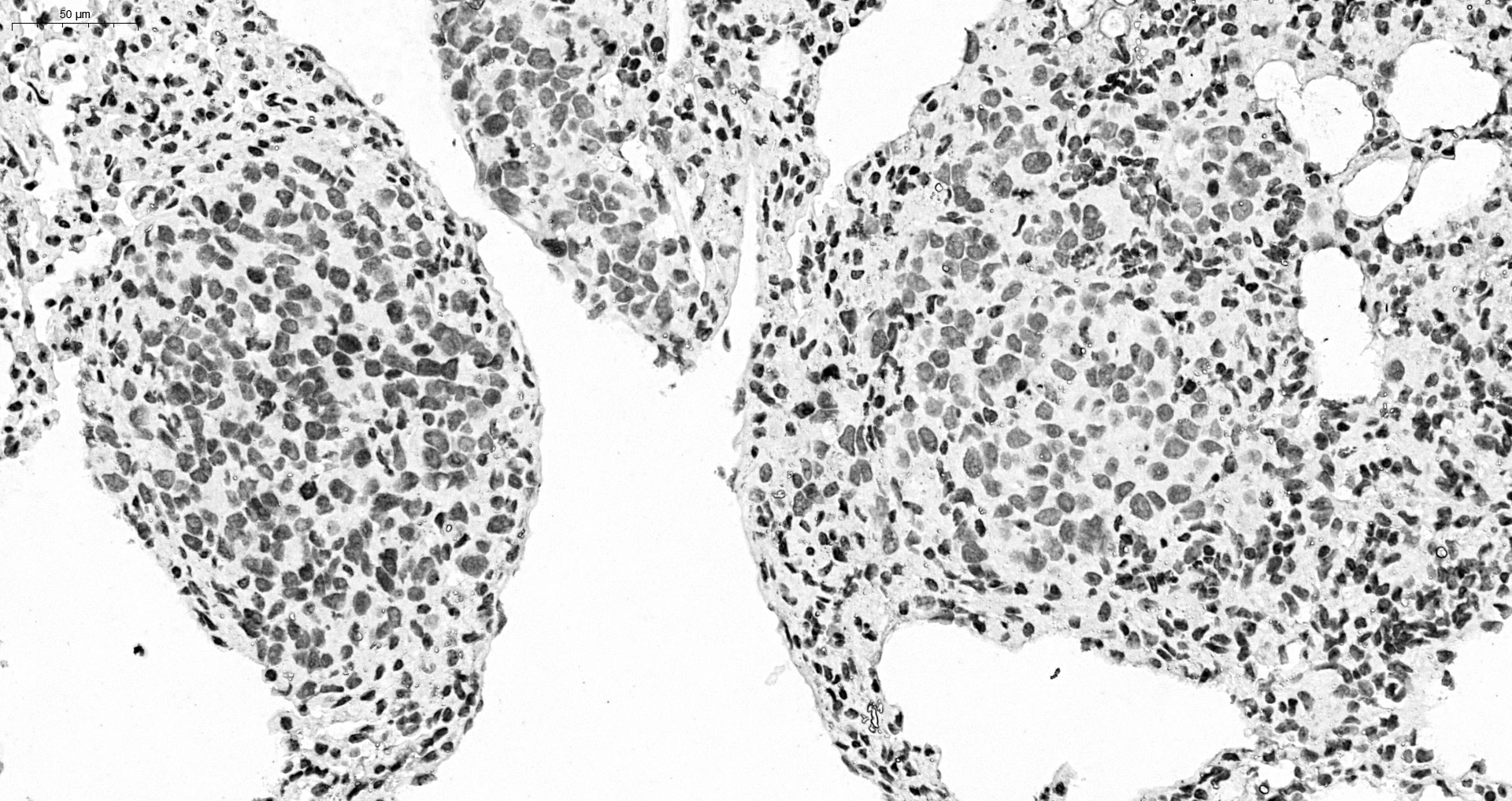

Supplement: Supplementary file 1 — Additional file 1. Raw data. [file 12935_2023_3076_MOESM1_ESM.zip › raw_data/IHC/E-cad/ΓæáSaoS-2+si-NC/1-1 ΓæáSaoS-2+si-NC.tif]

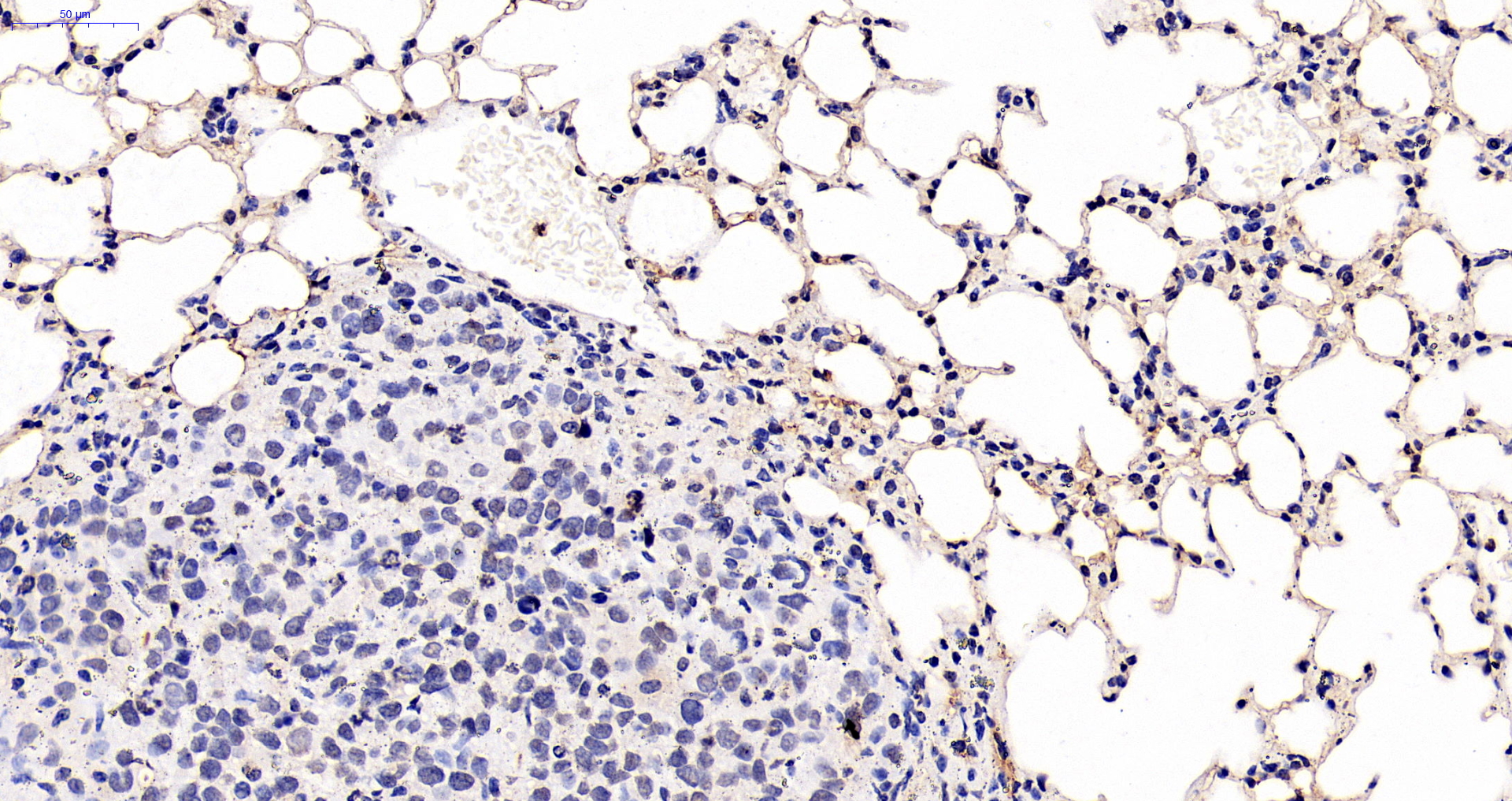

Supplement: Supplementary file 1 — Additional file 1. Raw data. [file 12935_2023_3076_MOESM1_ESM.zip › raw_data/IHC/E-cad/ΓæáSaoS-2+si-NC/1-2 ΓæáSaoS-2+si-NC.jpg]

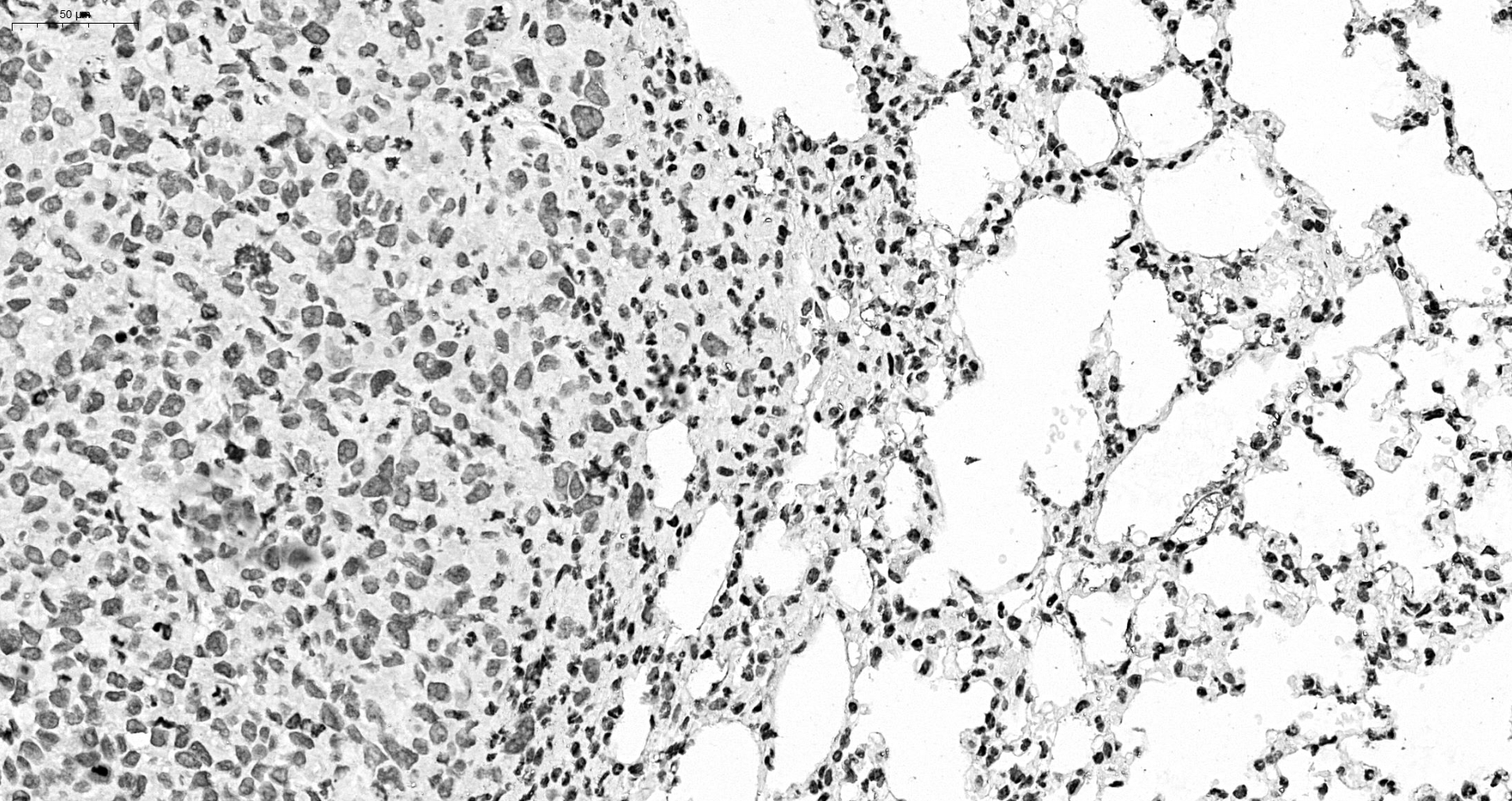

Supplement: Supplementary file 1 — Additional file 1. Raw data. [file 12935_2023_3076_MOESM1_ESM.zip › raw_data/IHC/E-cad/ΓæáSaoS-2+si-NC/3-1 ΓæáSaoS-2+si-NC.tif]

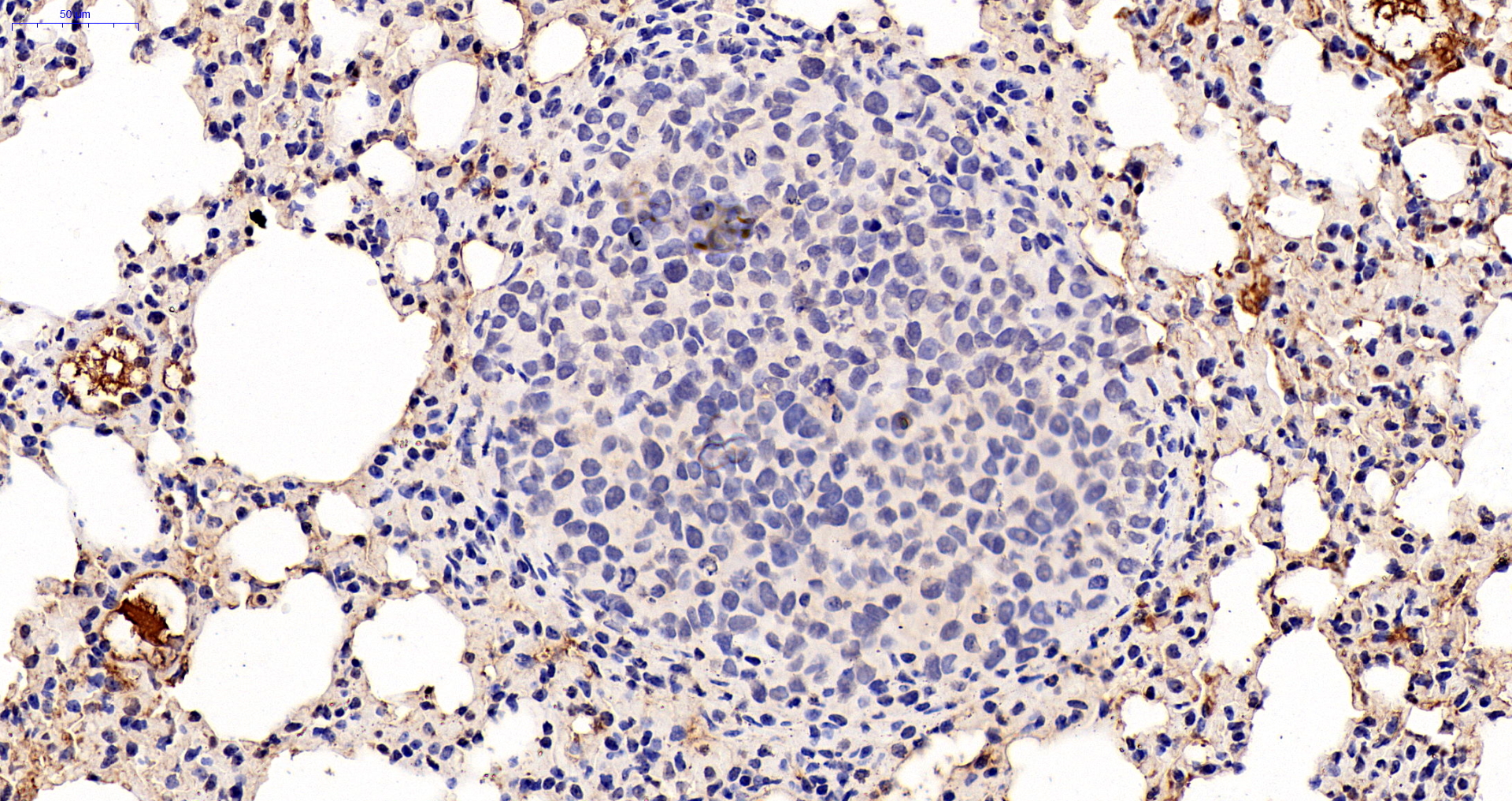

Supplement: Supplementary file 1 — Additional file 1. Raw data. [file 12935_2023_3076_MOESM1_ESM.zip › raw_data/IHC/E-cad/ΓæáSaoS-2+si-NC/2-2 ΓæáSaoS-2+si-NC.jpg]

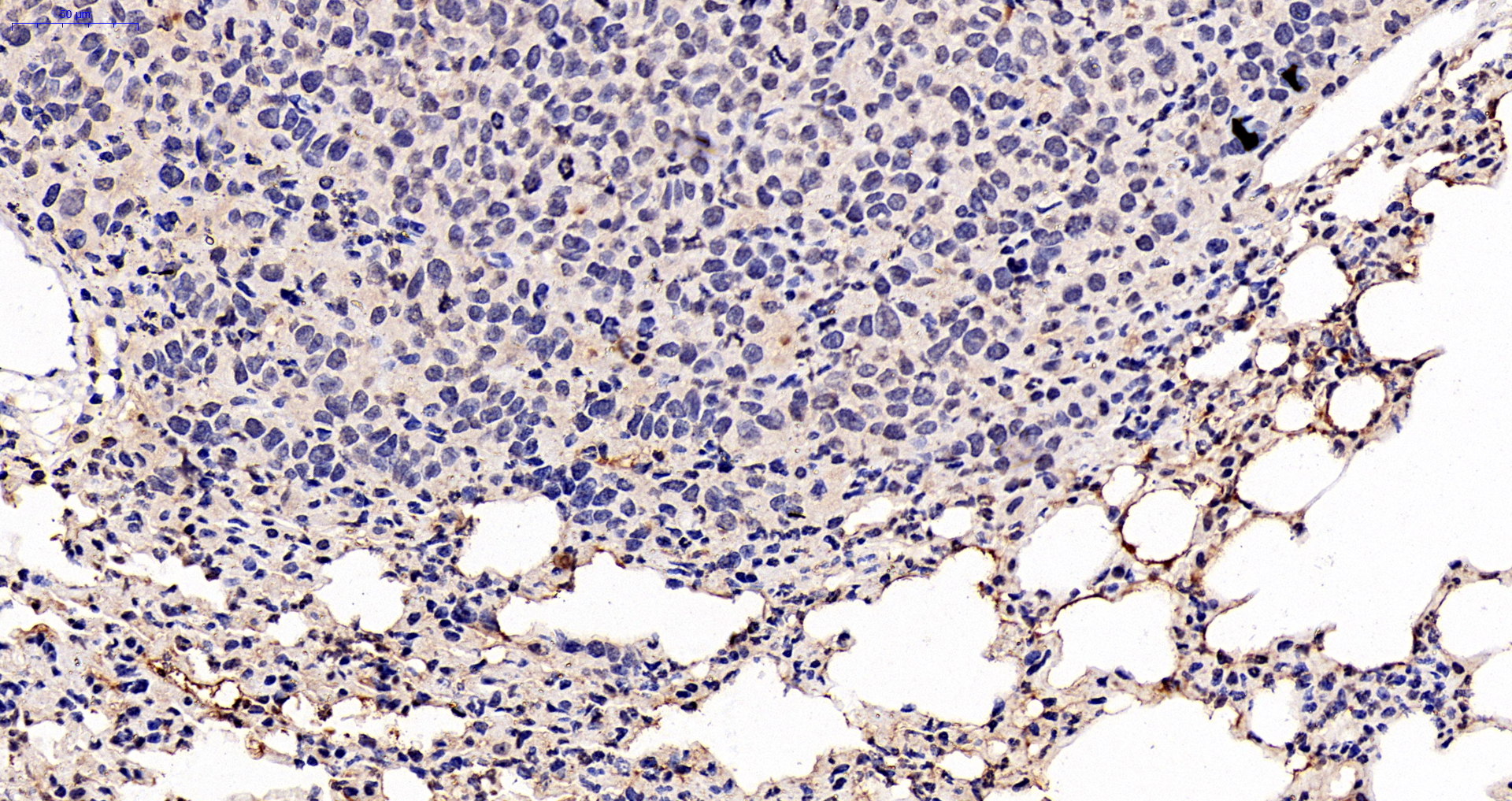

Supplement: Supplementary file 1 — Additional file 1. Raw data. [file 12935_2023_3076_MOESM1_ESM.zip › raw_data/IHC/E-cad/ΓæáSaoS-2+si-NC/3-2 ΓæáSaoS-2+si-NC.jpg]

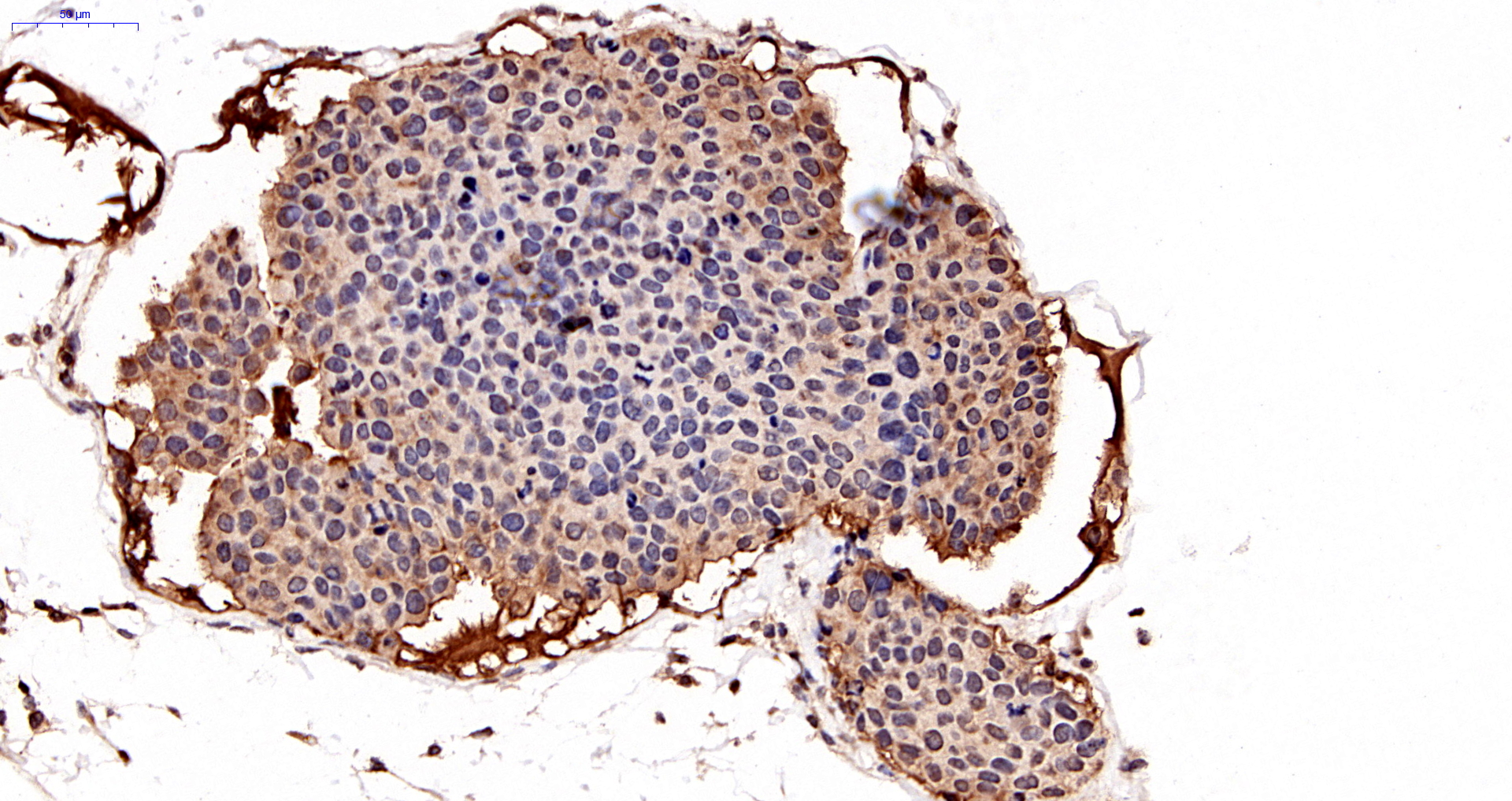

Supplement: Supplementary file 1 — Additional file 1. Raw data. [file 12935_2023_3076_MOESM1_ESM.zip › raw_data/IHC/E-cad/ΓæíSaoS-2+si-DIO3OS/1-2 ΓæíSaoS-2+si-DIO3OS.jpg]

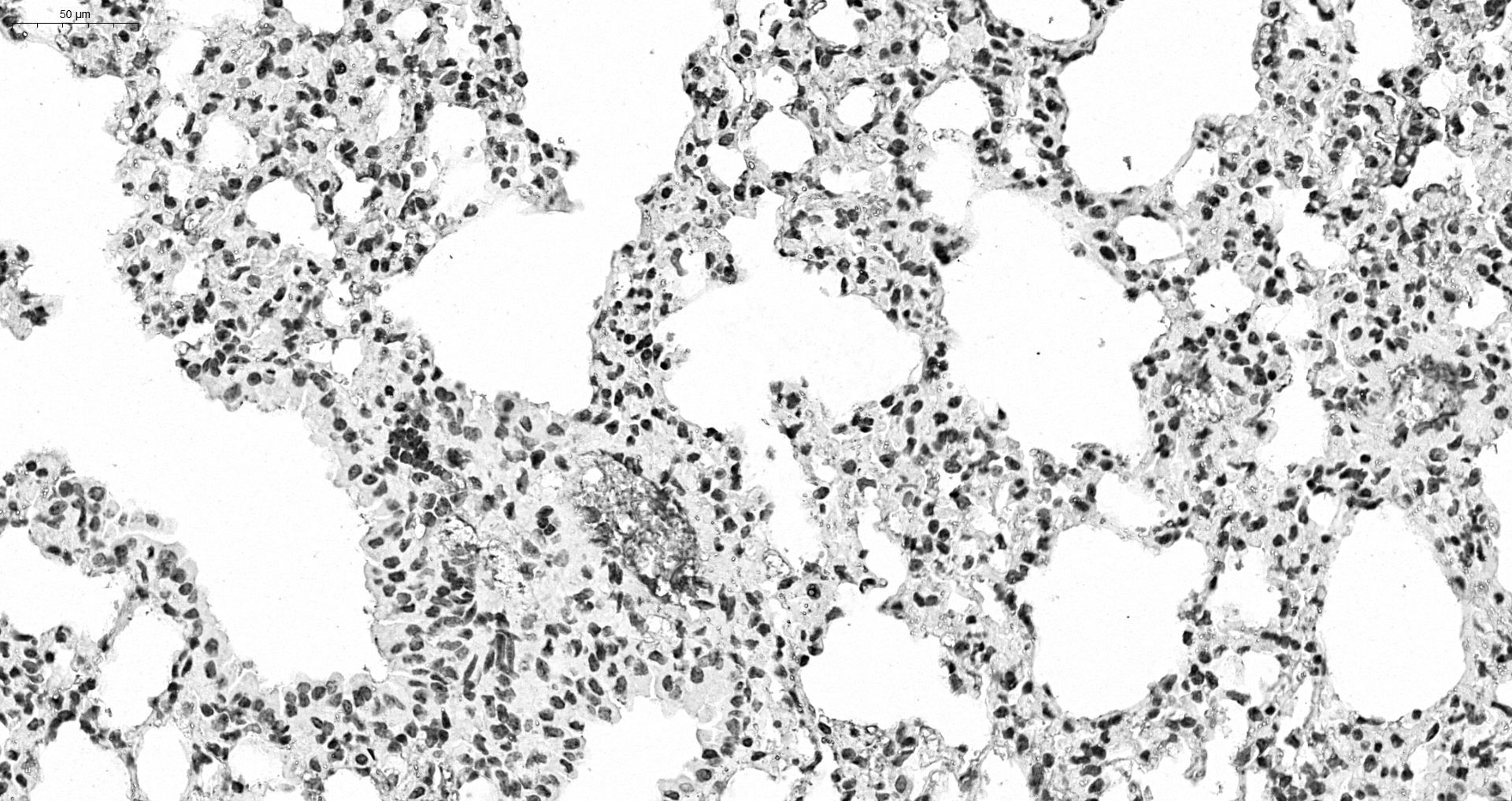

Supplement: Supplementary file 1 — Additional file 1. Raw data. [file 12935_2023_3076_MOESM1_ESM.zip › raw_data/IHC/E-cad/ΓæíSaoS-2+si-DIO3OS/2-2 ΓæíSaoS-2+si-DIO3OS.tif]

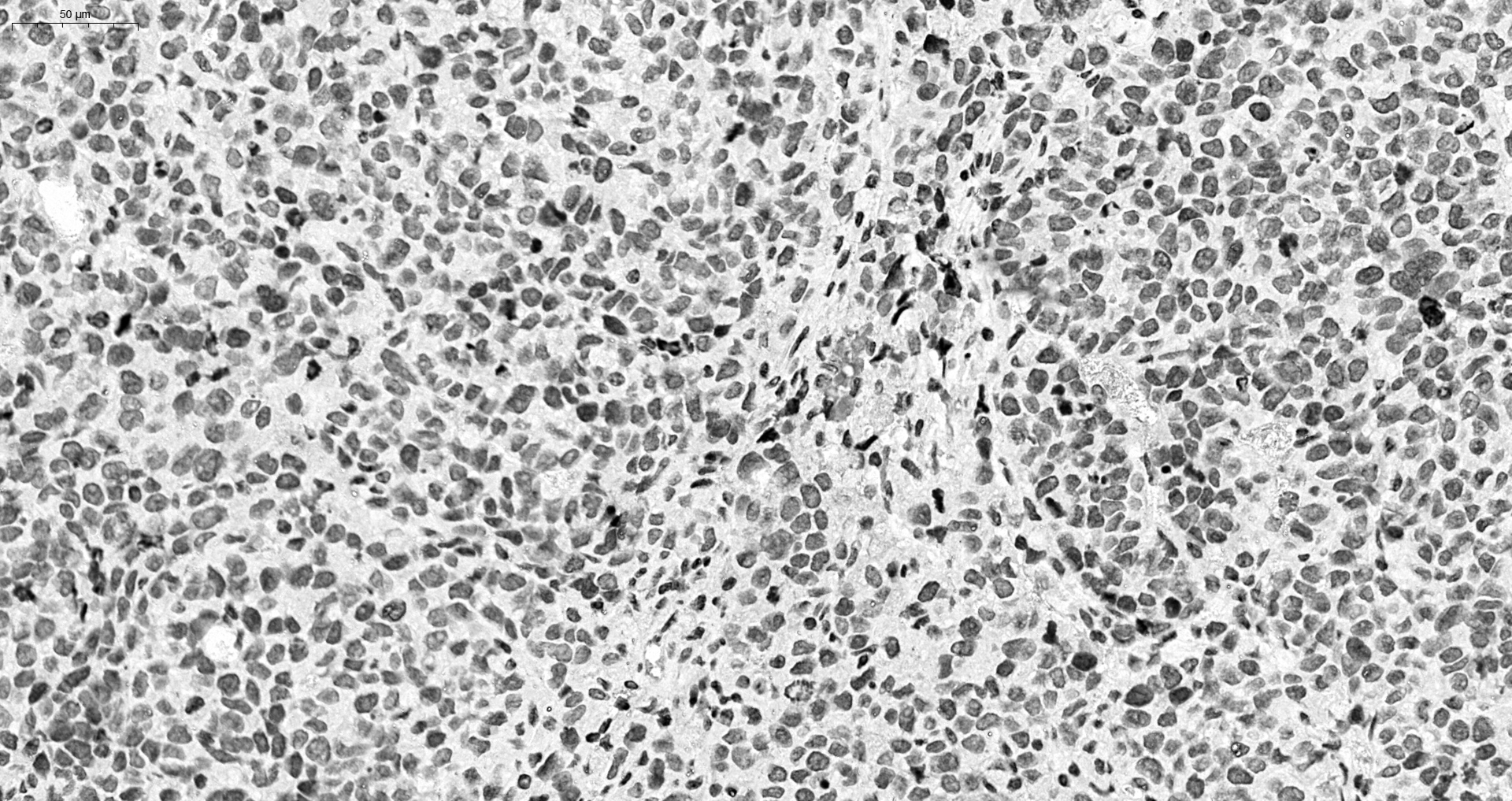

Supplement: Supplementary file 1 — Additional file 1. Raw data. [file 12935_2023_3076_MOESM1_ESM.zip › raw_data/IHC/E-cad/ΓæíSaoS-2+si-DIO3OS/3-1 ΓæíSaoS-2+si-DIO3OS.tif]

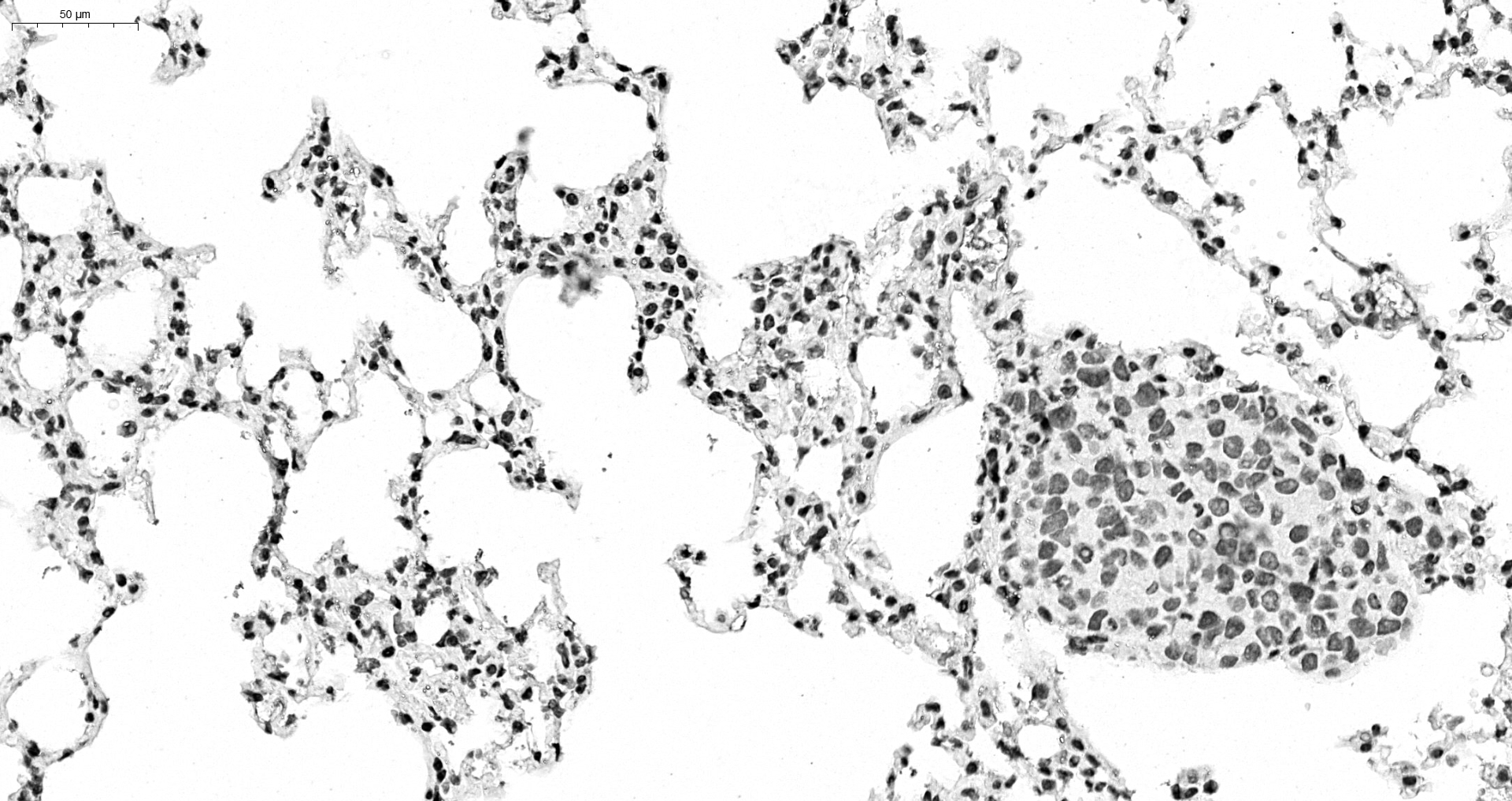

Supplement: Supplementary file 1 — Additional file 1. Raw data. [file 12935_2023_3076_MOESM1_ESM.zip › raw_data/IHC/E-cad/ΓæíSaoS-2+si-DIO3OS/3-3 ΓæíSaoS-2+si-DIO3OS.tif]

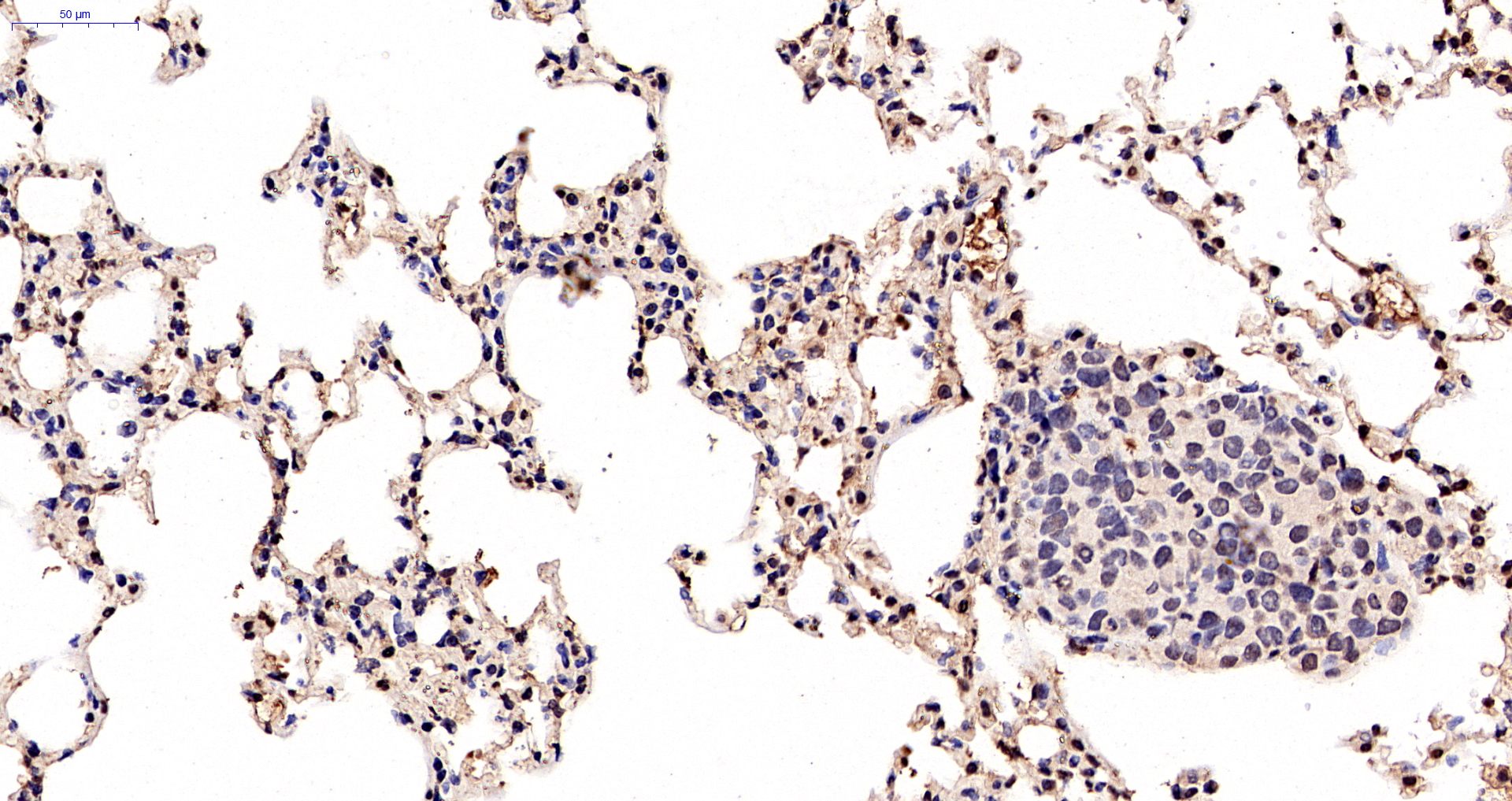

Supplement: Supplementary file 1 — Additional file 1. Raw data. [file 12935_2023_3076_MOESM1_ESM.zip › raw_data/IHC/E-cad/ΓæíSaoS-2+si-DIO3OS/3-3 ΓæíSaoS-2+si-DIO3OS.jpg]

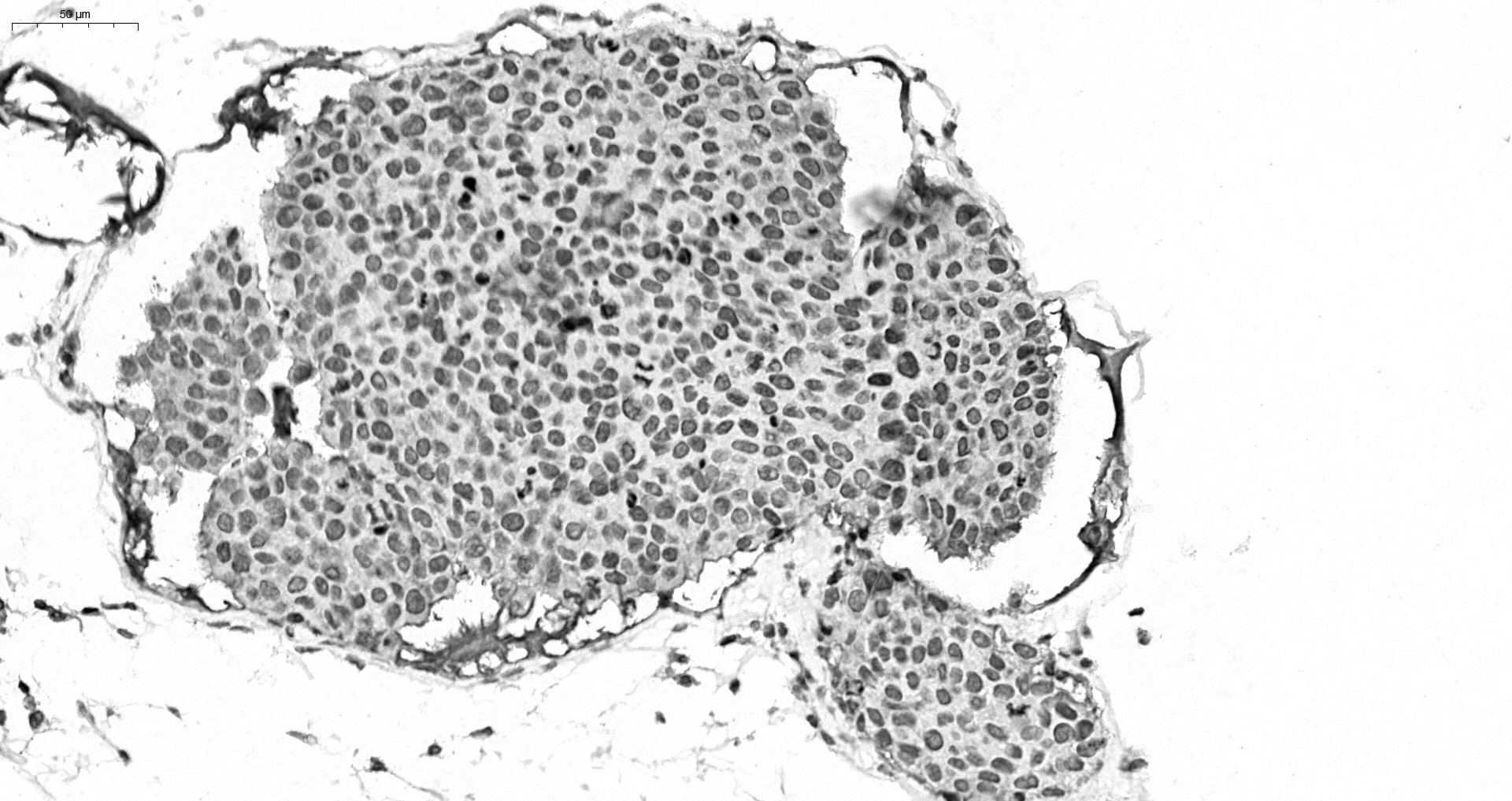

Supplement: Supplementary file 1 — Additional file 1. Raw data. [file 12935_2023_3076_MOESM1_ESM.zip › raw_data/IHC/E-cad/ΓæíSaoS-2+si-DIO3OS/1-2 ΓæíSaoS-2+si-DIO3OS.tif]

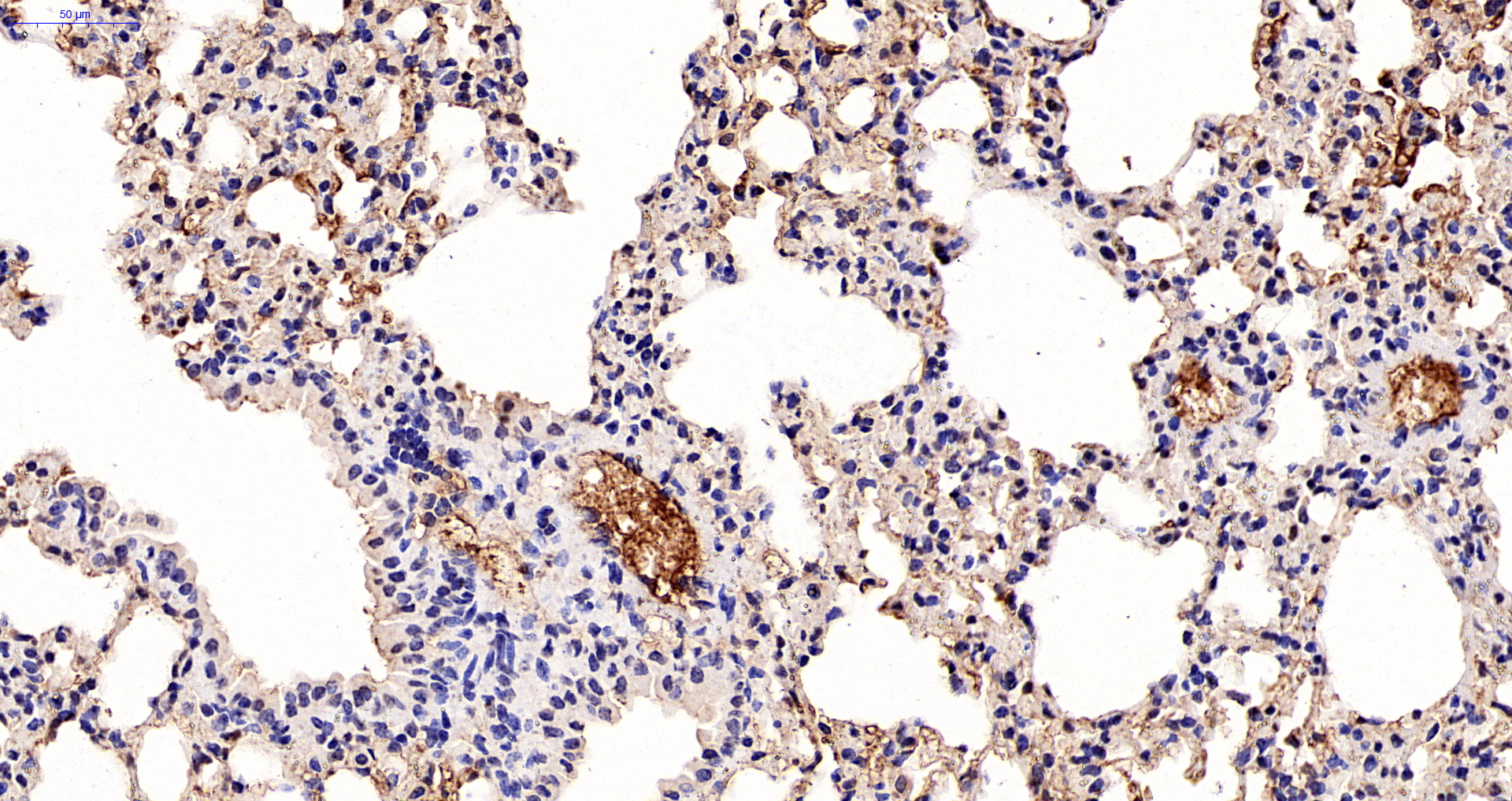

Supplement: Supplementary file 1 — Additional file 1. Raw data. [file 12935_2023_3076_MOESM1_ESM.zip › raw_data/IHC/E-cad/ΓæíSaoS-2+si-DIO3OS/2-2 ΓæíSaoS-2+si-DIO3OS.jpg]

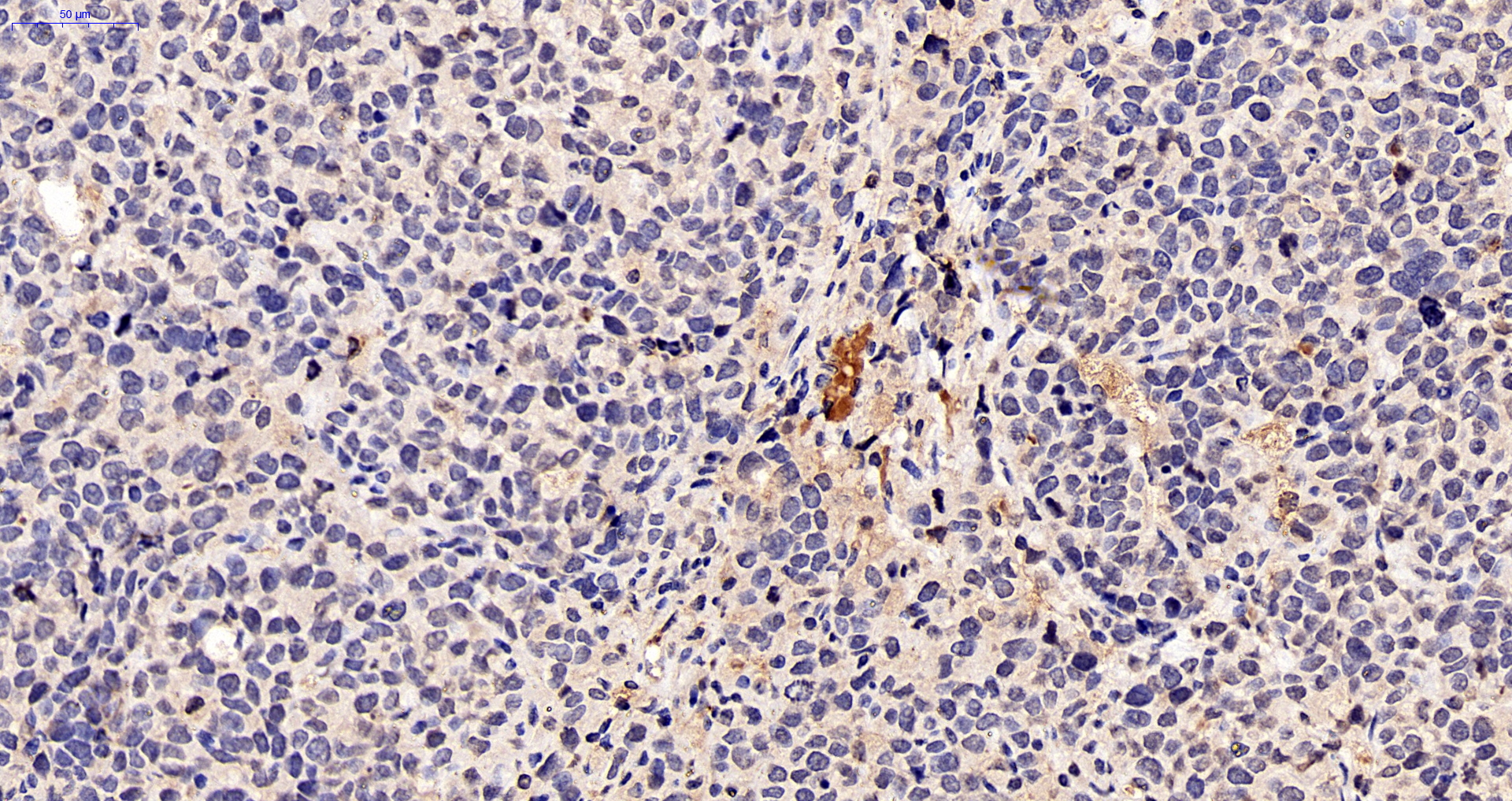

Supplement: Supplementary file 1 — Additional file 1. Raw data. [file 12935_2023_3076_MOESM1_ESM.zip › raw_data/IHC/E-cad/ΓæíSaoS-2+si-DIO3OS/3-1 ΓæíSaoS-2+si-DIO3OS.jpg]

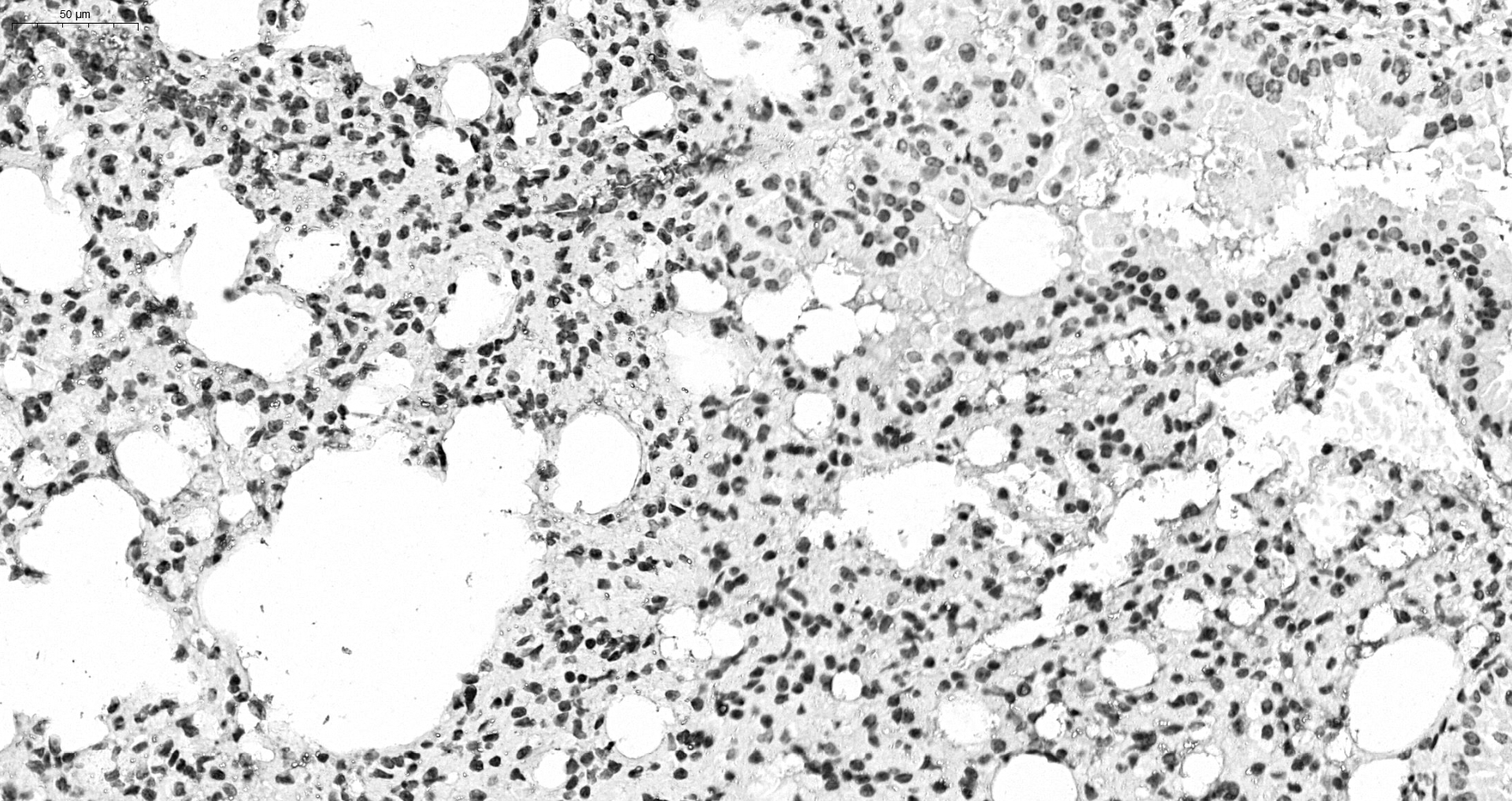

Supplement: Supplementary file 1 — Additional file 1. Raw data. [file 12935_2023_3076_MOESM1_ESM.zip › raw_data/IHC/E-cad/ΓæíSaoS-2+si-DIO3OS/2-3 ΓæíSaoS-2+si-DIO3OS.tif]

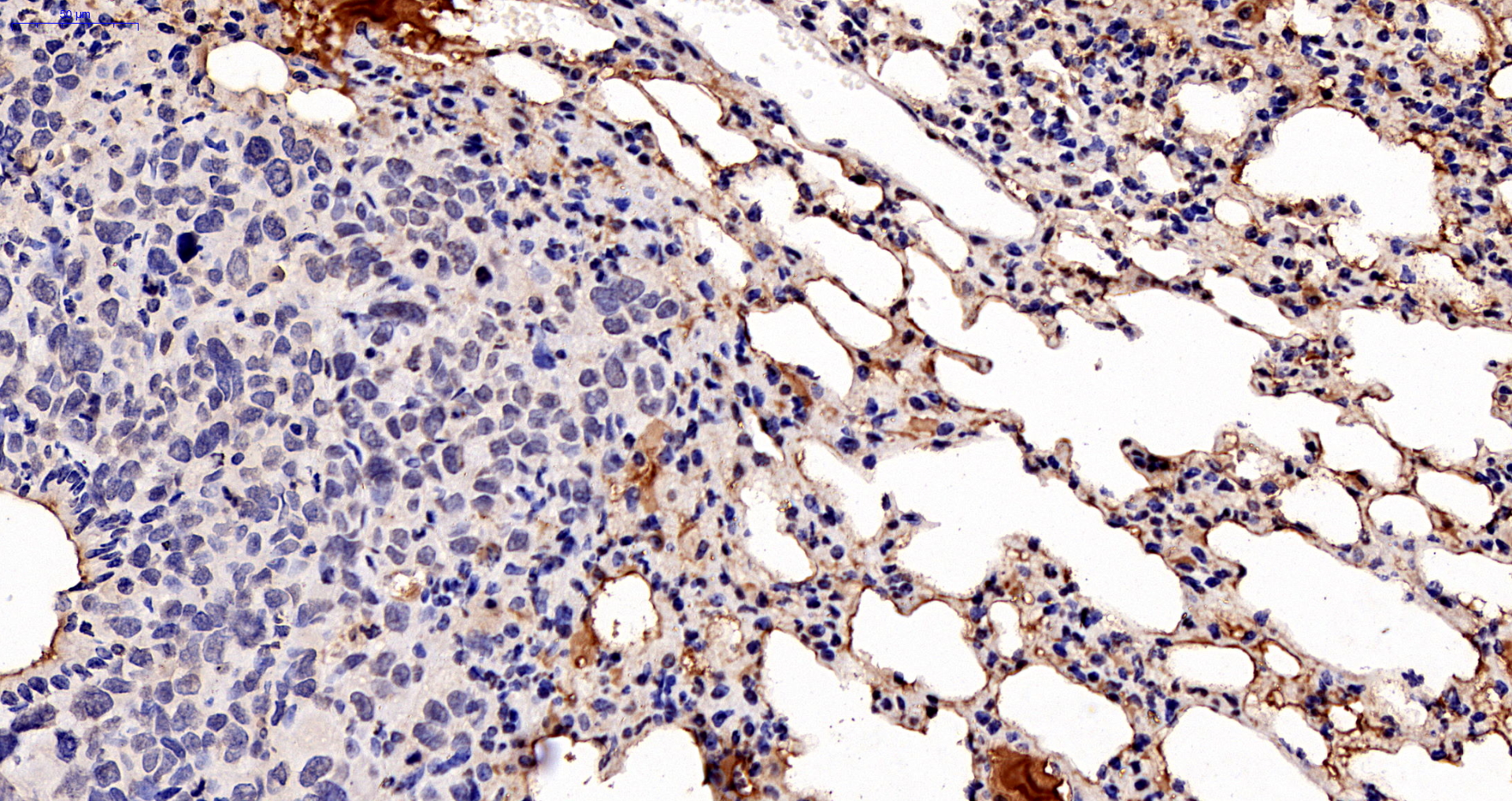

Supplement: Supplementary file 1 — Additional file 1. Raw data. [file 12935_2023_3076_MOESM1_ESM.zip › raw_data/IHC/E-cad/ΓæíSaoS-2+si-DIO3OS/1-3 ΓæíSaoS-2+si-DIO3OS.jpg]

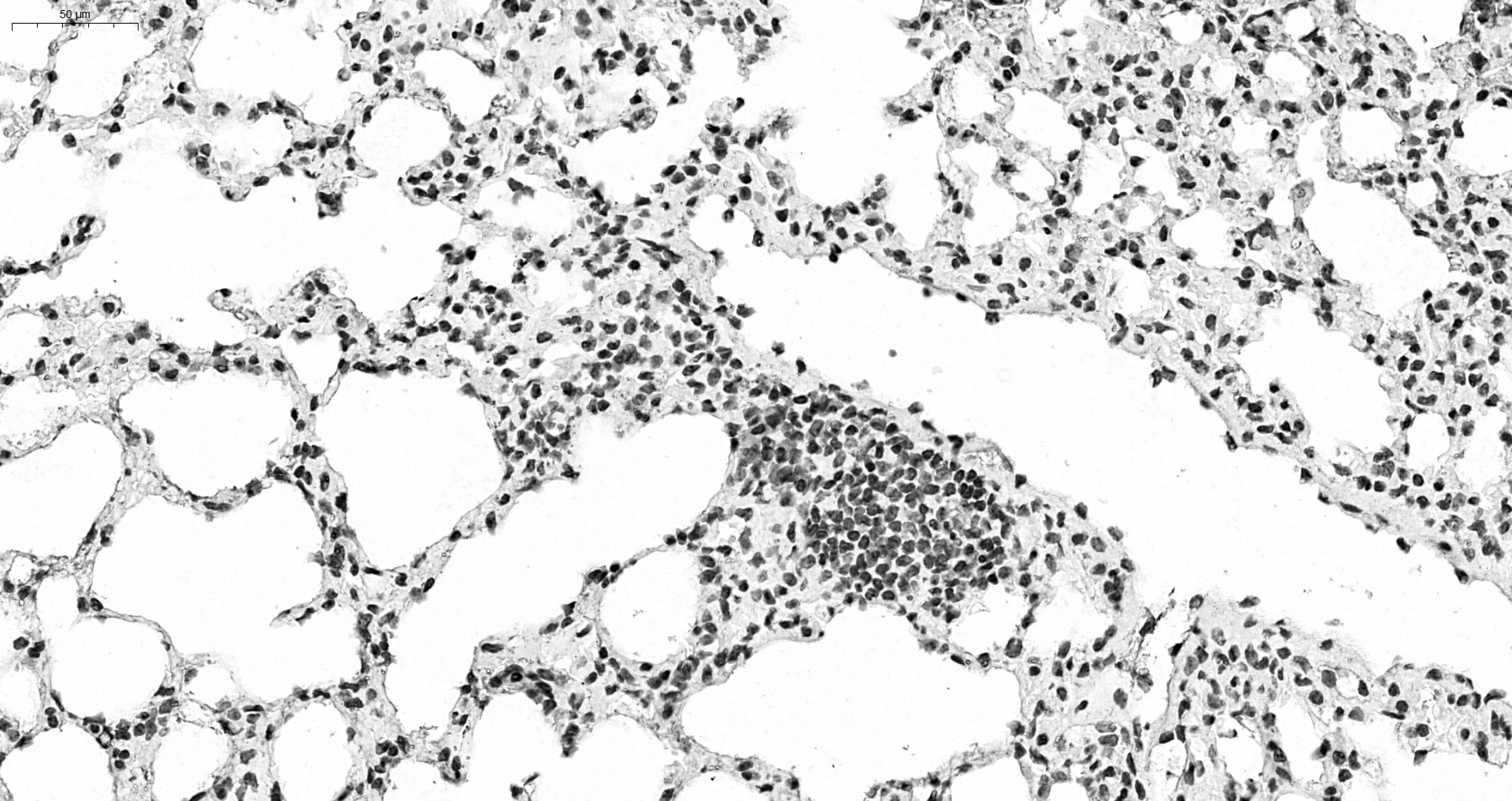

Supplement: Supplementary file 1 — Additional file 1. Raw data. [file 12935_2023_3076_MOESM1_ESM.zip › raw_data/IHC/E-cad/ΓæíSaoS-2+si-DIO3OS/2-1 ΓæíSaoS-2+si-DIO3OS.tif]

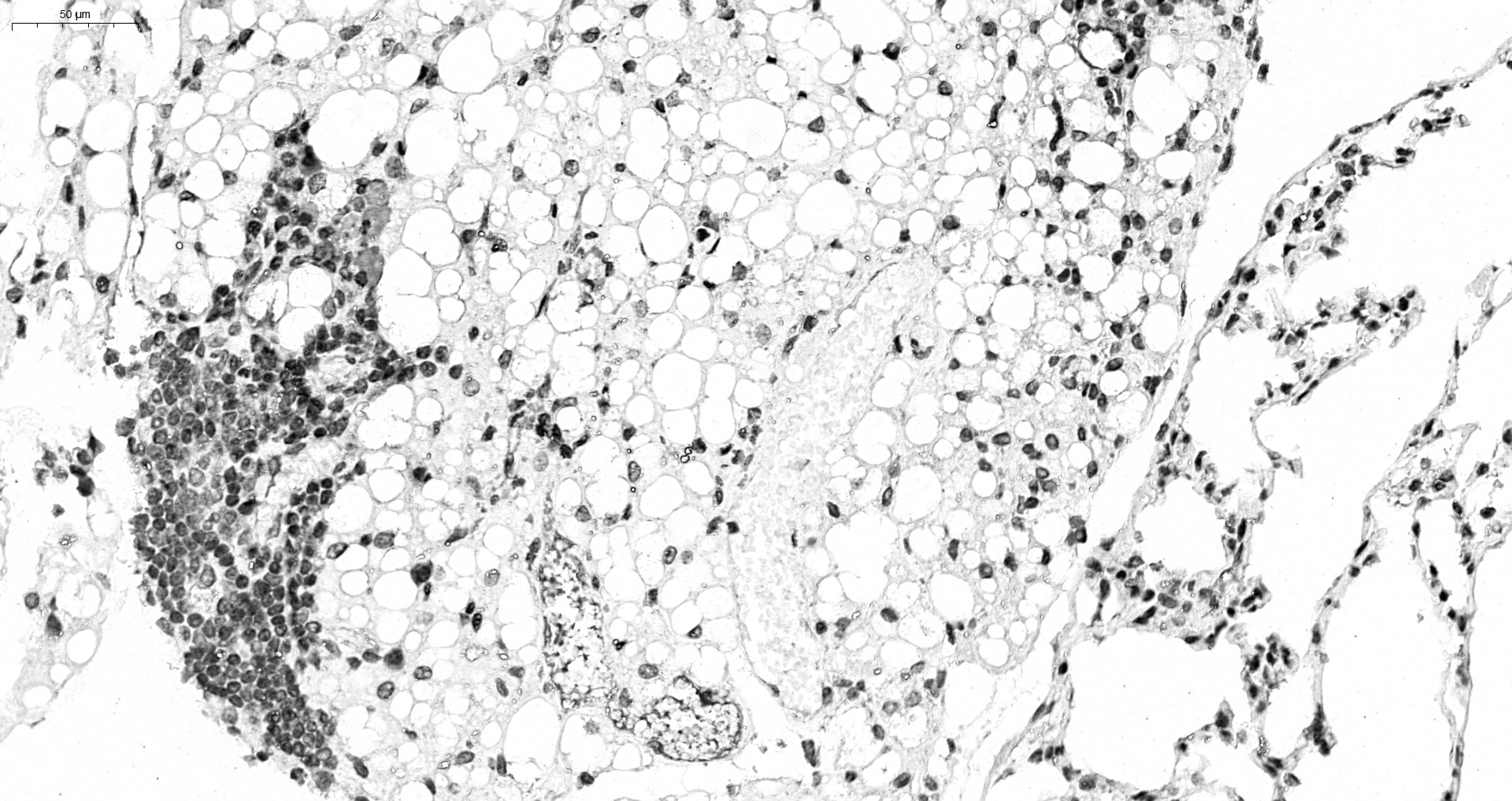

Supplement: Supplementary file 1 — Additional file 1. Raw data. [file 12935_2023_3076_MOESM1_ESM.zip › raw_data/IHC/E-cad/ΓæíSaoS-2+si-DIO3OS/3-2 ΓæíSaoS-2+si-DIO3OS.tif]

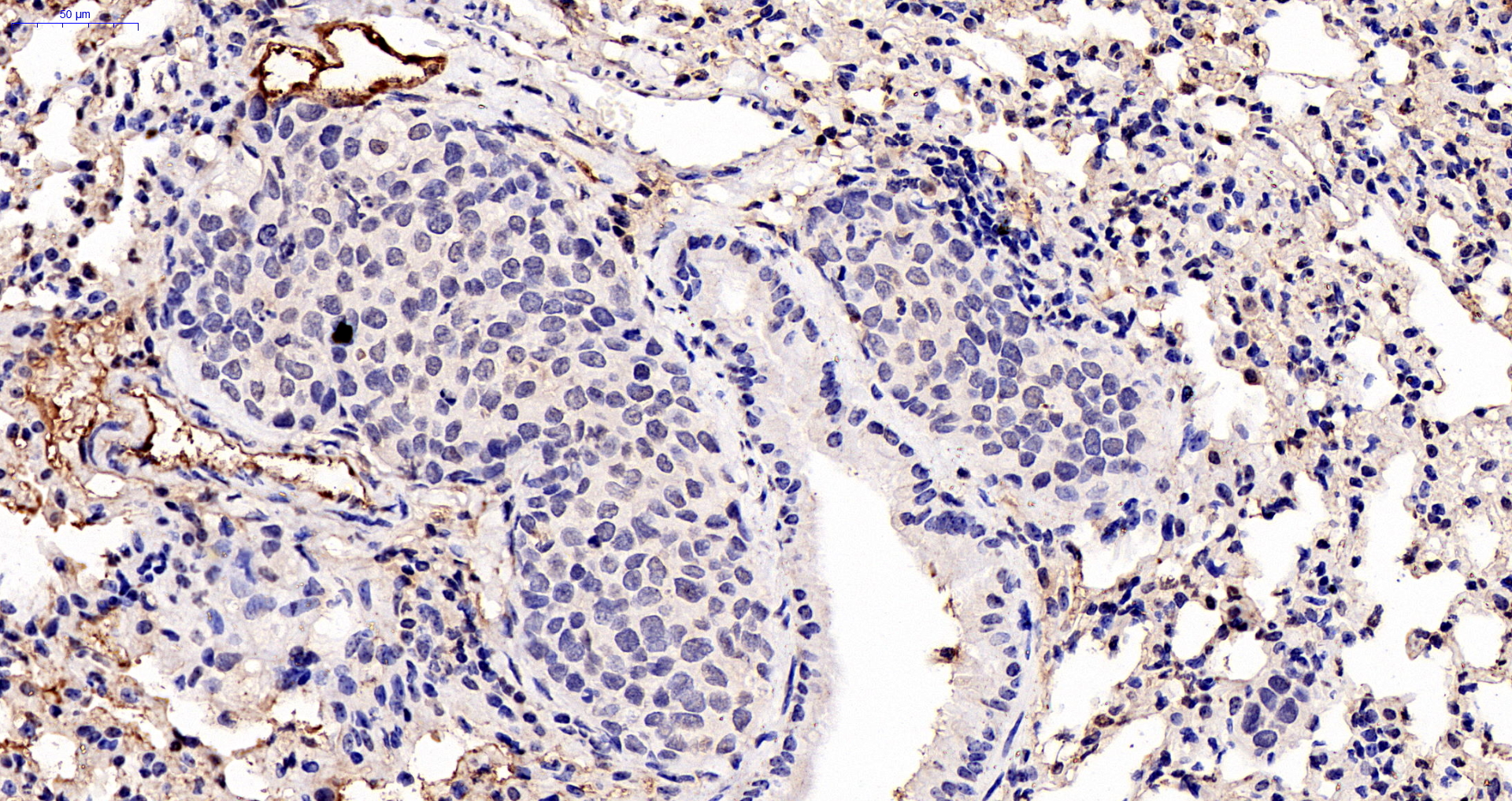

Supplement: Supplementary file 1 — Additional file 1. Raw data. [file 12935_2023_3076_MOESM1_ESM.zip › raw_data/IHC/E-cad/ΓæíSaoS-2+si-DIO3OS/1-1 ΓæíSaoS-2+si-DIO3OS.jpg]

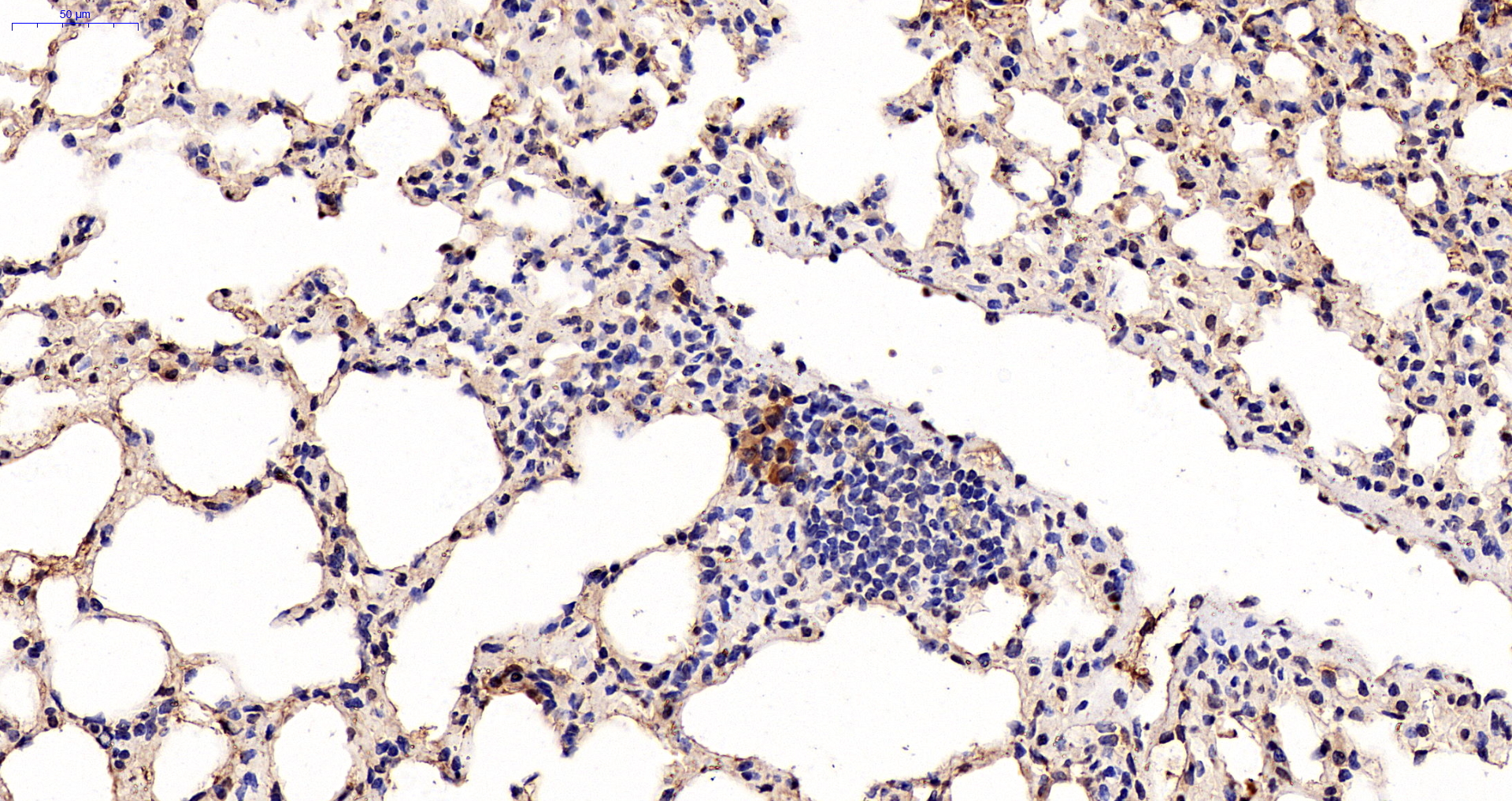

Supplement: Supplementary file 1 — Additional file 1. Raw data. [file 12935_2023_3076_MOESM1_ESM.zip › raw_data/IHC/E-cad/ΓæíSaoS-2+si-DIO3OS/2-1 ΓæíSaoS-2+si-DIO3OS.jpg]

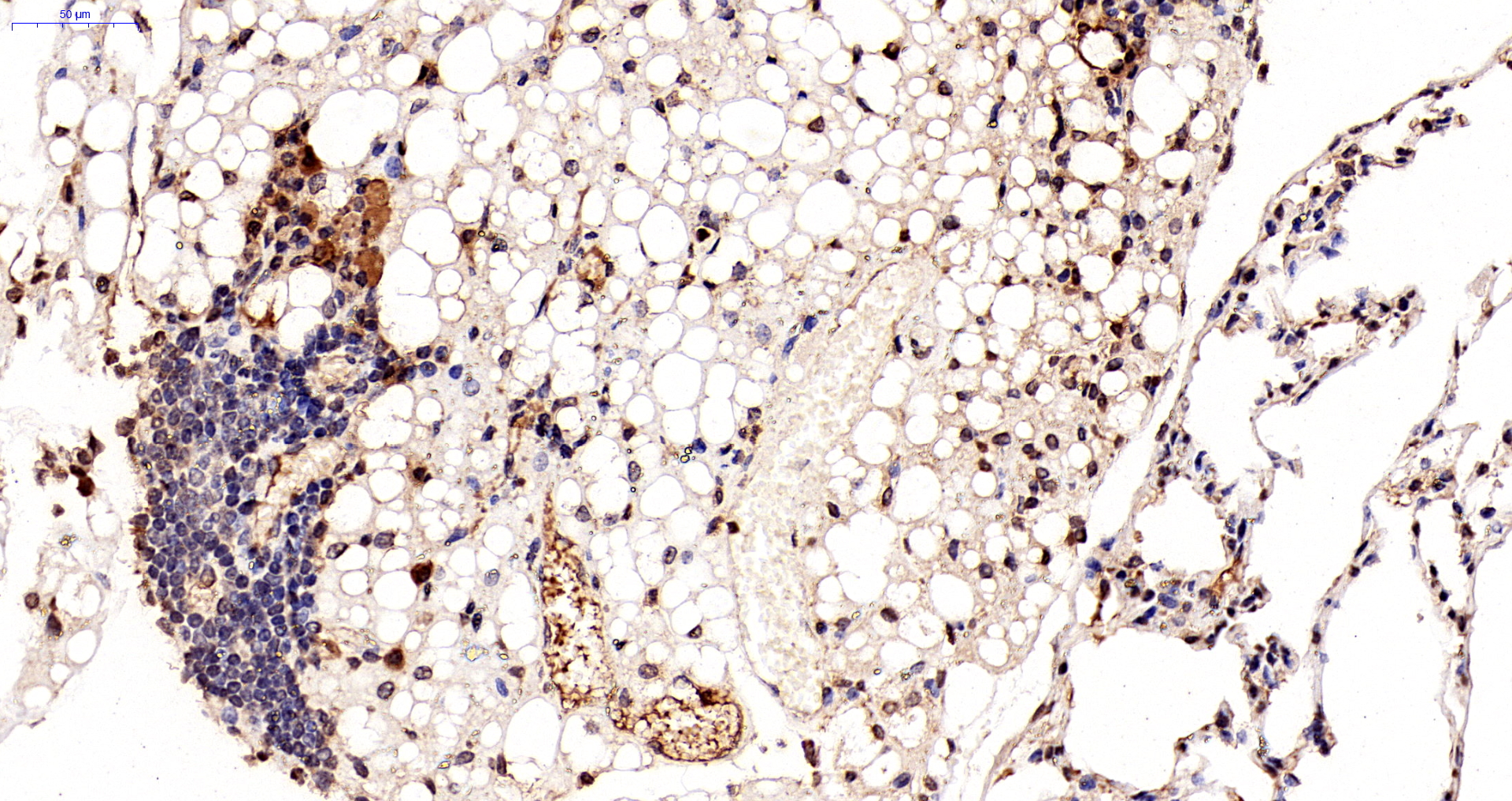

Supplement: Supplementary file 1 — Additional file 1. Raw data. [file 12935_2023_3076_MOESM1_ESM.zip › raw_data/IHC/E-cad/ΓæíSaoS-2+si-DIO3OS/3-2 ΓæíSaoS-2+si-DIO3OS.jpg]

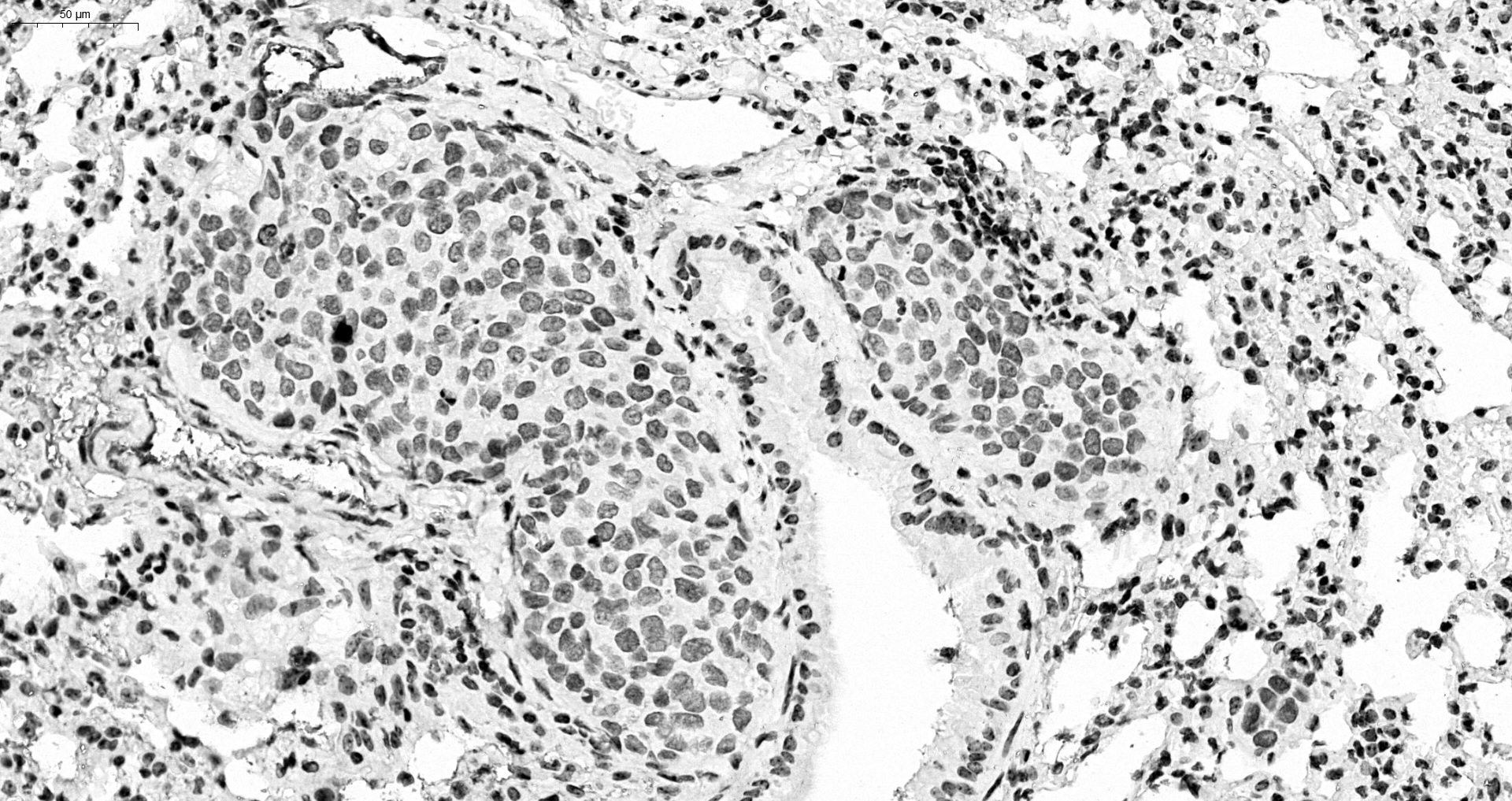

Supplement: Supplementary file 1 — Additional file 1. Raw data. [file 12935_2023_3076_MOESM1_ESM.zip › raw_data/IHC/E-cad/ΓæíSaoS-2+si-DIO3OS/1-1 ΓæíSaoS-2+si-DIO3OS.tif]

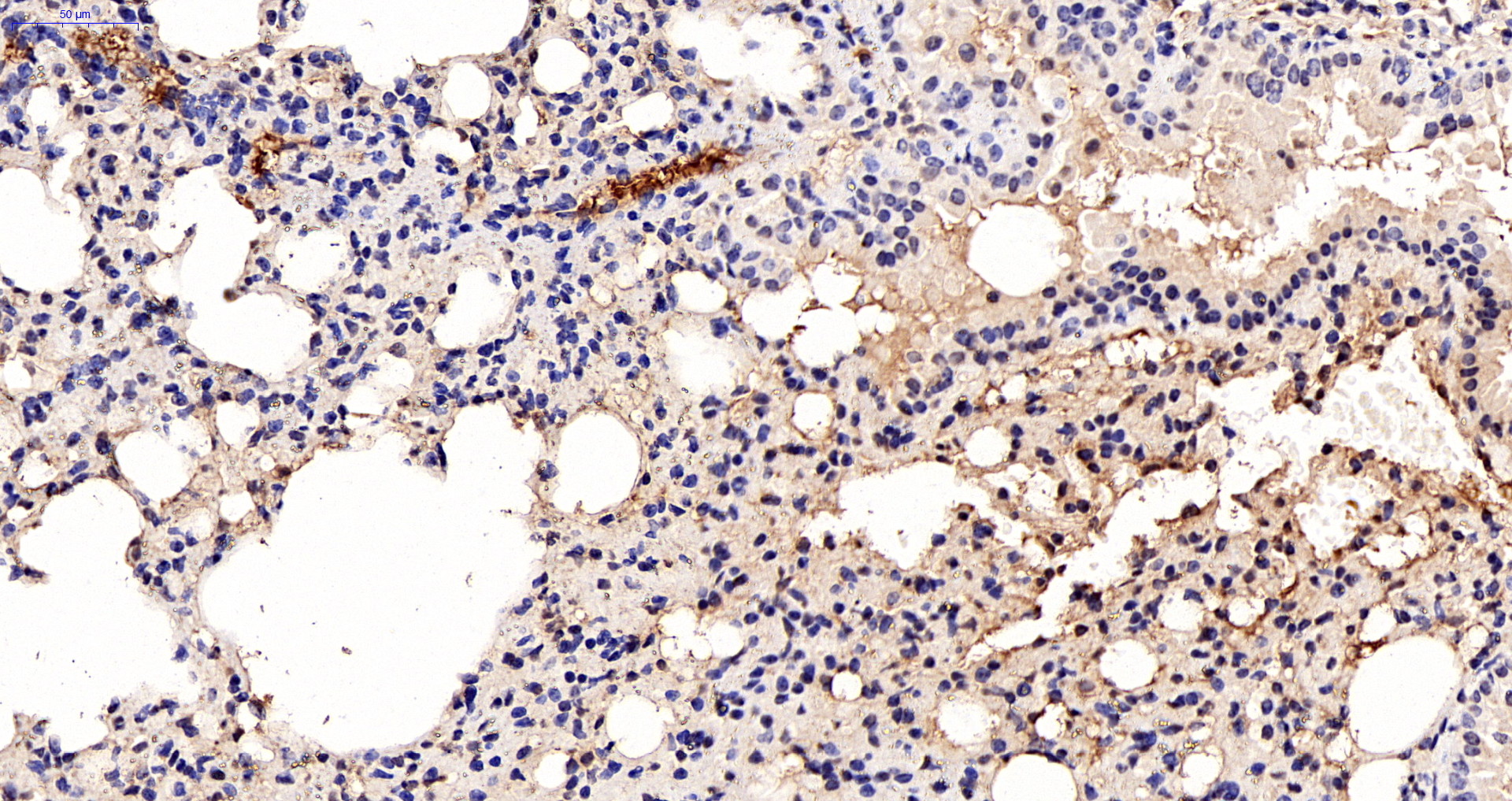

Supplement: Supplementary file 1 — Additional file 1. Raw data. [file 12935_2023_3076_MOESM1_ESM.zip › raw_data/IHC/E-cad/ΓæíSaoS-2+si-DIO3OS/2-3 ΓæíSaoS-2+si-DIO3OS.jpg]

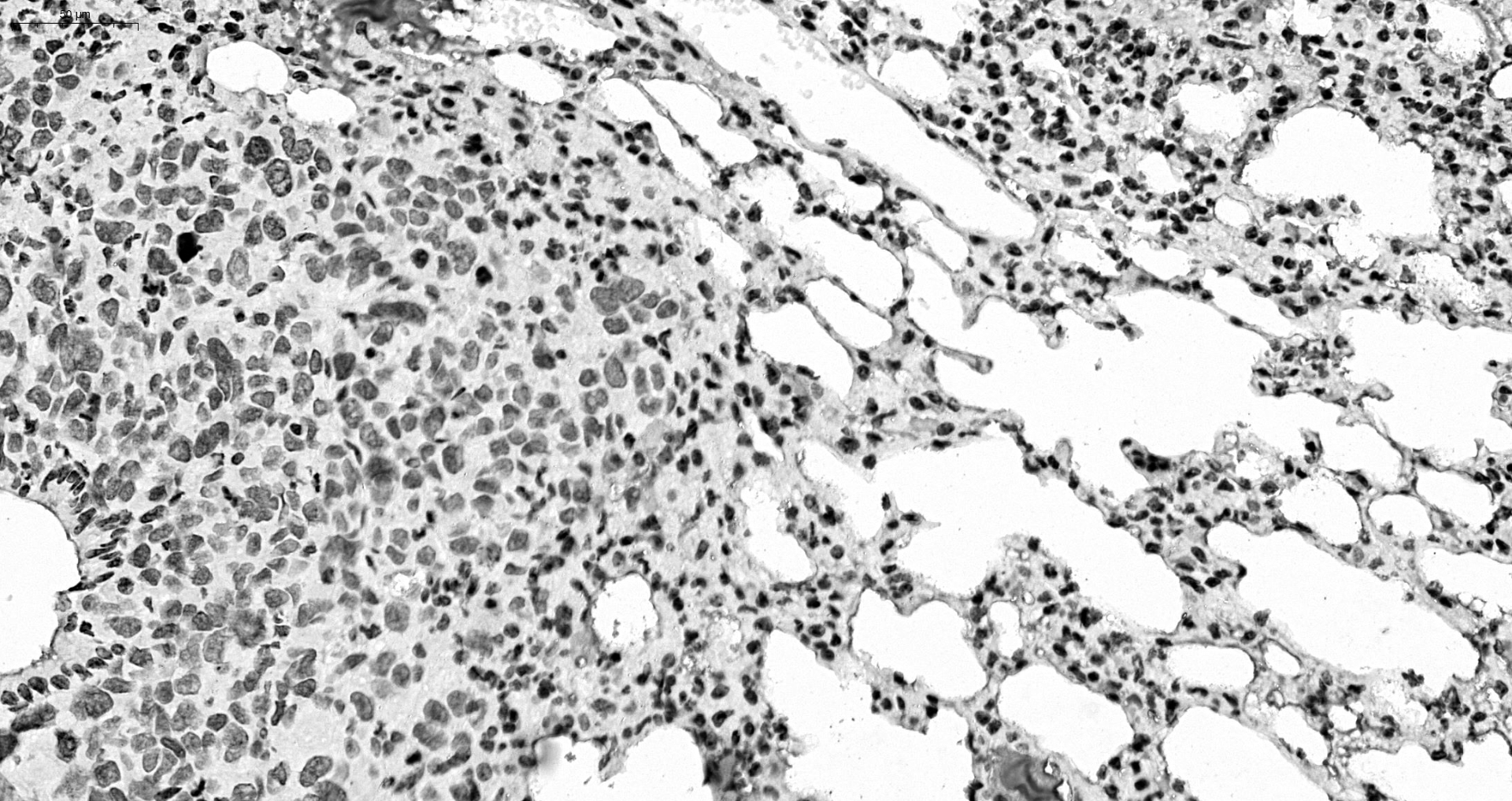

Supplement: Supplementary file 1 — Additional file 1. Raw data. [file 12935_2023_3076_MOESM1_ESM.zip › raw_data/IHC/E-cad/ΓæíSaoS-2+si-DIO3OS/1-3 ΓæíSaoS-2+si-DIO3OS.tif]

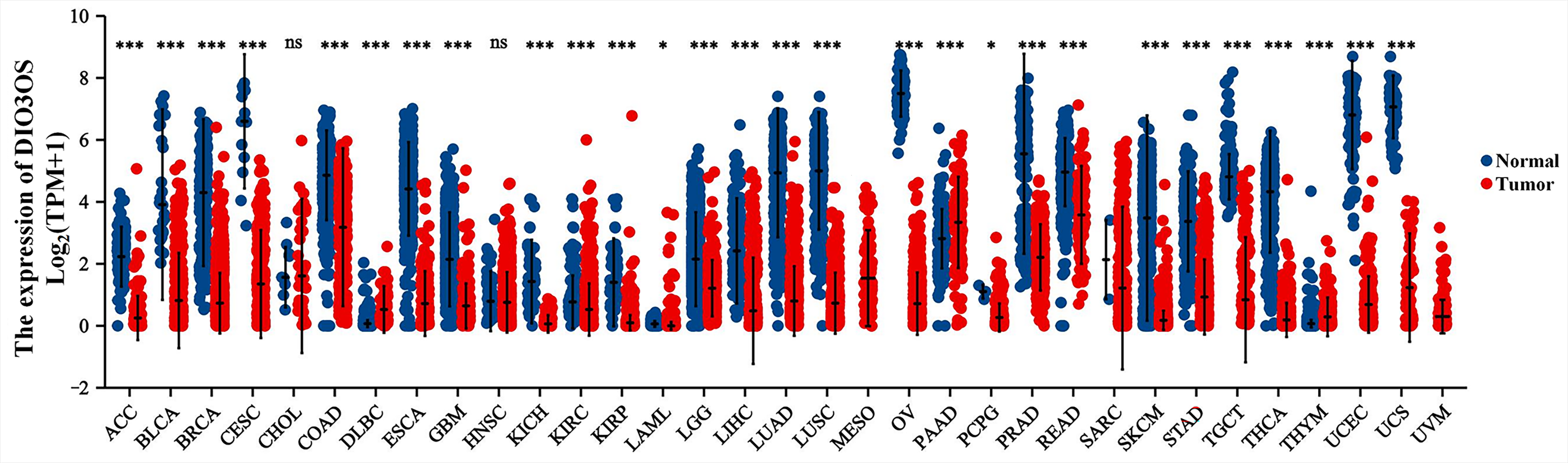

Supplement: Supplementary file 2 — Additional file 2: Figure S1. Differential expression of DIO3OS in pan-cancer. [file 12935_2023_3076_MOESM2_ESM.tif]
