# Supplementary figures and images for: Long intergenic non-coding RNA DIO3OS promotes osteosarcoma metastasis via activation of the TGF-β signaling pathway: a potential diagnostic and immunotherapeutic target for osteosarcoma (part 1 of 2)
Source: Cancer Cell Int. 2023 Sep 26;23:215. doi: 10.1186/s12935-023-03076-5 (PMC10521498; doi:10.1186/s12935-023-03076-5)

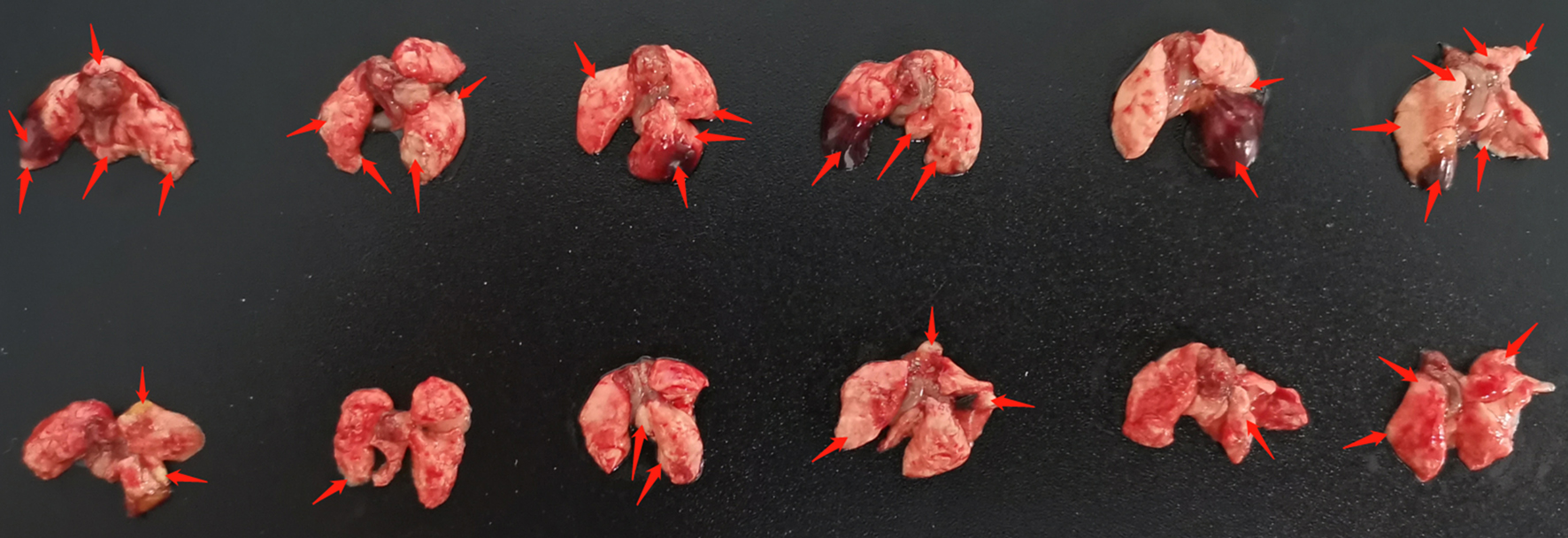

Supplement: Supplementary file 1 — Additional file 1. Raw data. [file 12935_2023_3076_MOESM1_ESM.zip › raw_data/lung marker/lung-1.jpg]

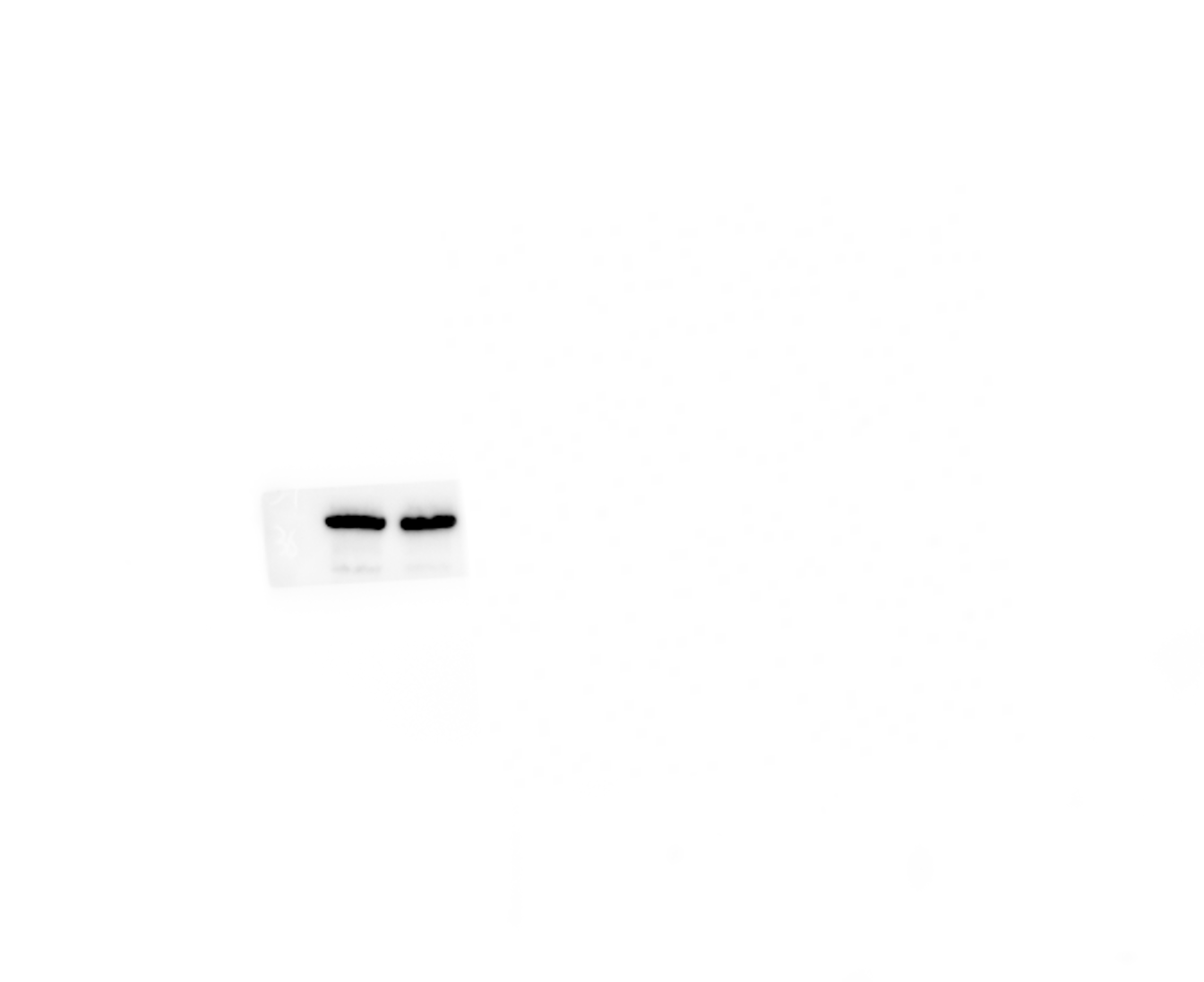

Supplement: Supplementary file 1 — Additional file 1. Raw data. [file 12935_2023_3076_MOESM1_ESM.zip › raw_data/figure7A1/smad2/SMAD2 3∩╝êΓæáSaoS-2+ si-NC∩╝¢ΓæíSaoS-2+ si-DIO3OS∩╝ë.tif]

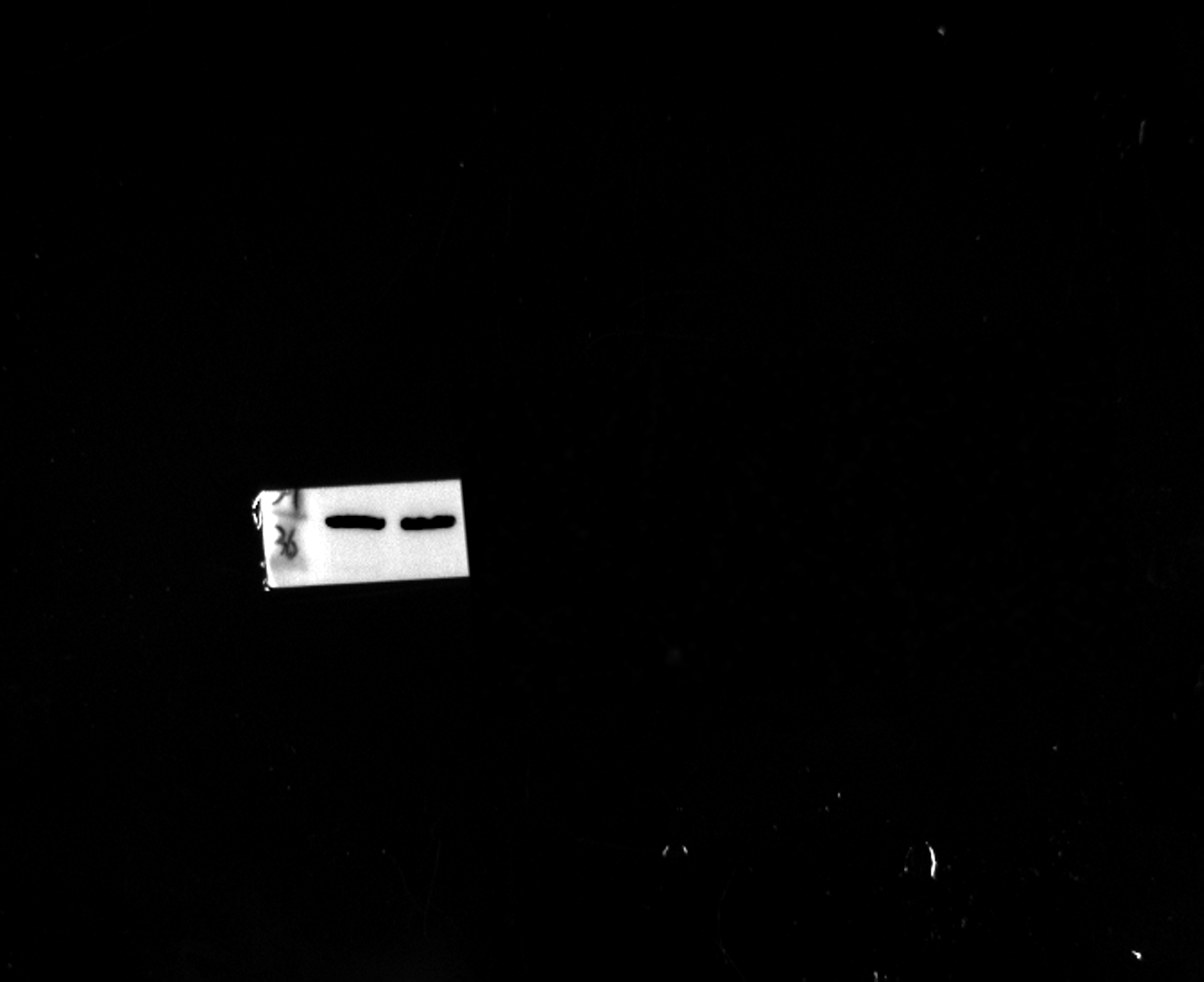

Supplement: Supplementary file 1 — Additional file 1. Raw data. [file 12935_2023_3076_MOESM1_ESM.zip › raw_data/figure7A1/smad2/SMAD2 3τÖ╜σàë∩╝êΓæáSaoS-2+ si-NC∩╝¢ΓæíSaoS-2+ si-DIO3OS∩╝ë.tif.tif]

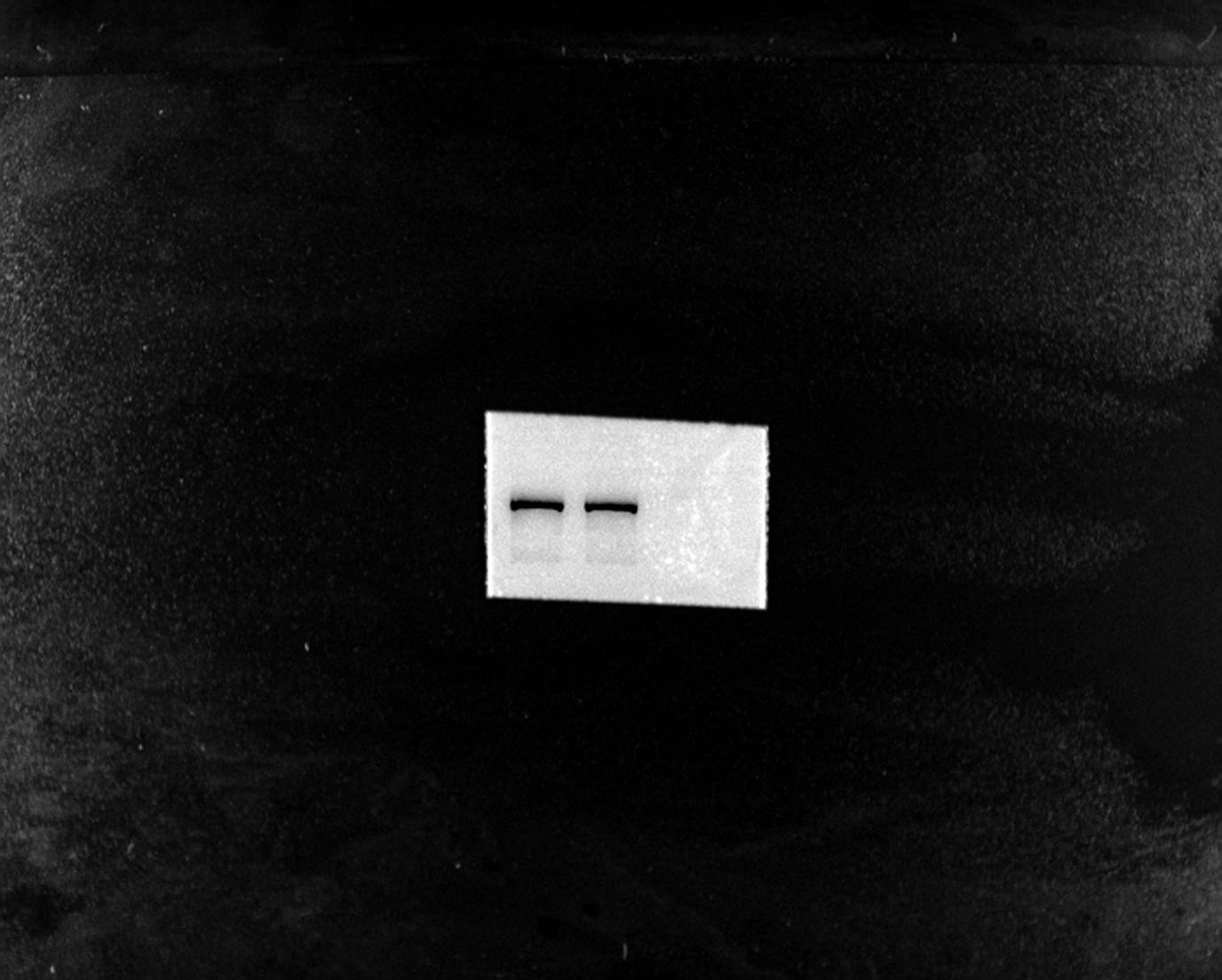

Supplement: Supplementary file 1 — Additional file 1. Raw data. [file 12935_2023_3076_MOESM1_ESM.zip › raw_data/figure7A1/smad2/SMAD2 1τÖ╜σàë∩╝êΓæáSaoS-2+ si-NC∩╝¢ΓæíSaoS-2+ si-DIO3OS∩╝ë.tif]

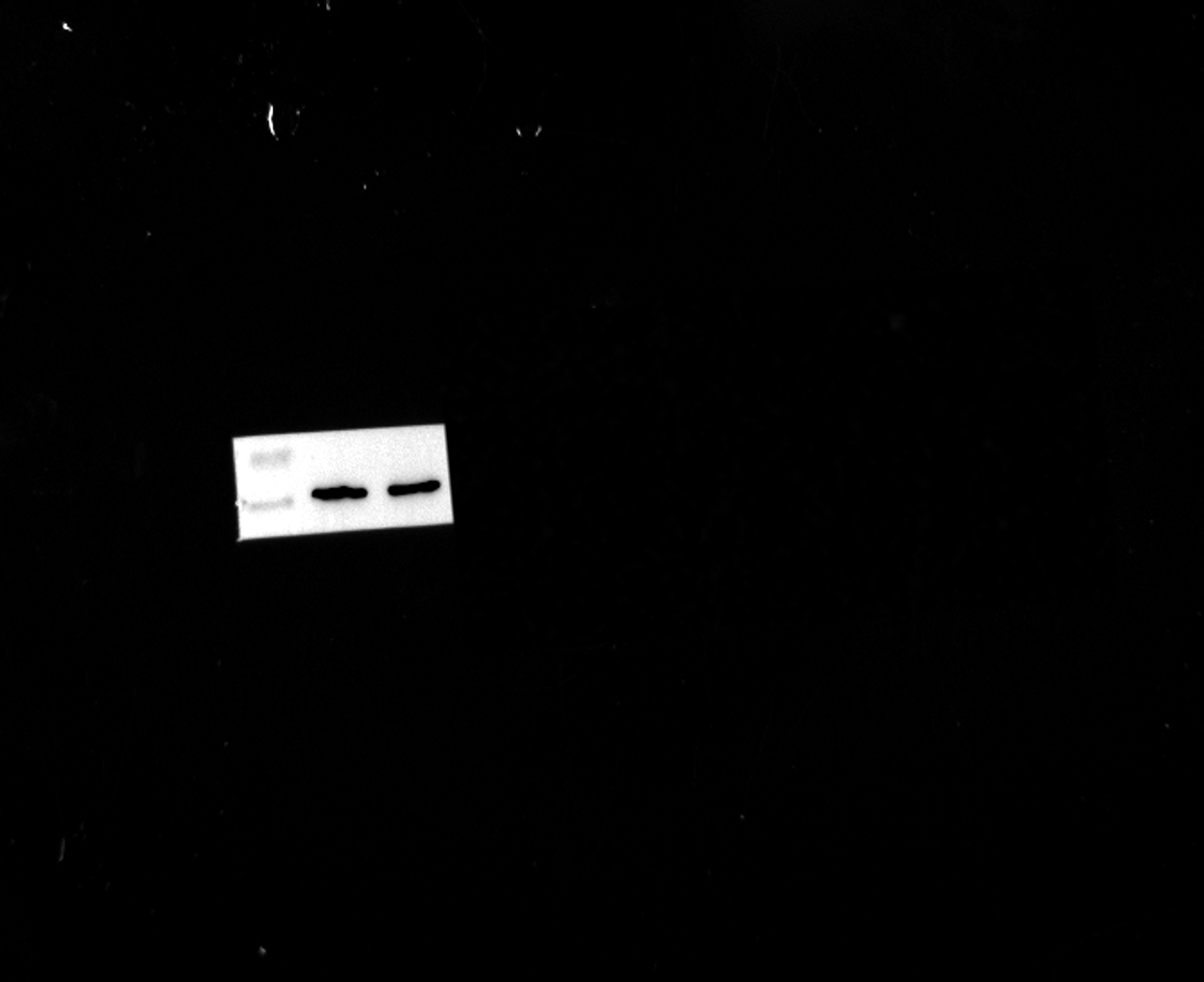

Supplement: Supplementary file 1 — Additional file 1. Raw data. [file 12935_2023_3076_MOESM1_ESM.zip › raw_data/figure7A1/smad2/SMAD2 2τÖ╜σàë∩╝êΓæáSaoS-2+ si-NC∩╝¢ΓæíSaoS-2+ si-DIO3OS∩╝ë.tif.tif]

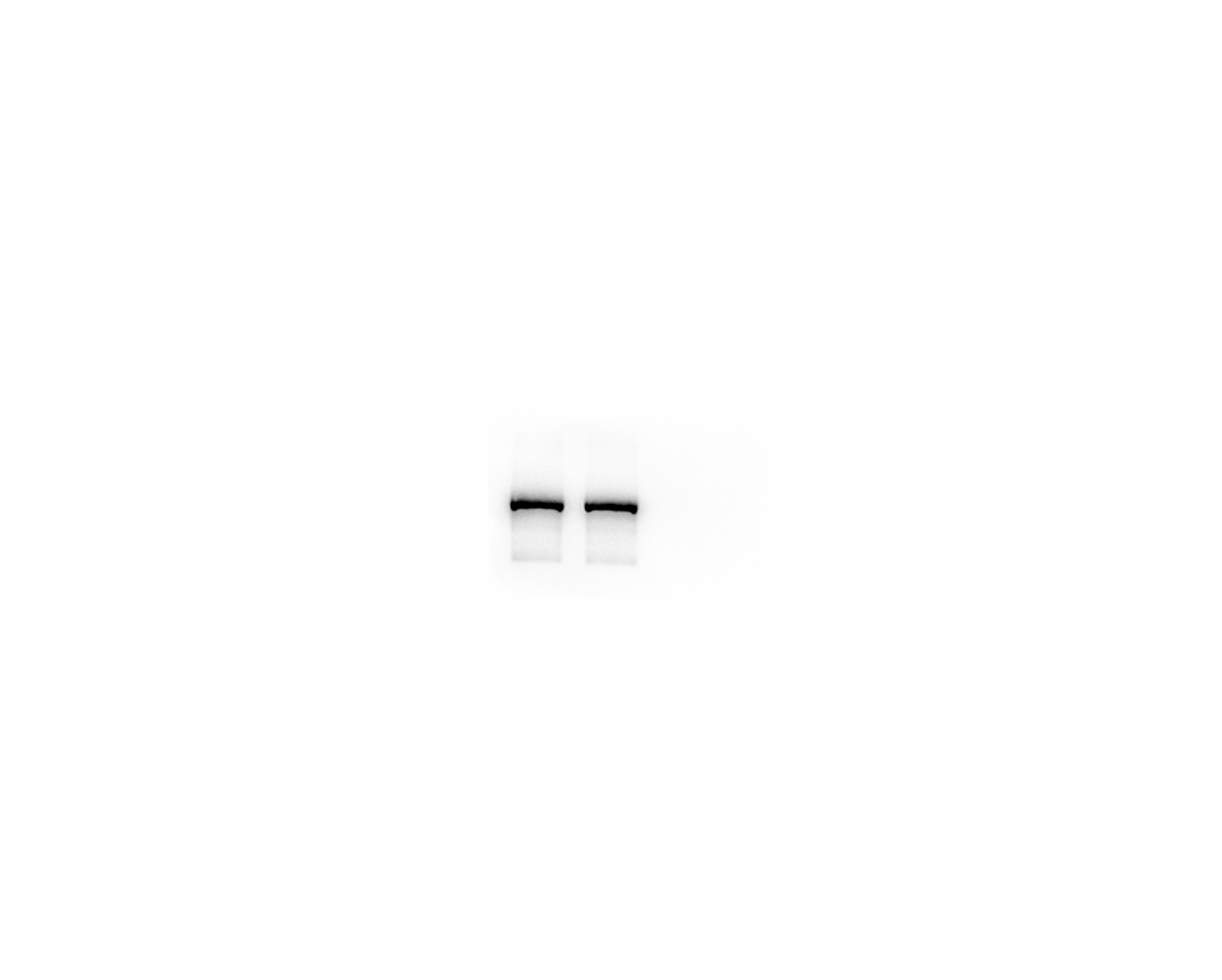

Supplement: Supplementary file 1 — Additional file 1. Raw data. [file 12935_2023_3076_MOESM1_ESM.zip › raw_data/figure7A1/smad2/SMAD2 1(ΓæáSaoS-2+ si-NC∩╝¢ΓæíSaoS-2+ si-DIO3OS∩╝ë.tif]

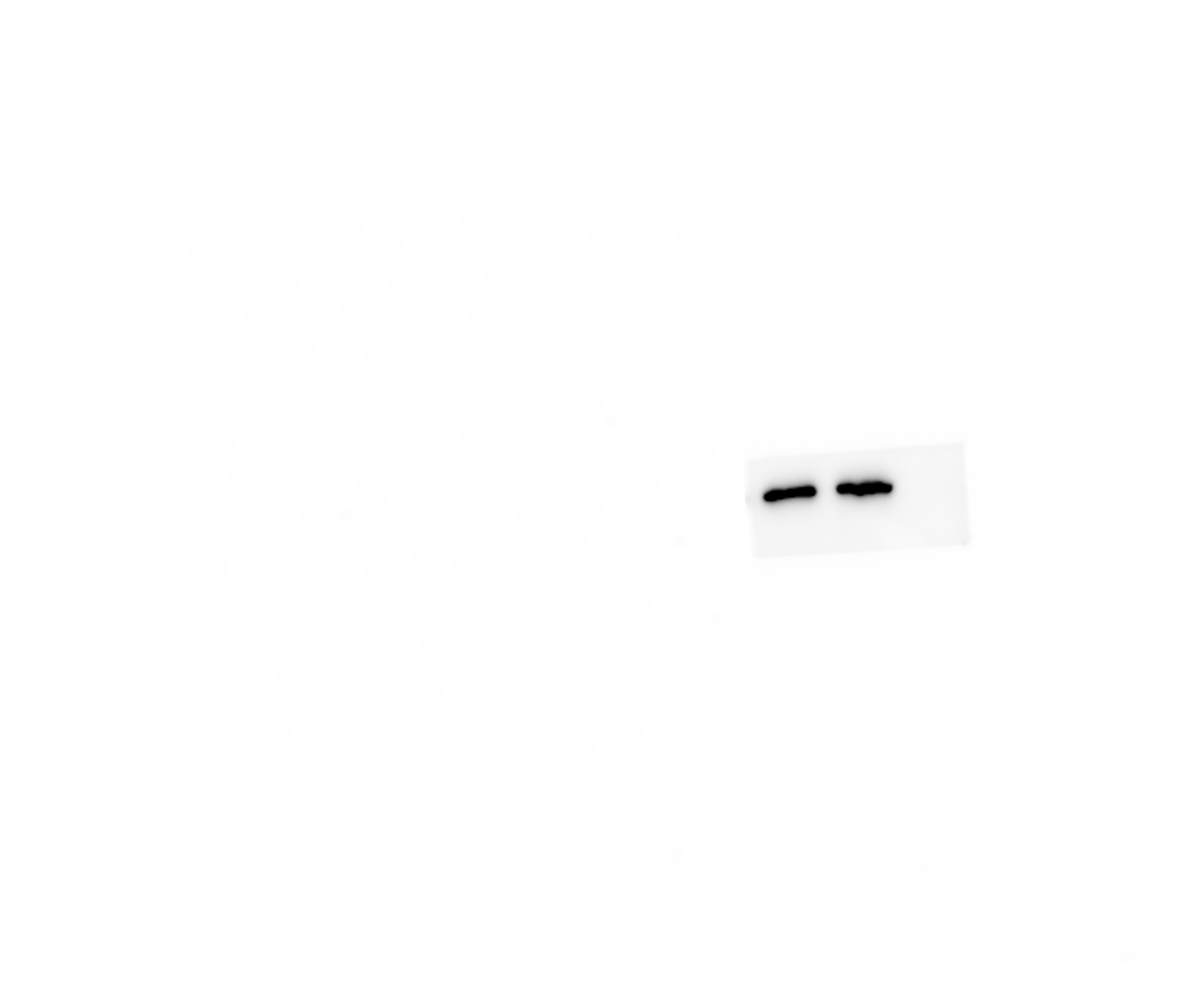

Supplement: Supplementary file 1 — Additional file 1. Raw data. [file 12935_2023_3076_MOESM1_ESM.zip › raw_data/figure7A1/smad2/SMAD2 2∩╝êΓæáSaoS-2+ si-NC∩╝¢ΓæíSaoS-2+ si-DIO3OS∩╝ë.tif.tif]

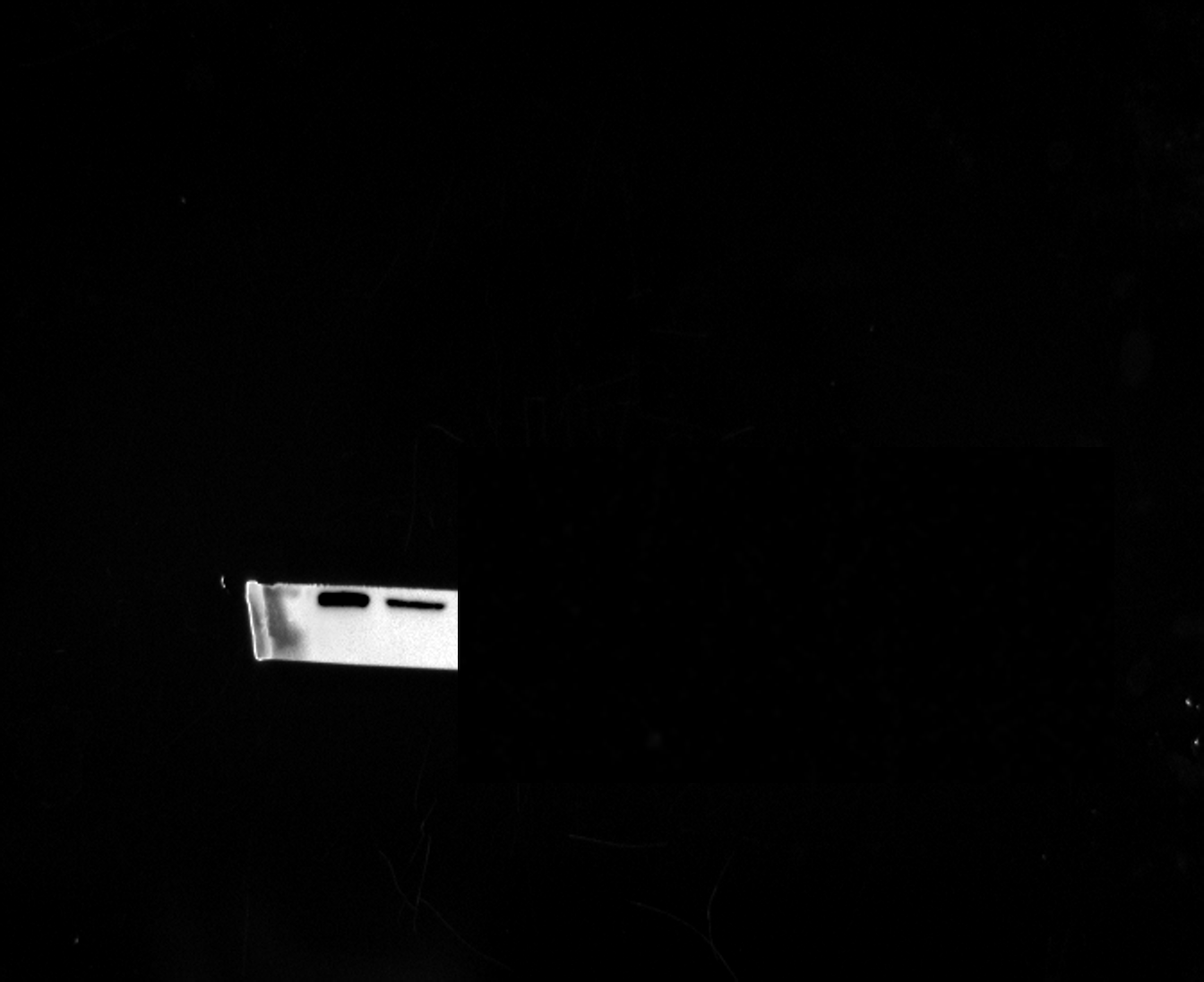

Supplement: Supplementary file 1 — Additional file 1. Raw data. [file 12935_2023_3076_MOESM1_ESM.zip › raw_data/figure7A1/p-smad2/p-SMAD2 3τÖ╜σàë∩╝êΓæáSaoS-2+ si-NC∩╝¢ΓæíSaoS-2+ si-DIO3OS∩╝ë.Tif]

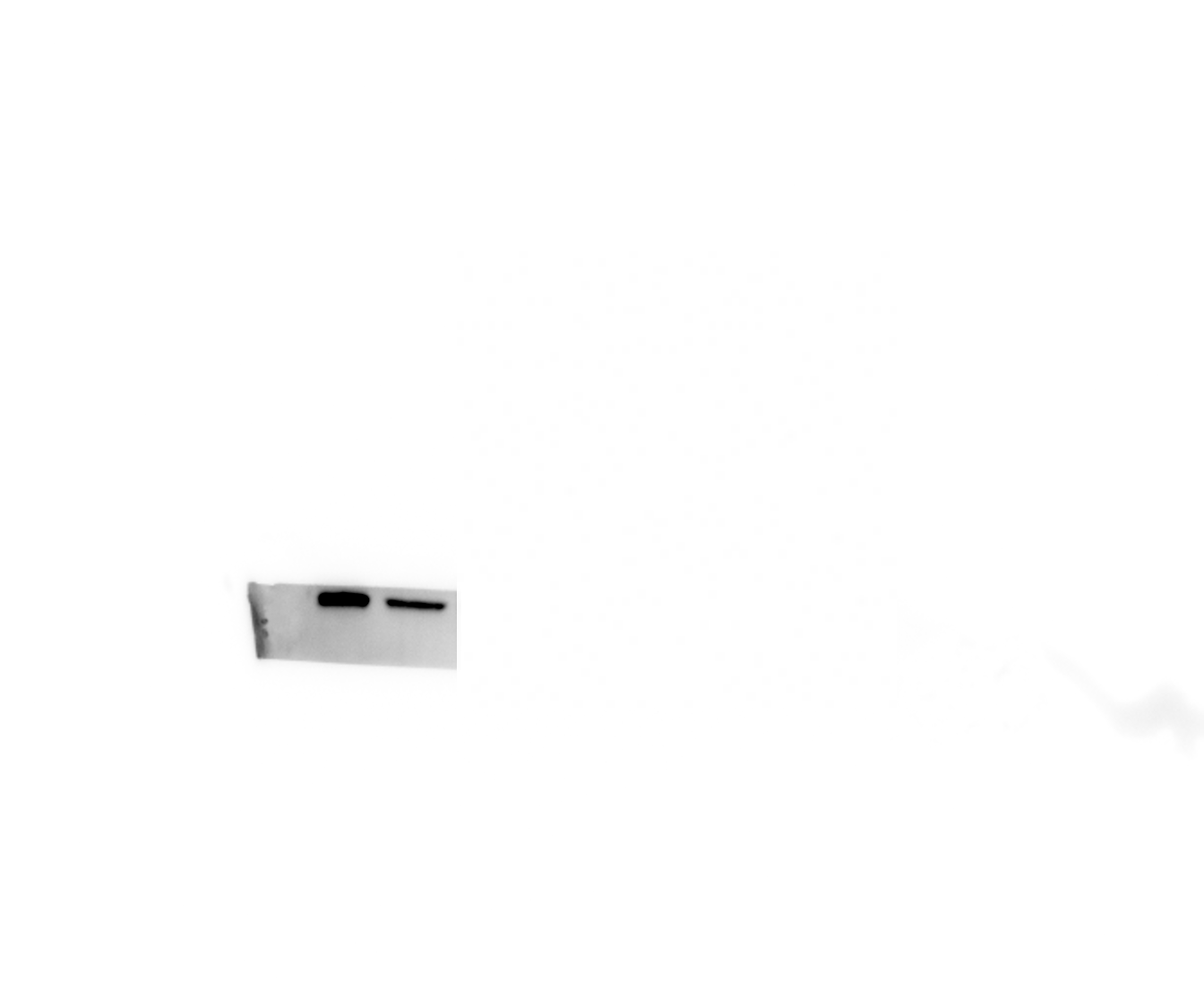

Supplement: Supplementary file 1 — Additional file 1. Raw data. [file 12935_2023_3076_MOESM1_ESM.zip › raw_data/figure7A1/p-smad2/p-SMAD2 3∩╝êΓæáSaoS-2+ si-NC∩╝¢ΓæíSaoS-2+ si-DIO3OS∩╝ë.Tif]

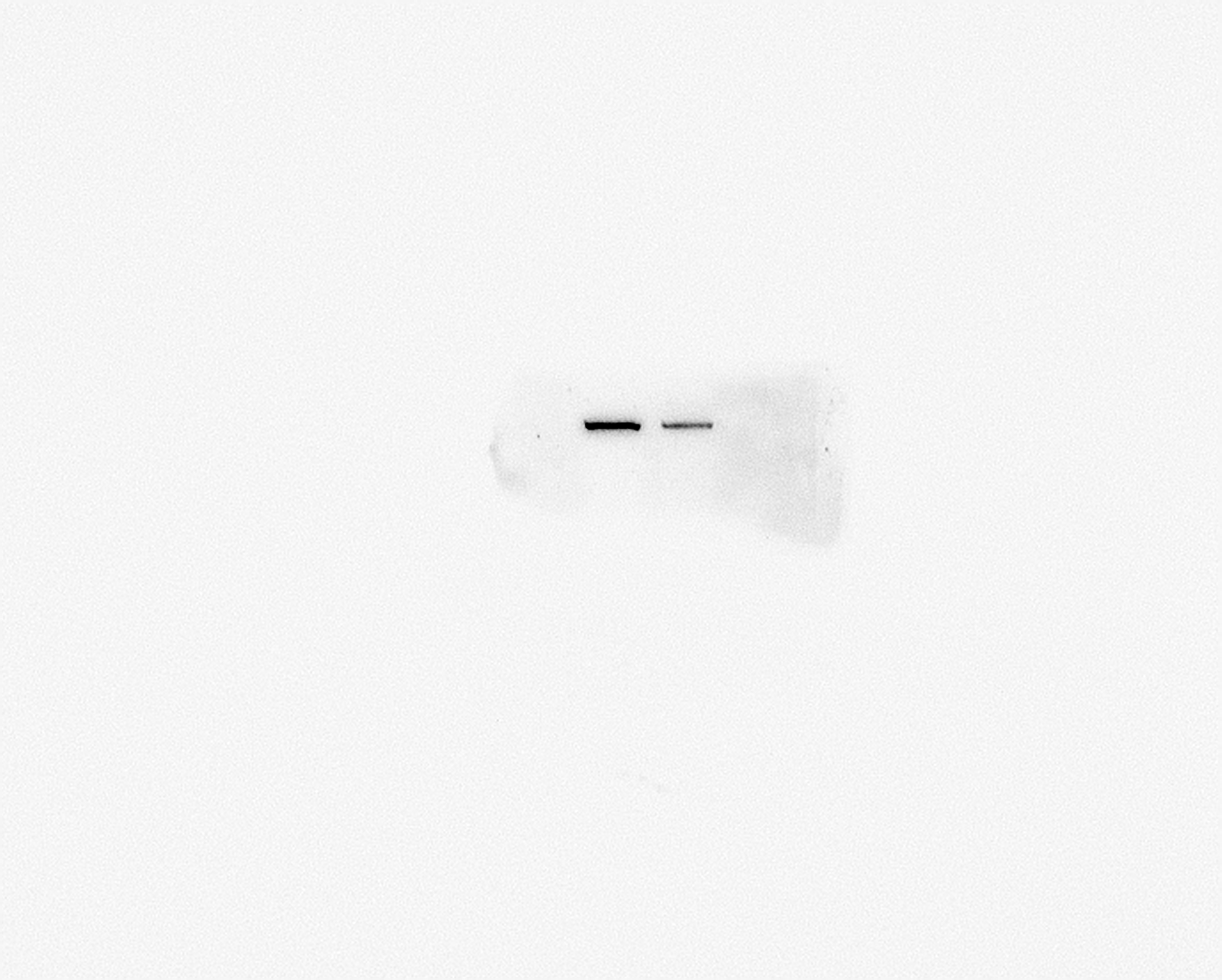

Supplement: Supplementary file 1 — Additional file 1. Raw data. [file 12935_2023_3076_MOESM1_ESM.zip › raw_data/figure7A1/p-smad2/p-SMAD2 1∩╝êΓæáSaoS-2+ si-NC∩╝¢ΓæíSaoS-2+ si-DIO3OS∩╝ë.tif]

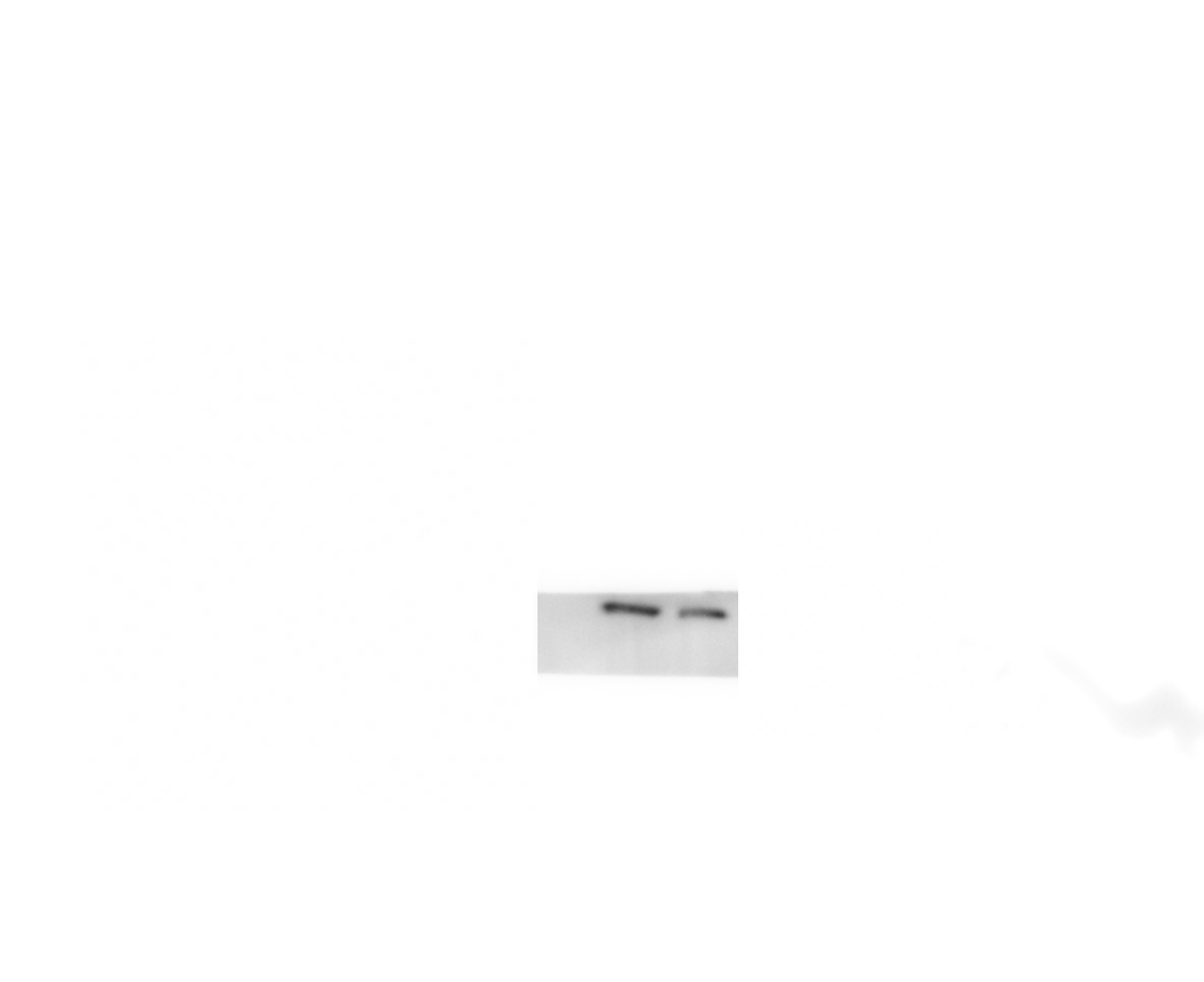

Supplement: Supplementary file 1 — Additional file 1. Raw data. [file 12935_2023_3076_MOESM1_ESM.zip › raw_data/figure7A1/p-smad2/p-SMAD2 2∩╝êΓæáSaoS-2+ si-NC∩╝¢ΓæíSaoS-2+ si-DIO3OS∩╝ë.tif]

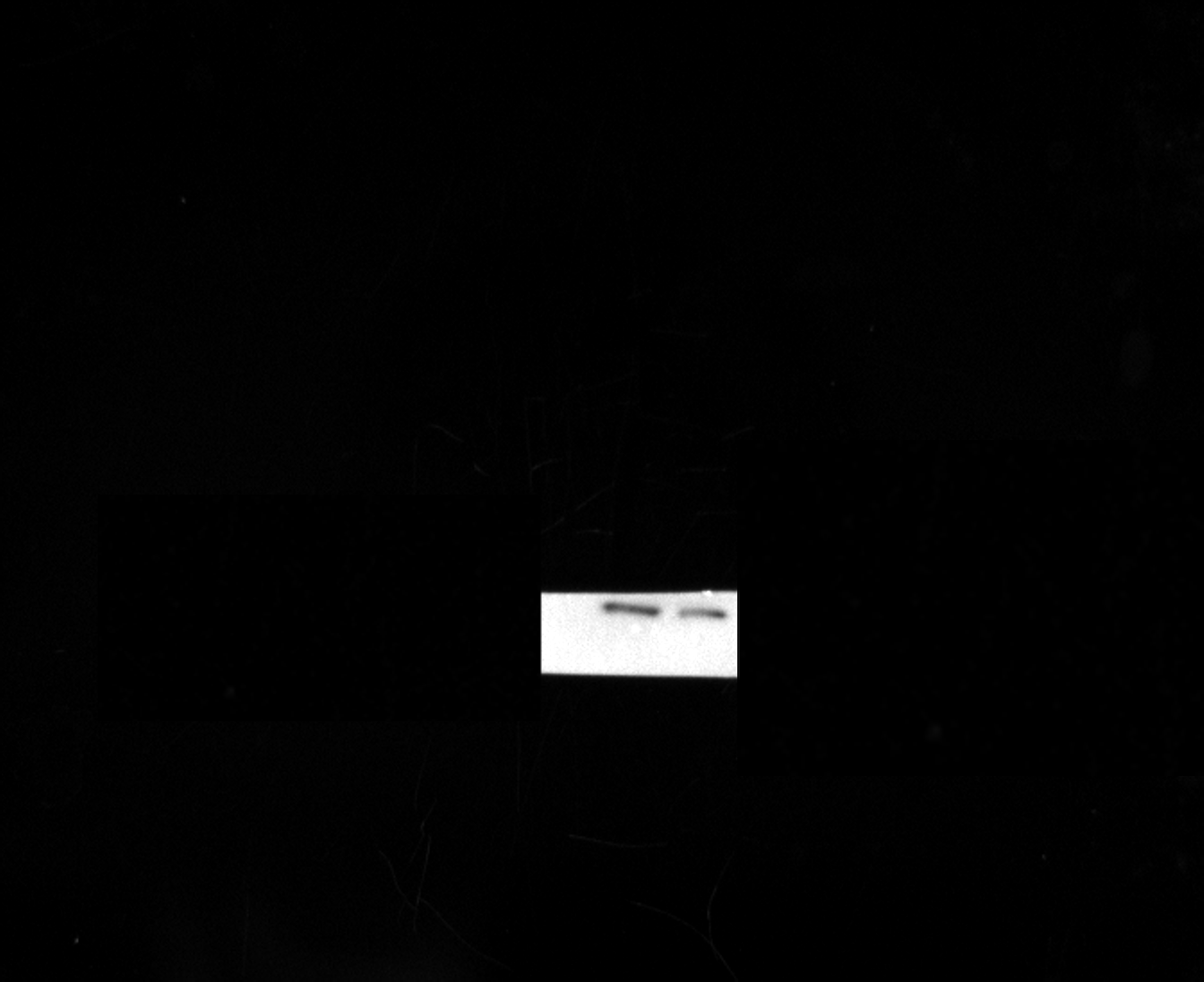

Supplement: Supplementary file 1 — Additional file 1. Raw data. [file 12935_2023_3076_MOESM1_ESM.zip › raw_data/figure7A1/p-smad2/p-SMAD2 2τÖ╜σàë∩╝êΓæáSaoS-2+ si-NC∩╝¢ΓæíSaoS-2+ si-DIO3OS∩╝ë.tif]

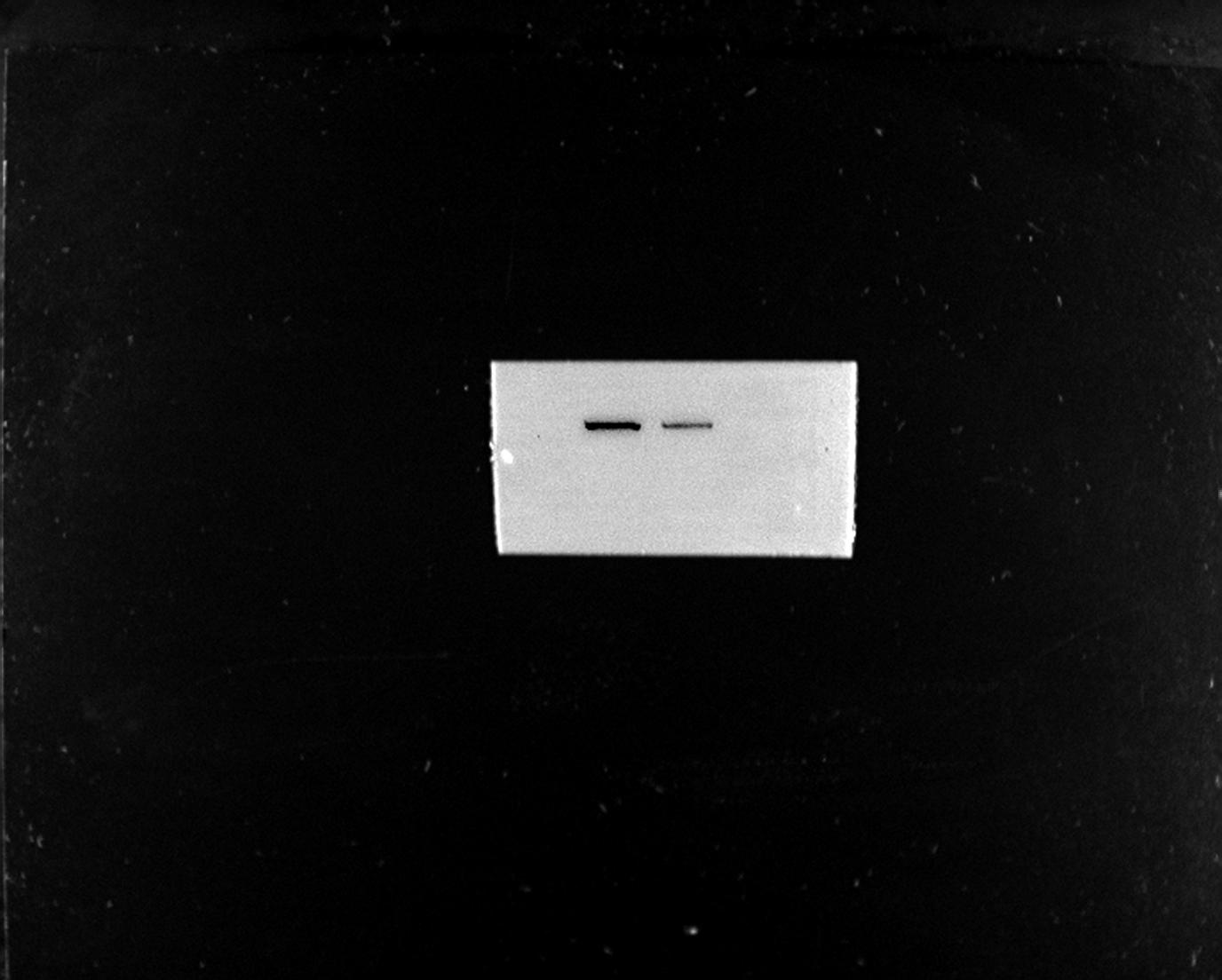

Supplement: Supplementary file 1 — Additional file 1. Raw data. [file 12935_2023_3076_MOESM1_ESM.zip › raw_data/figure7A1/p-smad2/p-SMAD2 1τÖ╜σàë∩╝êΓæáSaoS-2+ si-NC∩╝¢ΓæíSaoS-2+ si-DIO3OS∩╝ë.tif]

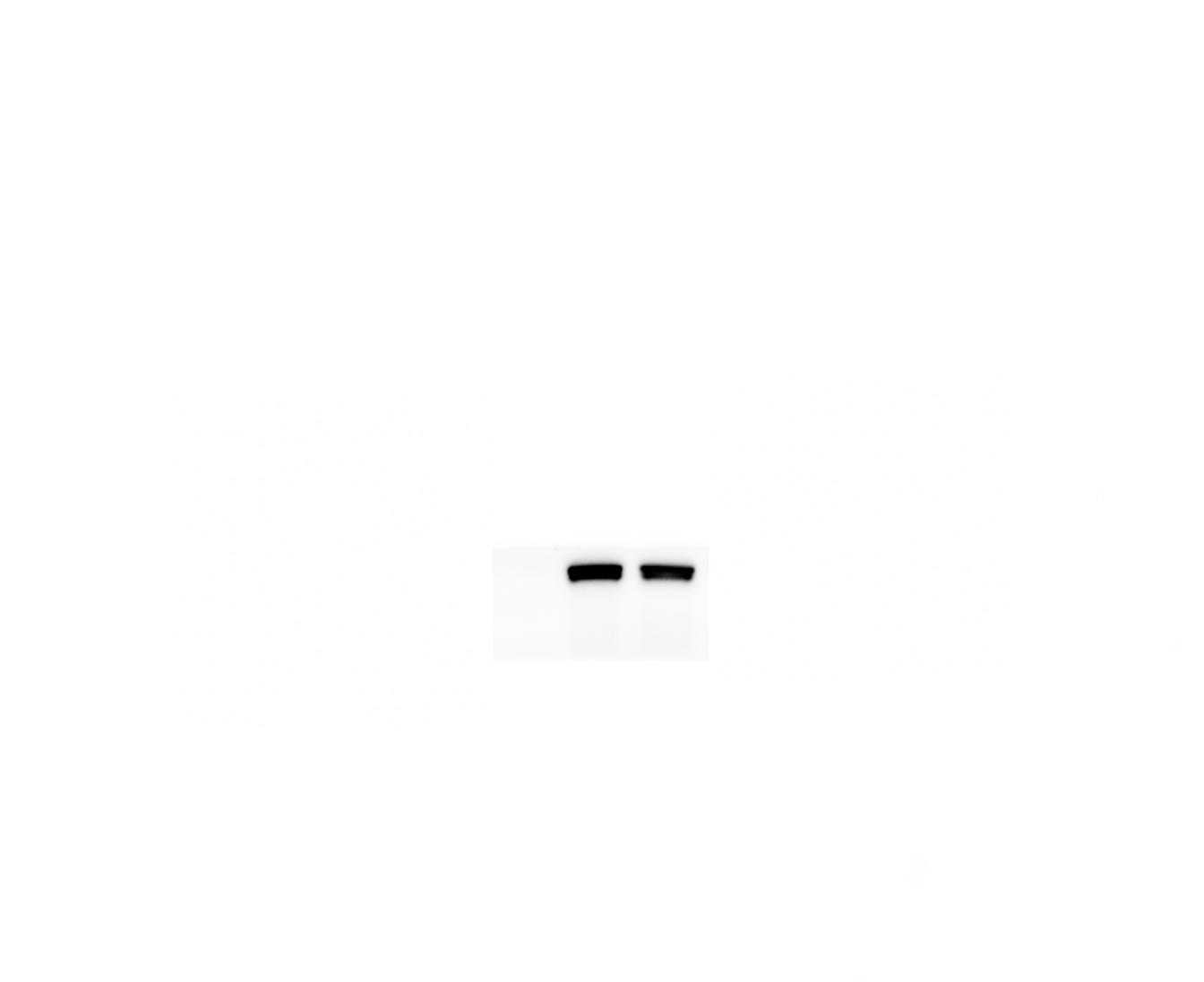

Supplement: Supplementary file 1 — Additional file 1. Raw data. [file 12935_2023_3076_MOESM1_ESM.zip › raw_data/figure7A1/gapdh/GAPDH 2∩╝êΓæáSaoS-2+ si-NC∩╝¢ΓæíSaoS-2+ si-DIO3OS∩╝ë.tif.Tif]

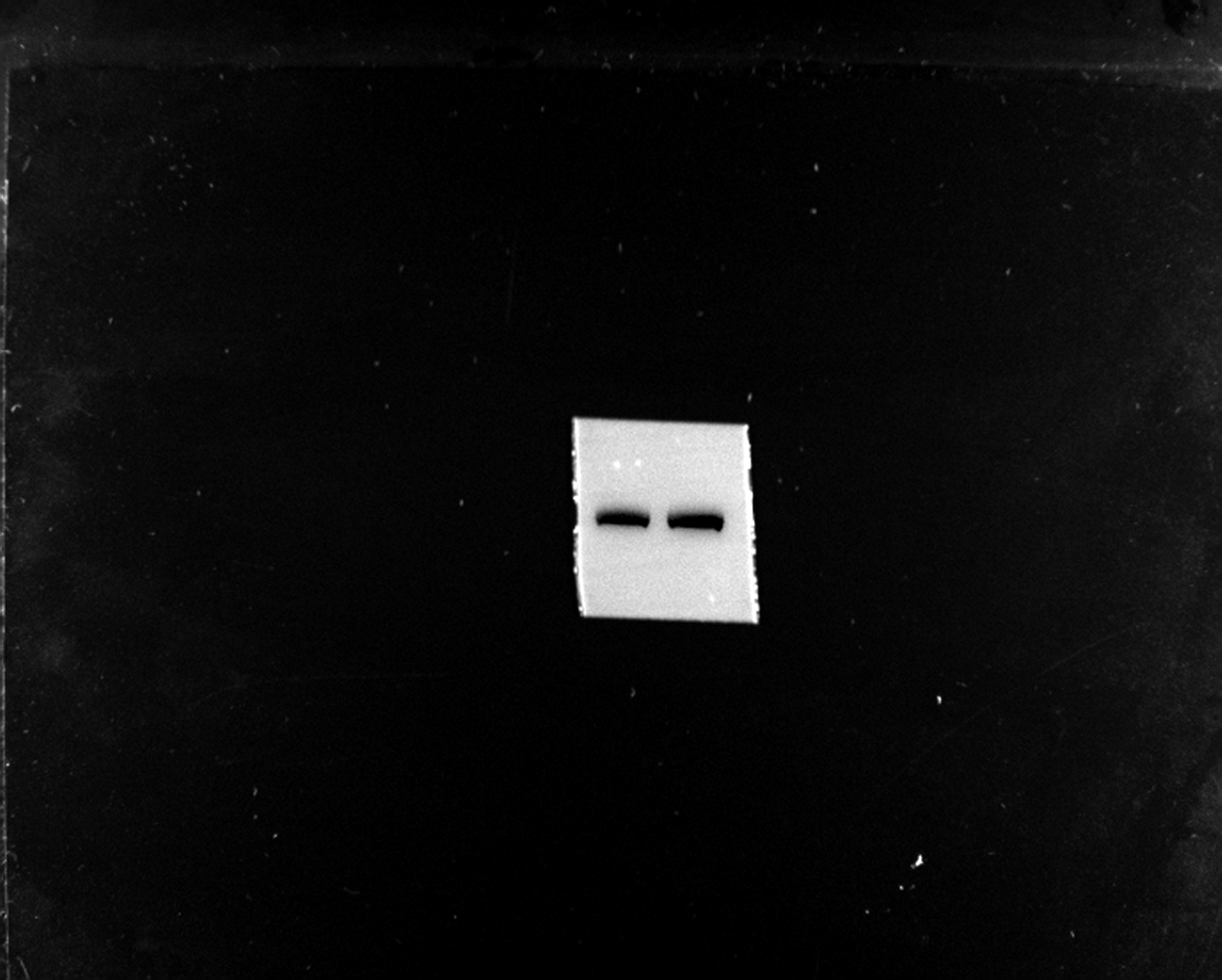

Supplement: Supplementary file 1 — Additional file 1. Raw data. [file 12935_2023_3076_MOESM1_ESM.zip › raw_data/figure7A1/gapdh/GAPDH 1τÖ╜σàë∩╝êΓæáSaoS-2+ si-NC∩╝¢ΓæíSaoS-2+ si-DIO3OS∩╝ë.tif]

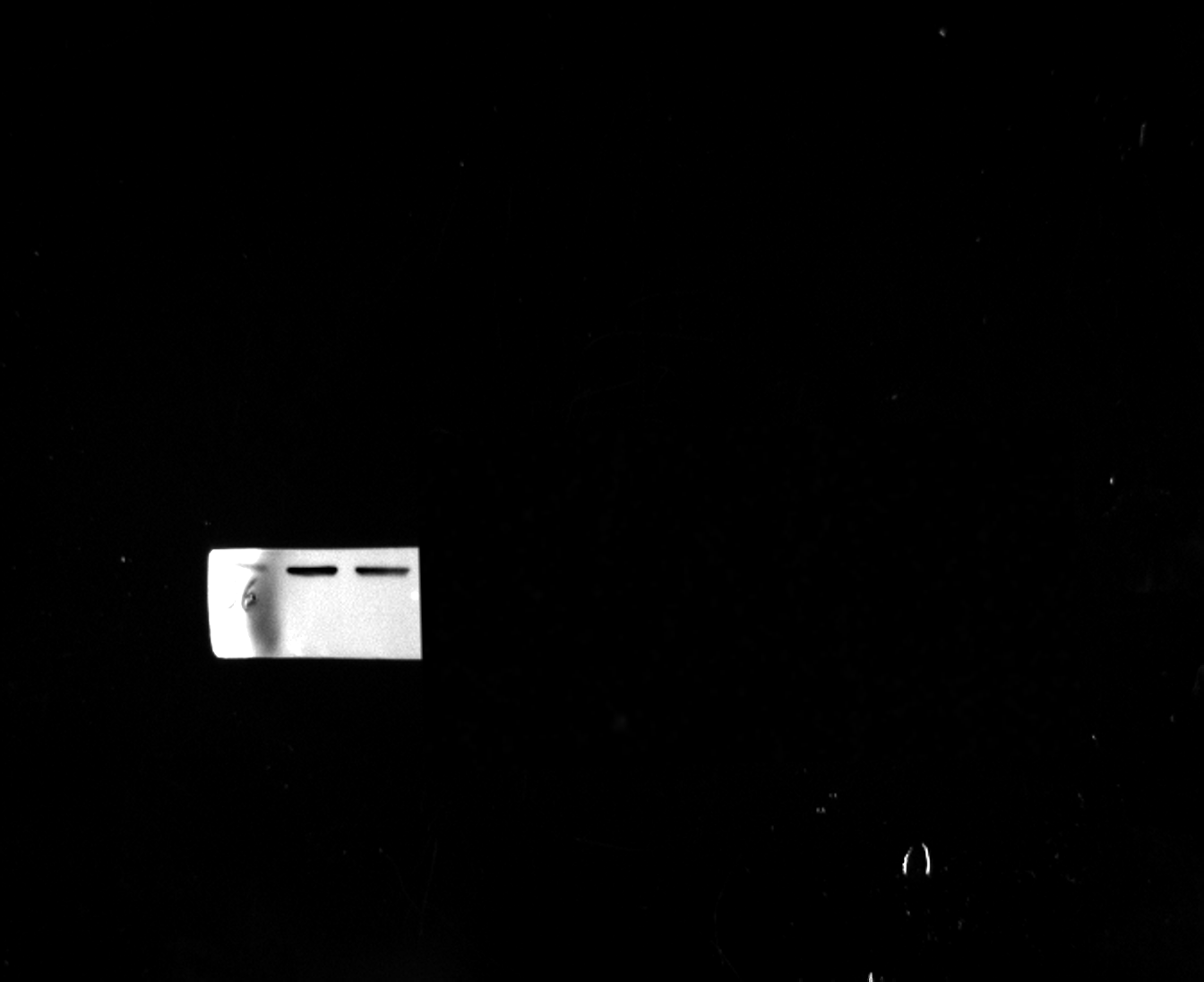

Supplement: Supplementary file 1 — Additional file 1. Raw data. [file 12935_2023_3076_MOESM1_ESM.zip › raw_data/figure7A1/gapdh/GAPDH 3τÖ╜σàë∩╝êΓæáSaoS-2+ si-NC∩╝¢ΓæíSaoS-2+ si-DIO3OS∩╝ë.tif.tif]

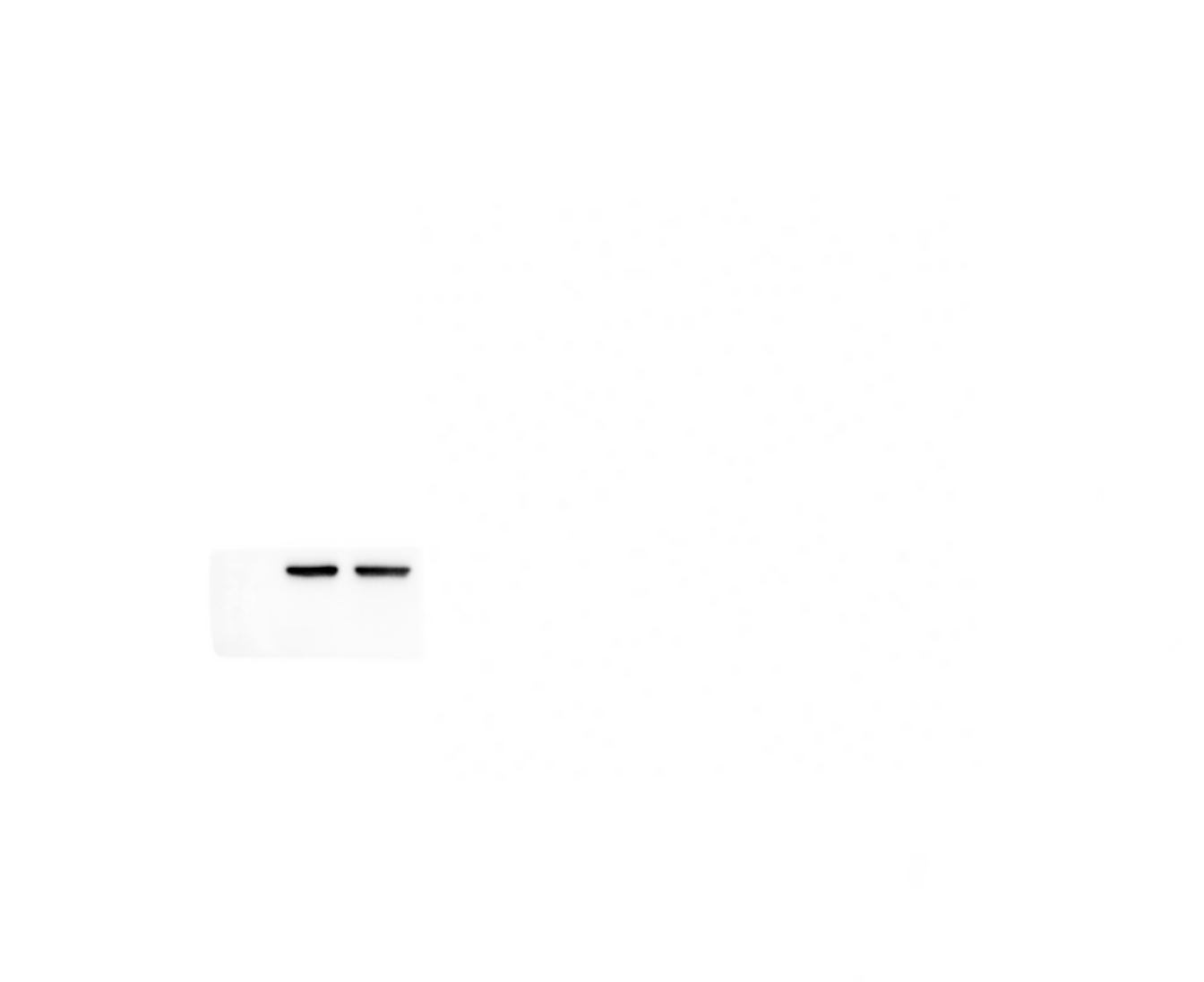

Supplement: Supplementary file 1 — Additional file 1. Raw data. [file 12935_2023_3076_MOESM1_ESM.zip › raw_data/figure7A1/gapdh/GAPDH 3∩╝êΓæáSaoS-2+ si-NC∩╝¢ΓæíSaoS-2+ si-DIO3OS∩╝ë.tif.tif]

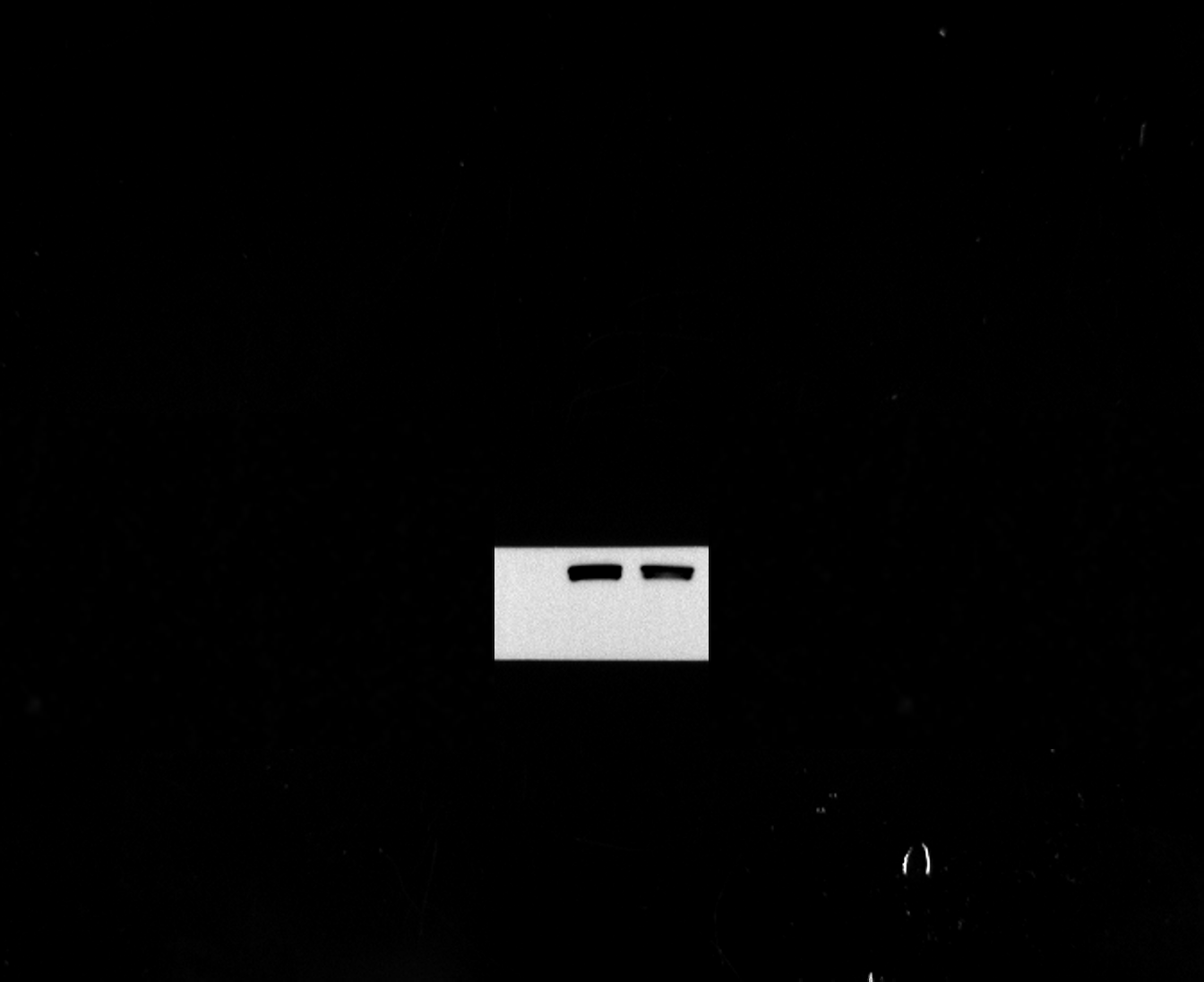

Supplement: Supplementary file 1 — Additional file 1. Raw data. [file 12935_2023_3076_MOESM1_ESM.zip › raw_data/figure7A1/gapdh/GAPDH 2τÖ╜σàë∩╝êΓæáSaoS-2+ si-NC∩╝¢ΓæíSaoS-2+ si-DIO3OS∩╝ë.tif.Tif]

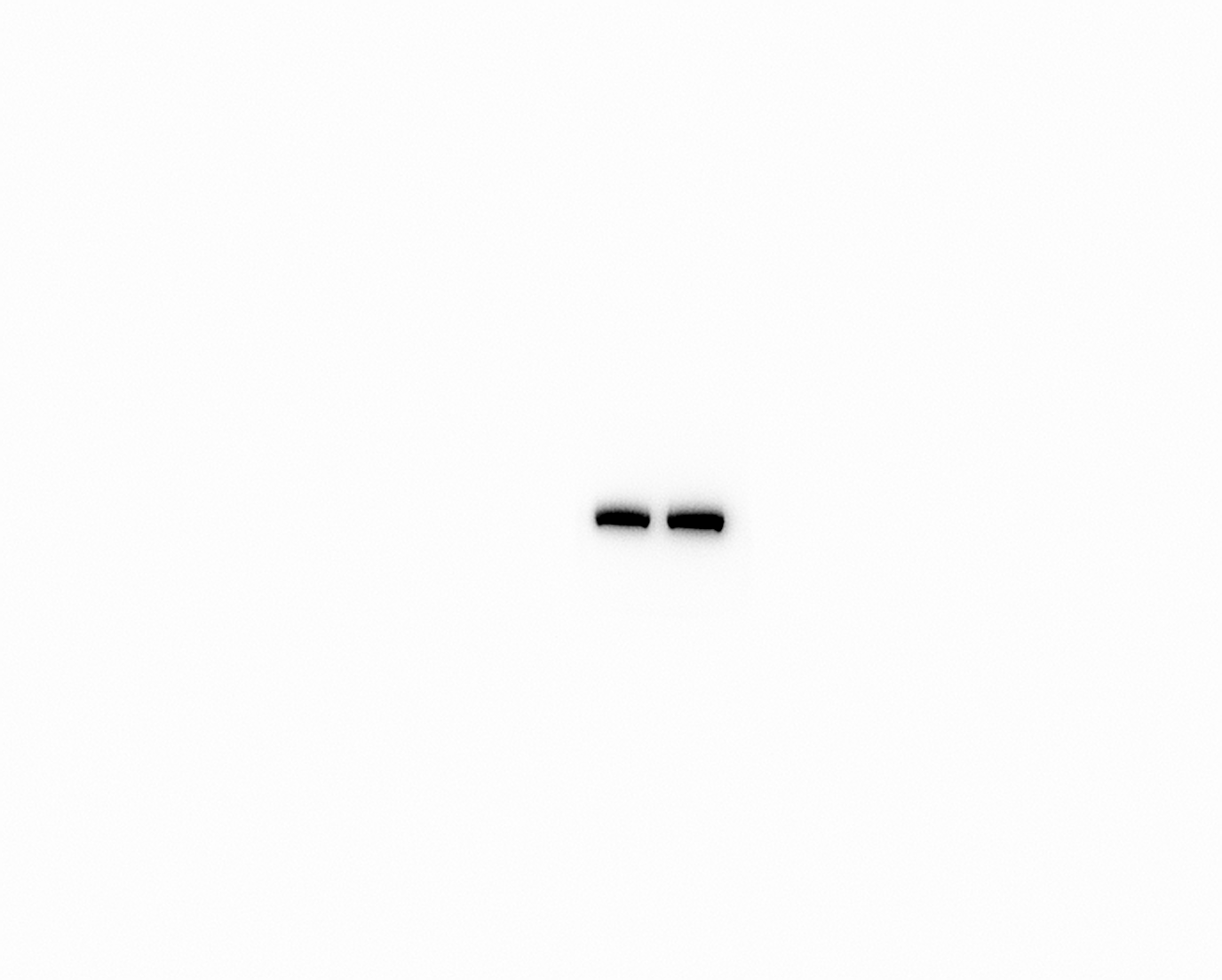

Supplement: Supplementary file 1 — Additional file 1. Raw data. [file 12935_2023_3076_MOESM1_ESM.zip › raw_data/figure7A1/gapdh/GAPDH 1∩╝êΓæáSaoS-2+ si-NC∩╝¢ΓæíSaoS-2+ si-DIO3OS∩╝ë.tif]

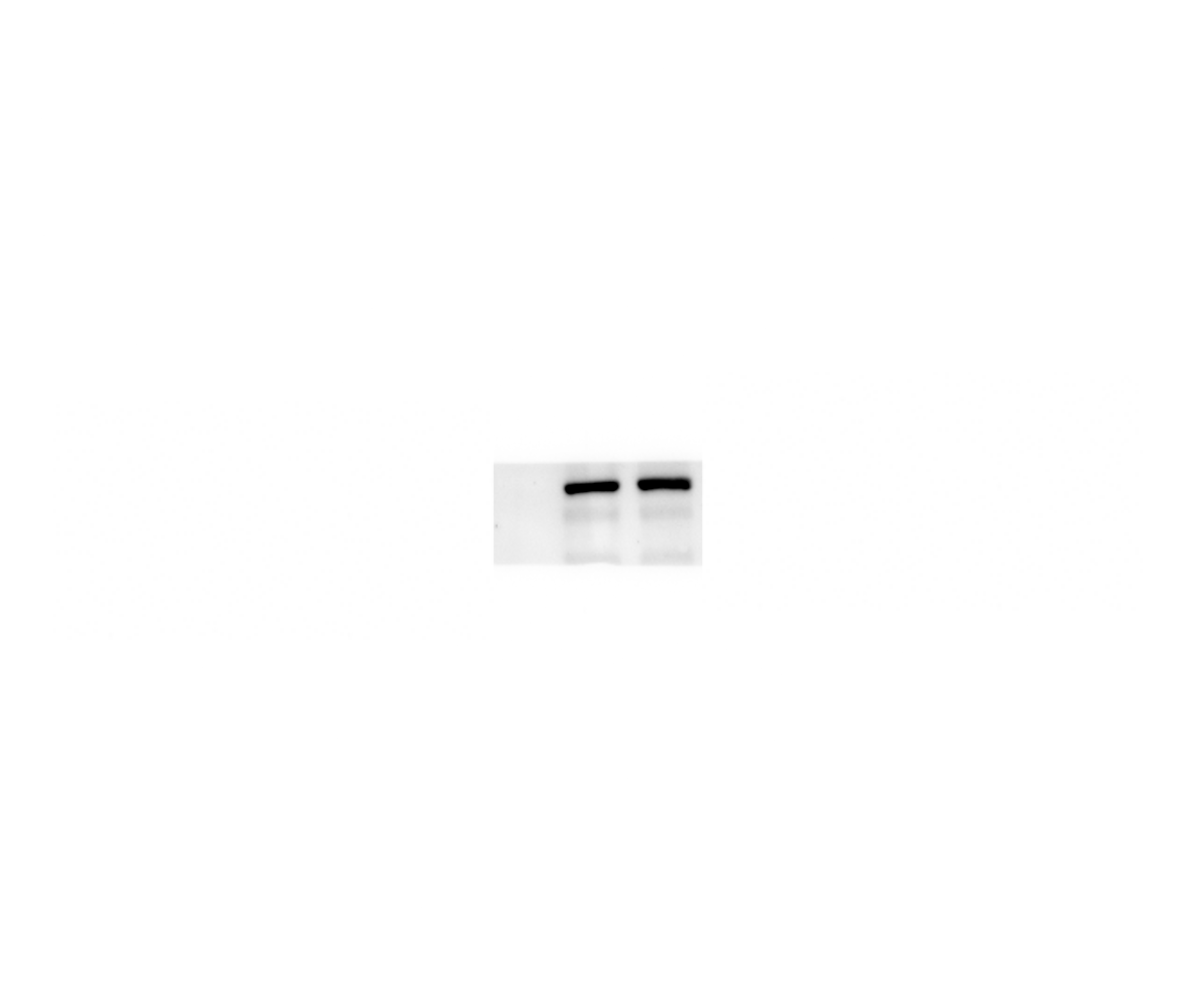

Supplement: Supplementary file 1 — Additional file 1. Raw data. [file 12935_2023_3076_MOESM1_ESM.zip › raw_data/figure7B1/smad2/SMAD2 2∩╝êΓæóU2OS+si-NC∩╝¢ΓæúU2OS+si-DIO3OS∩╝ë.Tif]

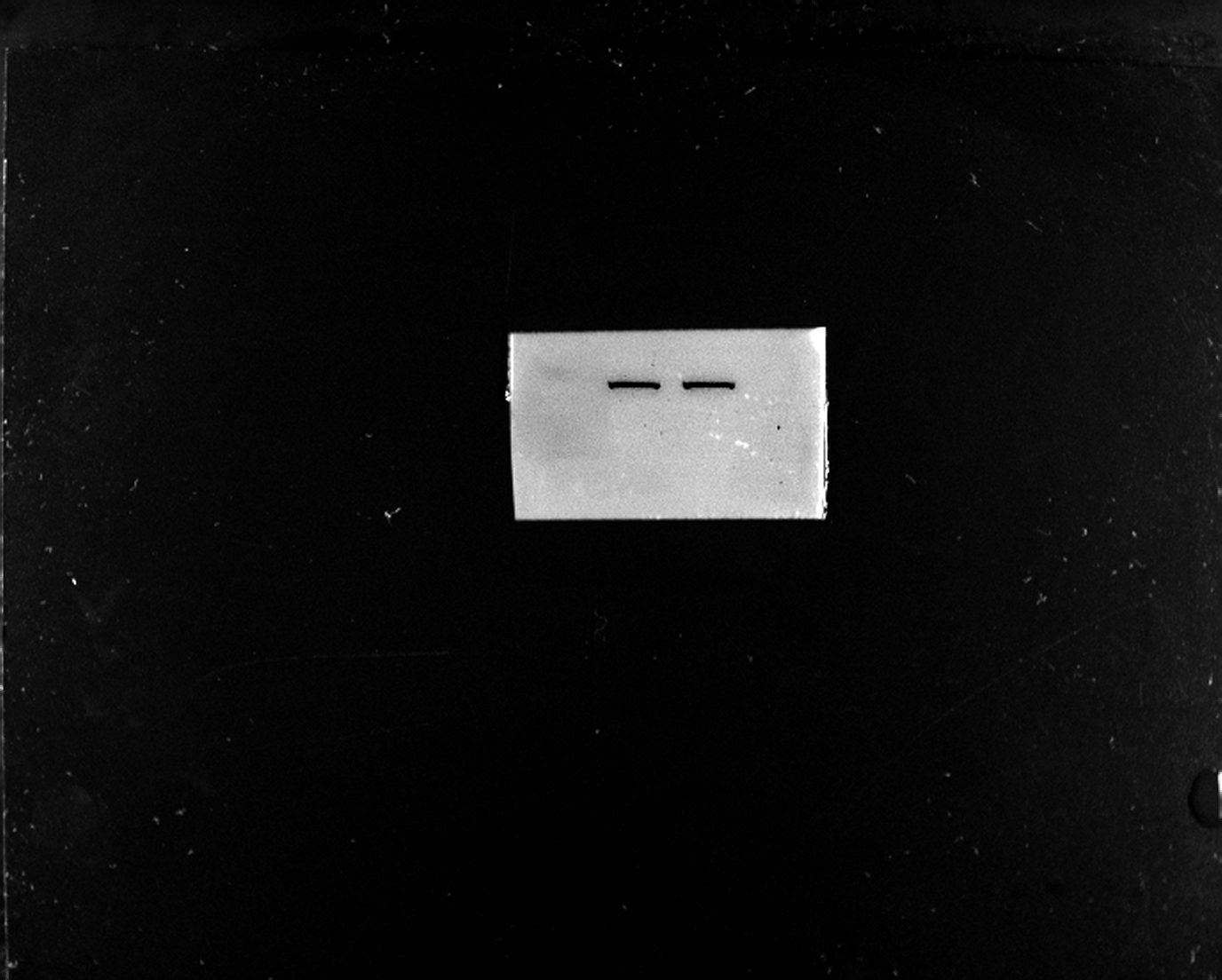

Supplement: Supplementary file 1 — Additional file 1. Raw data. [file 12935_2023_3076_MOESM1_ESM.zip › raw_data/figure7B1/smad2/SMAD2 1τÖ╜σàë∩╝êΓæóU2OS+si-NC∩╝¢ΓæúU2OS+si-DIO3OS∩╝ë.tif]

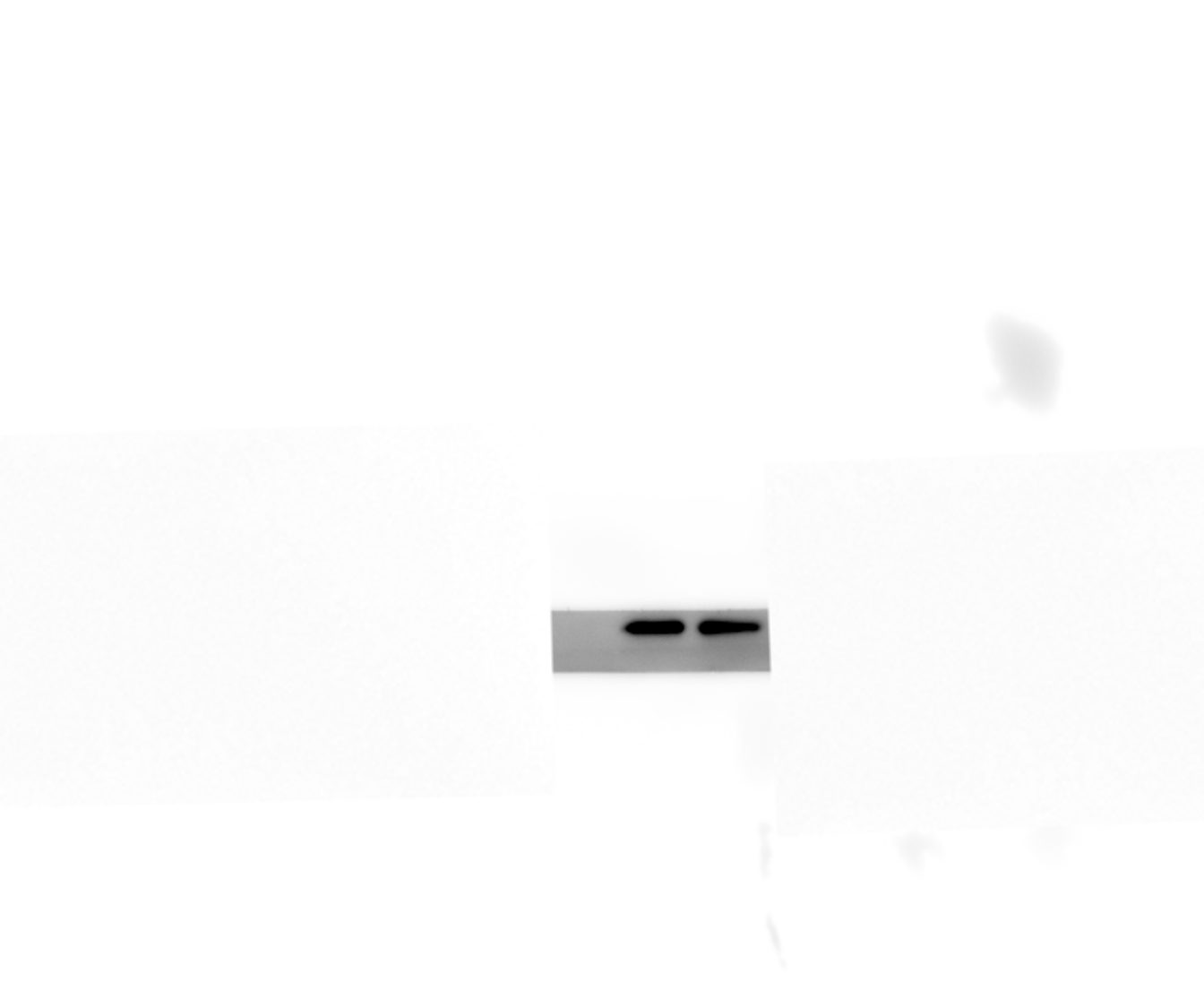

Supplement: Supplementary file 1 — Additional file 1. Raw data. [file 12935_2023_3076_MOESM1_ESM.zip › raw_data/figure7B1/smad2/SMAD2 3∩╝êΓæóU2OS+si-NC∩╝¢ΓæúU2OS+si-DIO3OS∩╝ë.Tif]

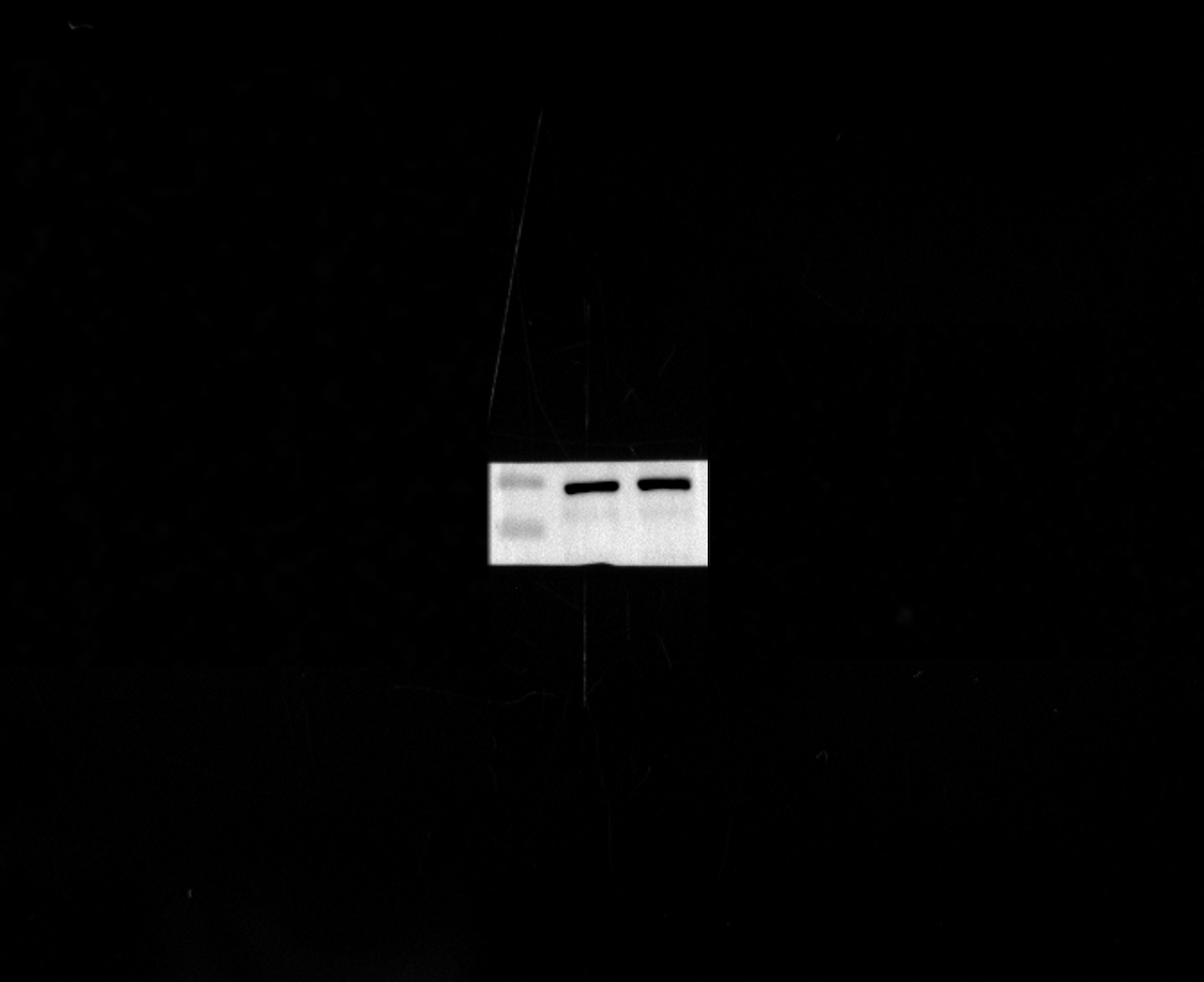

Supplement: Supplementary file 1 — Additional file 1. Raw data. [file 12935_2023_3076_MOESM1_ESM.zip › raw_data/figure7B1/smad2/SMAD2 2τÖ╜σàë∩╝êΓæóU2OS+si-NC∩╝¢ΓæúU2OS+si-DIO3OS∩╝ë.Tif]

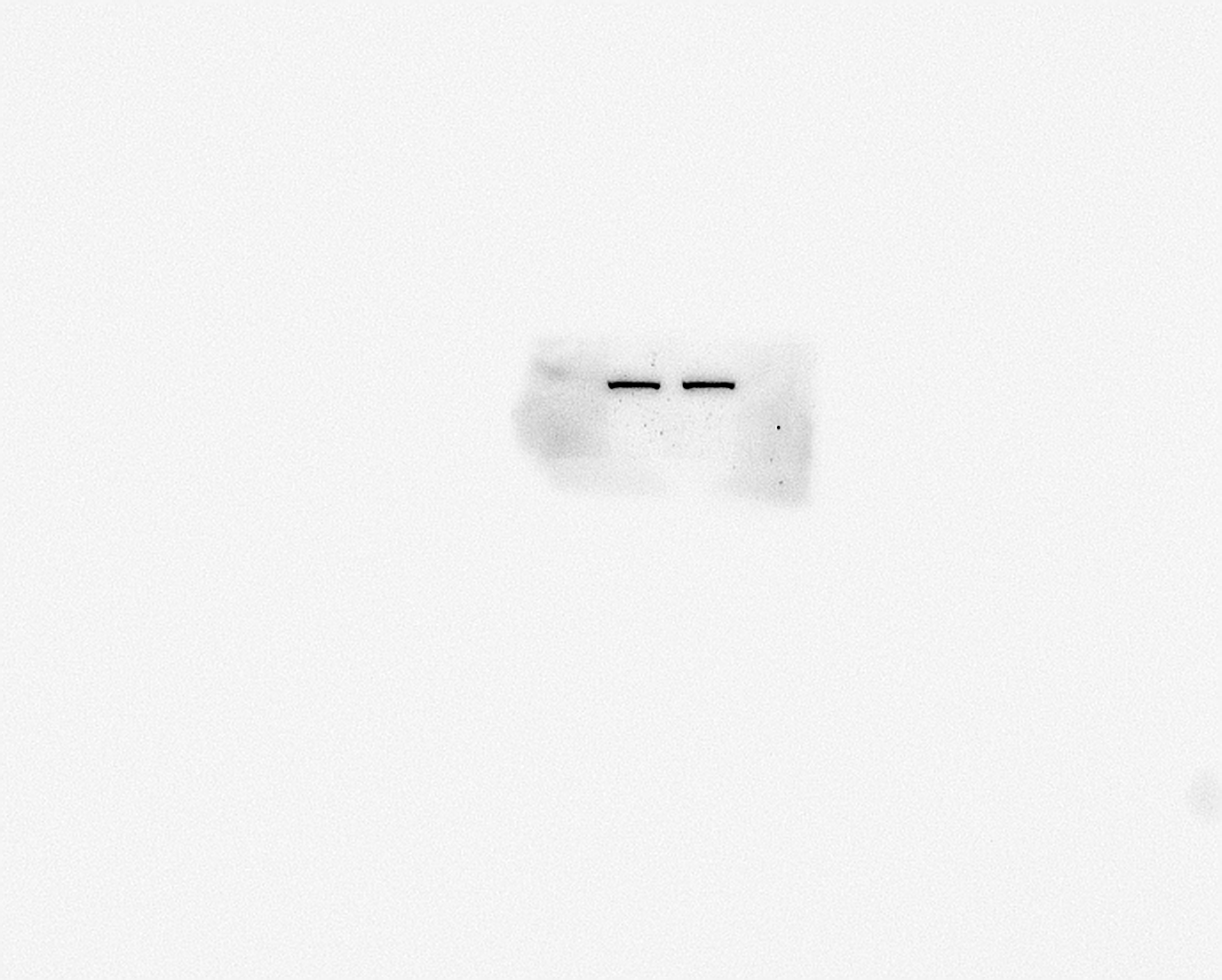

Supplement: Supplementary file 1 — Additional file 1. Raw data. [file 12935_2023_3076_MOESM1_ESM.zip › raw_data/figure7B1/smad2/SMAD2 1(ΓæóU2OS+si-NC∩╝¢ΓæúU2OS+si-DIO3OS∩╝ë.tif]

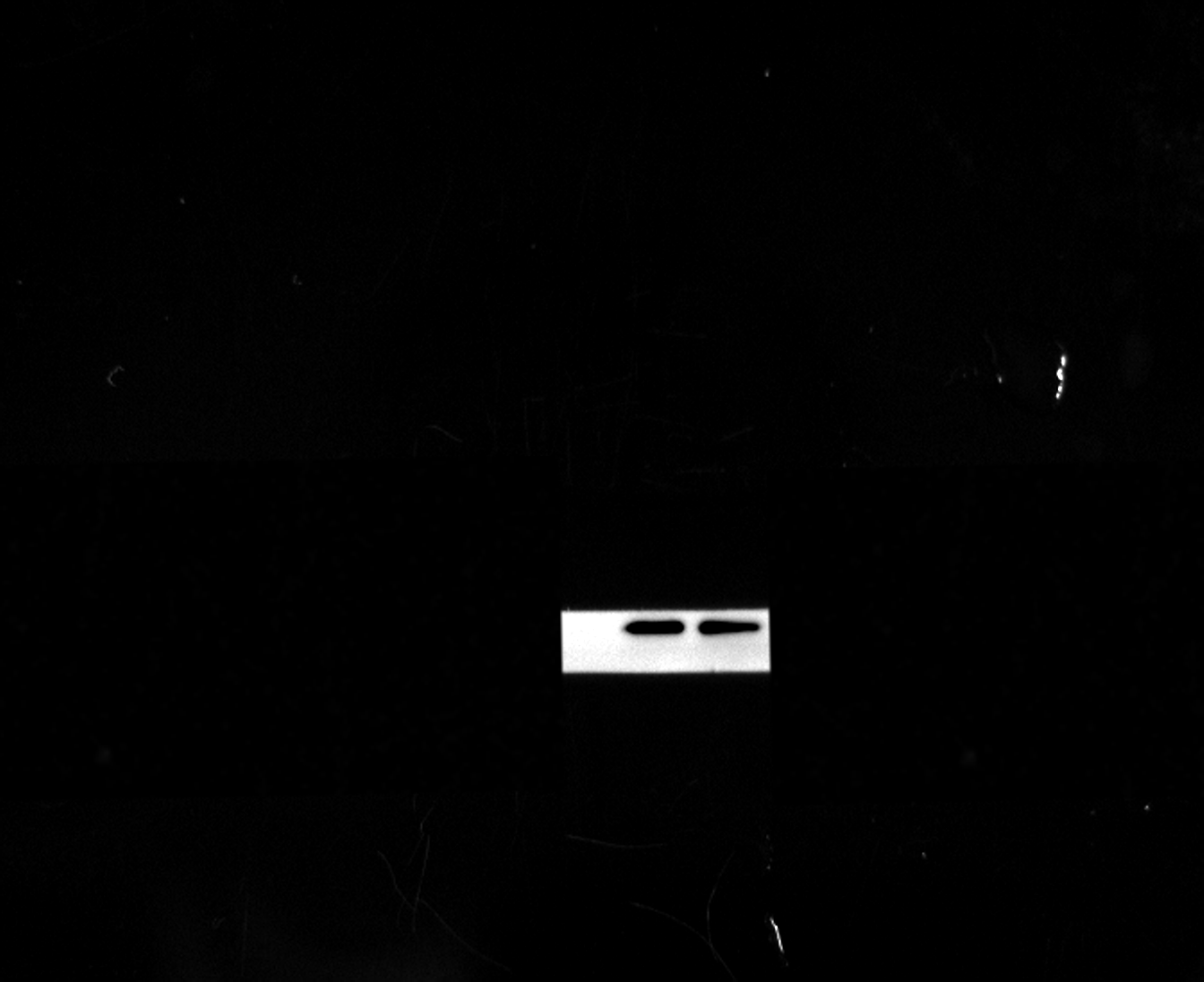

Supplement: Supplementary file 1 — Additional file 1. Raw data. [file 12935_2023_3076_MOESM1_ESM.zip › raw_data/figure7B1/smad2/SMAD2 3τÖ╜σàë∩╝êΓæóU2OS+si-NC∩╝¢ΓæúU2OS+si-DIO3OS∩╝ë.Tif]

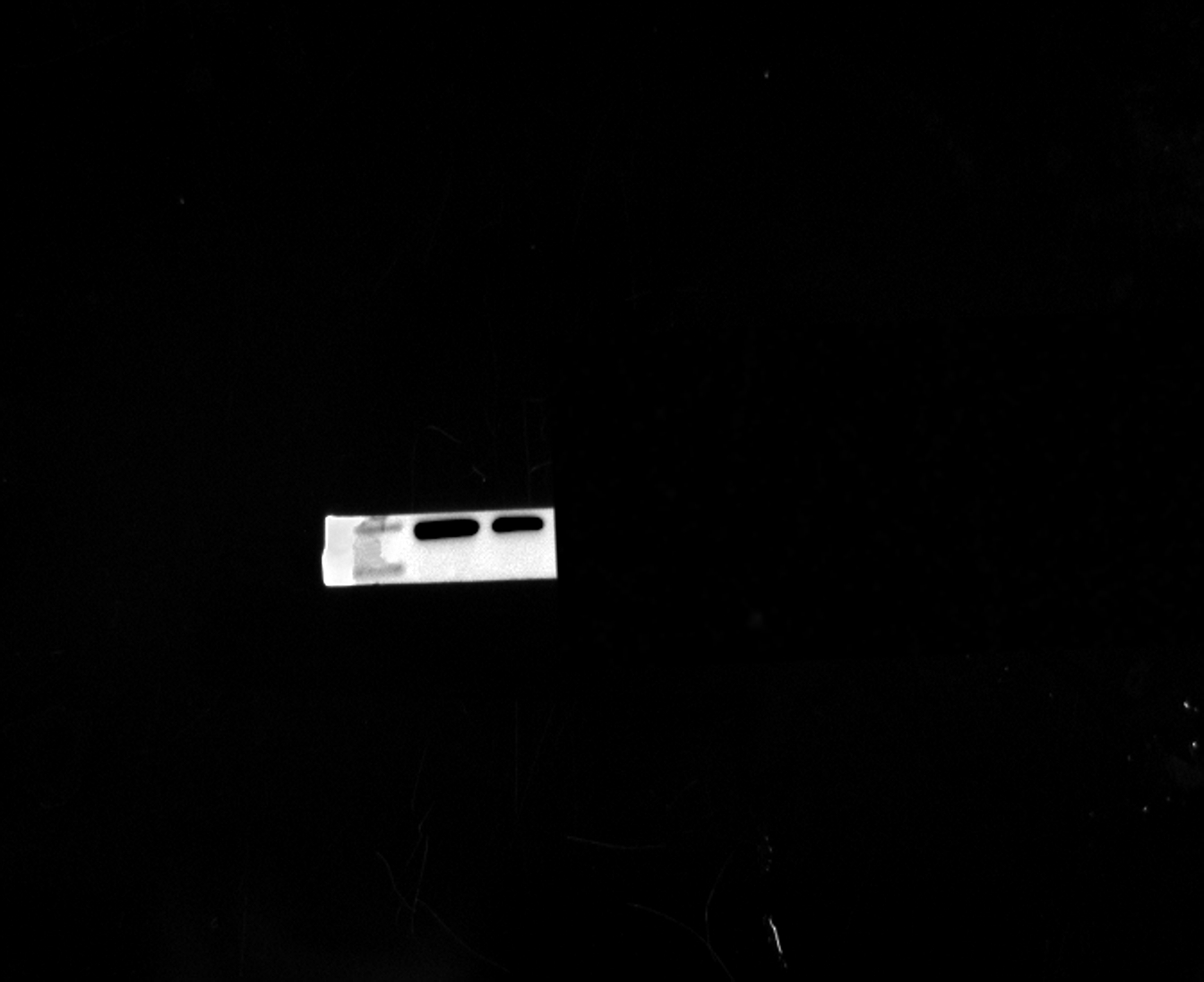

Supplement: Supplementary file 1 — Additional file 1. Raw data. [file 12935_2023_3076_MOESM1_ESM.zip › raw_data/figure7B1/p-smad2/p-SMAD2 3τÖ╜σàë∩╝êΓæóU2OS+si-NC∩╝¢ΓæúU2OS+si-DIO3OS∩╝ë.tif]

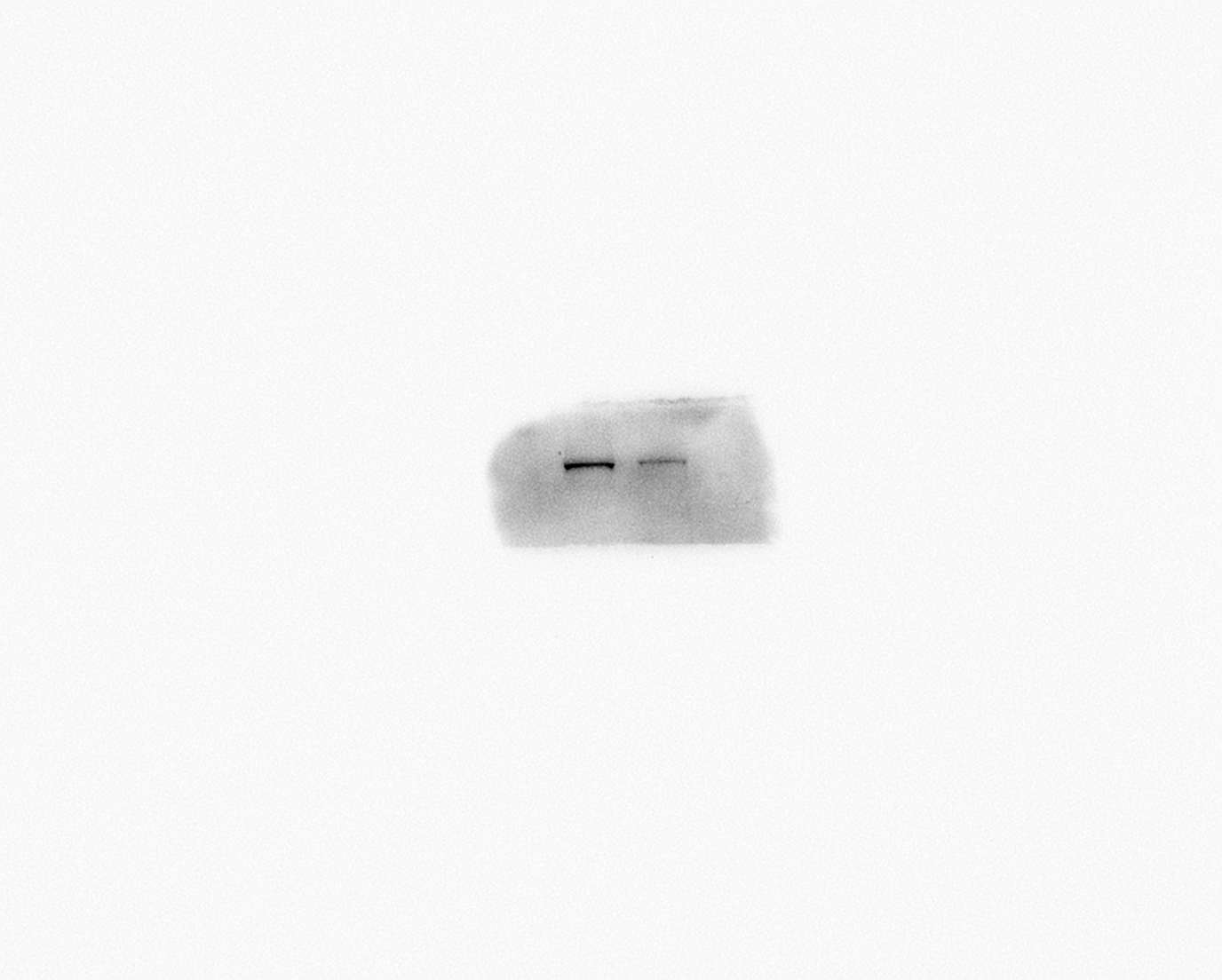

Supplement: Supplementary file 1 — Additional file 1. Raw data. [file 12935_2023_3076_MOESM1_ESM.zip › raw_data/figure7B1/p-smad2/p-SMAD2 1∩╝êΓæóU2OS+si-NC∩╝¢ΓæúU2OS+si-DIO3OS∩╝ë.tif]

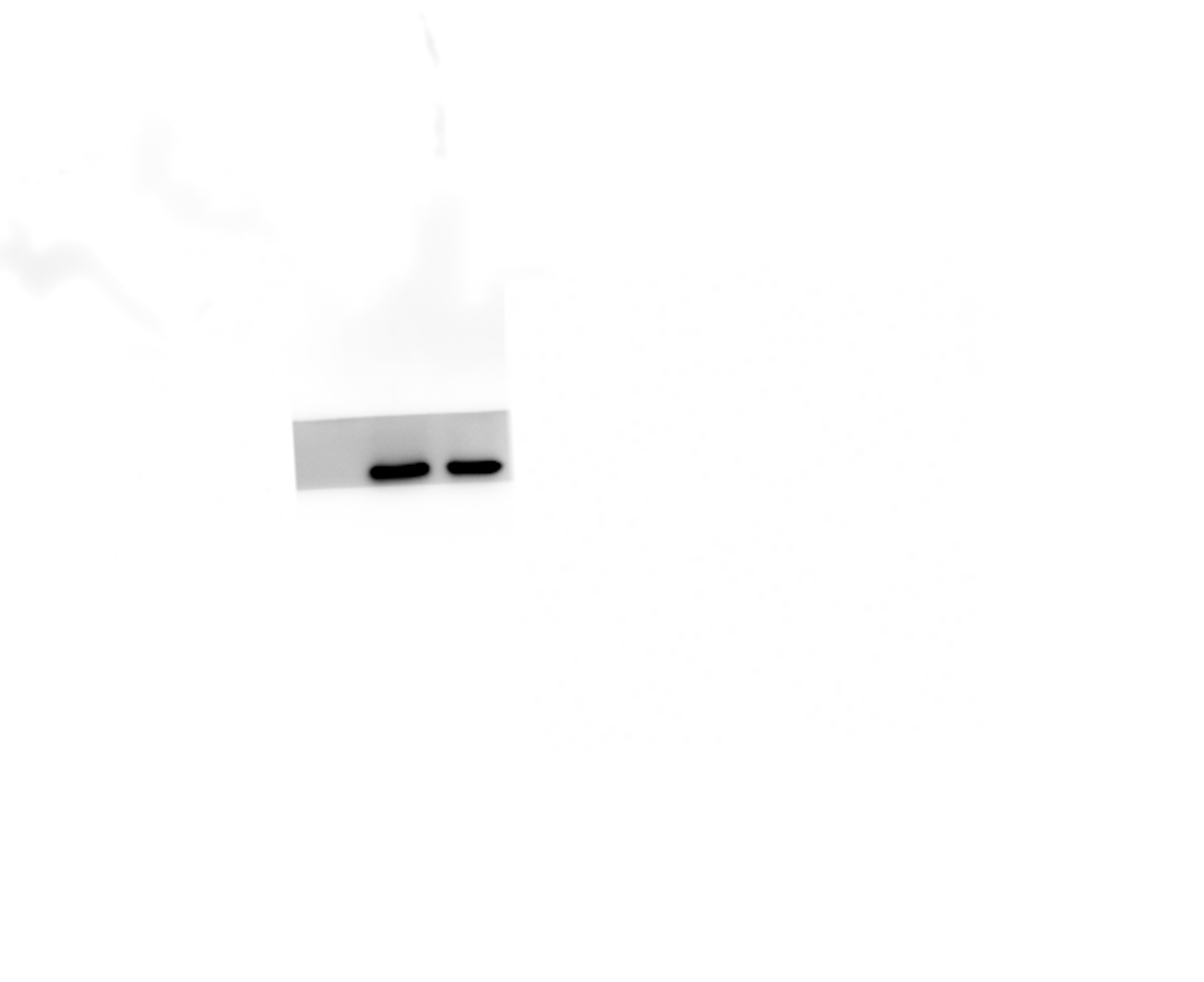

Supplement: Supplementary file 1 — Additional file 1. Raw data. [file 12935_2023_3076_MOESM1_ESM.zip › raw_data/figure7B1/p-smad2/p-SMAD2 2∩╝êΓæóU2OS+si-NC∩╝¢ΓæúU2OS+si-DIO3OS∩╝ë.Tif]

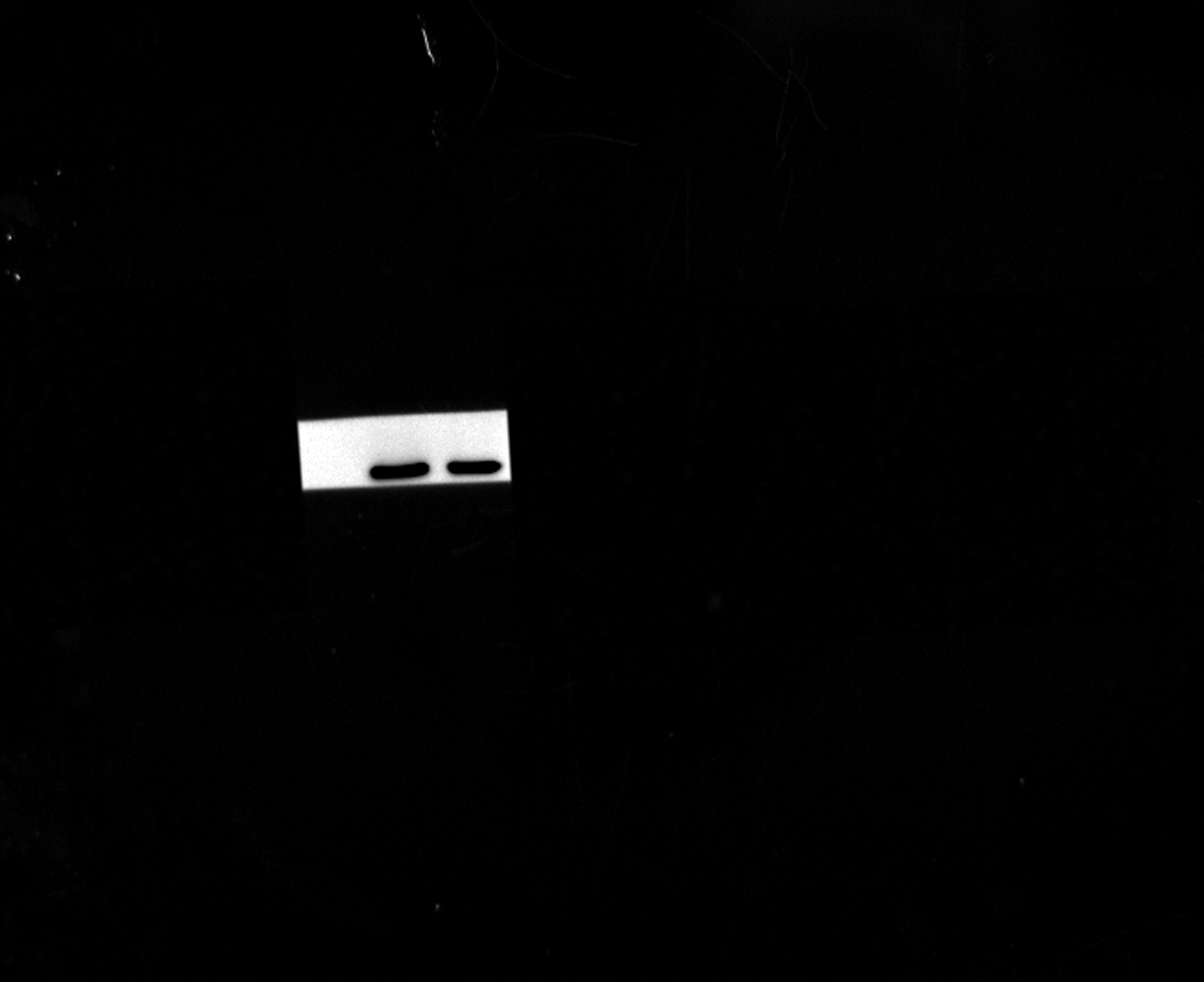

Supplement: Supplementary file 1 — Additional file 1. Raw data. [file 12935_2023_3076_MOESM1_ESM.zip › raw_data/figure7B1/p-smad2/p-SMAD2 2τÖ╜σàë∩╝êΓæóU2OS+si-NC∩╝¢ΓæúU2OS+si-DIO3OS∩╝ë.Tif]

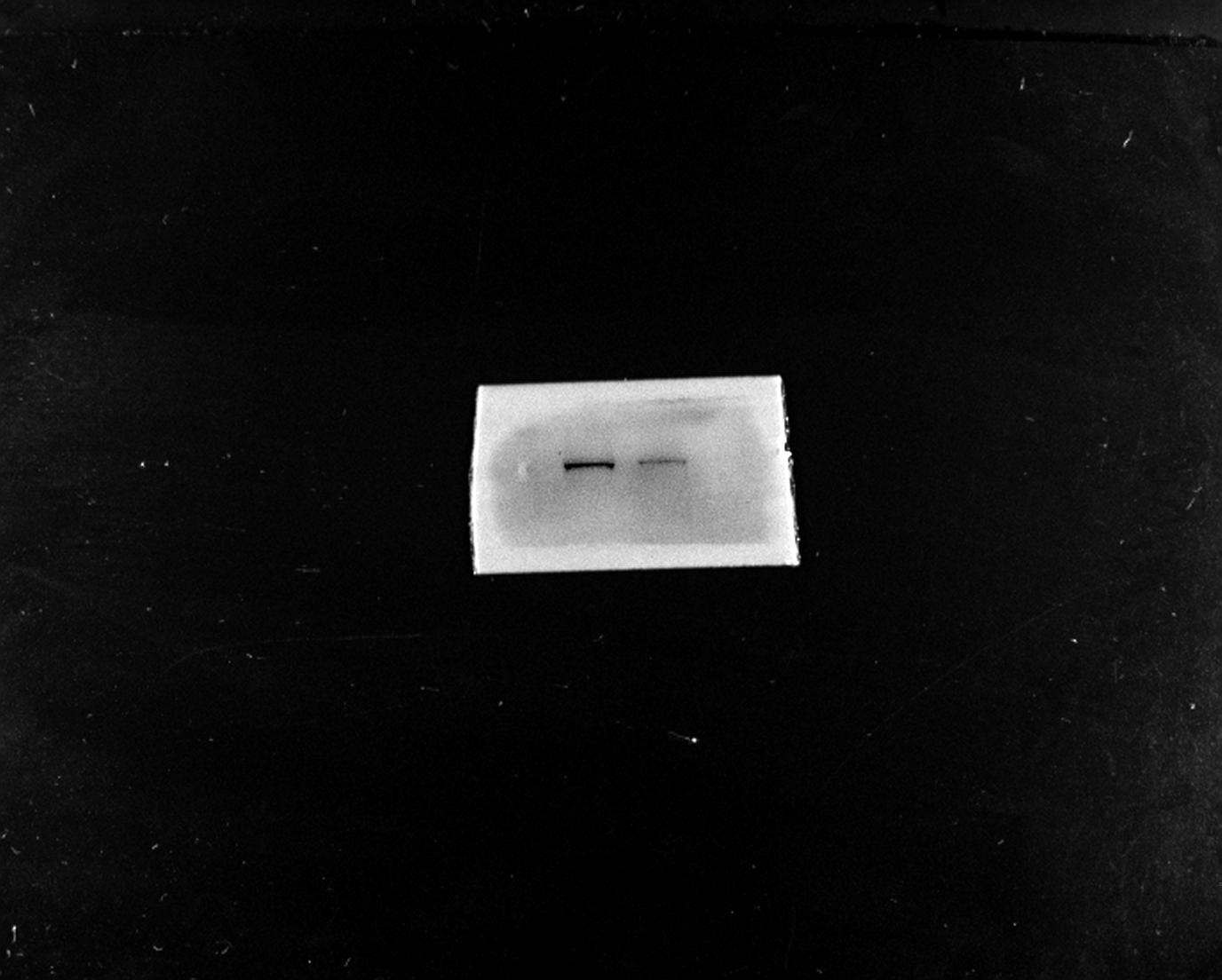

Supplement: Supplementary file 1 — Additional file 1. Raw data. [file 12935_2023_3076_MOESM1_ESM.zip › raw_data/figure7B1/p-smad2/p-SMAD2 1τÖ╜σàë∩╝êΓæóU2OS+si-NC∩╝¢ΓæúU2OS+si-DIO3OS∩╝ë.tif]

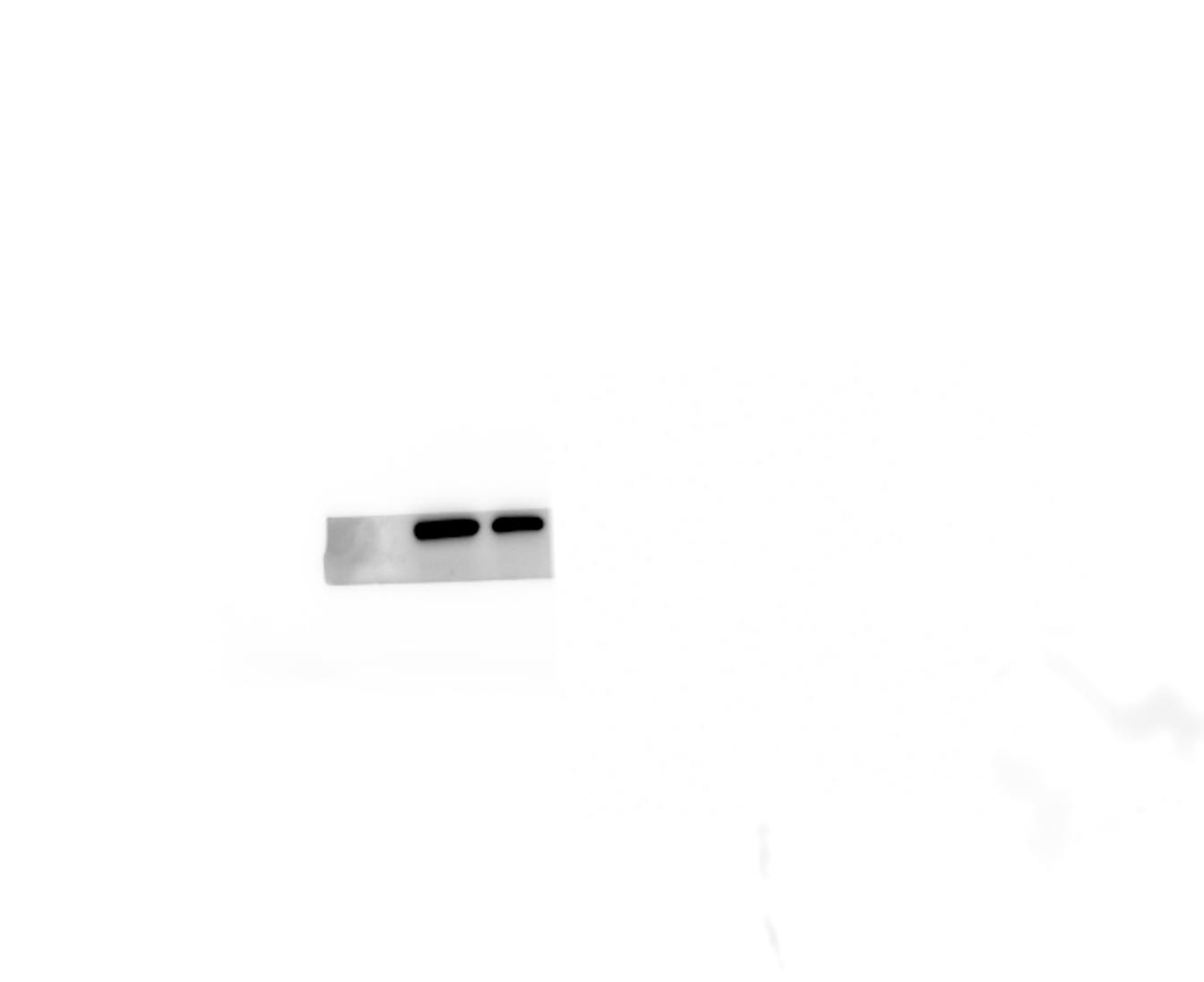

Supplement: Supplementary file 1 — Additional file 1. Raw data. [file 12935_2023_3076_MOESM1_ESM.zip › raw_data/figure7B1/p-smad2/p-SMAD2 3∩╝êΓæóU2OS+si-NC∩╝¢ΓæúU2OS+si-DIO3OS∩╝ë.tif]

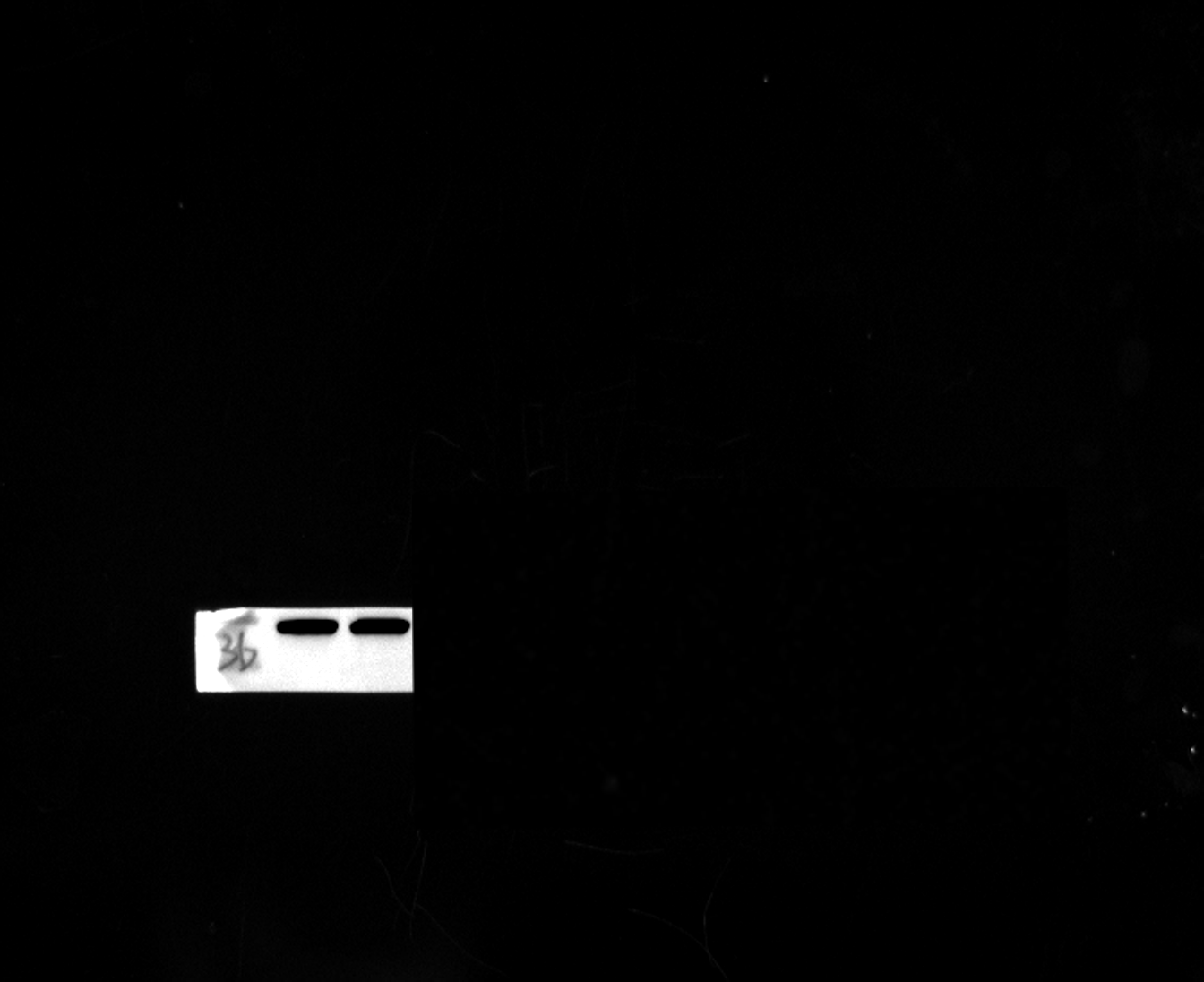

Supplement: Supplementary file 1 — Additional file 1. Raw data. [file 12935_2023_3076_MOESM1_ESM.zip › raw_data/figure7B1/gapdh/GAPDH 2τÖ╜σàë∩╝êΓæóU2OS+si-NC∩╝¢ΓæúU2OS+si-DIO3OS∩╝ë.tif]

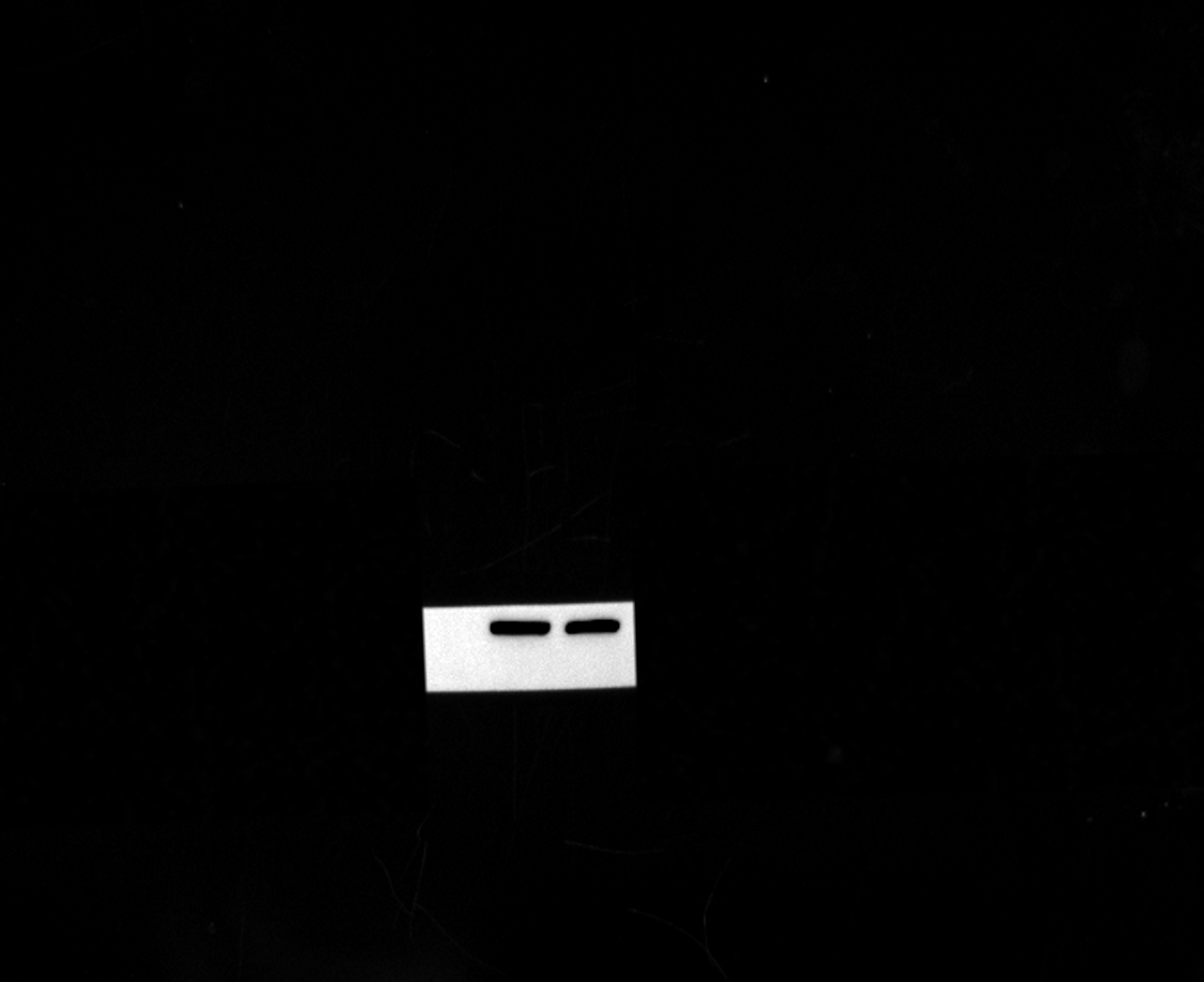

Supplement: Supplementary file 1 — Additional file 1. Raw data. [file 12935_2023_3076_MOESM1_ESM.zip › raw_data/figure7B1/gapdh/GAPDH 3τÖ╜σàë∩╝êΓæóU2OS+si-NC∩╝¢ΓæúU2OS+si-DIO3OS∩╝ë.Tif]

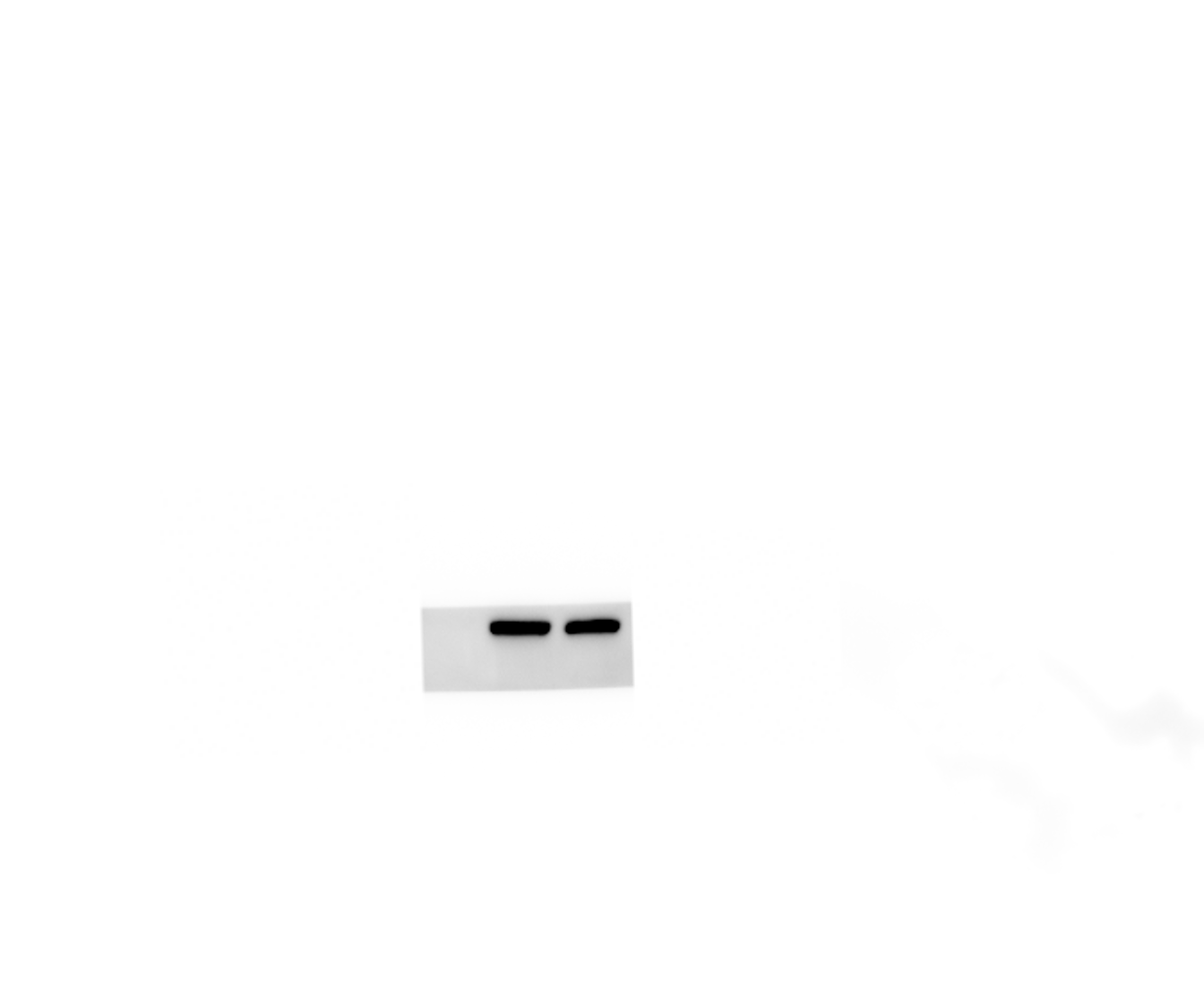

Supplement: Supplementary file 1 — Additional file 1. Raw data. [file 12935_2023_3076_MOESM1_ESM.zip › raw_data/figure7B1/gapdh/GAPDH 3∩╝êΓæóU2OS+si-NC∩╝¢ΓæúU2OS+si-DIO3OS∩╝ë.Tif]

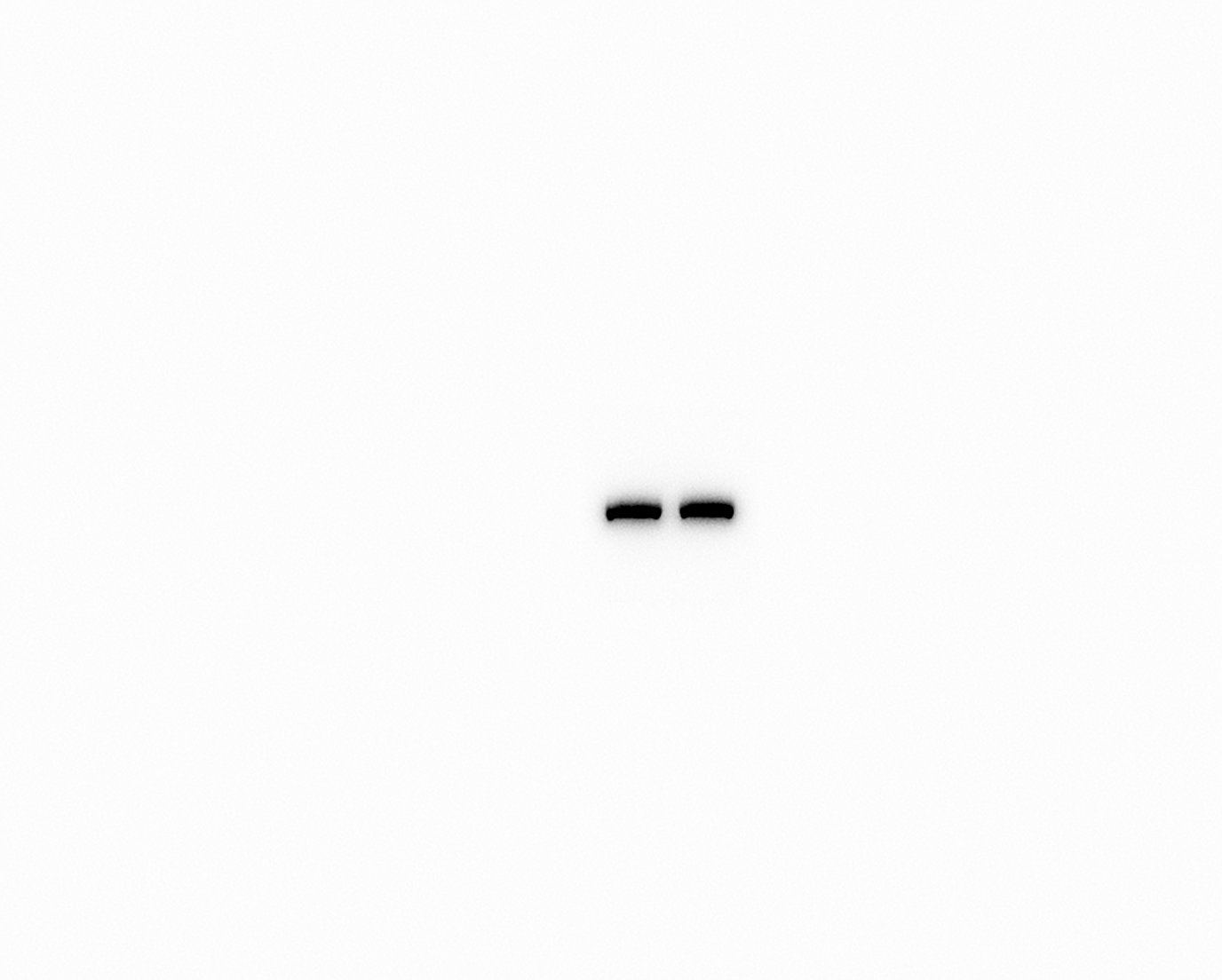

Supplement: Supplementary file 1 — Additional file 1. Raw data. [file 12935_2023_3076_MOESM1_ESM.zip › raw_data/figure7B1/gapdh/GAPDH 1∩╝êΓæóU2OS+si-NC∩╝¢ΓæúU2OS+si-DIO3OS∩╝ë.tif]

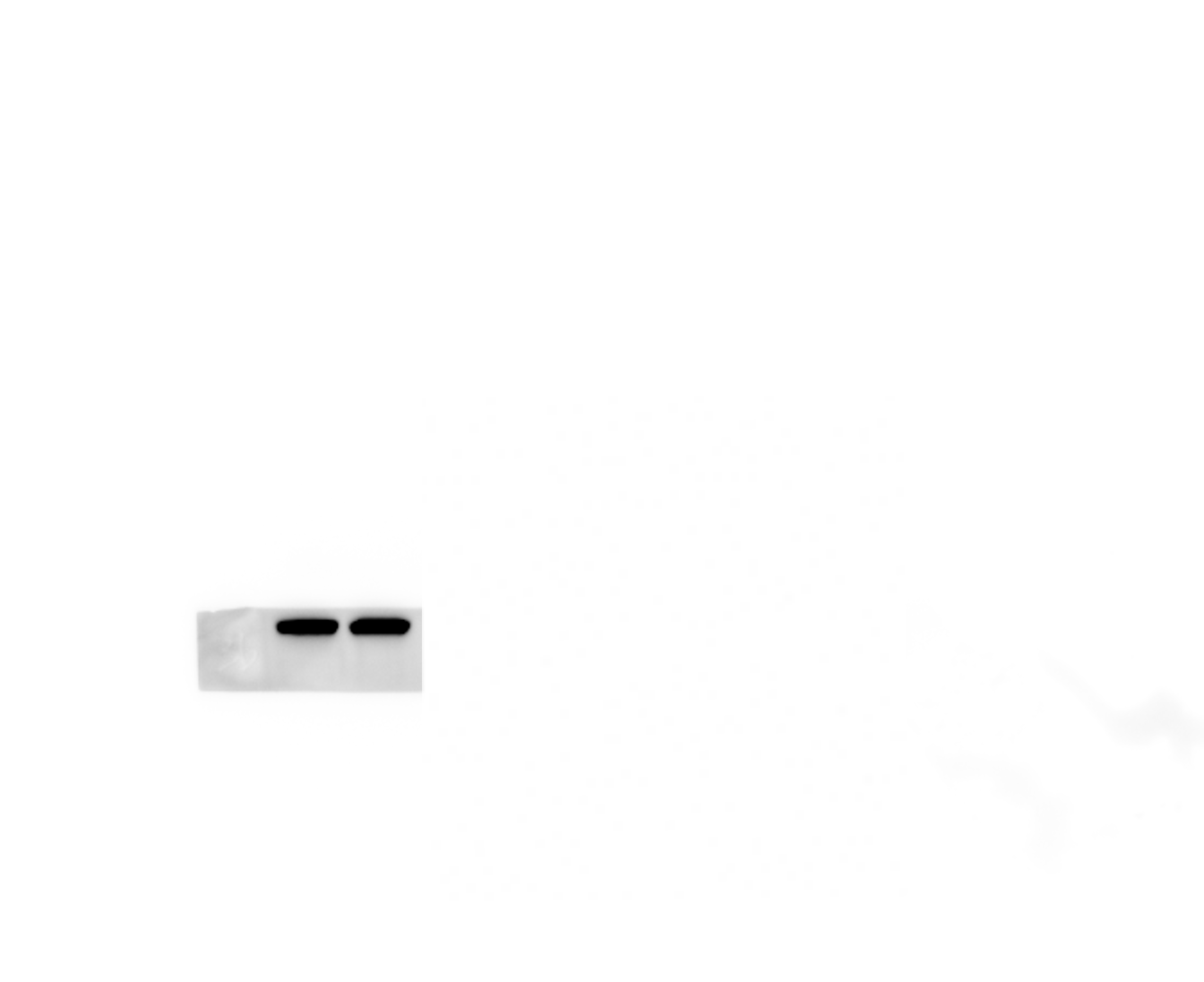

Supplement: Supplementary file 1 — Additional file 1. Raw data. [file 12935_2023_3076_MOESM1_ESM.zip › raw_data/figure7B1/gapdh/GAPDH 2∩╝êΓæóU2OS+si-NC∩╝¢ΓæúU2OS+si-DIO3OS∩╝ë.tif]

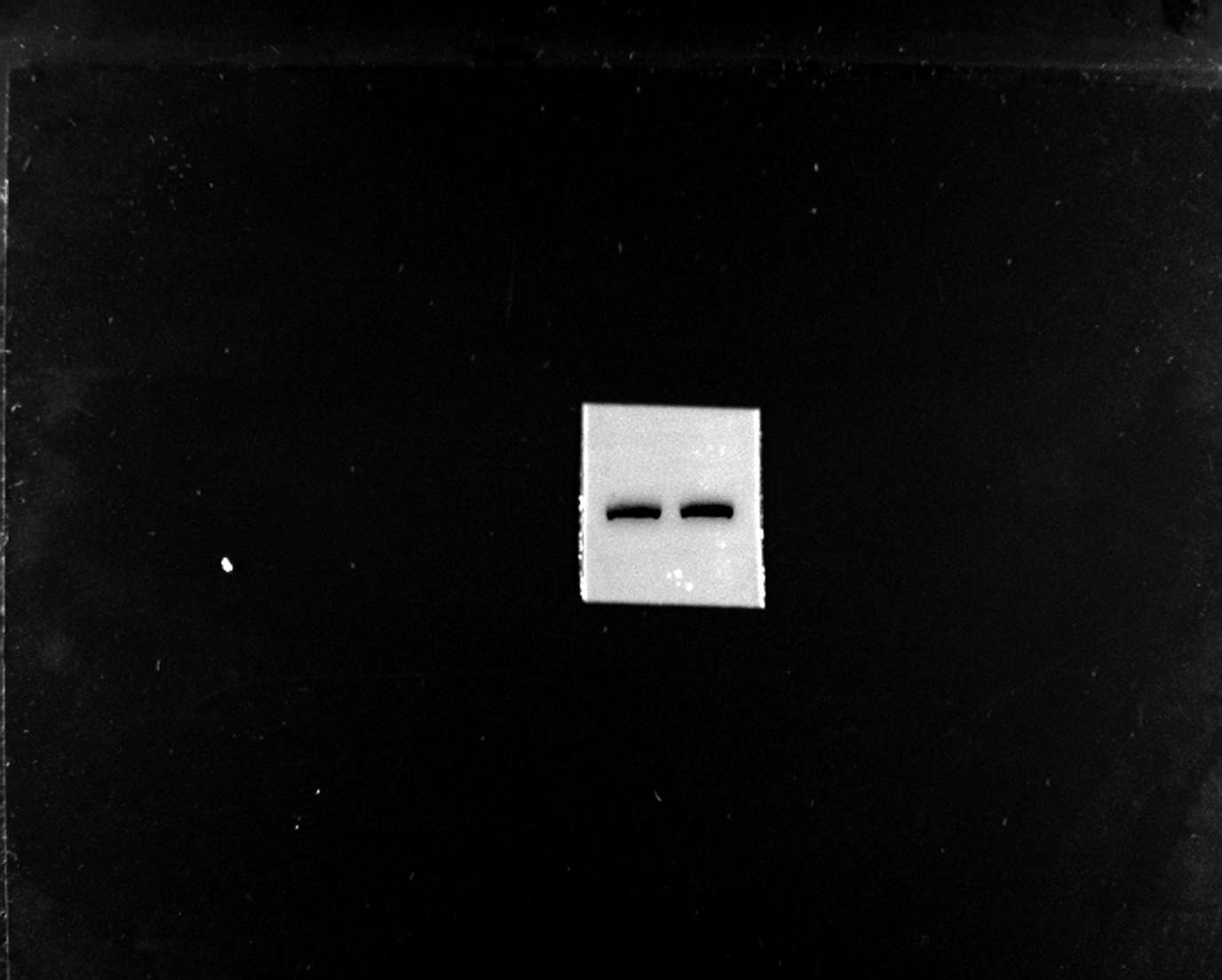

Supplement: Supplementary file 1 — Additional file 1. Raw data. [file 12935_2023_3076_MOESM1_ESM.zip › raw_data/figure7B1/gapdh/GAPDH 1τÖ╜σàë∩╝êΓæóU2OS+si-NC∩╝¢ΓæúU2OS+si-DIO3OS∩╝ë.tif]

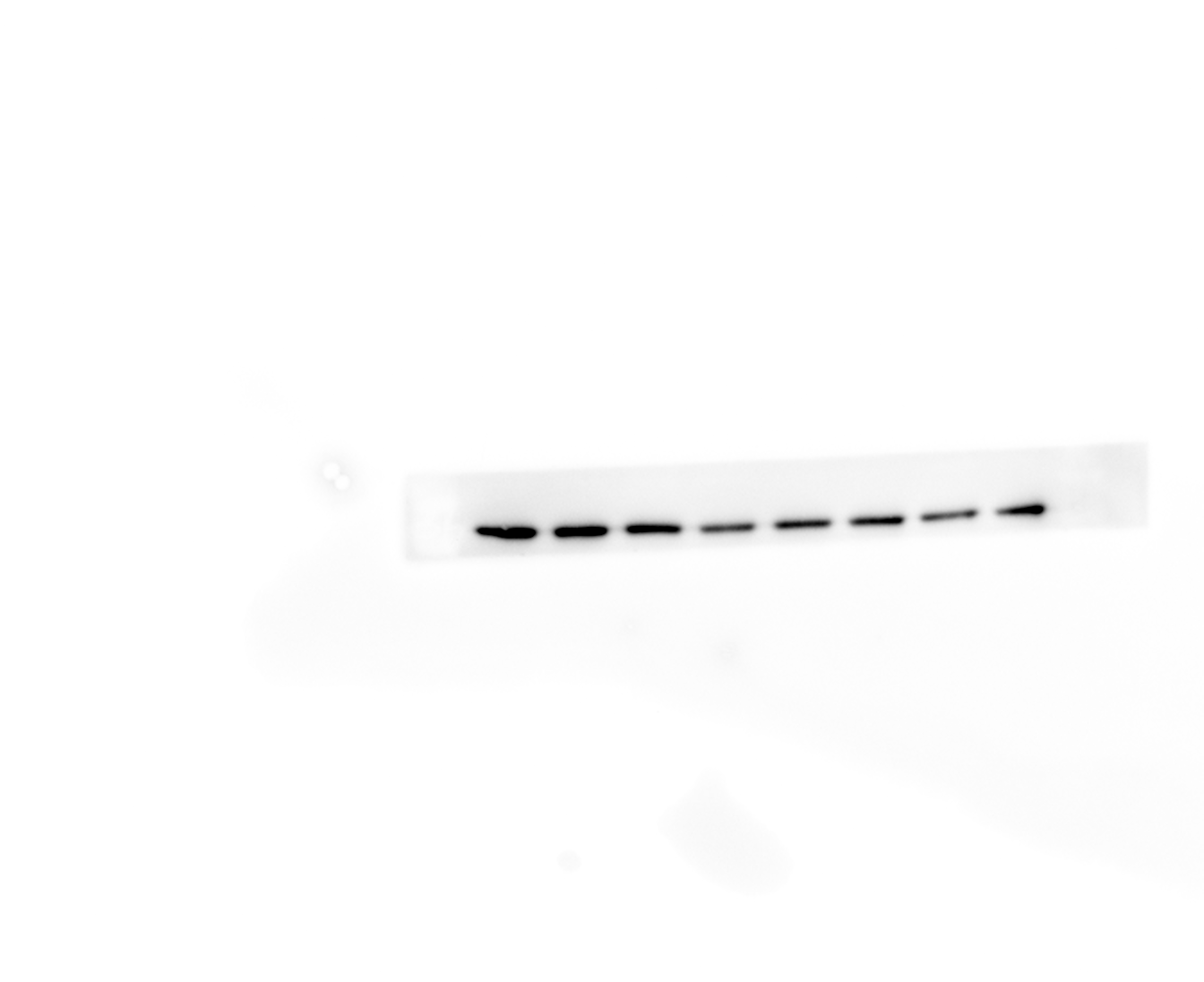

Supplement: Supplementary file 1 — Additional file 1. Raw data. [file 12935_2023_3076_MOESM1_ESM.zip › raw_data/figure7E/Figure.7 gapdh/GAPDH2(ΓæáSaoS-2+si-NC; ΓæíSaoS-2+si-DIO3OS; ΓæóSaoS-2+TGF-╬▓1+si-NC; ΓæúSaoS-2+TGF-╬▓1+si-DIO3OS).Tif]

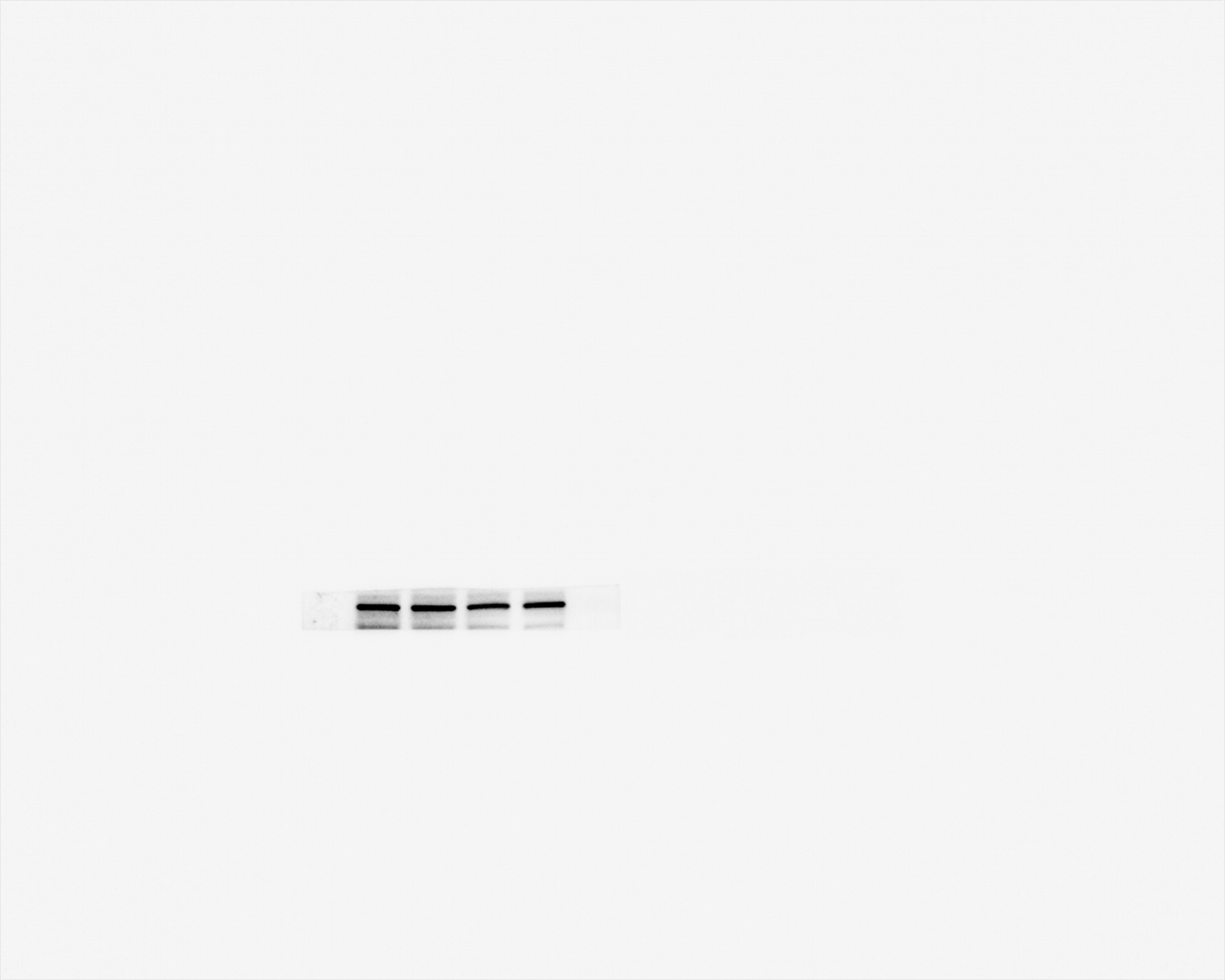

Supplement: Supplementary file 1 — Additional file 1. Raw data. [file 12935_2023_3076_MOESM1_ESM.zip › raw_data/figure7E/Figure.7 gapdh/GAPDH(ΓæáSaoS-2+si-NC; ΓæíSaoS-2+si-DIO3OS; ΓæóSaoS-2+TGF-╬▓1+si-NC; ΓæúSaoS-2+TGF-╬▓1+si-DIO3OS).tif]

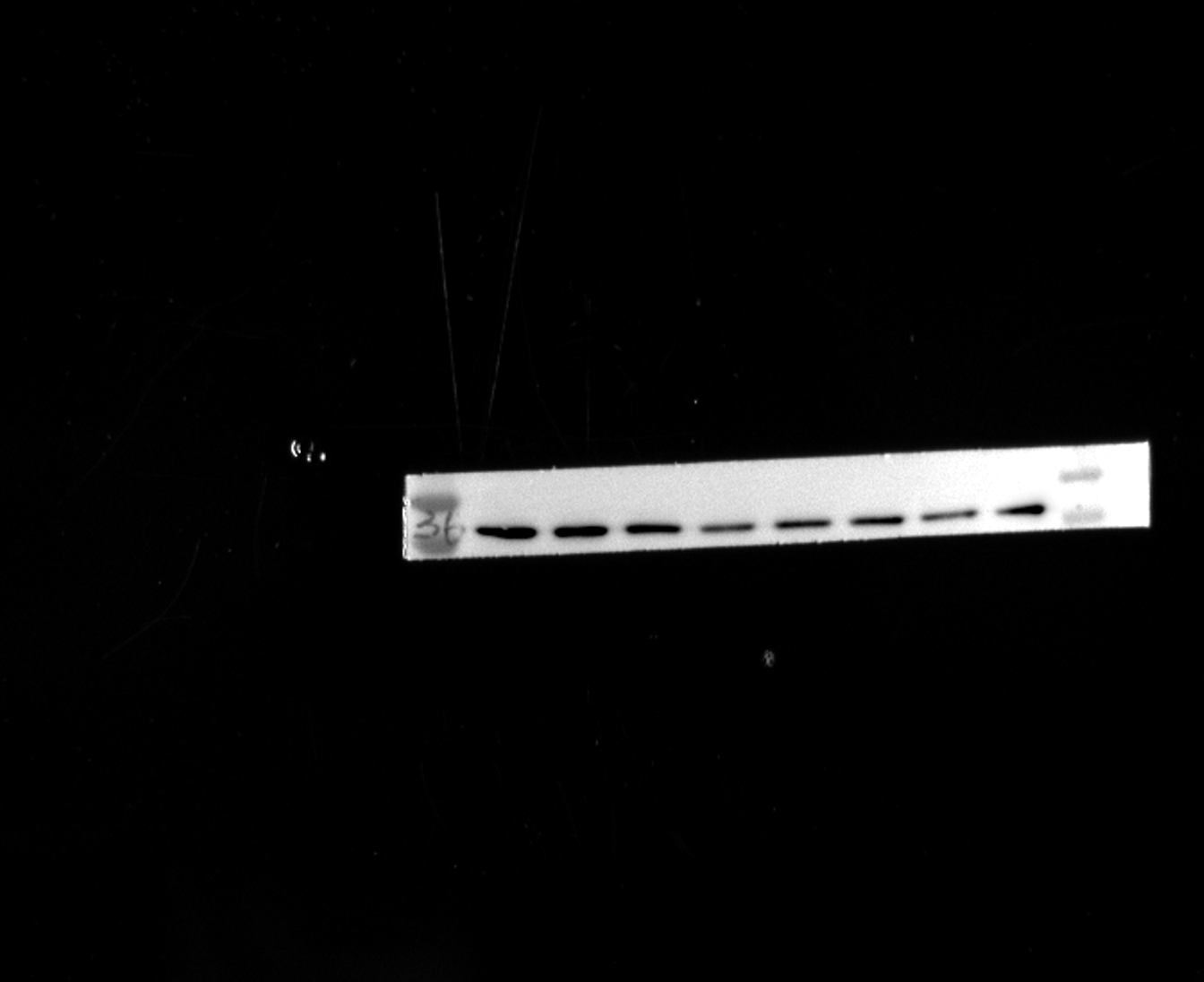

Supplement: Supplementary file 1 — Additional file 1. Raw data. [file 12935_2023_3076_MOESM1_ESM.zip › raw_data/figure7E/Figure.7 gapdh/GAPDH2τÖ╜σàë(ΓæáSaoS-2+si-NC; ΓæíSaoS-2+si-DIO3OS; ΓæóSaoS-2+TGF-╬▓1+si-NC; ΓæúSaoS-2+TGF-╬▓1+si-DIO3OS).Tif]

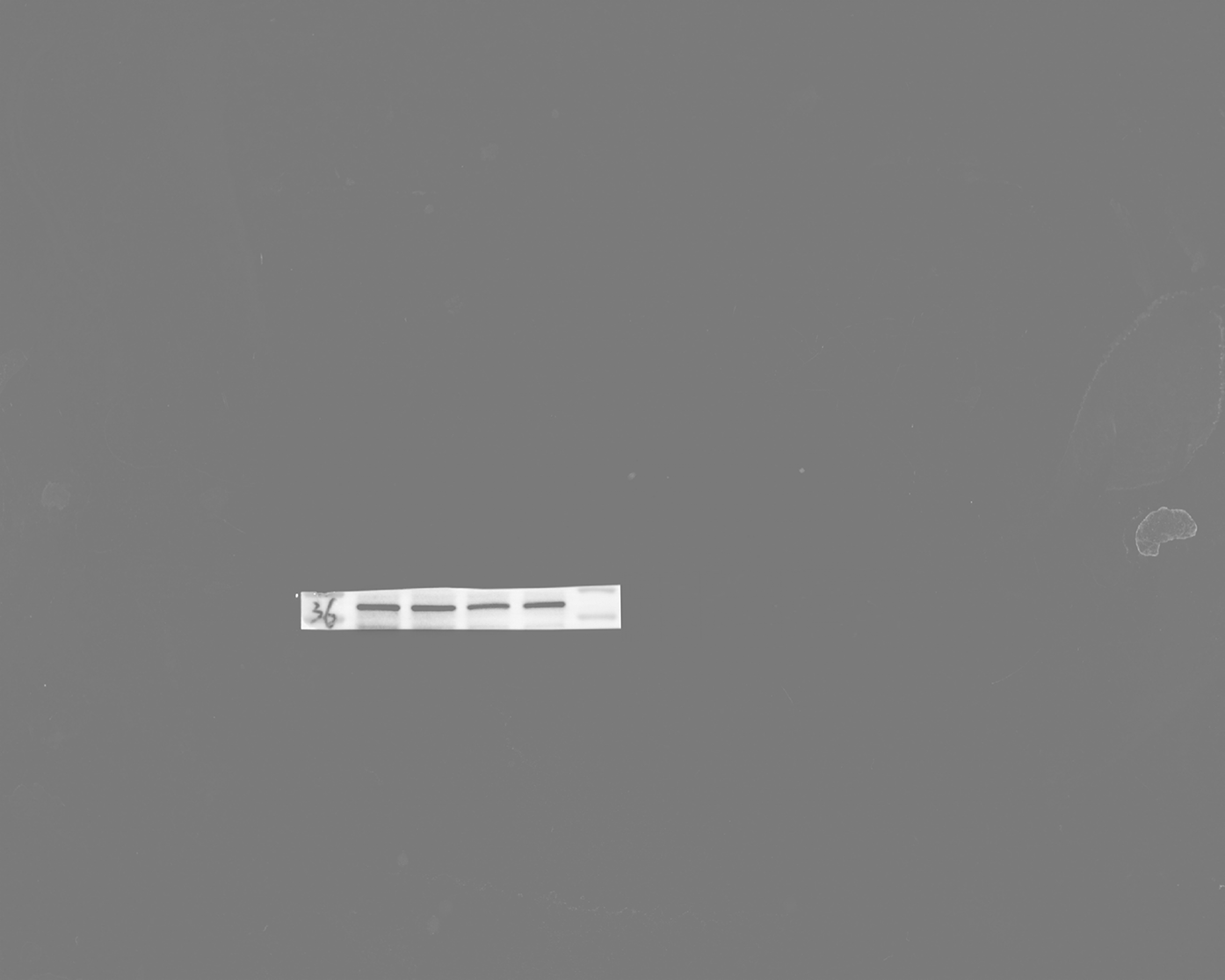

Supplement: Supplementary file 1 — Additional file 1. Raw data. [file 12935_2023_3076_MOESM1_ESM.zip › raw_data/figure7E/Figure.7 gapdh/GAPDHτÖ╜σàë(ΓæáSaoS-2+si-NC; ΓæíSaoS-2+si-DIO3OS; ΓæóSaoS-2+TGF-╬▓1+si-NC; ΓæúSaoS-2+TGF-╬▓1+si-DIO3OS).tif]

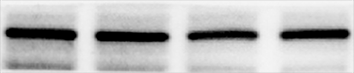

Supplement: Supplementary file 1 — Additional file 1. Raw data. [file 12935_2023_3076_MOESM1_ESM.zip › raw_data/figure7E/Figure.7 gapdh/gapdh.tif]

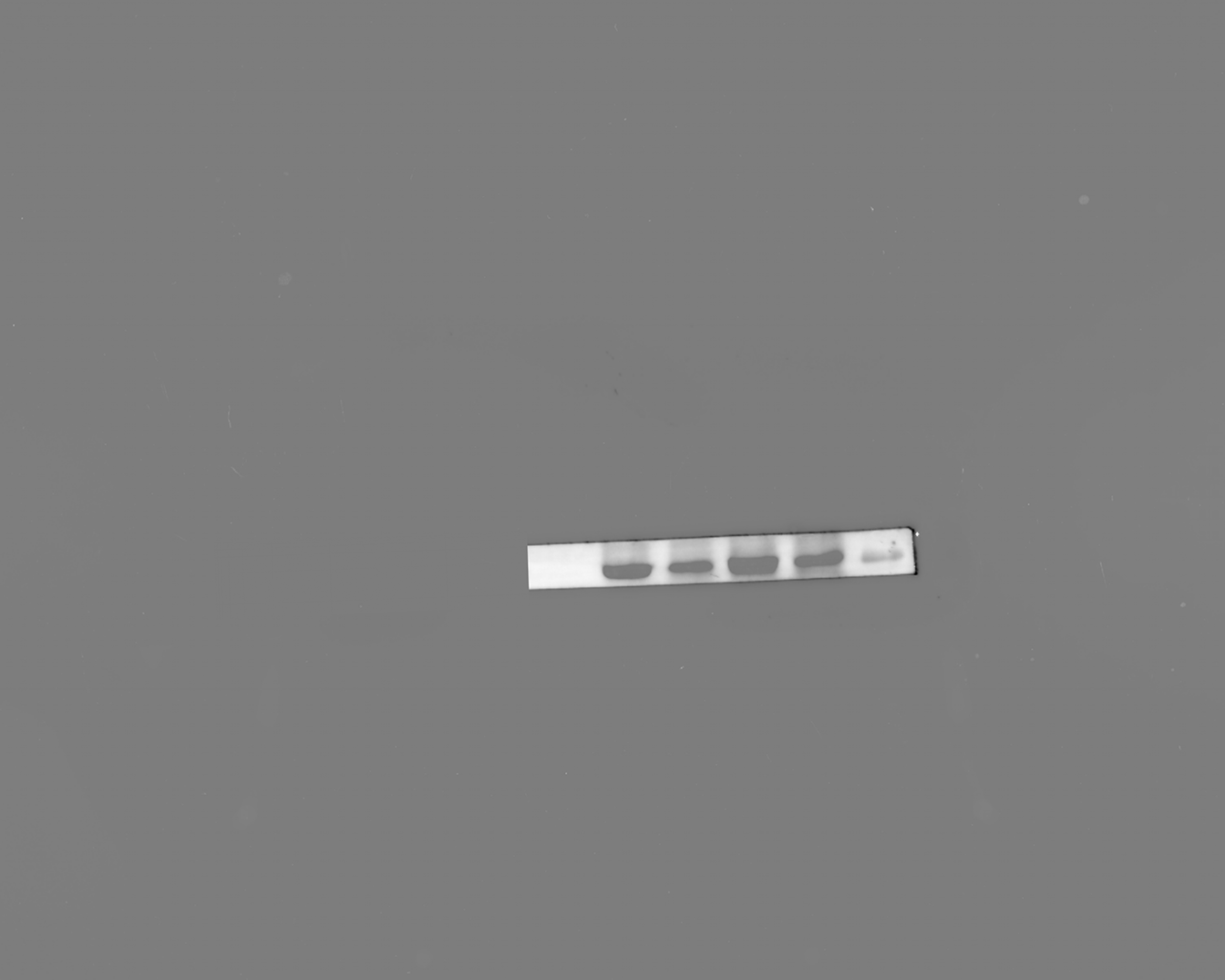

Supplement: Supplementary file 1 — Additional file 1. Raw data. [file 12935_2023_3076_MOESM1_ESM.zip › raw_data/figure7E/Figure.7 Vimentin/VimentinτÖ╜σàë(ΓæáSaoS-2+si-NC; ΓæíSaoS-2+si-DIO3OS; ΓæóSaoS-2+TGF-╬▓1+si-NC; ΓæúSaoS-2+TGF-╬▓1+si-DIO3OS).tif]

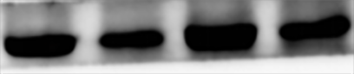

Supplement: Supplementary file 1 — Additional file 1. Raw data. [file 12935_2023_3076_MOESM1_ESM.zip › raw_data/figure7E/Figure.7 Vimentin/Vimentin.tif]

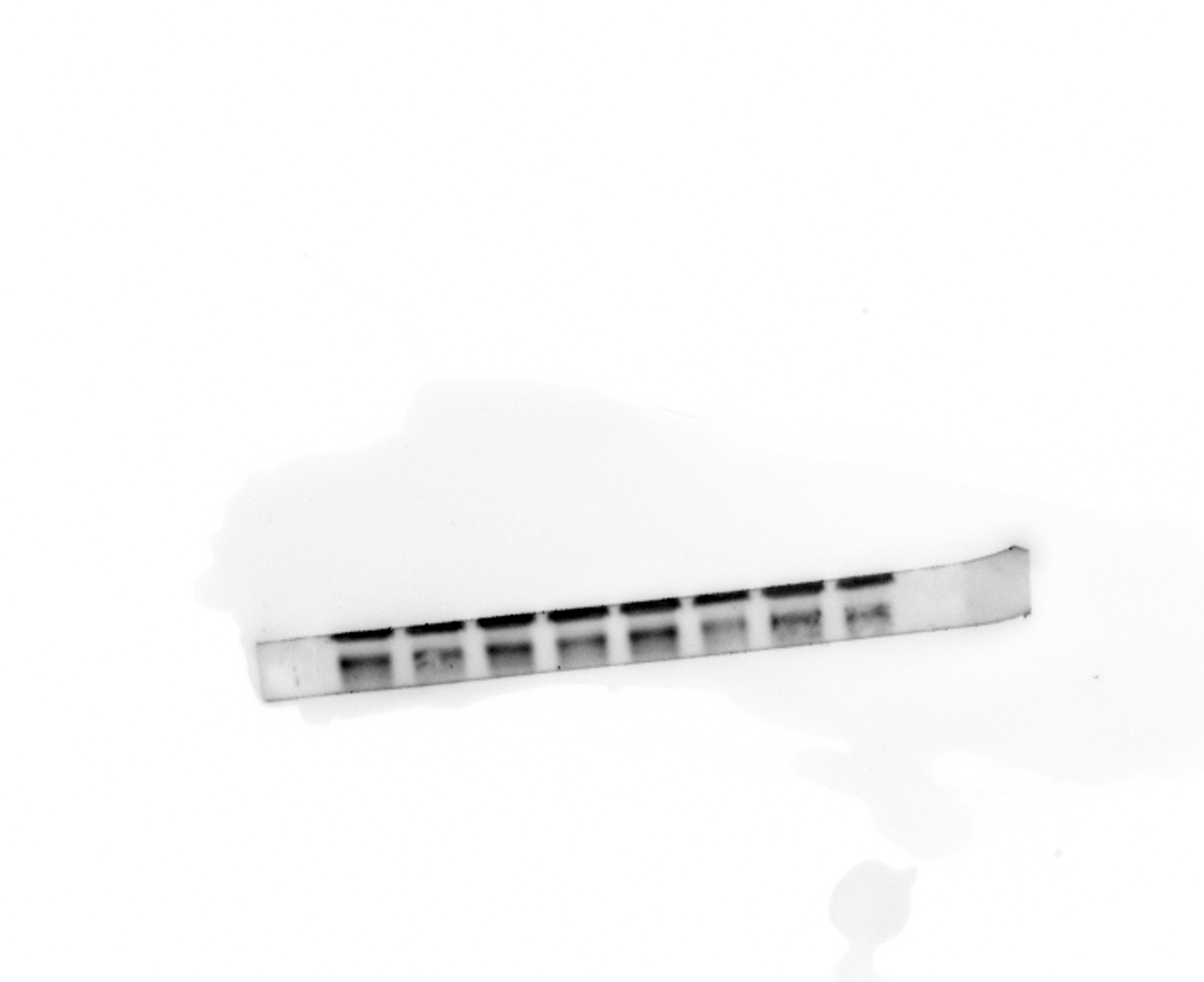

Supplement: Supplementary file 1 — Additional file 1. Raw data. [file 12935_2023_3076_MOESM1_ESM.zip › raw_data/figure7E/Figure.7 Vimentin/Vimentin2(ΓæáSaoS-2+si-NC; ΓæíSaoS-2+si-DIO3OS; ΓæóSaoS-2+TGF-╬▓1+si-NC; ΓæúSaoS-2+TGF-╬▓1+si-DIO3OS).Tif]

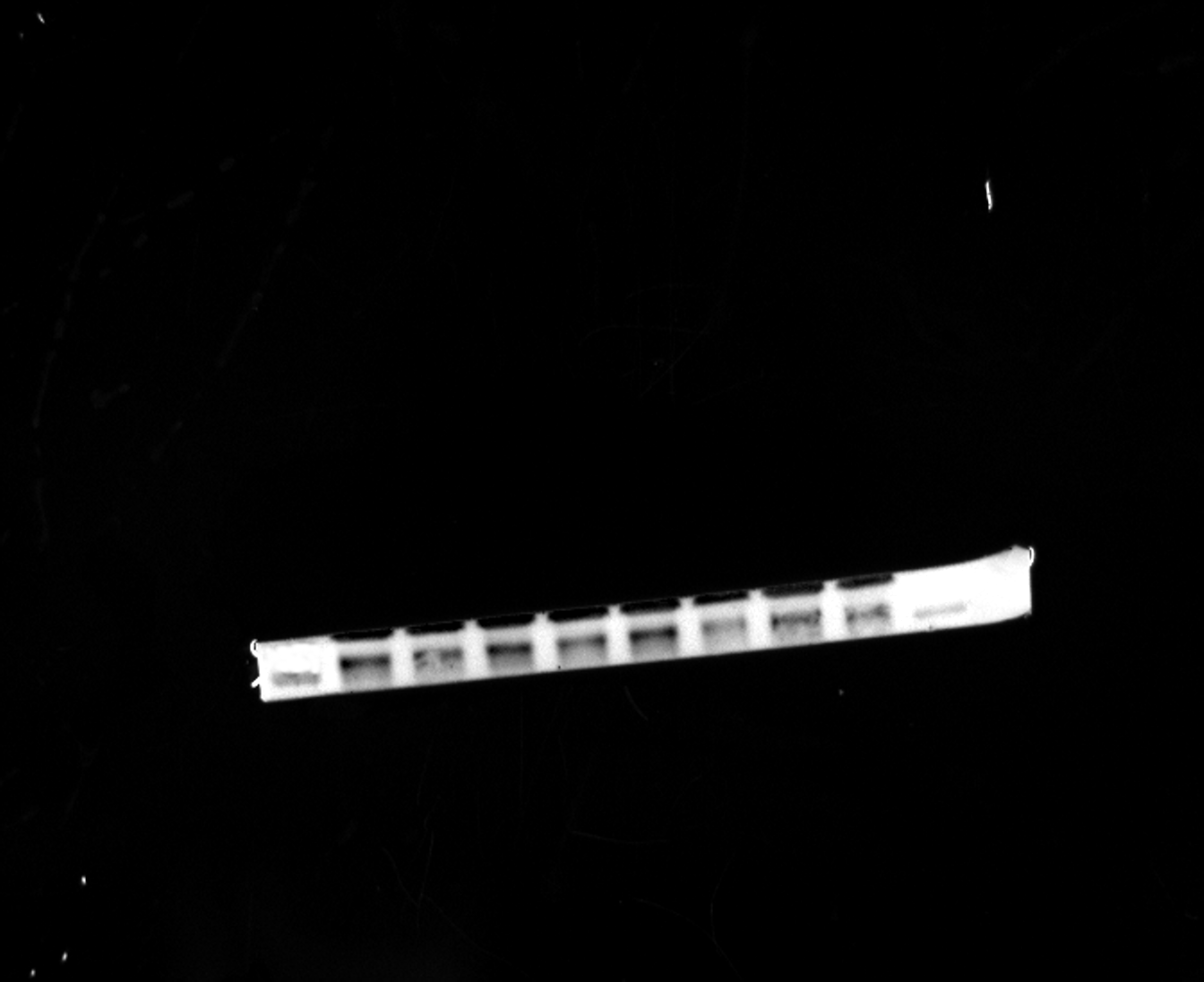

Supplement: Supplementary file 1 — Additional file 1. Raw data. [file 12935_2023_3076_MOESM1_ESM.zip › raw_data/figure7E/Figure.7 Vimentin/Vimentin2τÖ╜σàë(ΓæáSaoS-2+si-NC; ΓæíSaoS-2+si-DIO3OS; ΓæóSaoS-2+TGF-╬▓1+si-NC; ΓæúSaoS-2+TGF-╬▓1+si-DIO3OS).Tif]

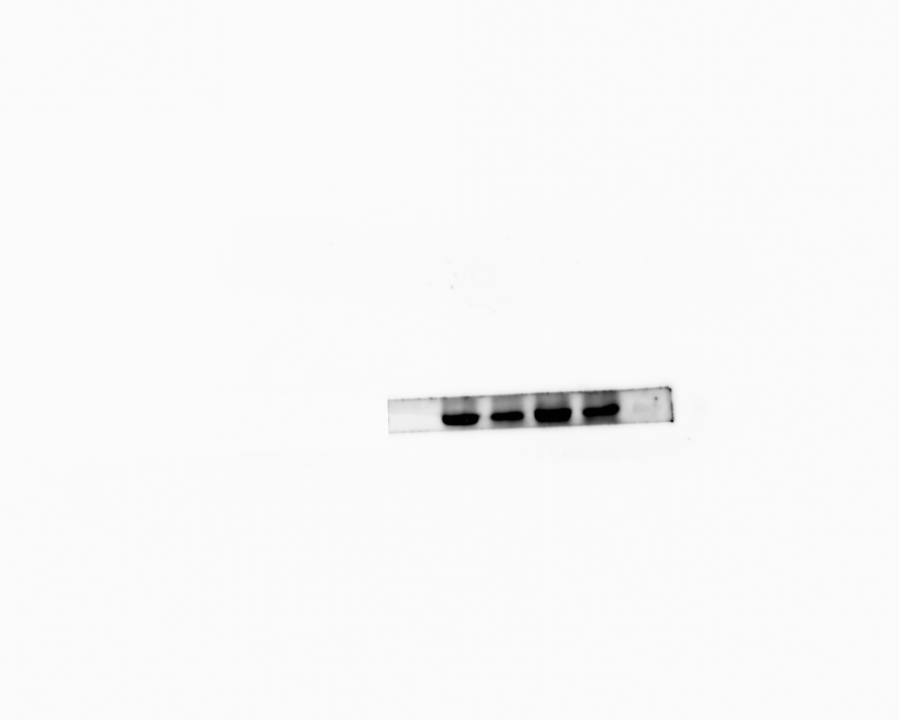

Supplement: Supplementary file 1 — Additional file 1. Raw data. [file 12935_2023_3076_MOESM1_ESM.zip › raw_data/figure7E/Figure.7 Vimentin/Vimentin(ΓæáSaoS-2+si-NC; ΓæíSaoS-2+si-DIO3OS; ΓæóSaoS-2+TGF-╬▓1+si-NC; ΓæúSaoS-2+TGF-╬▓1+si-DIO3OS).tif]

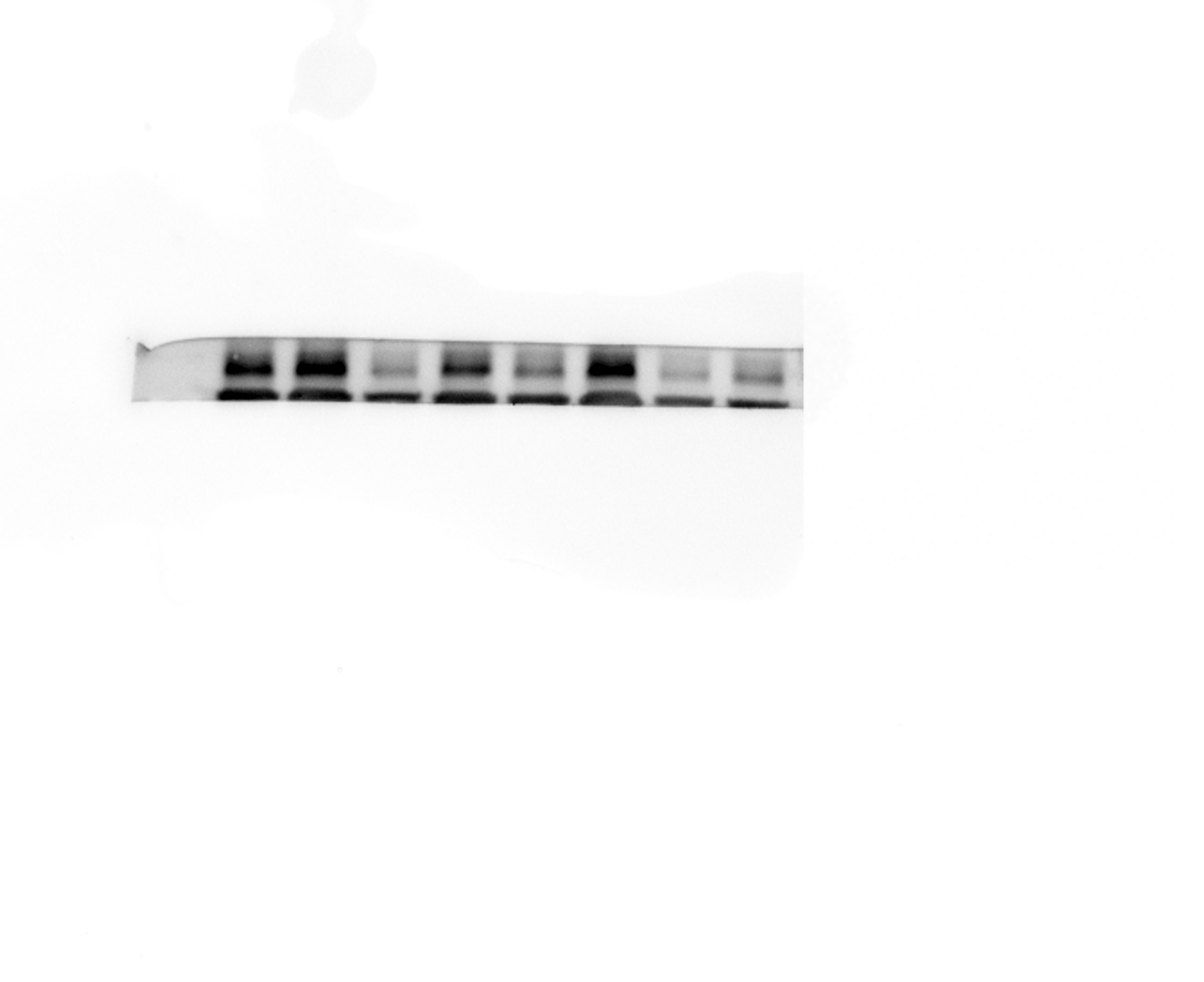

Supplement: Supplementary file 1 — Additional file 1. Raw data. [file 12935_2023_3076_MOESM1_ESM.zip › raw_data/figure7E/Figure.7 E-cad/E-cad2(ΓæáSaoS-2+si-NC; ΓæíSaoS-2+si-DIO3OS; ΓæóSaoS-2+TGF-╬▓1+si-NC; ΓæúSaoS-2+TGF-╬▓1+si-DIO3OS).Tif]

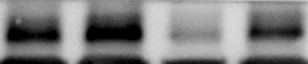

Supplement: Supplementary file 1 — Additional file 1. Raw data. [file 12935_2023_3076_MOESM1_ESM.zip › raw_data/figure7E/Figure.7 E-cad/E-cad.tif]

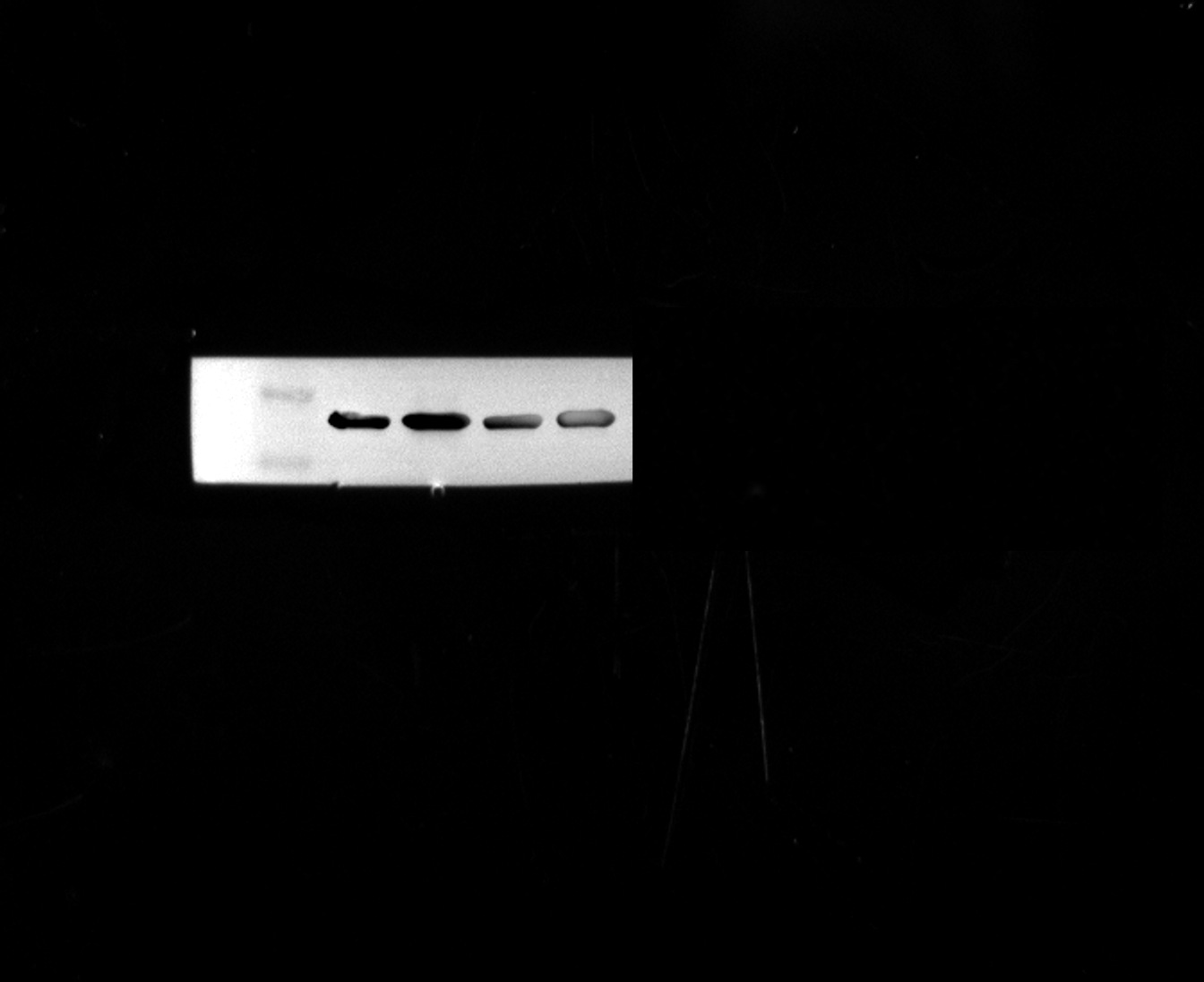

Supplement: Supplementary file 1 — Additional file 1. Raw data. [file 12935_2023_3076_MOESM1_ESM.zip › raw_data/figure7E/Figure.7 E-cad/E-cadτÖ╜σàë(ΓæáSaoS-2+si-NC; ΓæíSaoS-2+si-DIO3OS; ΓæóSaoS-2+TGF-╬▓1+si-NC; ΓæúSaoS-2+TGF-╬▓1+si-DIO3OS).Tif]

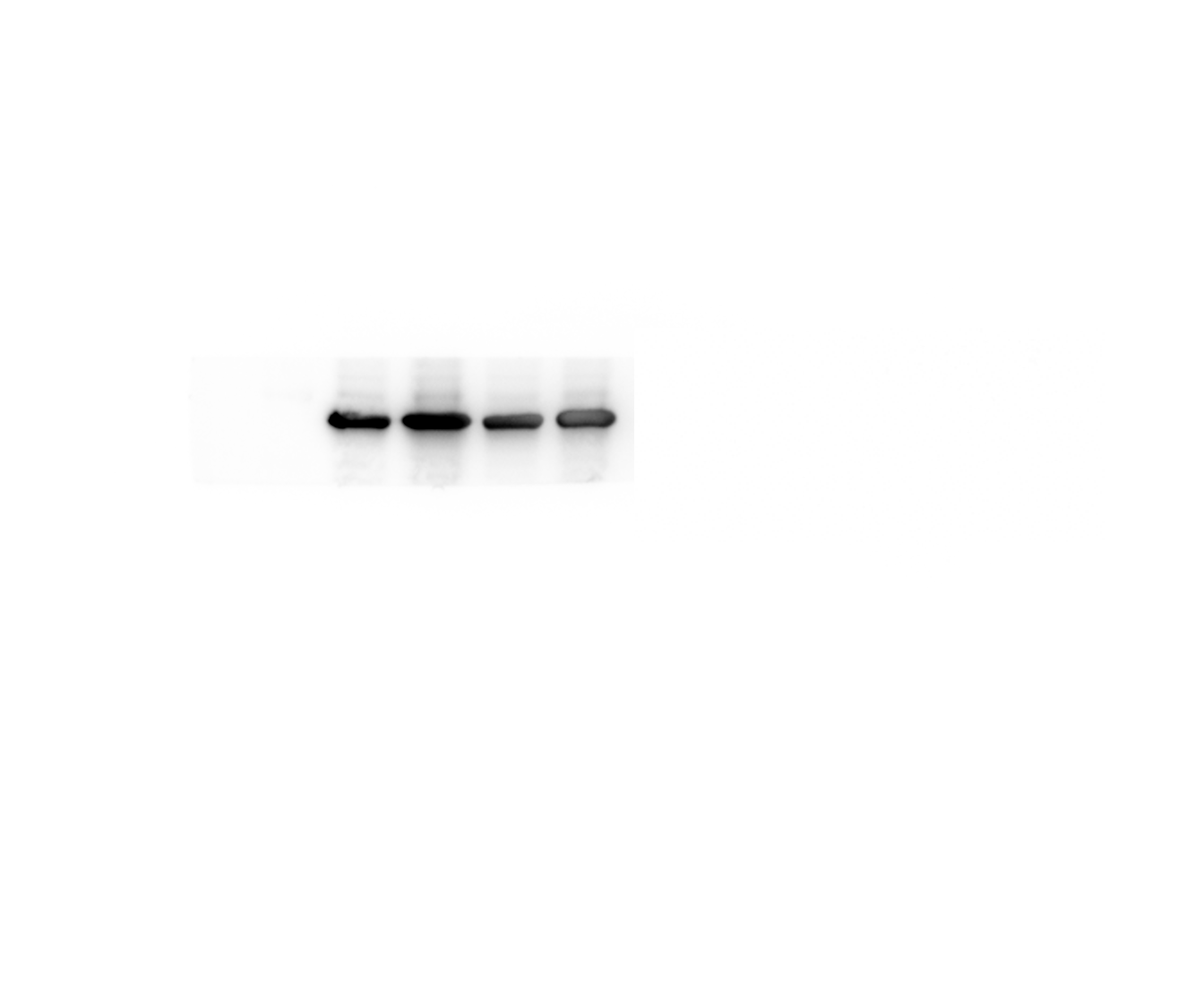

Supplement: Supplementary file 1 — Additional file 1. Raw data. [file 12935_2023_3076_MOESM1_ESM.zip › raw_data/figure7E/Figure.7 E-cad/E-cad(ΓæáSaoS-2+si-NC; ΓæíSaoS-2+si-DIO3OS; ΓæóSaoS-2+TGF-╬▓1+si-NC; ΓæúSaoS-2+TGF-╬▓1+si-DIO3OS).Tif]

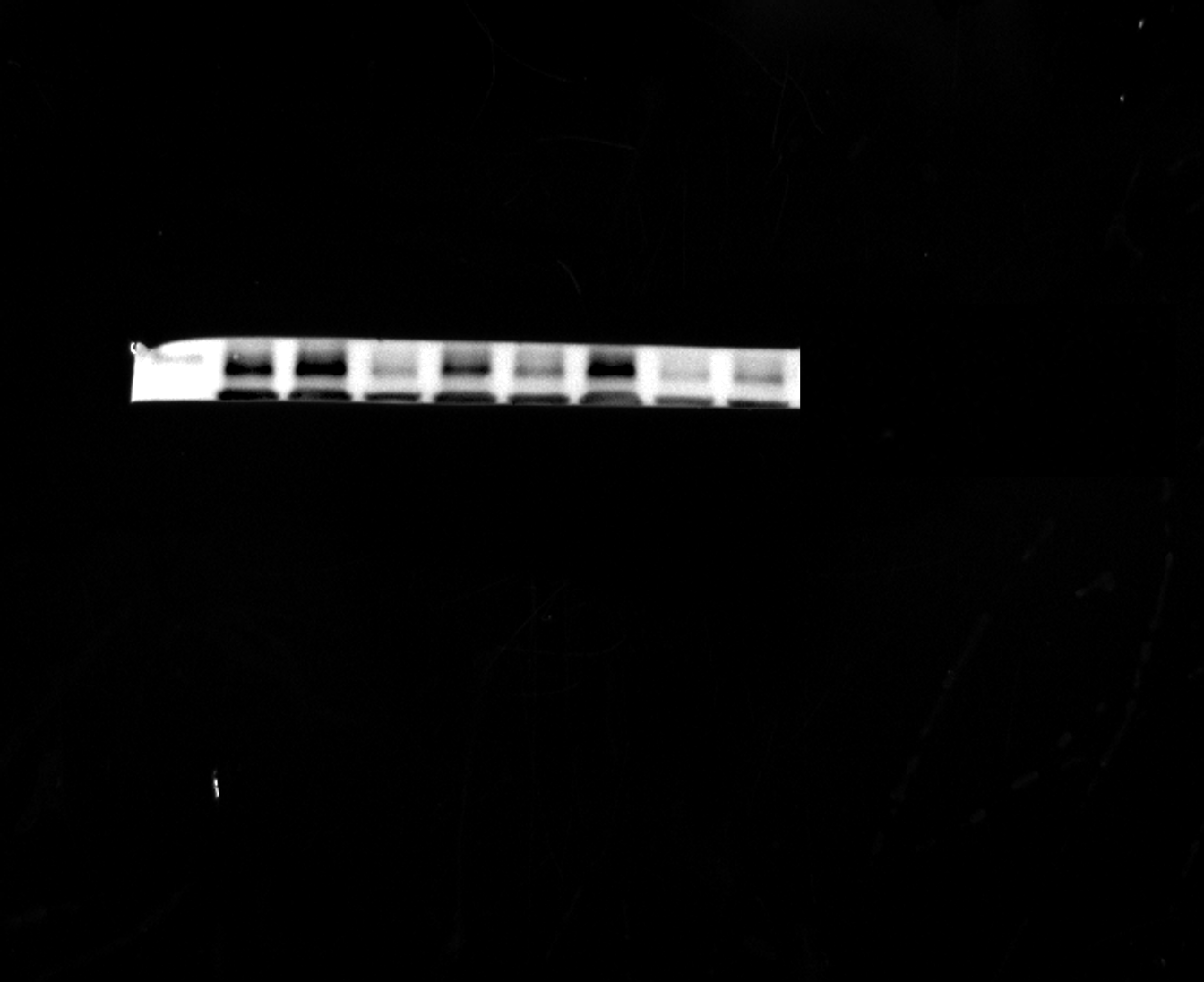

Supplement: Supplementary file 1 — Additional file 1. Raw data. [file 12935_2023_3076_MOESM1_ESM.zip › raw_data/figure7E/Figure.7 E-cad/E-cad2τÖ╜σàë(ΓæáSaoS-2+si-NC; ΓæíSaoS-2+si-DIO3OS; ΓæóSaoS-2+TGF-╬▓1+si-NC; ΓæúSaoS-2+TGF-╬▓1+si-DIO3OS).Tif]

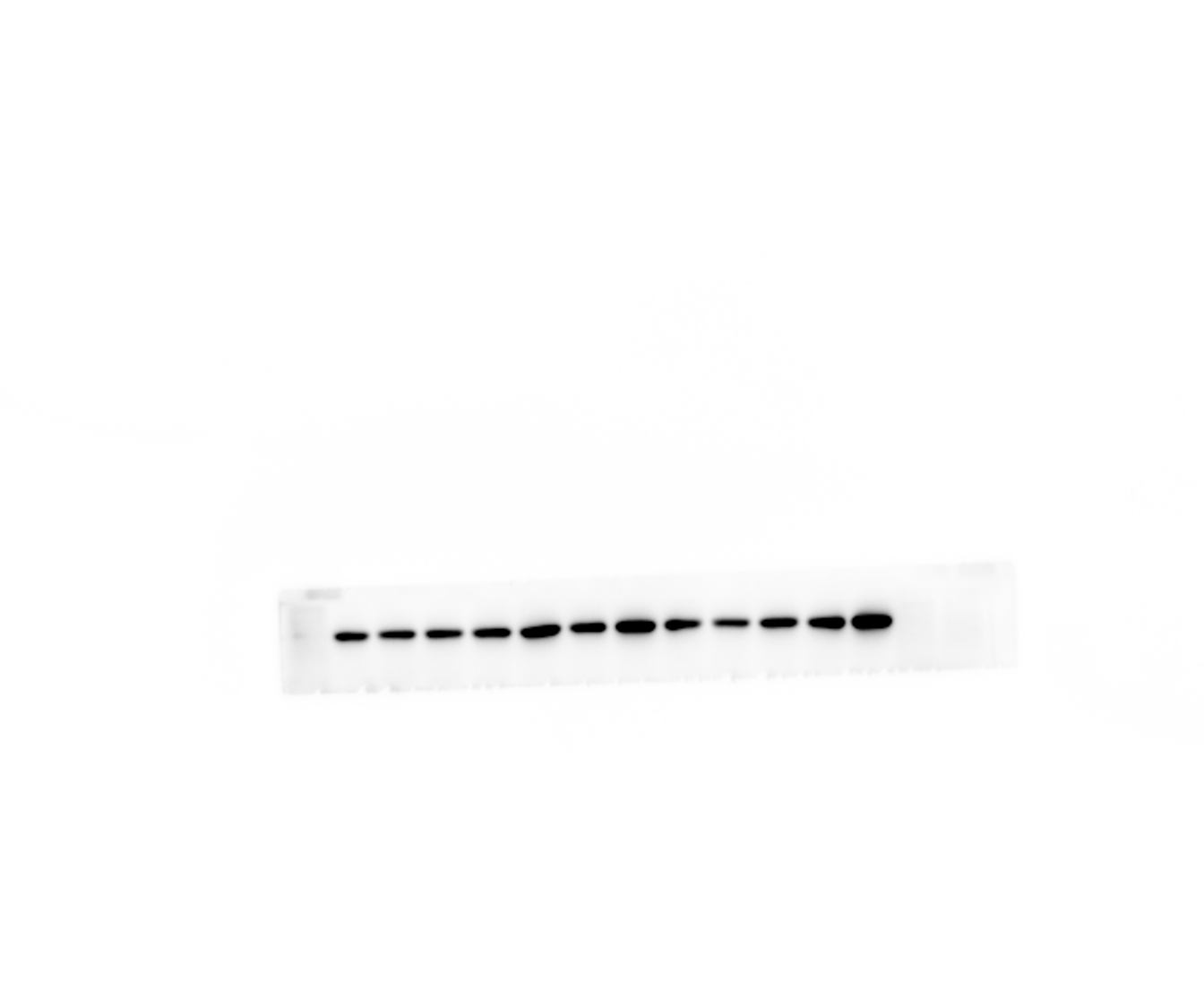

Supplement: Supplementary file 1 — Additional file 1. Raw data. [file 12935_2023_3076_MOESM1_ESM.zip › raw_data/figure8-h1/SMAD2/SMAD2 31-6∩╝ÜSaoS-2+si-NC∩╝¢7-12∩╝ÜSaoS-2+si-DIO3OS∩╝ë.tif.Tif]

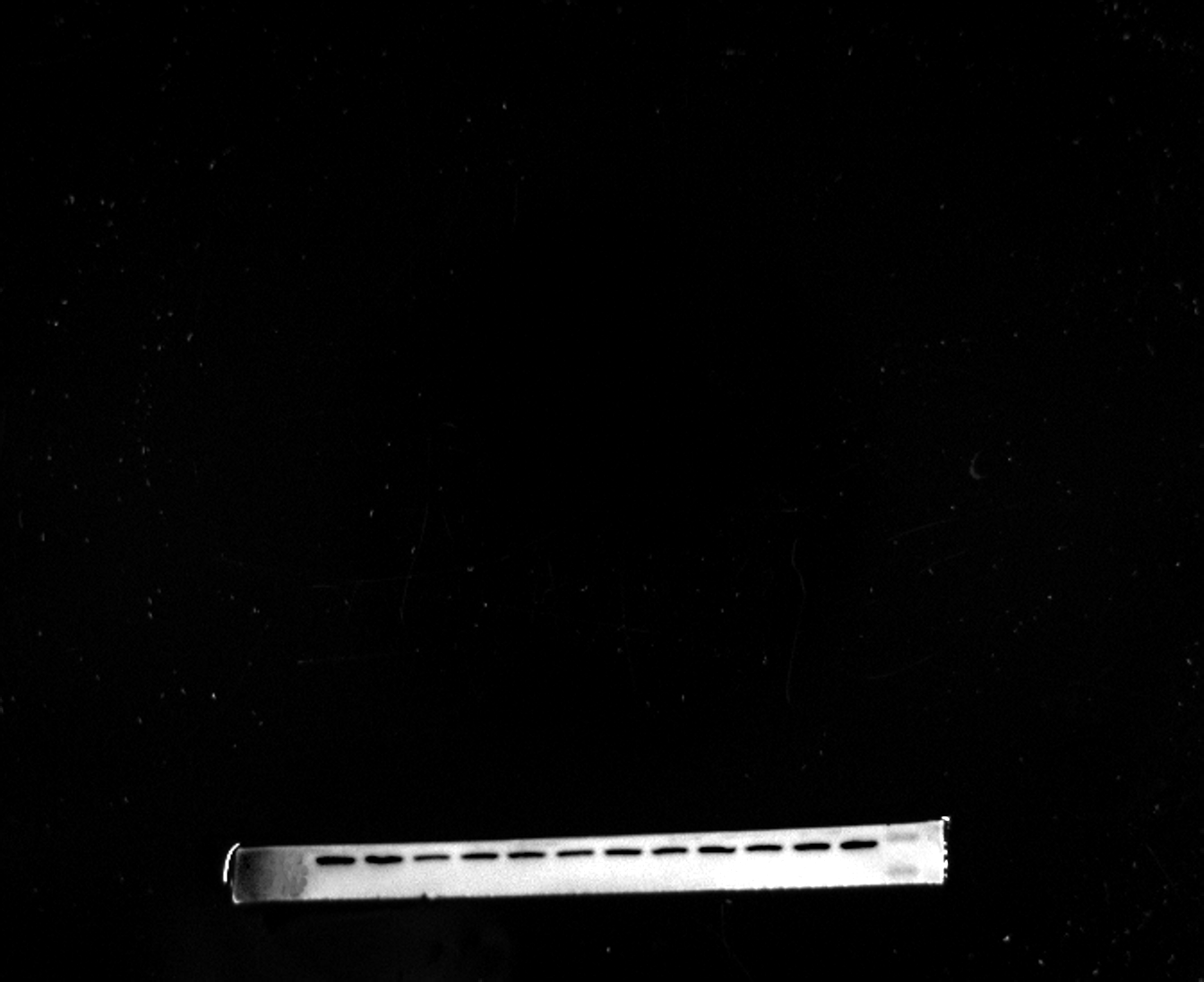

Supplement: Supplementary file 1 — Additional file 1. Raw data. [file 12935_2023_3076_MOESM1_ESM.zip › raw_data/figure8-h1/SMAD2/SMAD2 2τÖ╜σàë1-6∩╝ÜSaoS-2+si-NC∩╝¢7-12∩╝ÜSaoS-2+si-DIO3OS∩╝ë.tif.Tif]

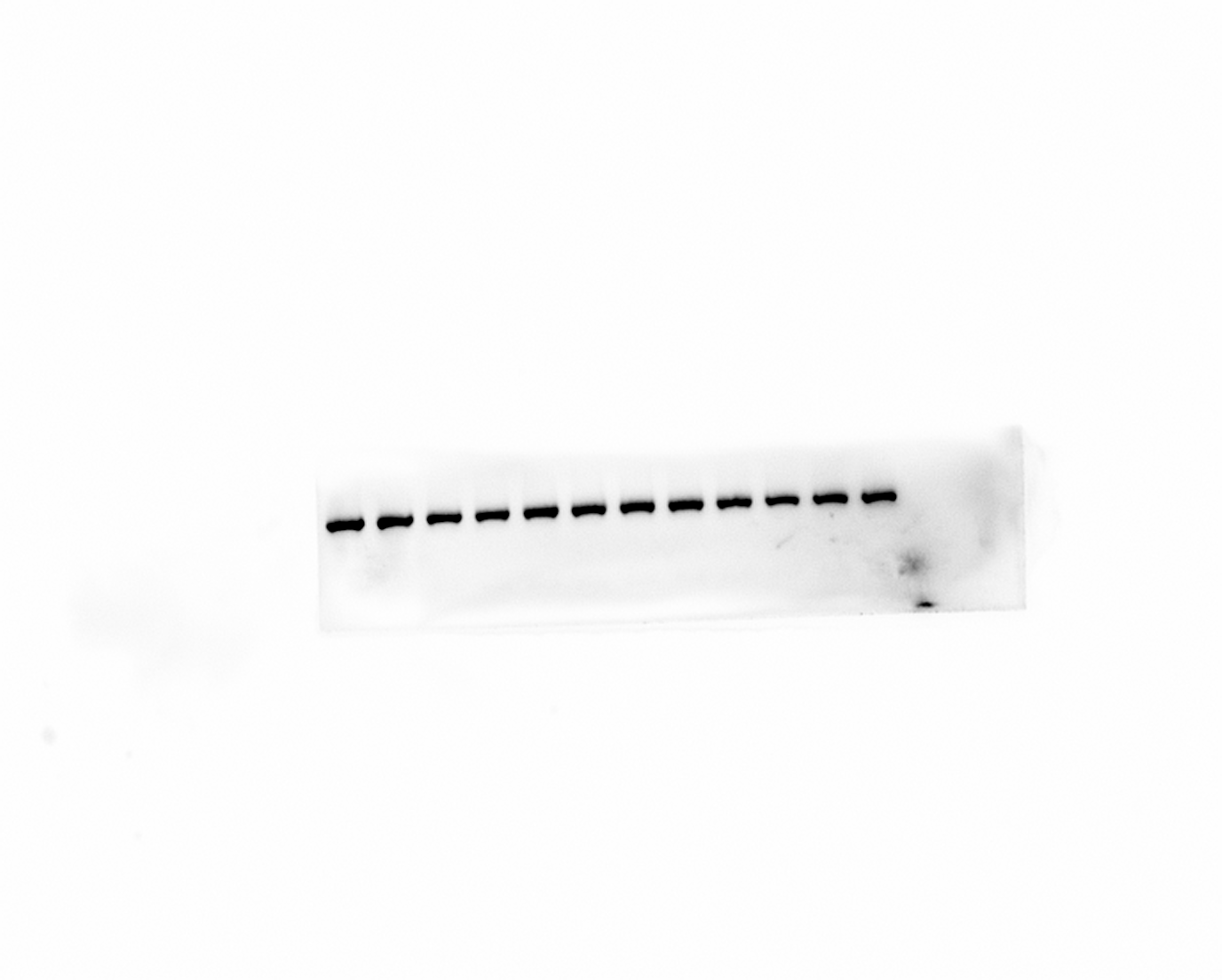

Supplement: Supplementary file 1 — Additional file 1. Raw data. [file 12935_2023_3076_MOESM1_ESM.zip › raw_data/figure8-h1/SMAD2/SMAD2 1∩╝ê1-6∩╝ÜSaoS-2+si-NC∩╝¢7-12∩╝ÜSaoS-2+si-DIO3OS∩╝ë.tif]

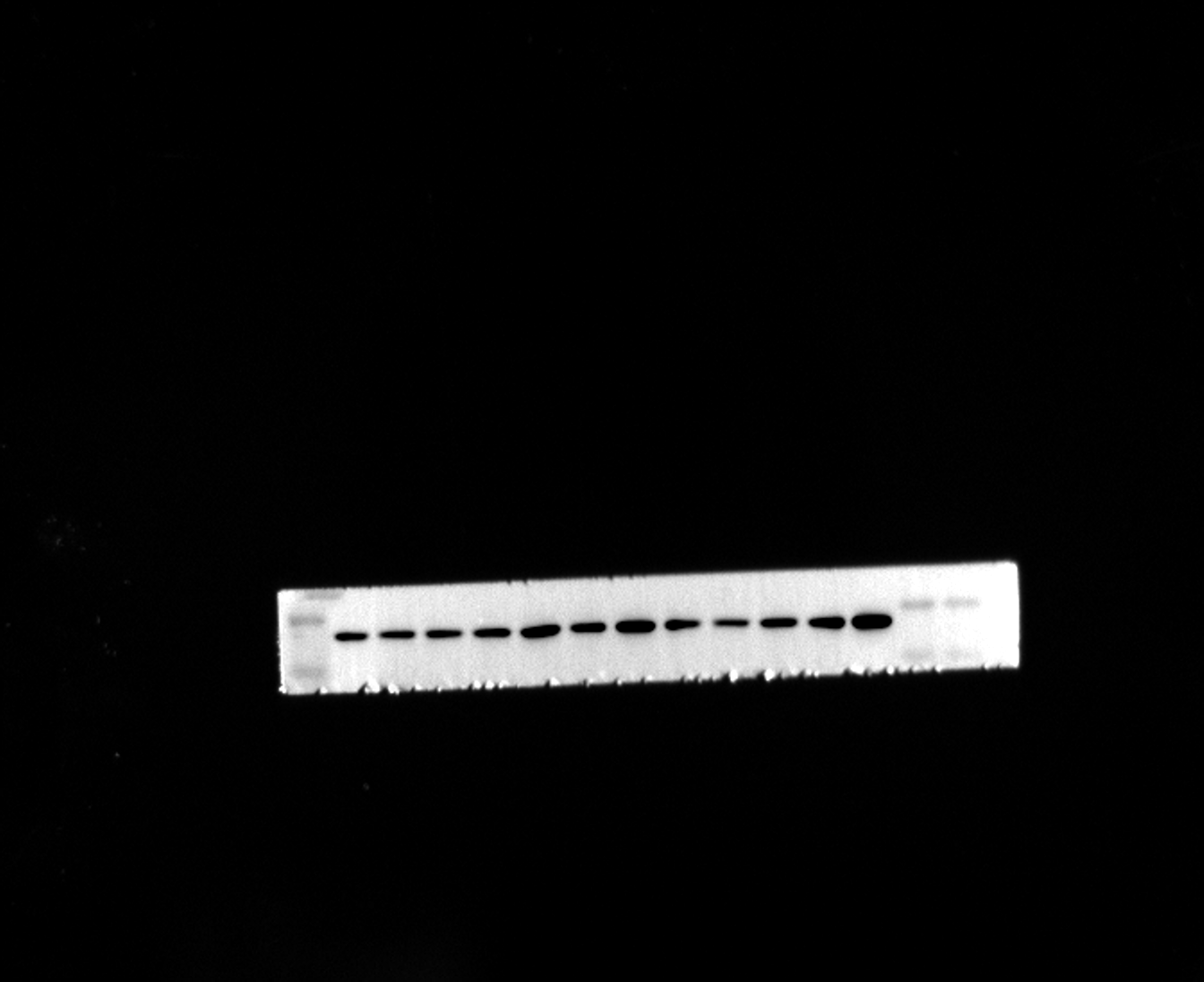

Supplement: Supplementary file 1 — Additional file 1. Raw data. [file 12935_2023_3076_MOESM1_ESM.zip › raw_data/figure8-h1/SMAD2/SMAD2 3τÖ╜σàë1-6∩╝ÜSaoS-2+si-NC∩╝¢7-12∩╝ÜSaoS-2+si-DIO3OS∩╝ë.tif.Tif]

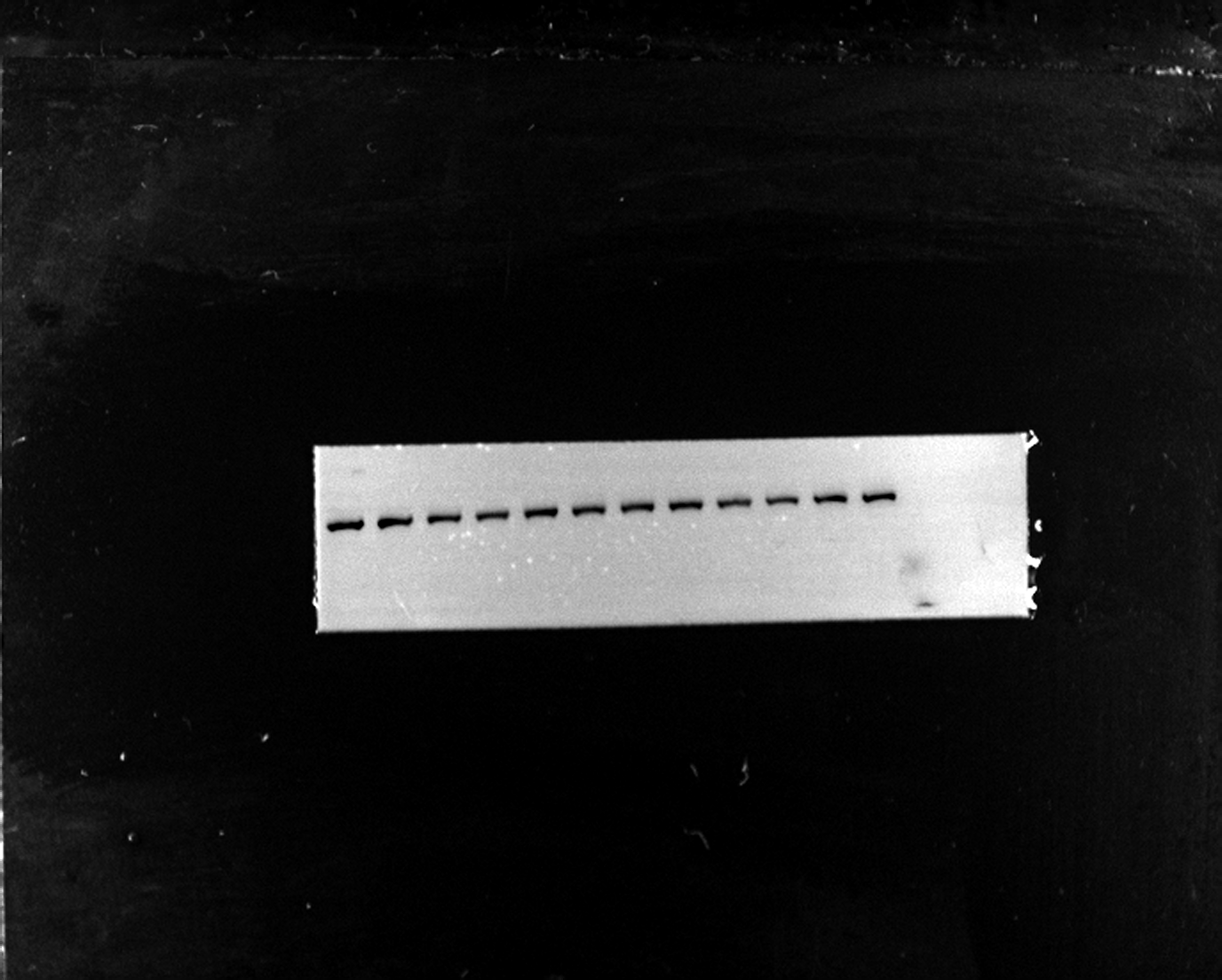

Supplement: Supplementary file 1 — Additional file 1. Raw data. [file 12935_2023_3076_MOESM1_ESM.zip › raw_data/figure8-h1/SMAD2/SMAD2 1τÖ╜σàë1-6∩╝ÜSaoS-2+si-NC∩╝¢7-12∩╝ÜSaoS-2+si-DIO3OS∩╝ë.tif]

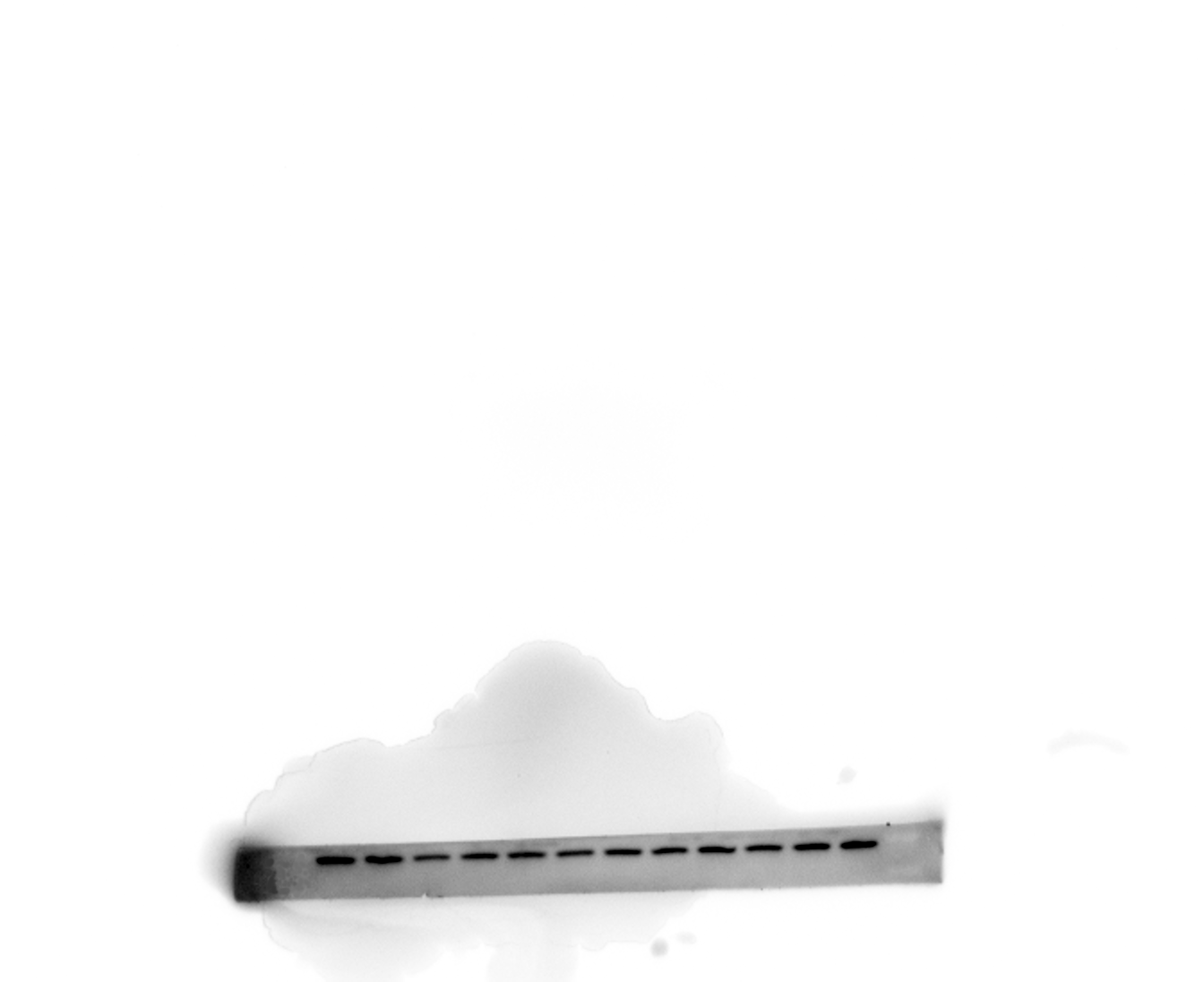

Supplement: Supplementary file 1 — Additional file 1. Raw data. [file 12935_2023_3076_MOESM1_ESM.zip › raw_data/figure8-h1/SMAD2/SMAD2 21-6∩╝ÜSaoS-2+si-NC∩╝¢7-12∩╝ÜSaoS-2+si-DIO3OS∩╝ë.tif.Tif]

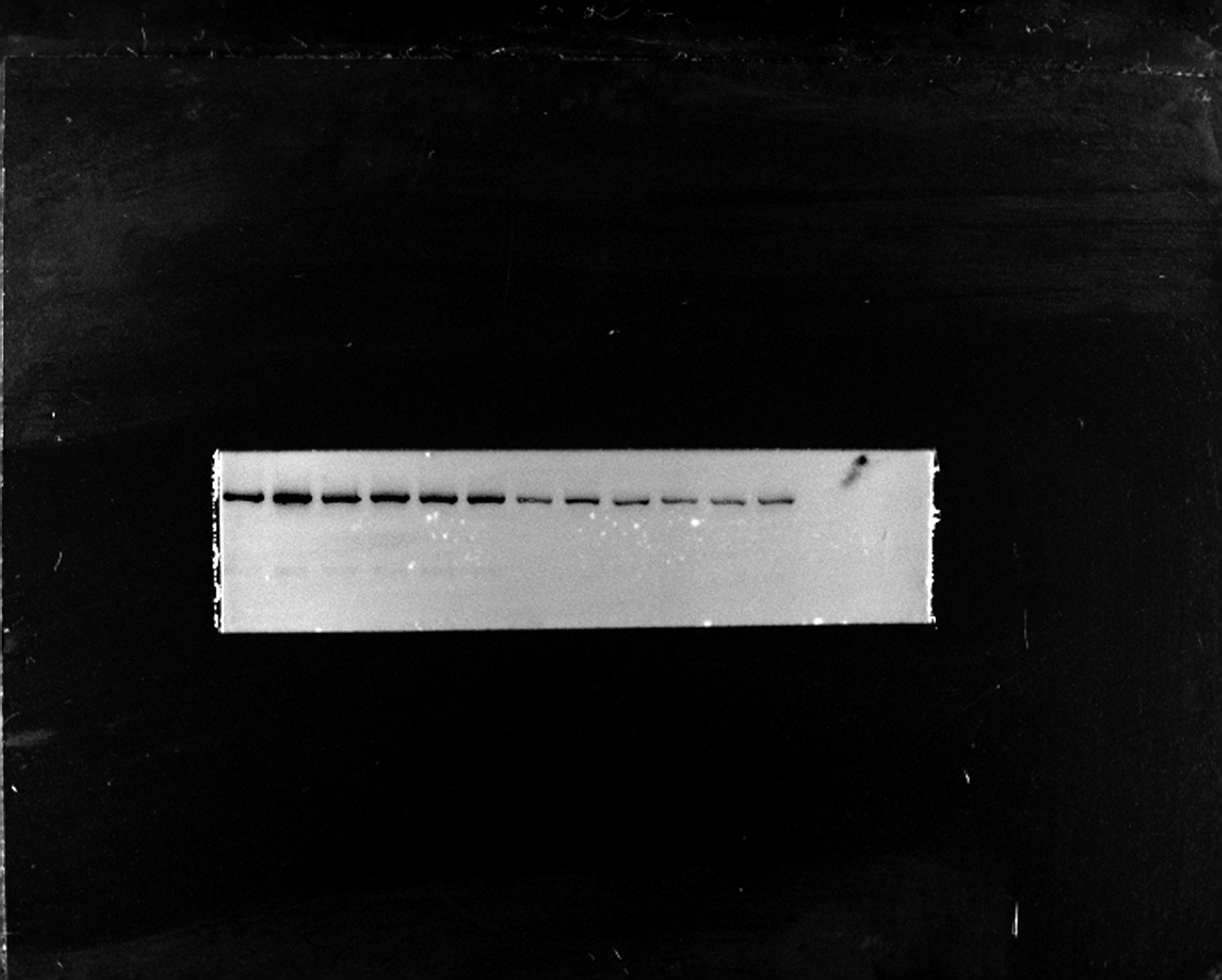

Supplement: Supplementary file 1 — Additional file 1. Raw data. [file 12935_2023_3076_MOESM1_ESM.zip › raw_data/figure8-h1/P-SMAD2/p-SMAD2 1τÖ╜σàë∩╝ê1-6∩╝ÜSaoS-2+si-NC∩╝¢7-12∩╝ÜSaoS-2+si-DIO3OS∩╝ë.tif]

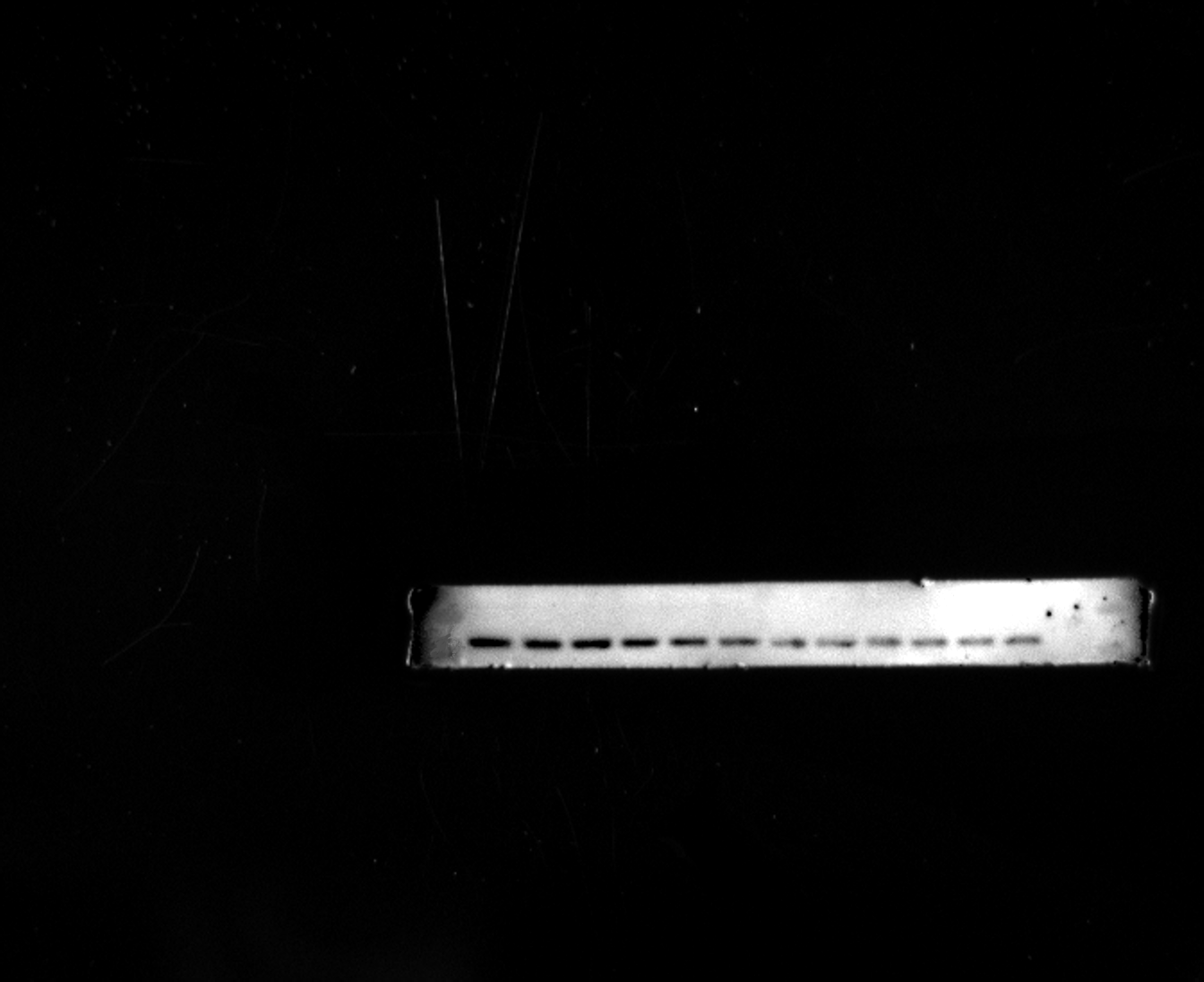

Supplement: Supplementary file 1 — Additional file 1. Raw data. [file 12935_2023_3076_MOESM1_ESM.zip › raw_data/figure8-h1/P-SMAD2/p-SMAD2 2τÖ╜σàë∩╝ê1-6∩╝ÜSaoS-2+si-NC∩╝¢7-12∩╝ÜSaoS-2+si-DIO3OS∩╝ë.Tif]

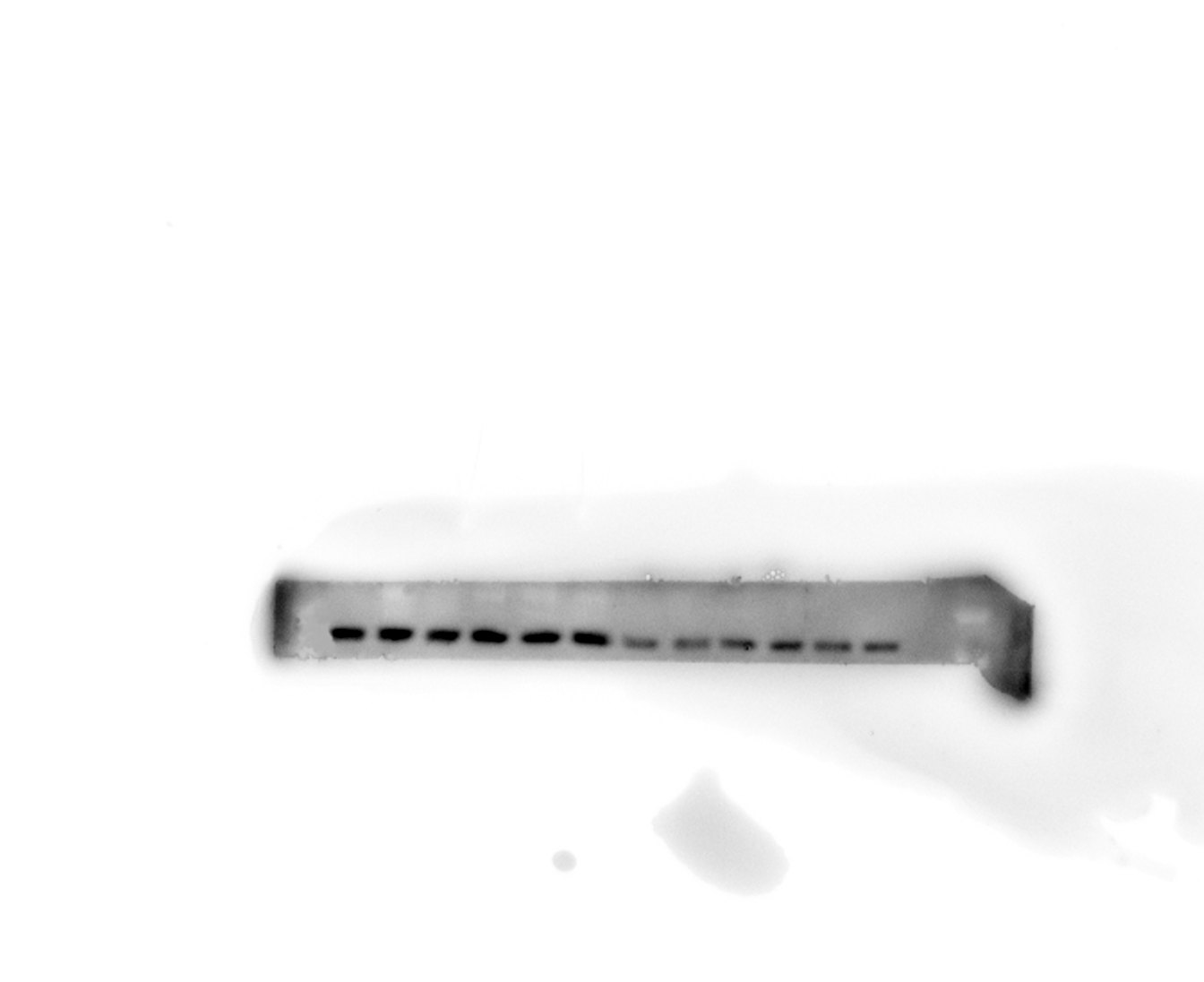

Supplement: Supplementary file 1 — Additional file 1. Raw data. [file 12935_2023_3076_MOESM1_ESM.zip › raw_data/figure8-h1/P-SMAD2/p-SMAD2 3∩╝ê1-6∩╝ÜSaoS-2+si-NC∩╝¢7-12∩╝ÜSaoS-2+si-DIO3OS∩╝ë.Tif]

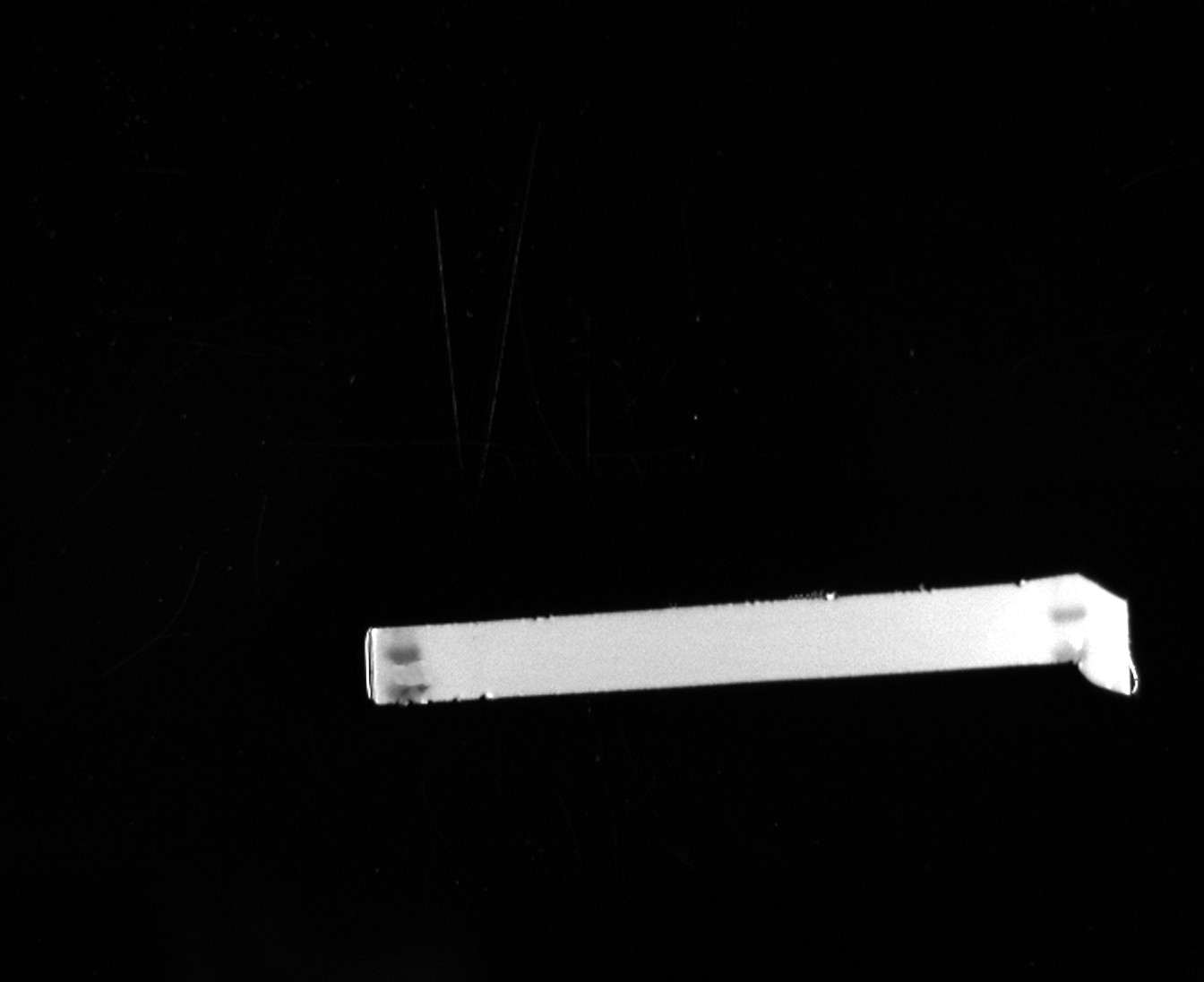

Supplement: Supplementary file 1 — Additional file 1. Raw data. [file 12935_2023_3076_MOESM1_ESM.zip › raw_data/figure8-h1/P-SMAD2/p-SMAD2 3τÖ╜σàë∩╝ê1-6∩╝ÜSaoS-2+si-NC∩╝¢7-12∩╝ÜSaoS-2+si-DIO3OS∩╝ë.Tif]

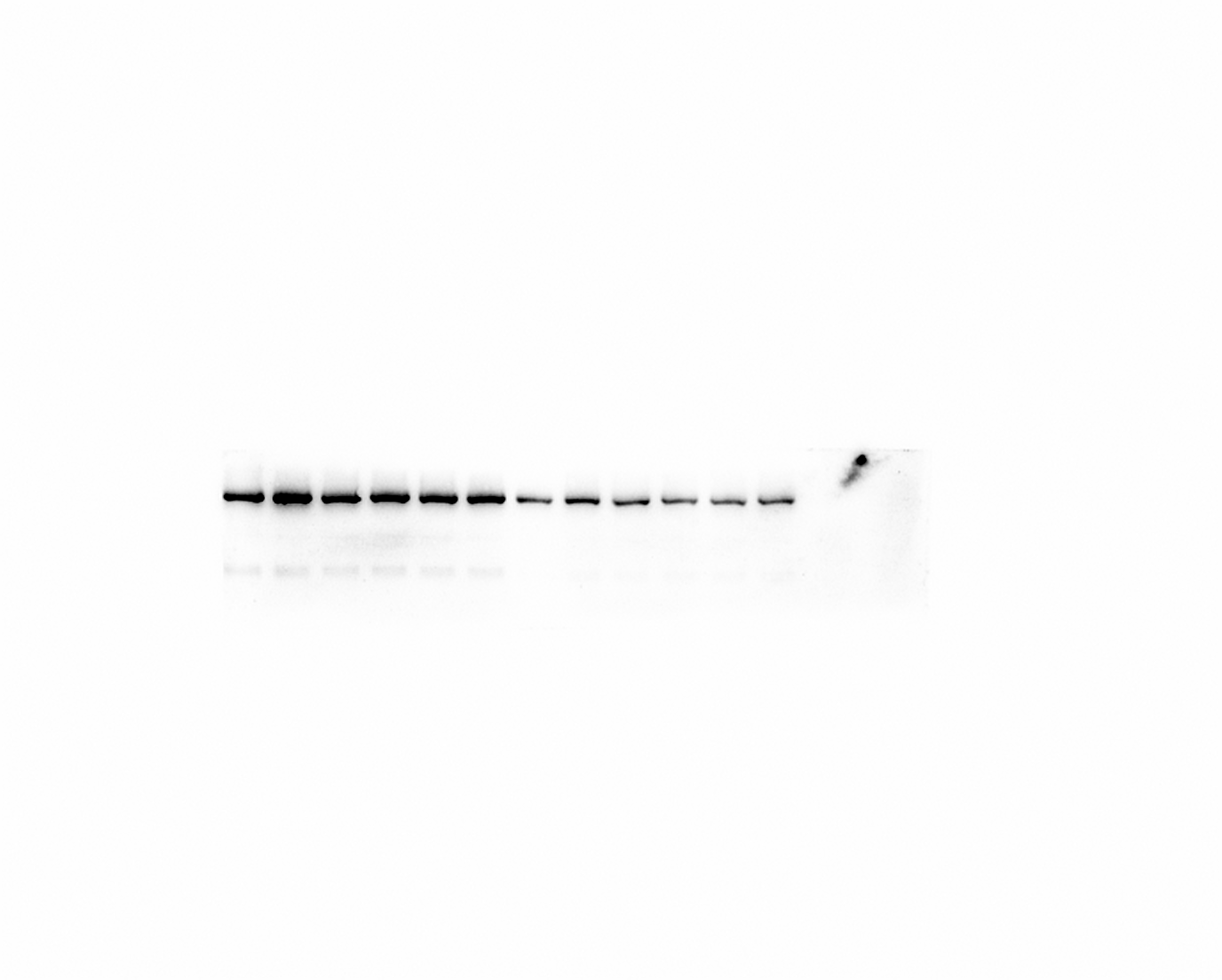

Supplement: Supplementary file 1 — Additional file 1. Raw data. [file 12935_2023_3076_MOESM1_ESM.zip › raw_data/figure8-h1/P-SMAD2/p-SMAD2 1∩╝ê1-6∩╝ÜSaoS-2+si-NC∩╝¢7-12∩╝ÜSaoS-2+si-DIO3OS∩╝ë.tif]

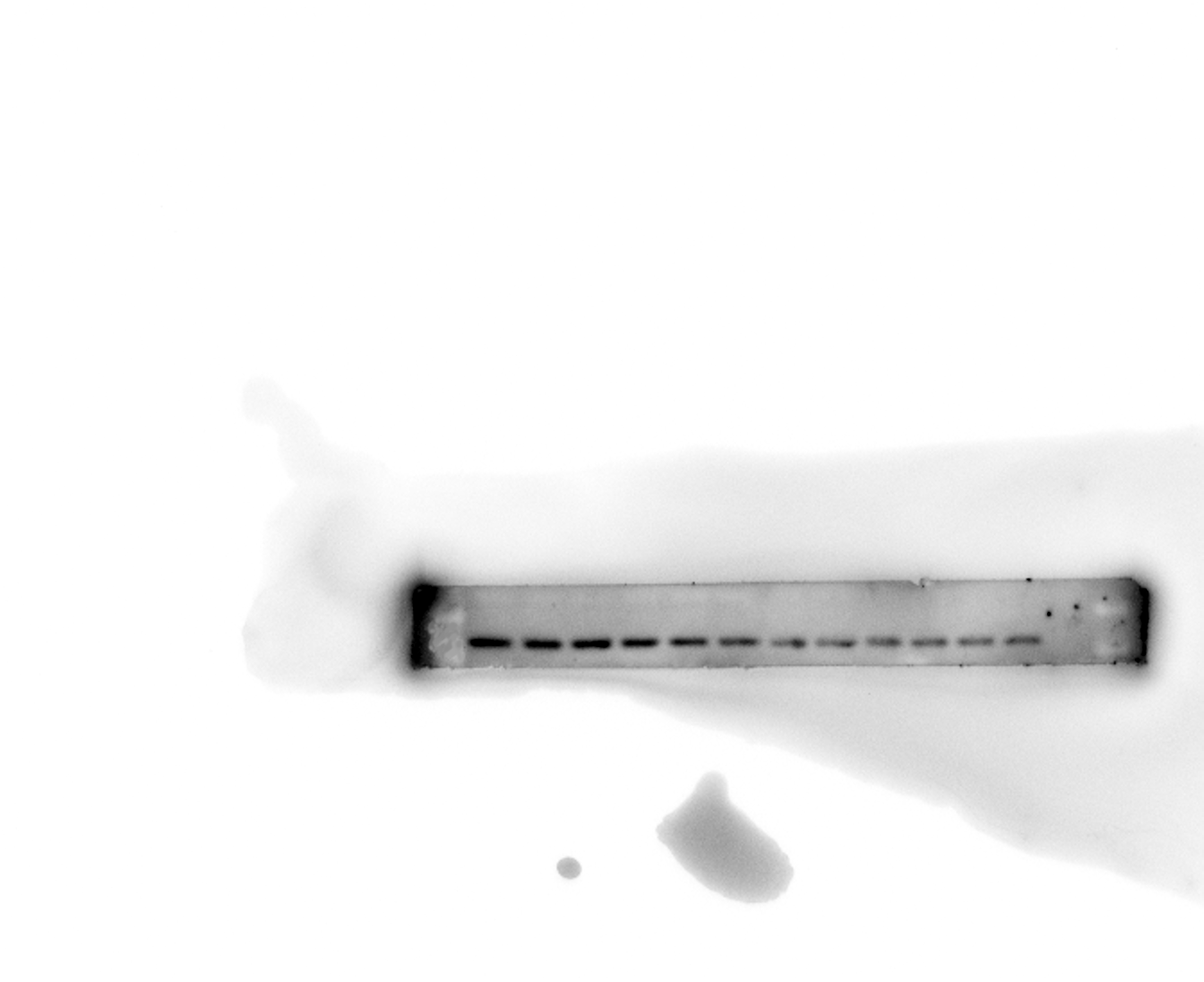

Supplement: Supplementary file 1 — Additional file 1. Raw data. [file 12935_2023_3076_MOESM1_ESM.zip › raw_data/figure8-h1/P-SMAD2/p-SMAD2 2∩╝ê1-6∩╝ÜSaoS-2+si-NC∩╝¢7-12∩╝ÜSaoS-2+si-DIO3OS∩╝ë.Tif]

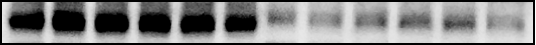

Supplement: Supplementary file 1 — Additional file 1. Raw data. [file 12935_2023_3076_MOESM1_ESM.zip › raw_data/figure8-h1/Figure.8 Vimentin/Vimentin.tif]

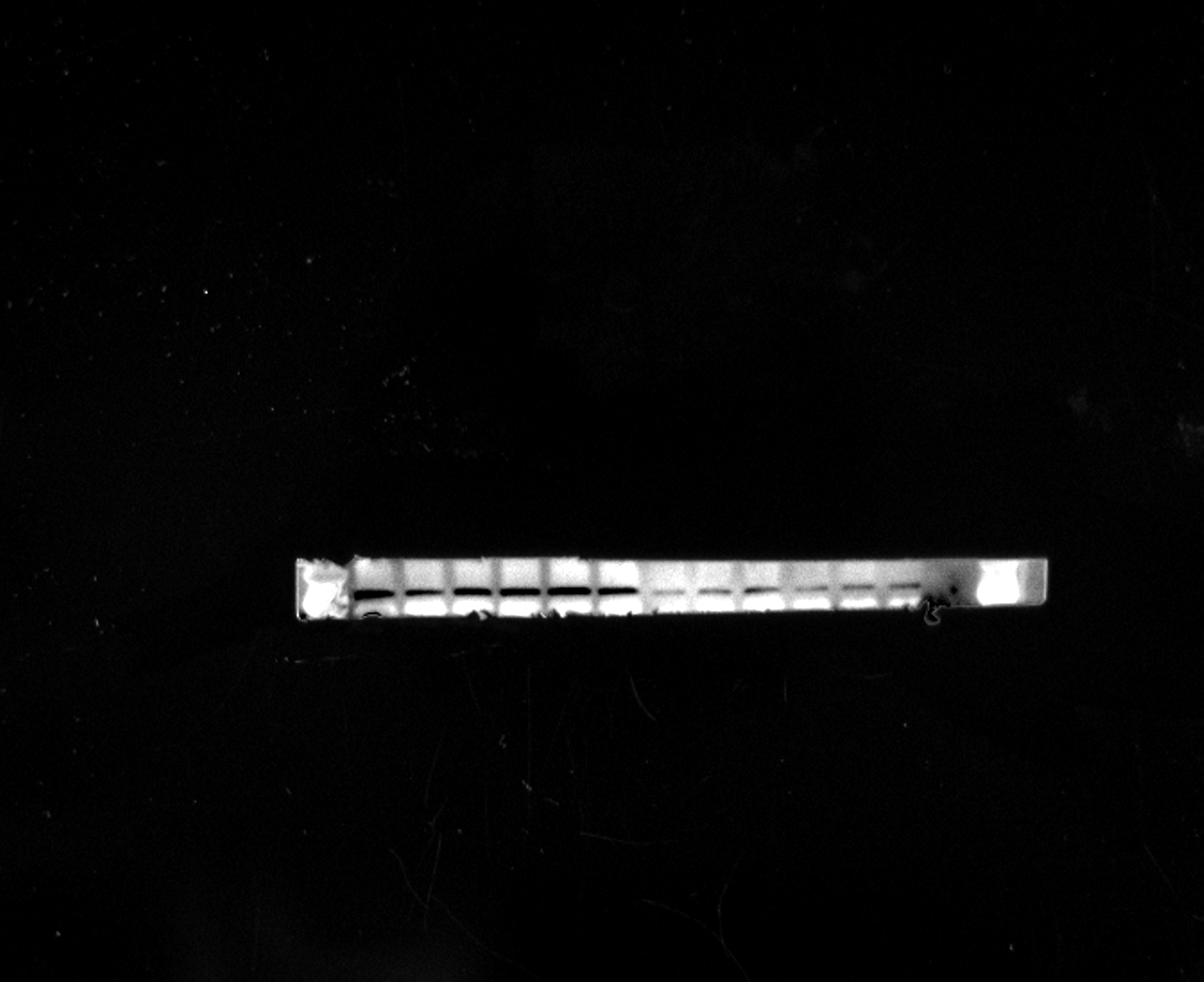

Supplement: Supplementary file 1 — Additional file 1. Raw data. [file 12935_2023_3076_MOESM1_ESM.zip › raw_data/figure8-h1/Figure.8 Vimentin/Vimentin3τÖ╜σàë∩╝êgapdh(1-6∩╝ÜSaoS-2+si-NC∩╝¢7-12∩╝ÜSaoS-2+si-DIO3OS∩╝ë.Tif.Tif]

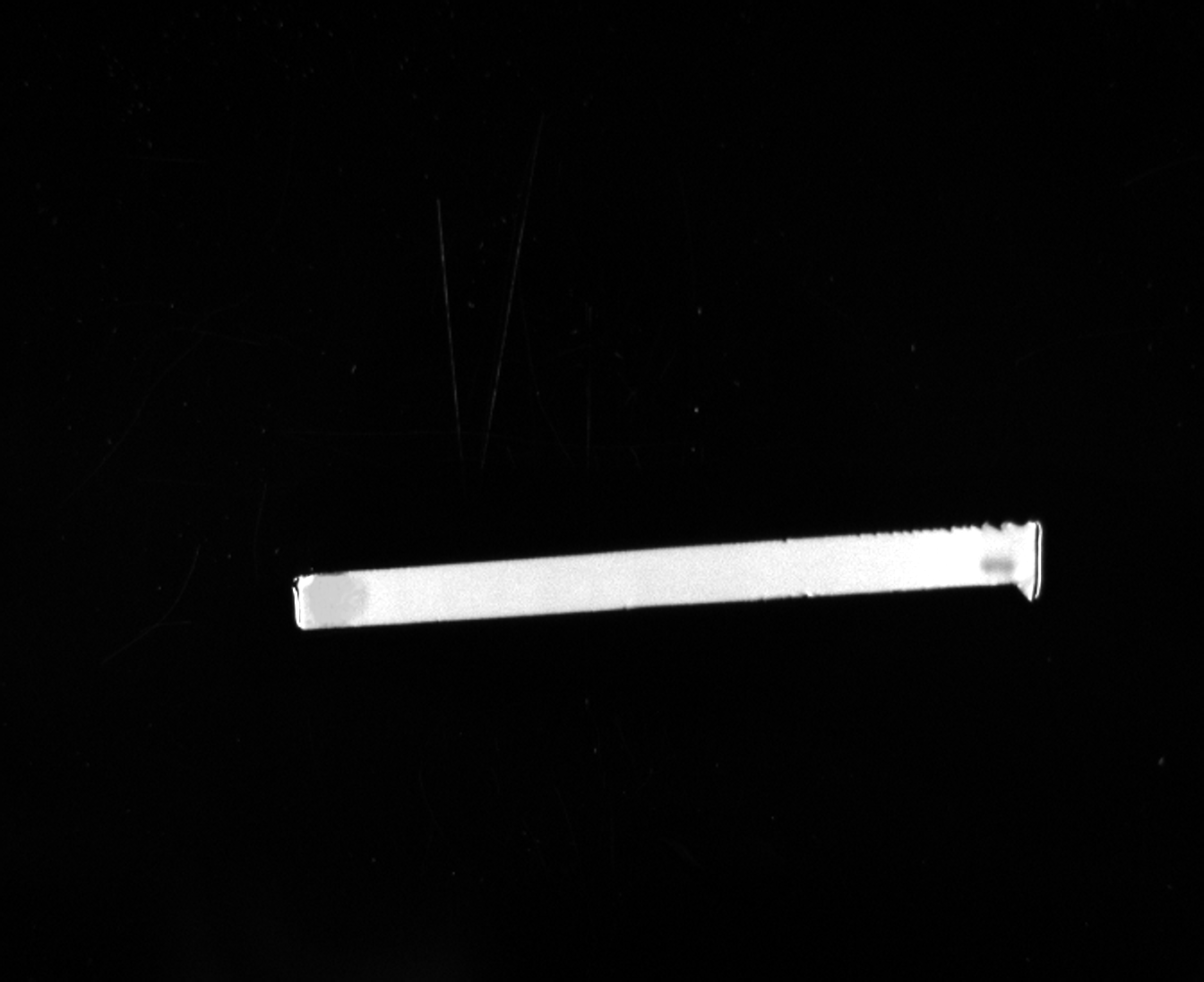

Supplement: Supplementary file 1 — Additional file 1. Raw data. [file 12935_2023_3076_MOESM1_ESM.zip › raw_data/figure8-h1/Figure.8 Vimentin/Vimentin2∩╝êτÖ╜σàëgapdh(1-6∩╝ÜSaoS-2+si-NC∩╝¢7-12∩╝ÜSaoS-2+si-DIO3OS∩╝ë.Tif]

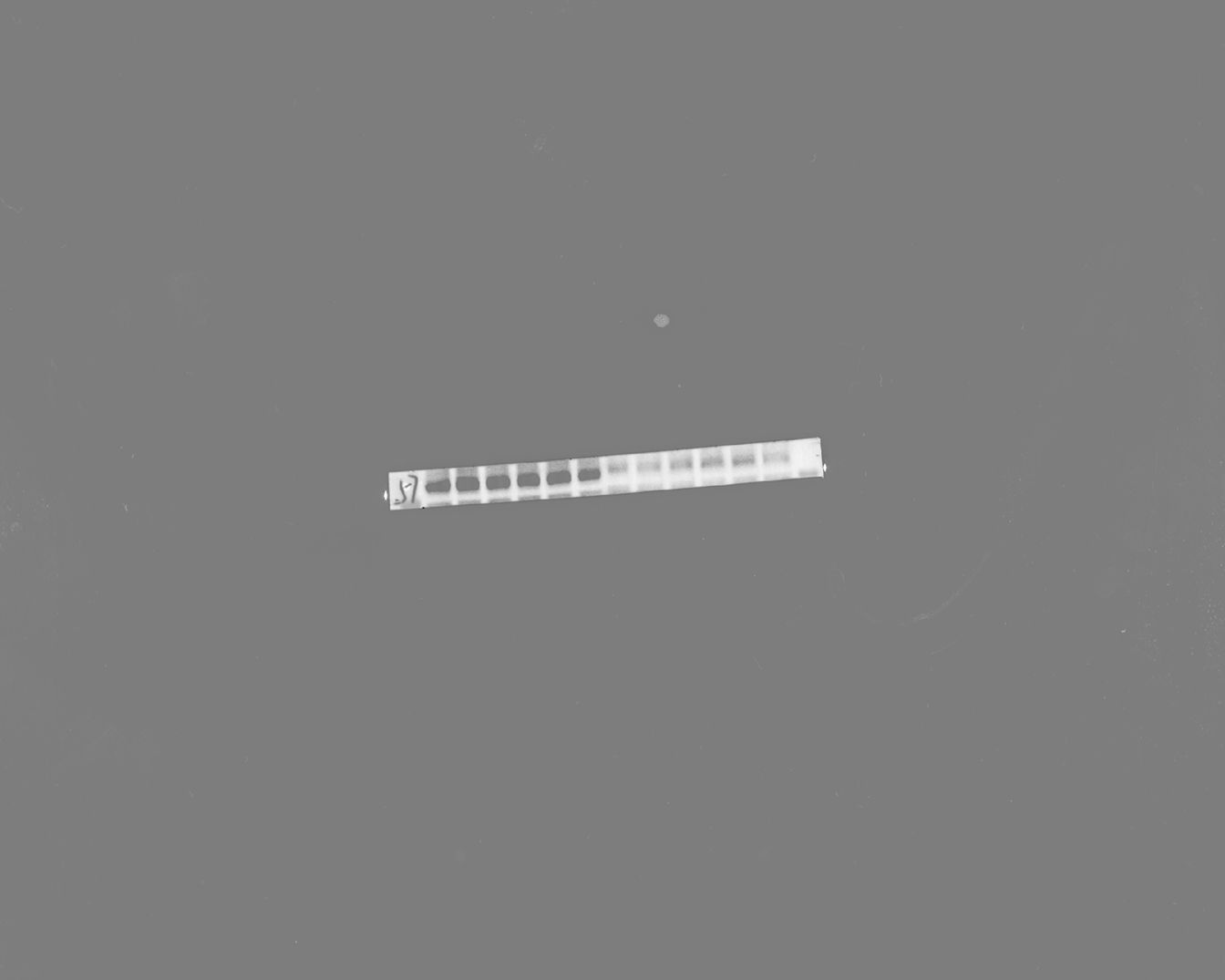

Supplement: Supplementary file 1 — Additional file 1. Raw data. [file 12935_2023_3076_MOESM1_ESM.zip › raw_data/figure8-h1/Figure.8 Vimentin/Vimentin1τÖ╜σàë∩╝êgapdh(1-6∩╝ÜSaoS-2+si-NC∩╝¢7-12∩╝ÜSaoS-2+si-DIO3OS∩╝ë.tif]

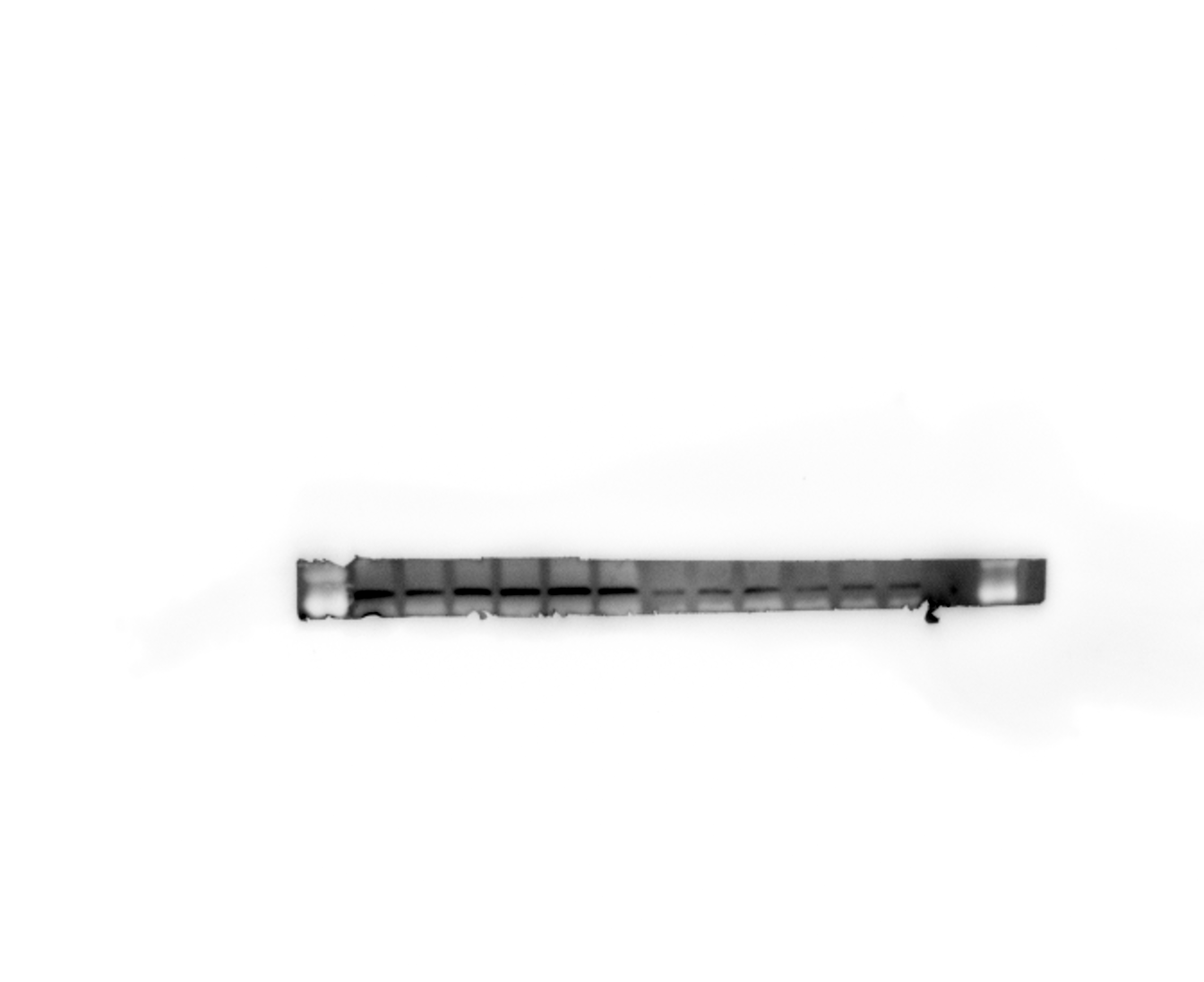

Supplement: Supplementary file 1 — Additional file 1. Raw data. [file 12935_2023_3076_MOESM1_ESM.zip › raw_data/figure8-h1/Figure.8 Vimentin/Vimentin3∩╝êgapdh(1-6∩╝ÜSaoS-2+si-NC∩╝¢7-12∩╝ÜSaoS-2+si-DIO3OS∩╝ë.Tif]

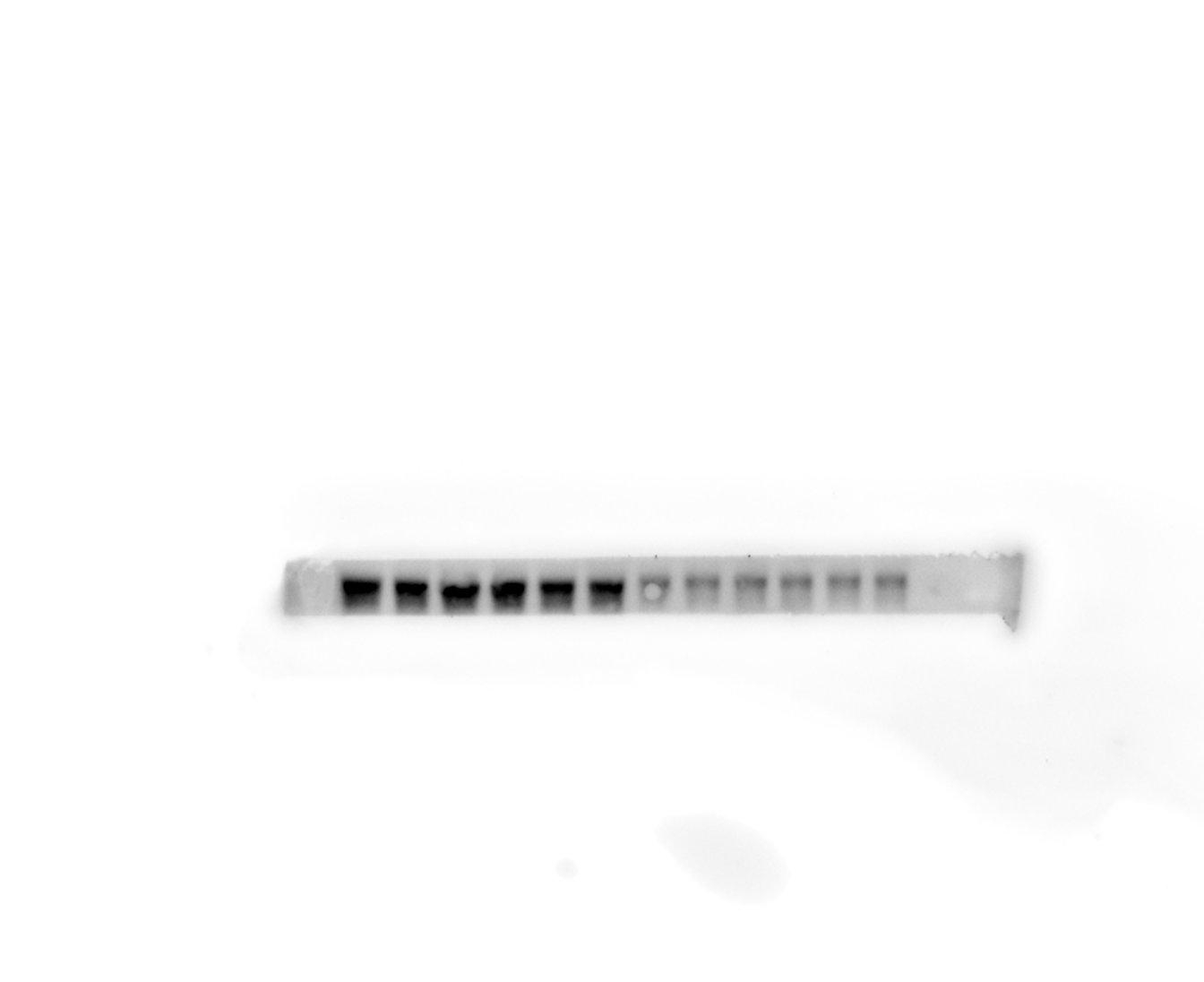

Supplement: Supplementary file 1 — Additional file 1. Raw data. [file 12935_2023_3076_MOESM1_ESM.zip › raw_data/figure8-h1/Figure.8 Vimentin/Vimentin2∩╝êgapdh(1-6∩╝ÜSaoS-2+si-NC∩╝¢7-12∩╝ÜSaoS-2+si-DIO3OS∩╝ë.Tif]

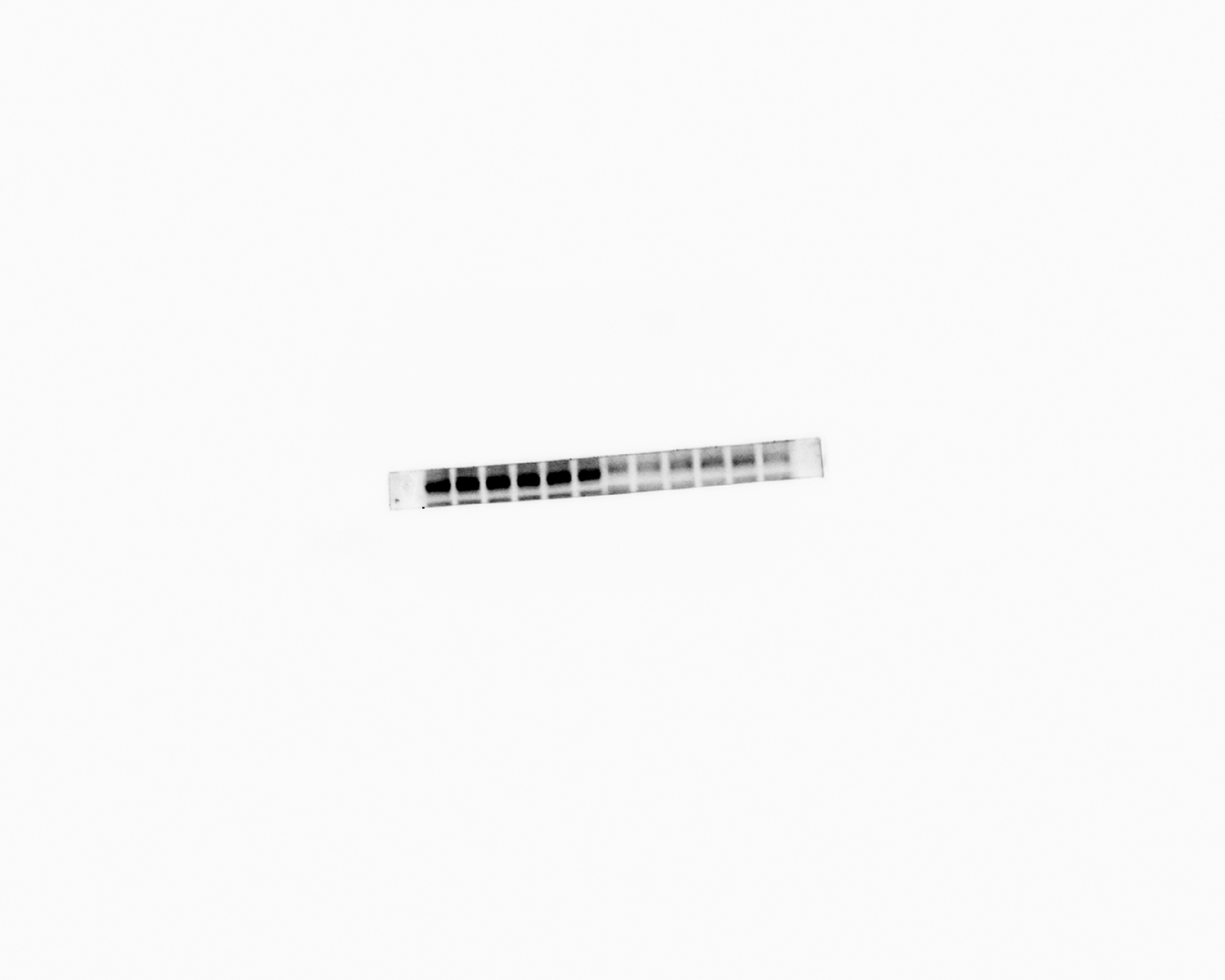

Supplement: Supplementary file 1 — Additional file 1. Raw data. [file 12935_2023_3076_MOESM1_ESM.zip › raw_data/figure8-h1/Figure.8 Vimentin/Vimentin1∩╝êgapdh(1-6∩╝ÜSaoS-2+si-NC∩╝¢7-12∩╝ÜSaoS-2+si-DIO3OS∩╝ë.tif]

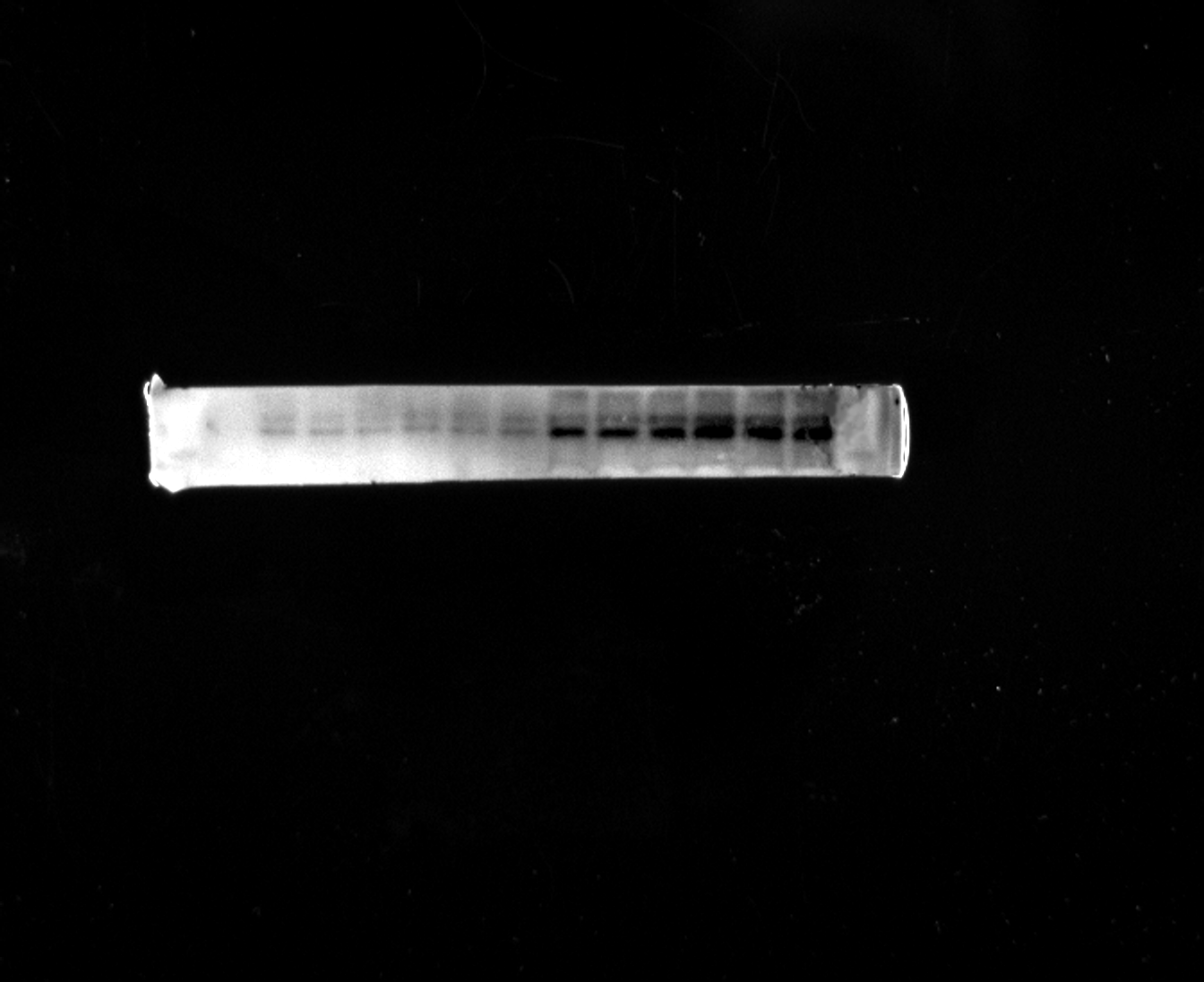

Supplement: Supplementary file 1 — Additional file 1. Raw data. [file 12935_2023_3076_MOESM1_ESM.zip › raw_data/figure8-h1/E-cad/E-cad1τÖ╜σàë∩╝êgapdh(1-6∩╝ÜSaoS-2+si-NC∩╝¢7-12∩╝ÜSaoS-2+si-DIO3OS∩╝ë.Tif]

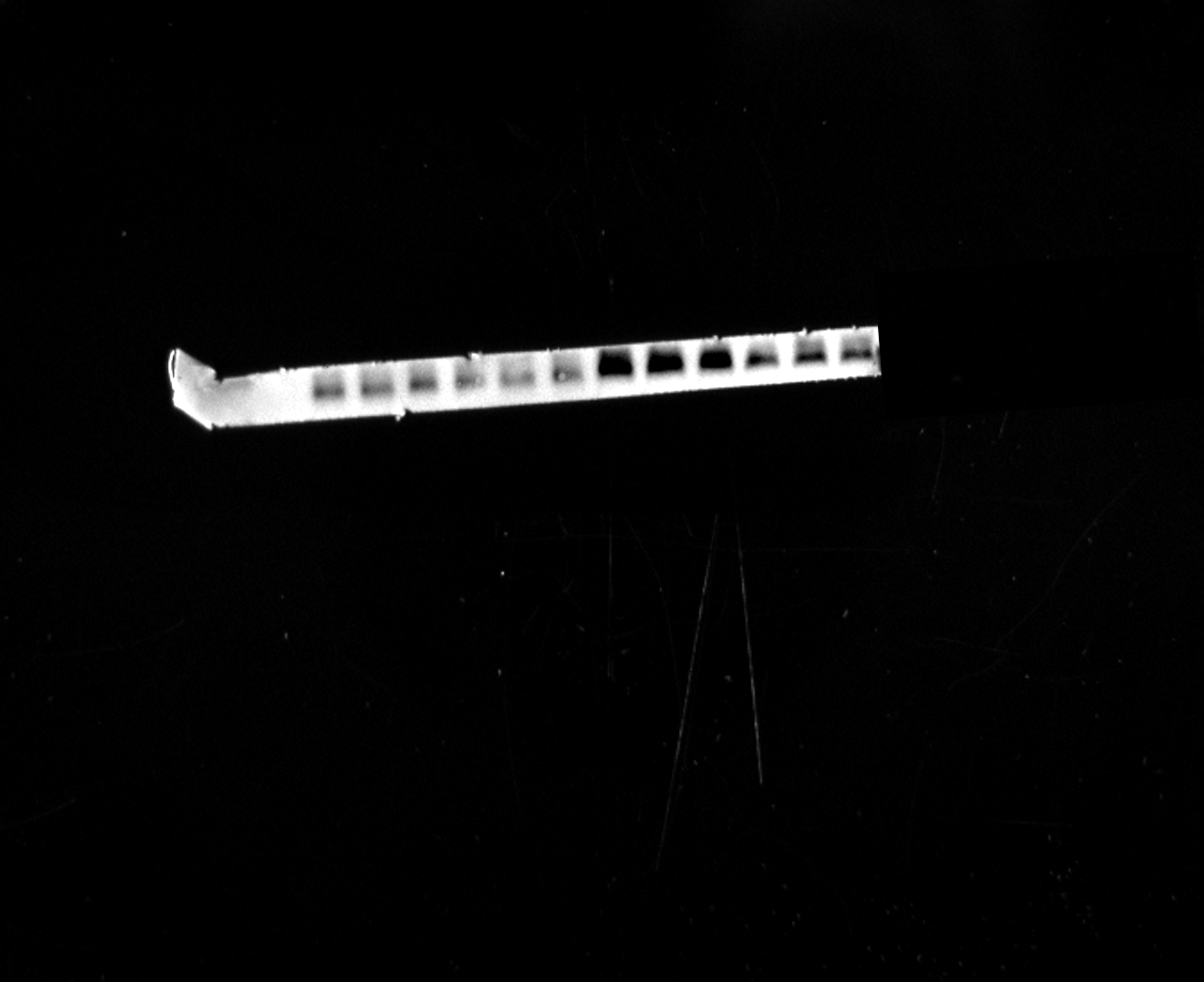

Supplement: Supplementary file 1 — Additional file 1. Raw data. [file 12935_2023_3076_MOESM1_ESM.zip › raw_data/figure8-h1/E-cad/E-cad3τÖ╜σàë∩╝êgapdh(1-6∩╝ÜSaoS-2+si-NC∩╝¢7-12∩╝ÜSaoS-2+si-DIO3OS∩╝ë.Tif.Tif]

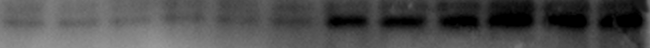

Supplement: Supplementary file 1 — Additional file 1. Raw data. [file 12935_2023_3076_MOESM1_ESM.zip › raw_data/figure8-h1/E-cad/E-cad.tif]

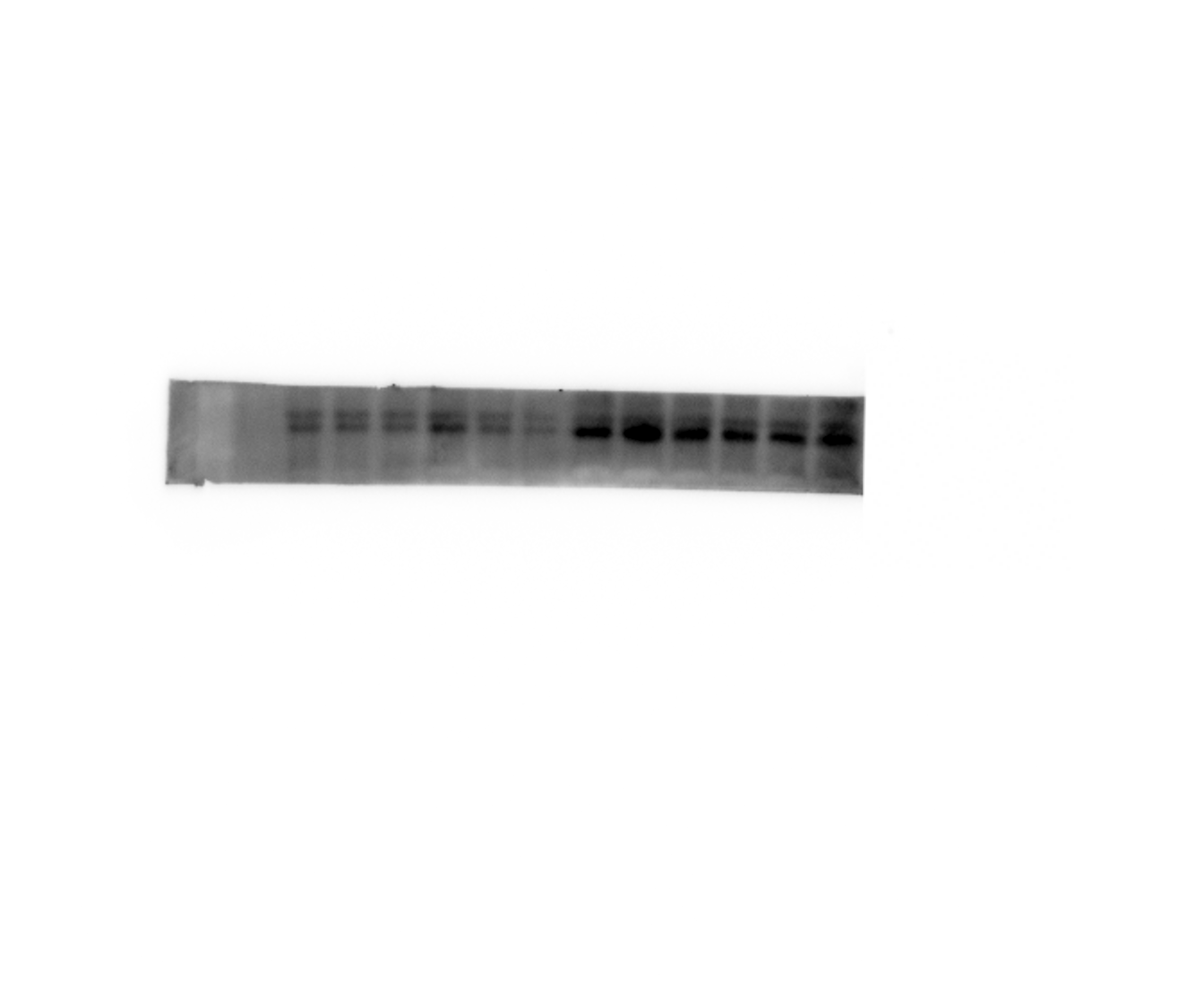

Supplement: Supplementary file 1 — Additional file 1. Raw data. [file 12935_2023_3076_MOESM1_ESM.zip › raw_data/figure8-h1/E-cad/E-cad2∩╝êgapdh(1-6∩╝ÜSaoS-2+si-NC∩╝¢7-12∩╝ÜSaoS-2+si-DIO3OS∩╝ë.Tif.Tif]

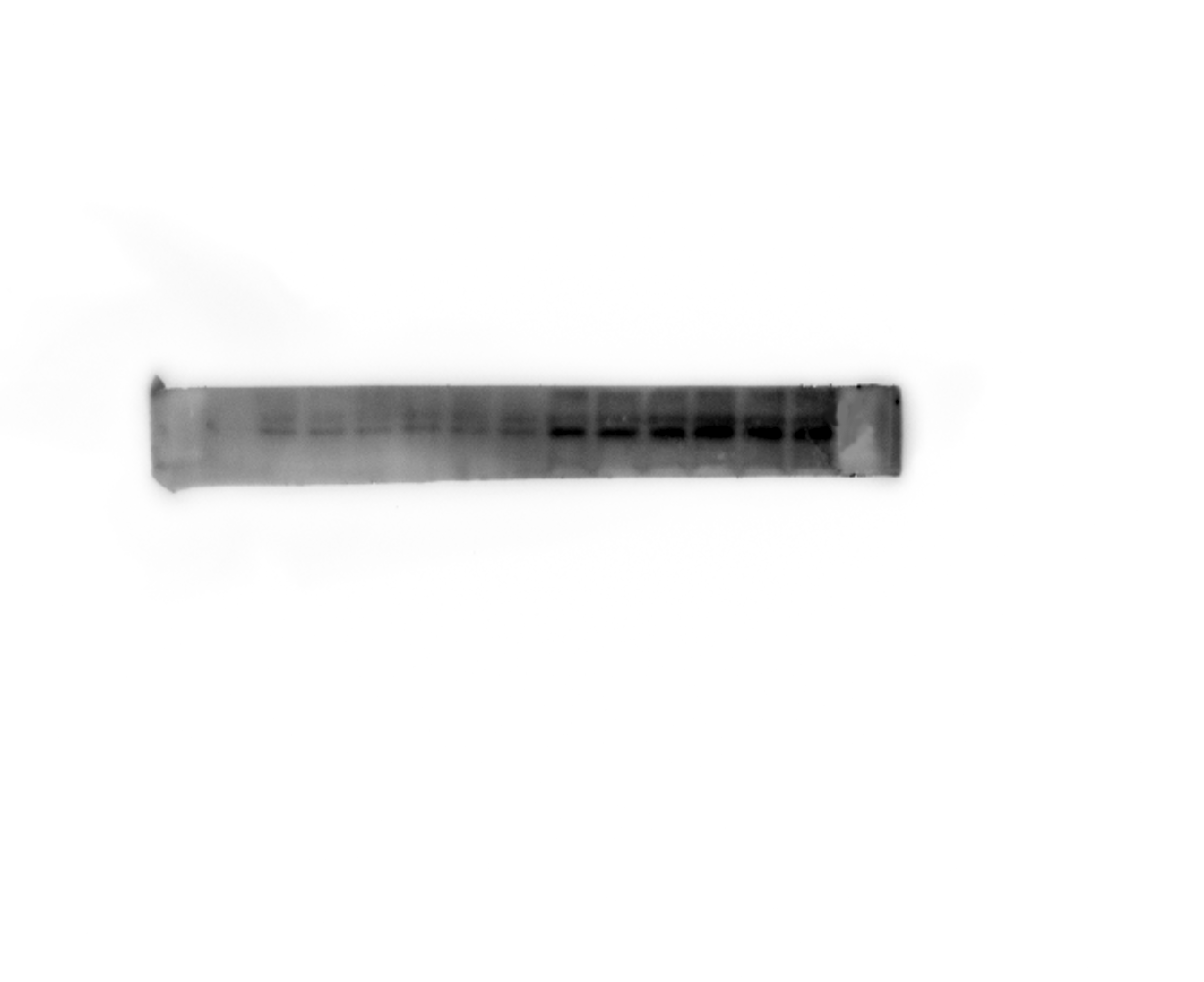

Supplement: Supplementary file 1 — Additional file 1. Raw data. [file 12935_2023_3076_MOESM1_ESM.zip › raw_data/figure8-h1/E-cad/E-cad1∩╝êgapdh(1-6∩╝ÜSaoS-2+si-NC∩╝¢7-12∩╝ÜSaoS-2+si-DIO3OS∩╝ë.Tif.Tif]

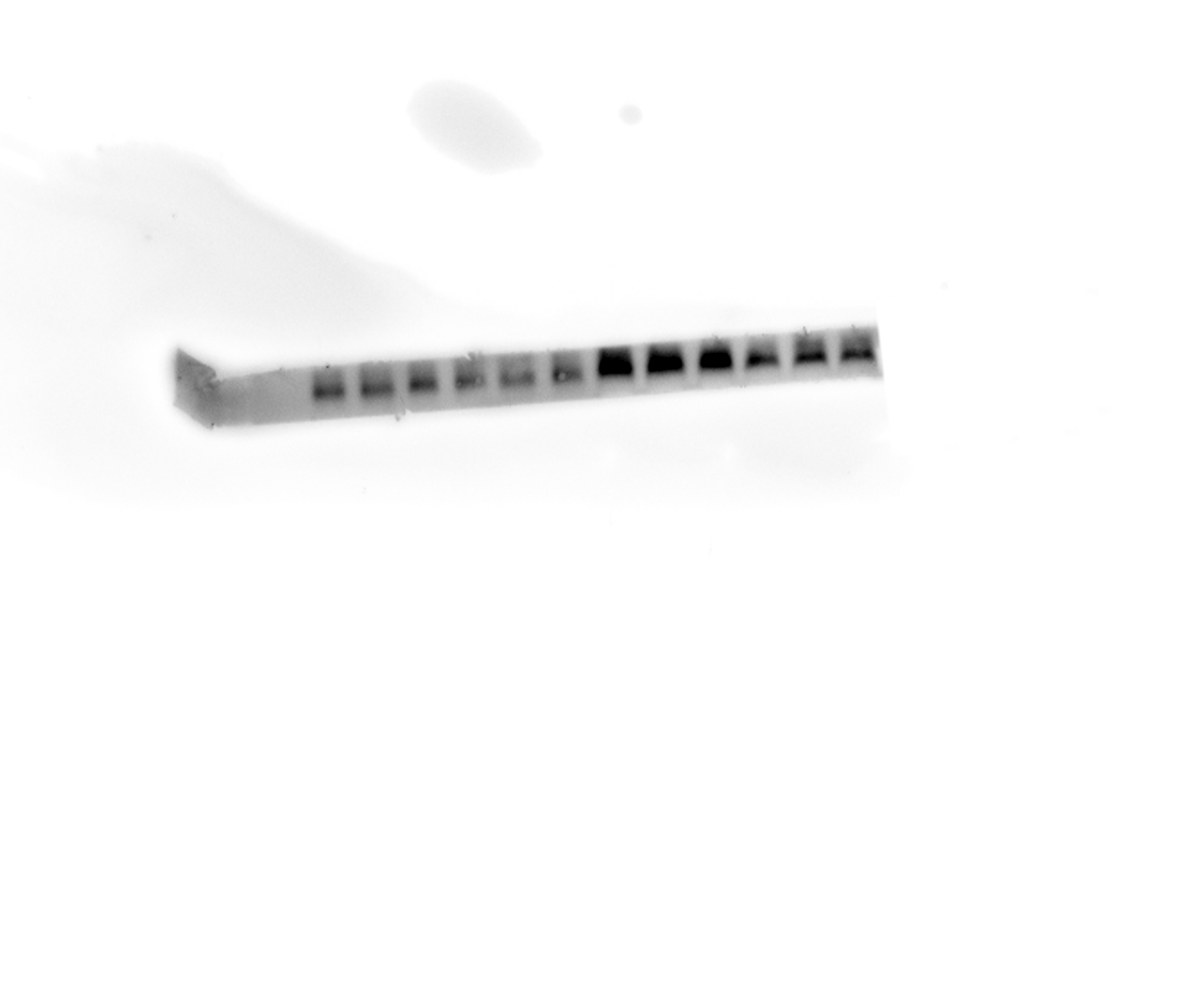

Supplement: Supplementary file 1 — Additional file 1. Raw data. [file 12935_2023_3076_MOESM1_ESM.zip › raw_data/figure8-h1/E-cad/E-cad3∩╝êgapdh(1-6∩╝ÜSaoS-2+si-NC∩╝¢7-12∩╝ÜSaoS-2+si-DIO3OS∩╝ë.Tif.Tif]

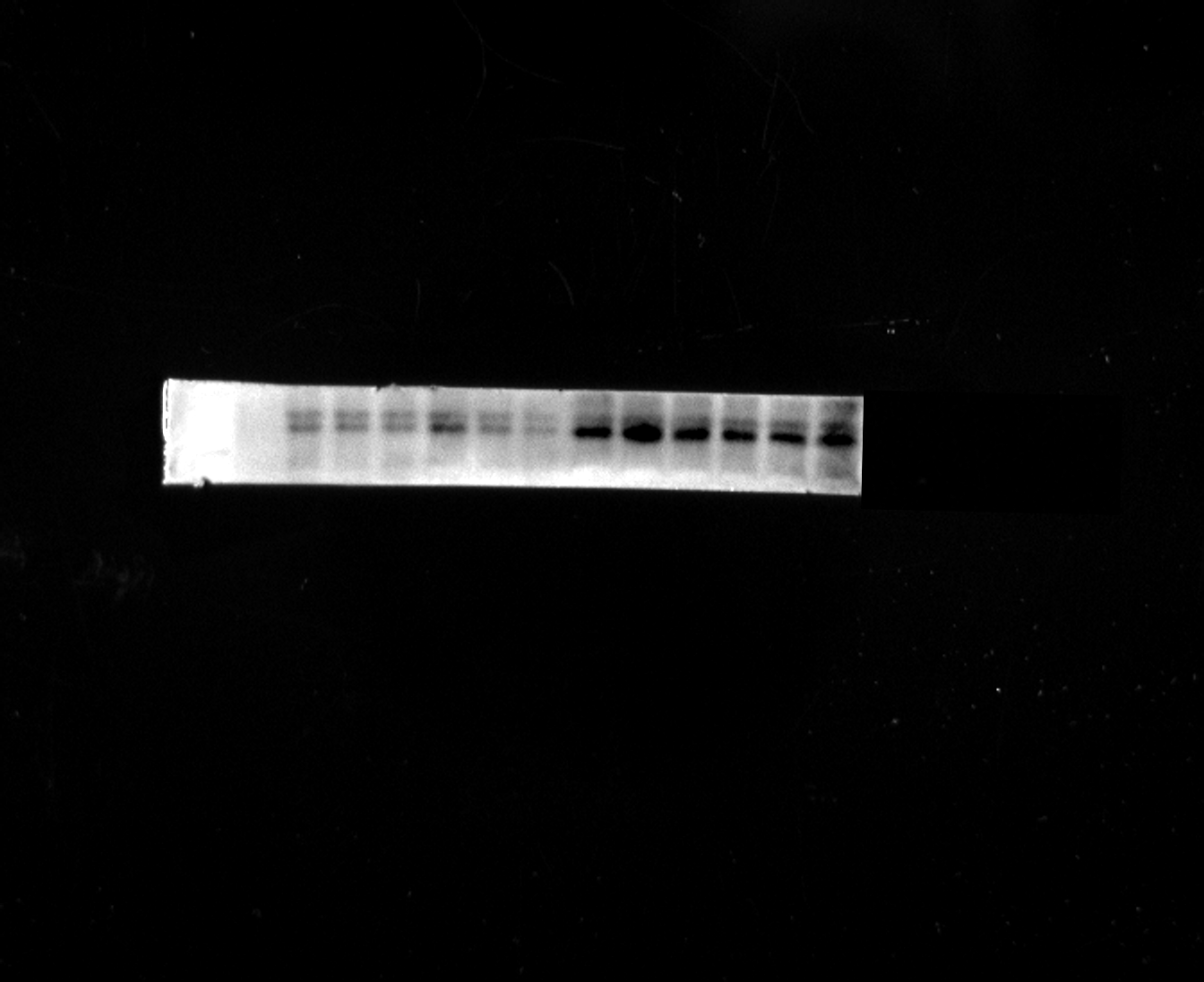

Supplement: Supplementary file 1 — Additional file 1. Raw data. [file 12935_2023_3076_MOESM1_ESM.zip › raw_data/figure8-h1/E-cad/E-cad2τÖ╜σàë∩╝êgapdh(1-6∩╝ÜSaoS-2+si-NC∩╝¢7-12∩╝ÜSaoS-2+si-DIO3OS∩╝ë.Tif.Tif]

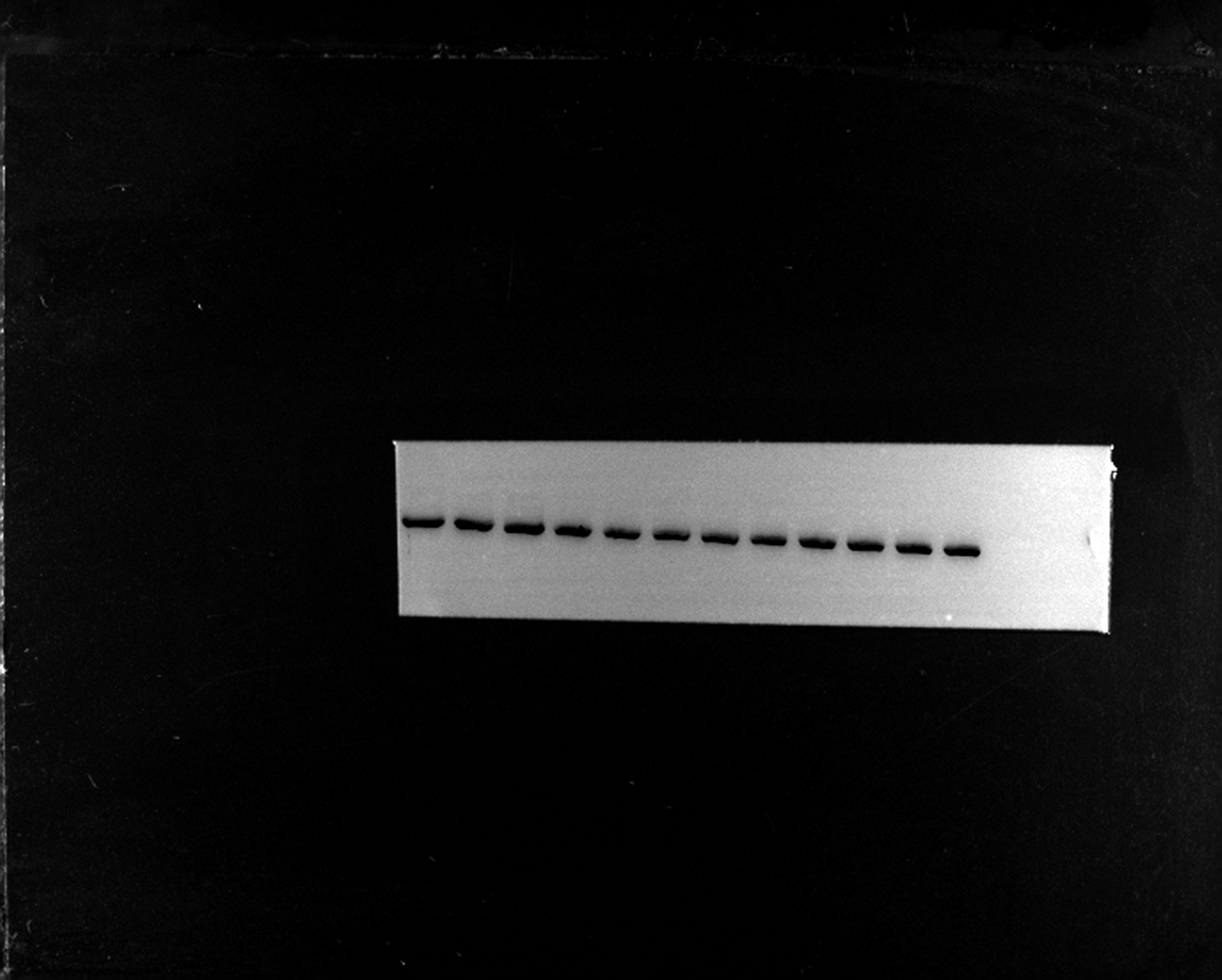

Supplement: Supplementary file 1 — Additional file 1. Raw data. [file 12935_2023_3076_MOESM1_ESM.zip › raw_data/figure8-h1/GAPDH/GAPDHτÖ╜σàë1∩╝ê1-6∩╝ÜSaoS-2+si-NC∩╝¢7-12∩╝ÜSaoS-2+si-DIO3OS∩╝ë.tif]

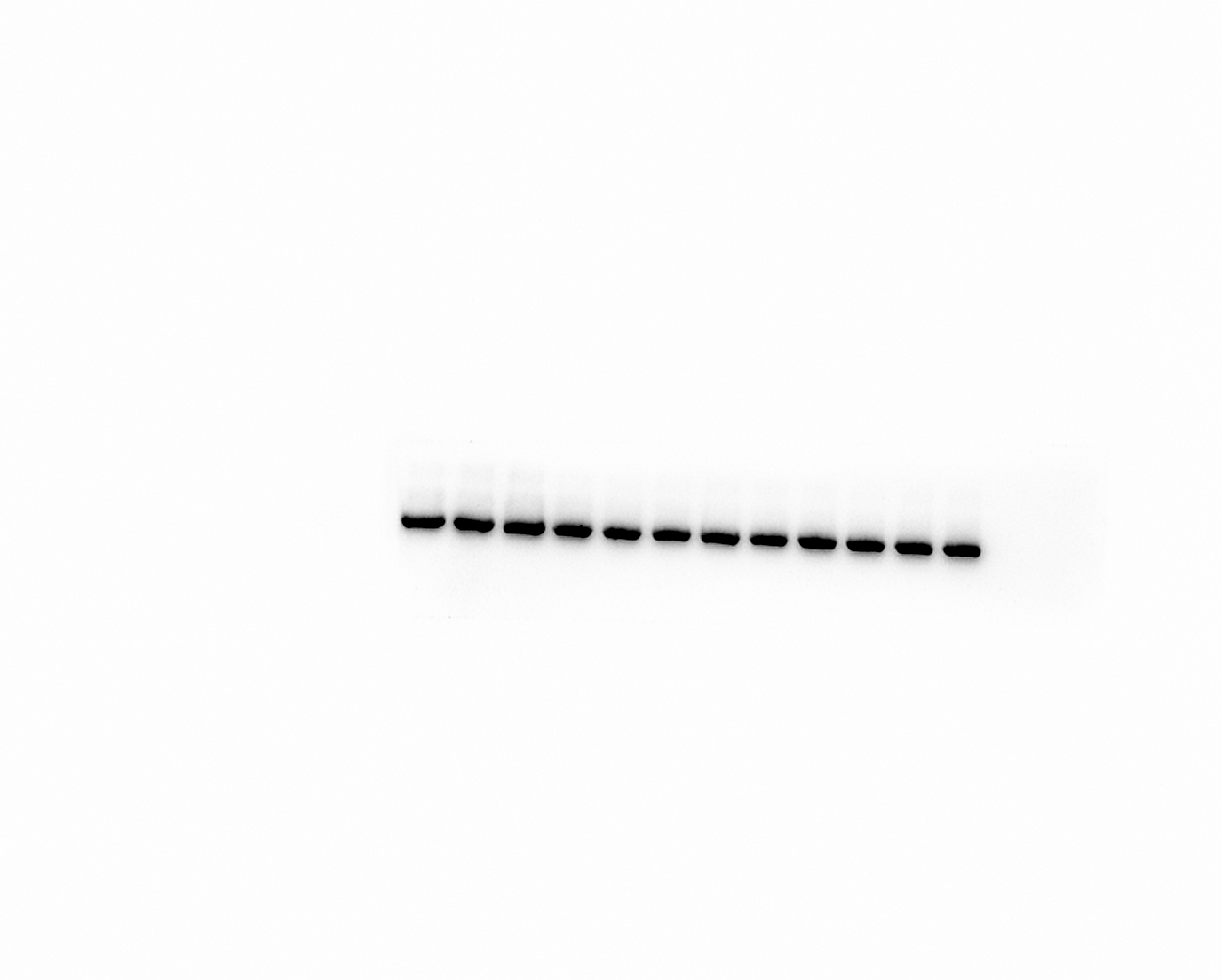

Supplement: Supplementary file 1 — Additional file 1. Raw data. [file 12935_2023_3076_MOESM1_ESM.zip › raw_data/figure8-h1/GAPDH/GAPDH1∩╝ê1-6∩╝ÜSaoS-2+si-NC∩╝¢7-12∩╝ÜSaoS-2+si-DIO3OS∩╝ë.tif]

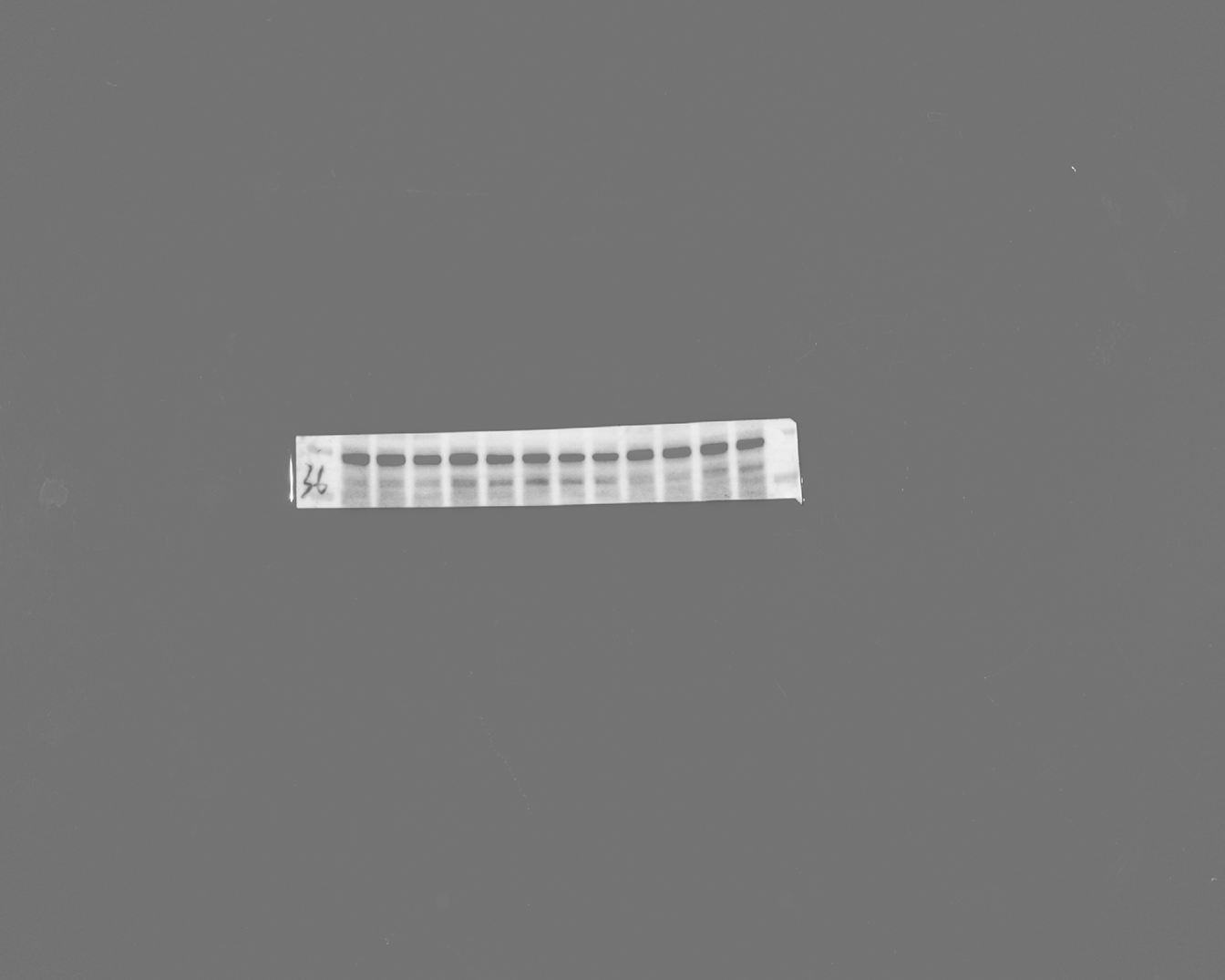

Supplement: Supplementary file 1 — Additional file 1. Raw data. [file 12935_2023_3076_MOESM1_ESM.zip › raw_data/figure8-h1/GAPDH/GAPDHτÖ╜σàë2(1-6∩╝ÜSaoS-2+si-NC∩╝¢7-12∩╝ÜSaoS-2+si-DIO3OS∩╝ë.tif]

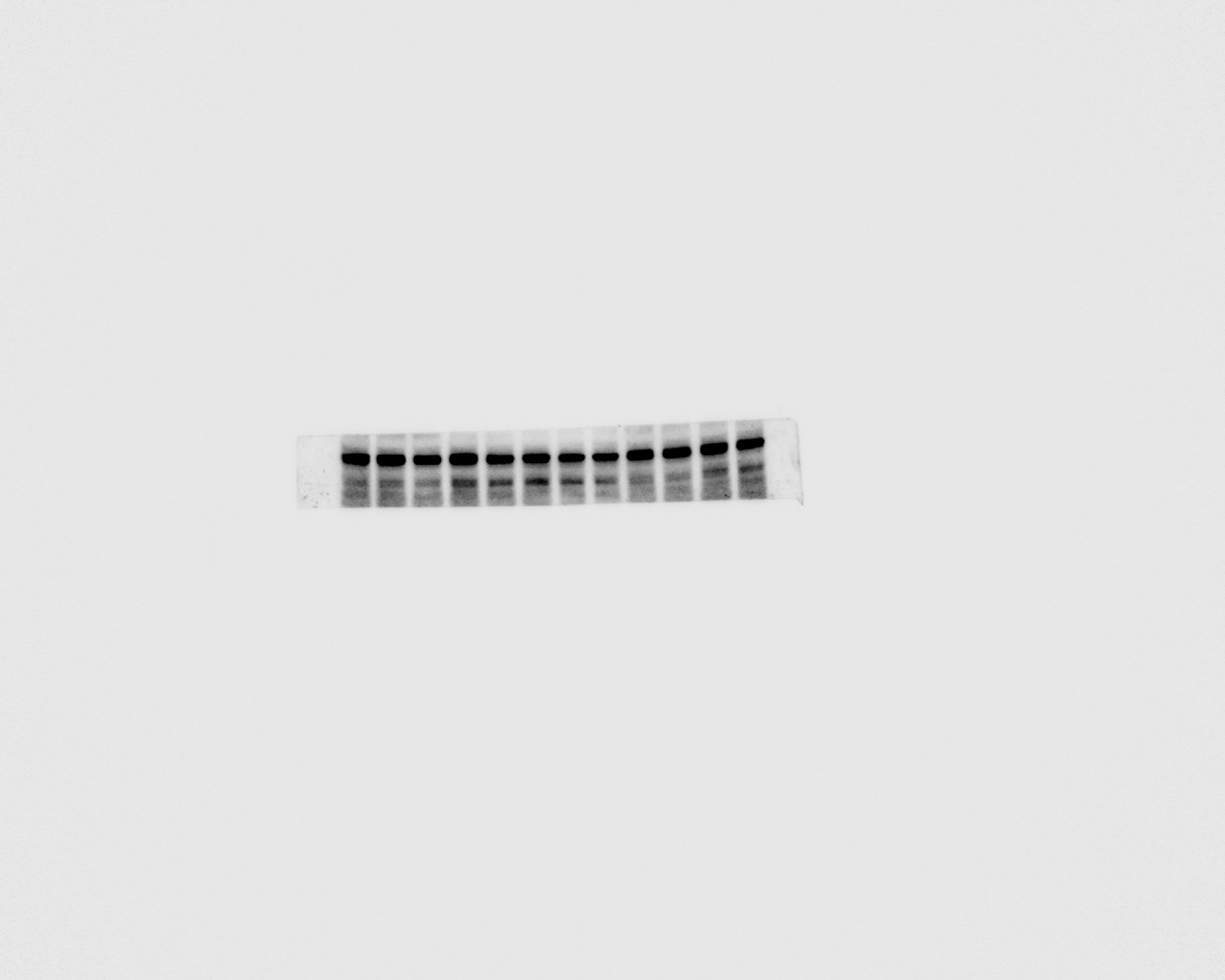

Supplement: Supplementary file 1 — Additional file 1. Raw data. [file 12935_2023_3076_MOESM1_ESM.zip › raw_data/figure8-h1/GAPDH/GAPDH2(1-6∩╝ÜSaoS-2+si-NC∩╝¢7-12∩╝ÜSaoS-2+si-DIO3OS∩╝ë.tif]

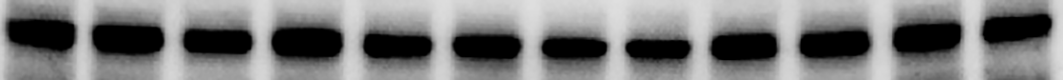

Supplement: Supplementary file 1 — Additional file 1. Raw data. [file 12935_2023_3076_MOESM1_ESM.zip › raw_data/figure8-h1/GAPDH/gapdh.tif]

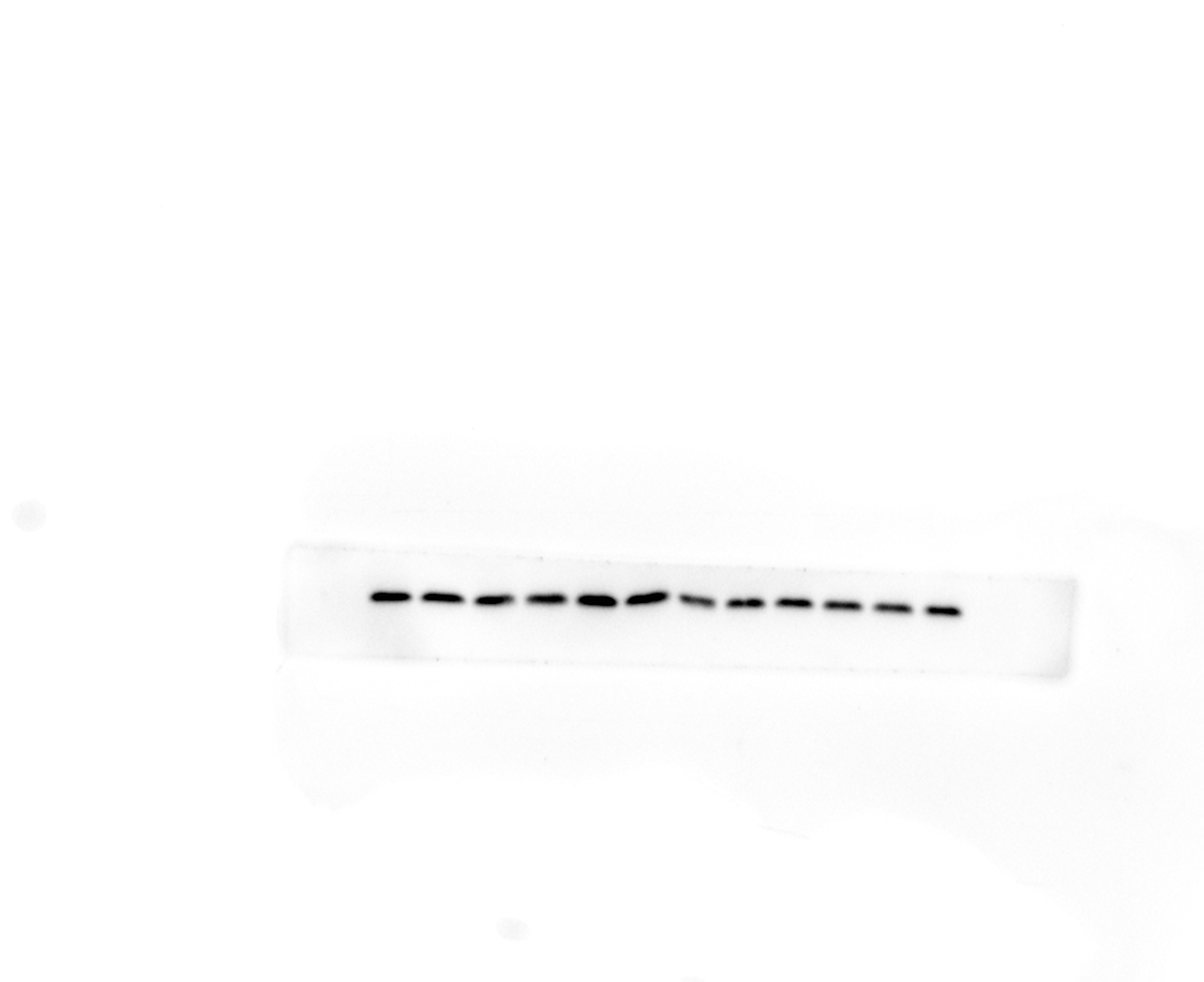

Supplement: Supplementary file 1 — Additional file 1. Raw data. [file 12935_2023_3076_MOESM1_ESM.zip › raw_data/figure8-h1/GAPDH/GAPDH3∩╝ê1-6∩╝ÜSaoS-2+si-NC∩╝¢7-12∩╝ÜSaoS-2+si-DIO3OS∩╝ë.Tif]

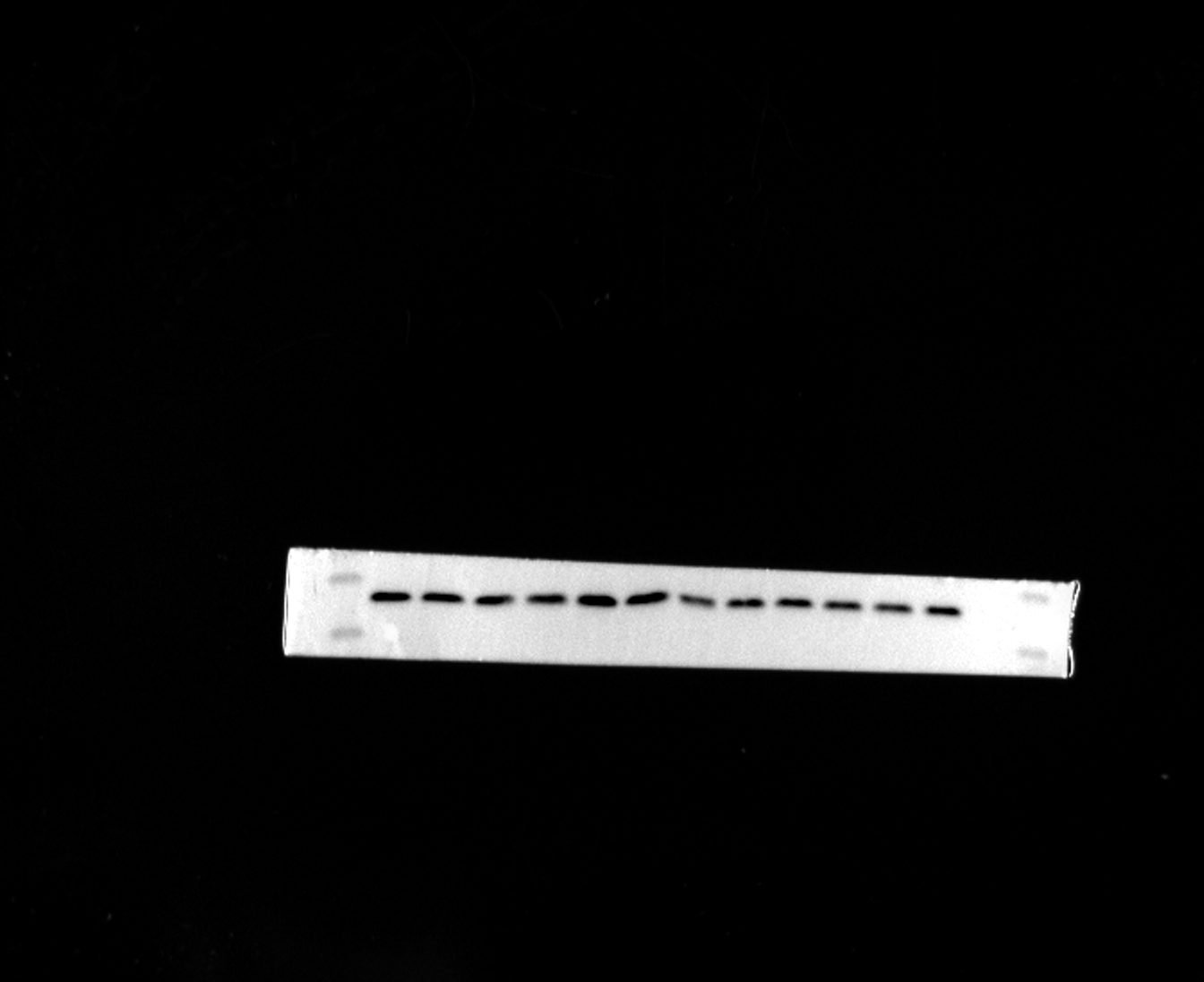

Supplement: Supplementary file 1 — Additional file 1. Raw data. [file 12935_2023_3076_MOESM1_ESM.zip › raw_data/figure8-h1/GAPDH/GAPDHτÖ╜σàë3∩╝ê1-6∩╝ÜSaoS-2+si-NC∩╝¢7-12∩╝ÜSaoS-2+si-DIO3OS∩╝ë.Tif]

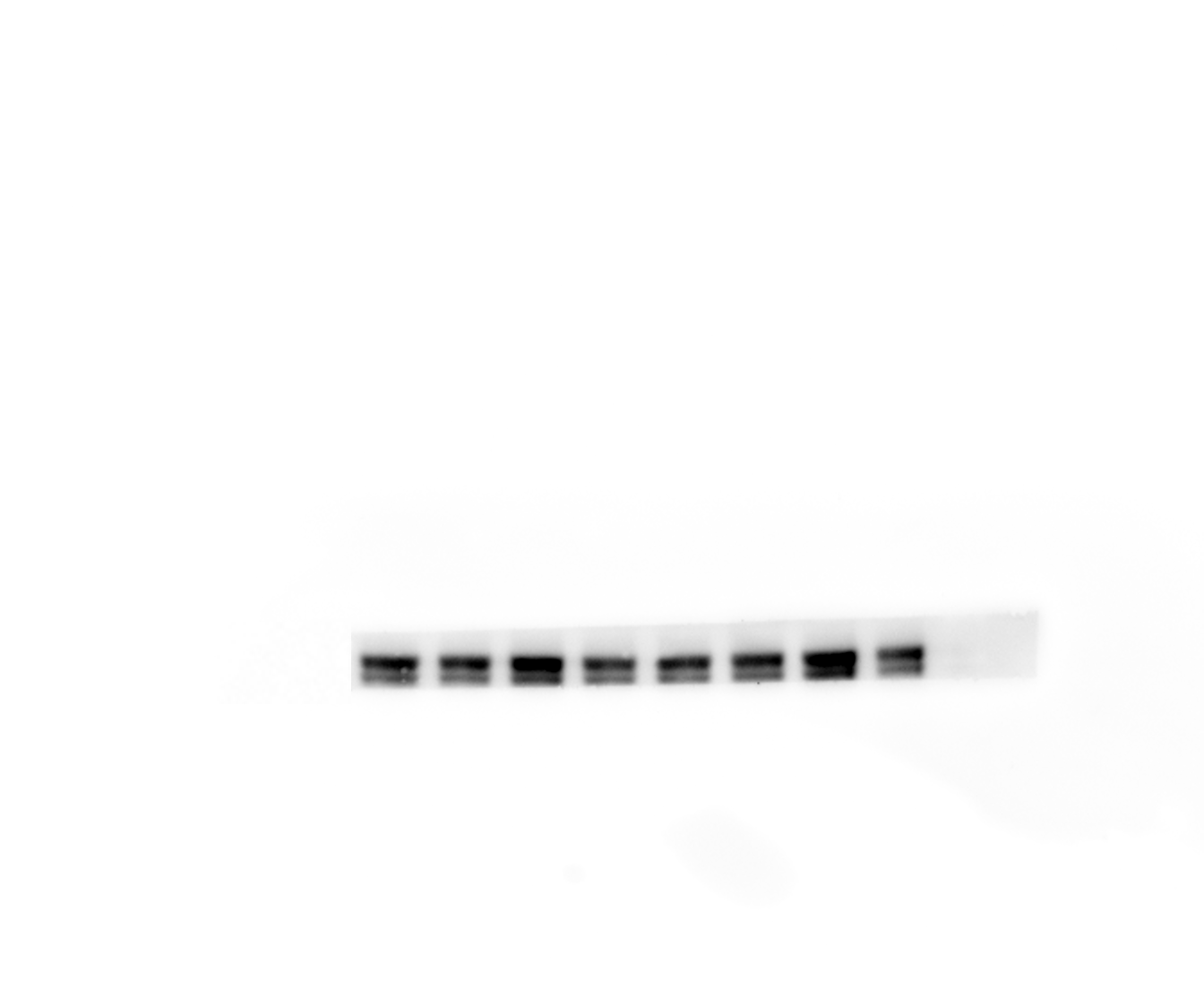

Supplement: Supplementary file 1 — Additional file 1. Raw data. [file 12935_2023_3076_MOESM1_ESM.zip › raw_data/figure7C/smad2/SMAD2 2(ΓæáSaoS-2+si-NC∩╝¢ΓæíSaoS-2+si-DIO3OS∩╝¢ΓæóSaoS-2+si-NC+TGF-╬▓1∩╝¢ΓæúSaoS-2+si-DIO3OS+TGF-╬▓1∩╝ë.Tif]

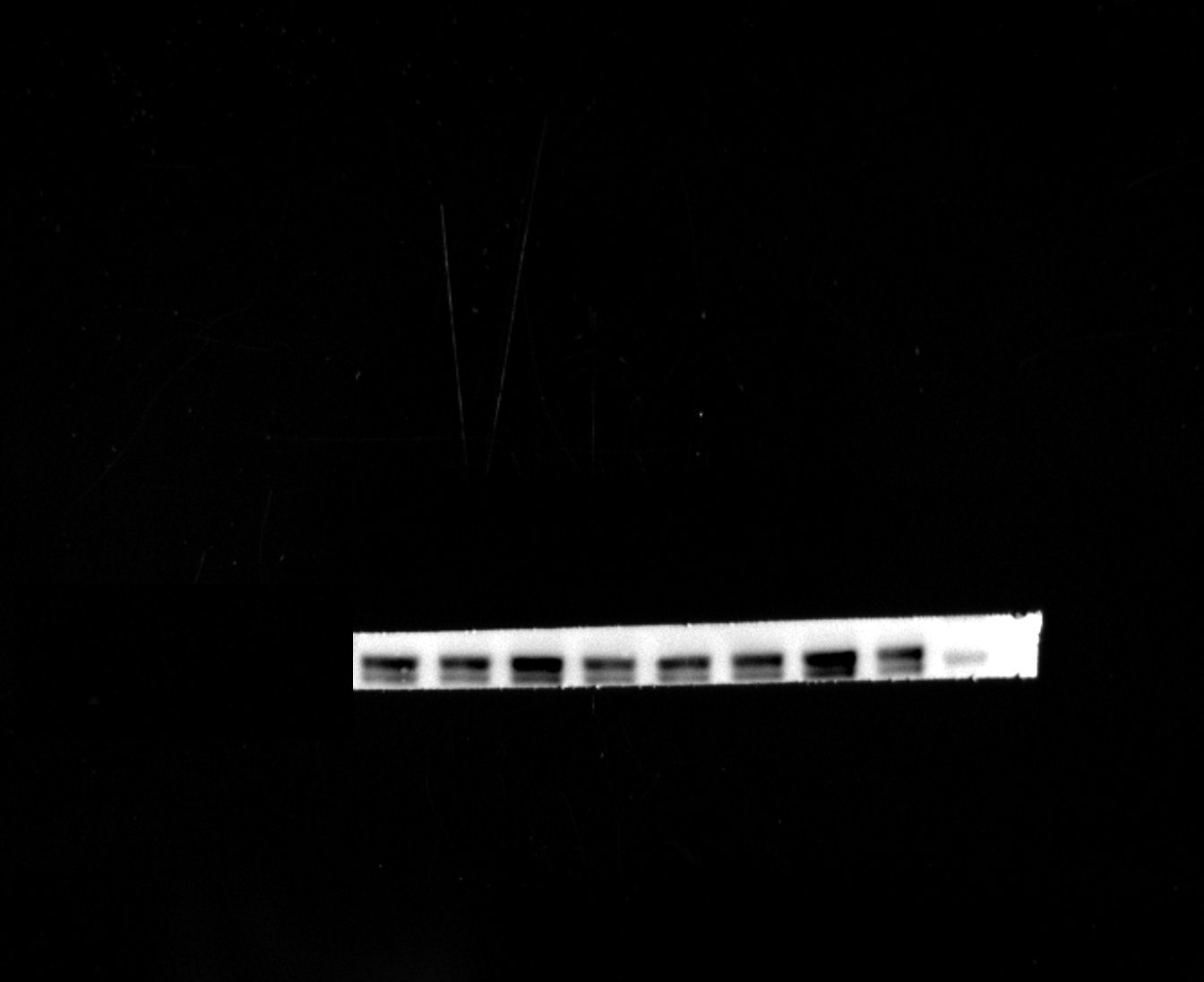

Supplement: Supplementary file 1 — Additional file 1. Raw data. [file 12935_2023_3076_MOESM1_ESM.zip › raw_data/figure7C/smad2/SMAD2 2τÖ╜σàë∩╝êΓæáSaoS-2+si-NC∩╝¢ΓæíSaoS-2+si-DIO3OS∩╝¢ΓæóSaoS-2+si-NC+TGF-╬▓1∩╝¢ΓæúSaoS-2+si-DIO3OS+TGF-╬▓1∩╝ë.Tif]

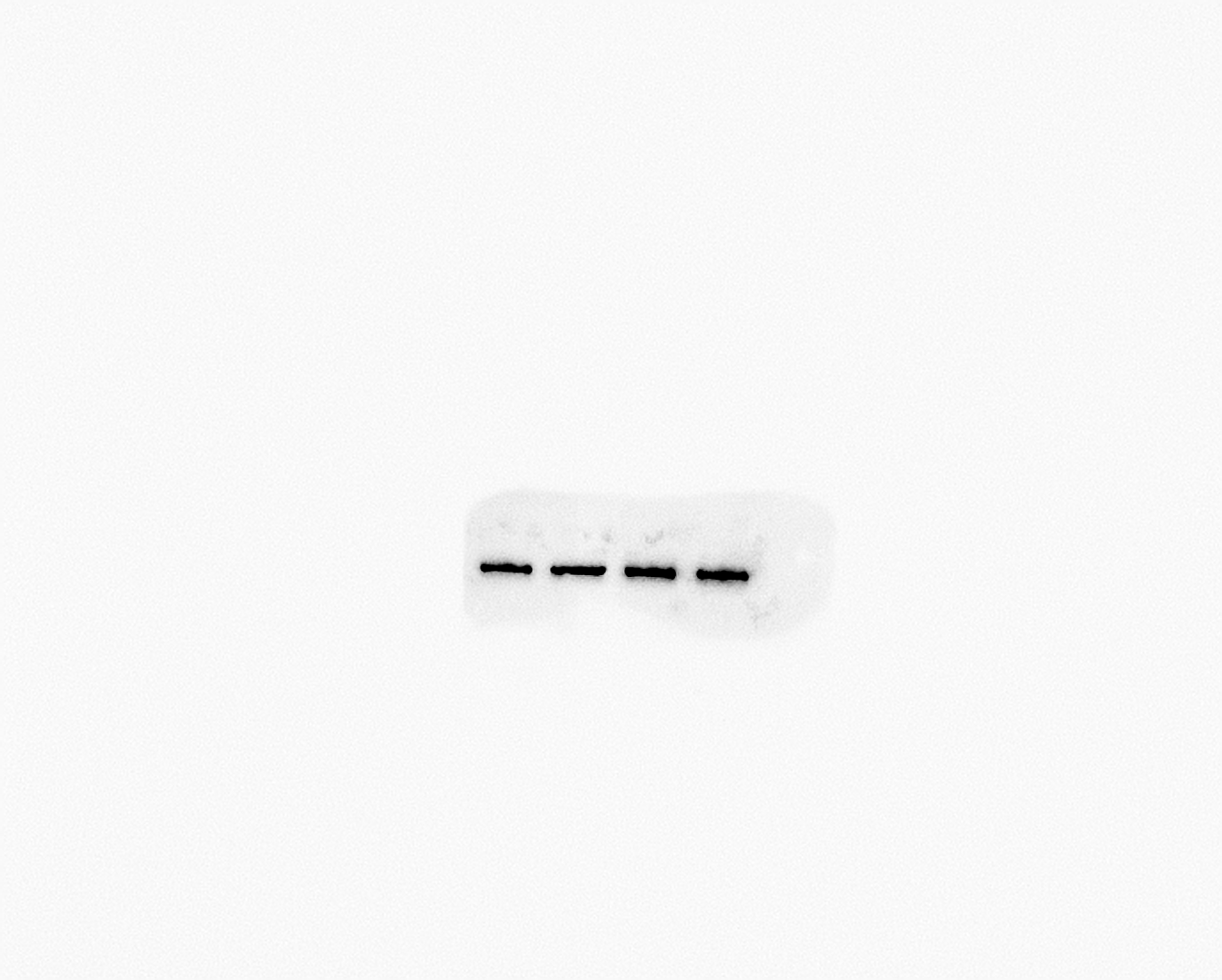

Supplement: Supplementary file 1 — Additional file 1. Raw data. [file 12935_2023_3076_MOESM1_ESM.zip › raw_data/figure7C/smad2/SMAD2∩╝êΓæáSaoS-2+si-NC∩╝¢ΓæíSaoS-2+si-DIO3OS∩╝¢ΓæóSaoS-2+si-NC+TGF-╬▓1∩╝¢ΓæúSaoS-2+si-DIO3OS+TGF-╬▓1∩╝ë.tif]

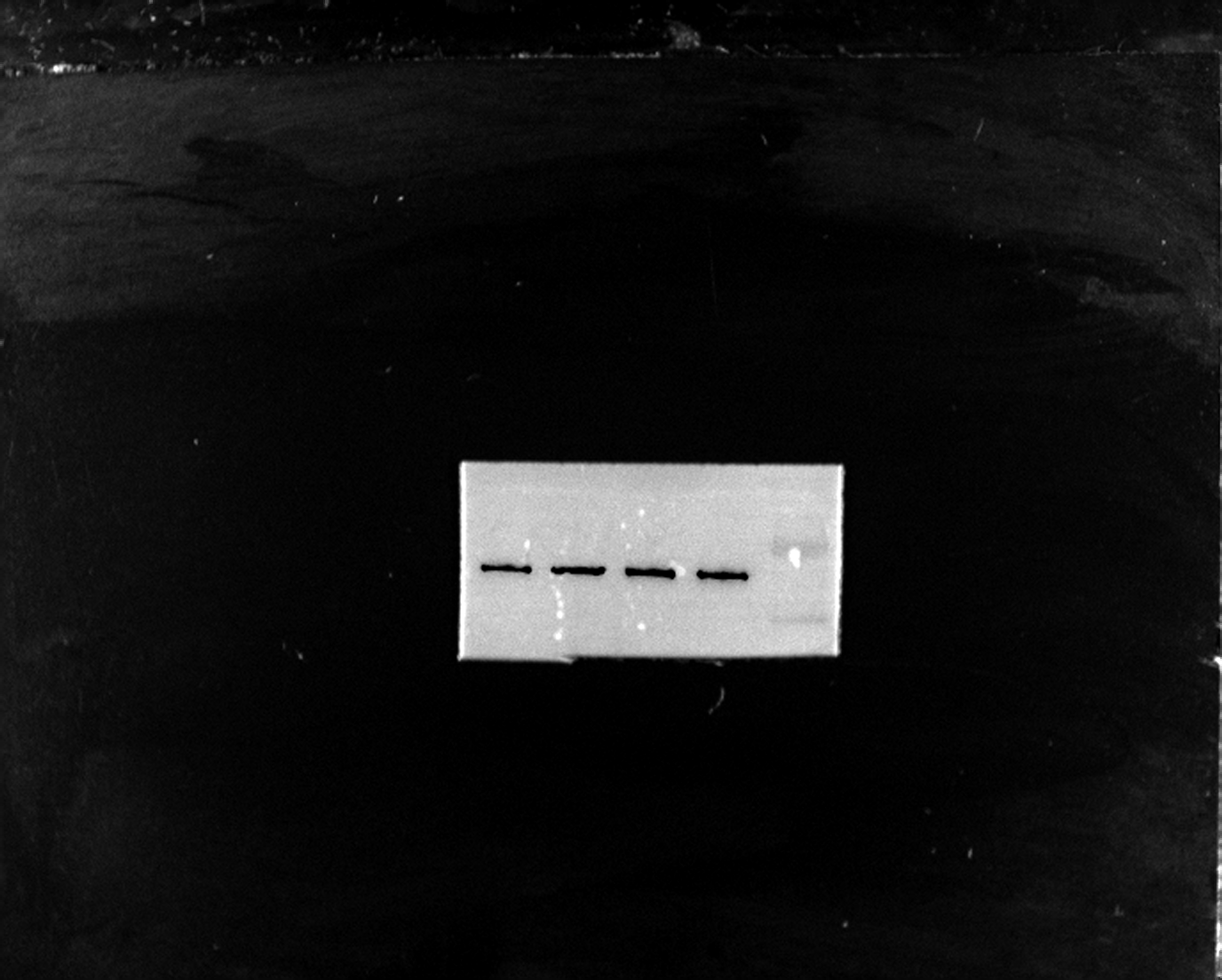

Supplement: Supplementary file 1 — Additional file 1. Raw data. [file 12935_2023_3076_MOESM1_ESM.zip › raw_data/figure7C/smad2/SMAD2τÖ╜σàë∩╝êΓæáSaoS-2+si-NC∩╝¢ΓæíSaoS-2+si-DIO3OS∩╝¢ΓæóSaoS-2+si-NC+TGF-╬▓1∩╝¢ΓæúSaoS-2+si-DIO3OS+TGF-╬▓1∩╝ë.tif]

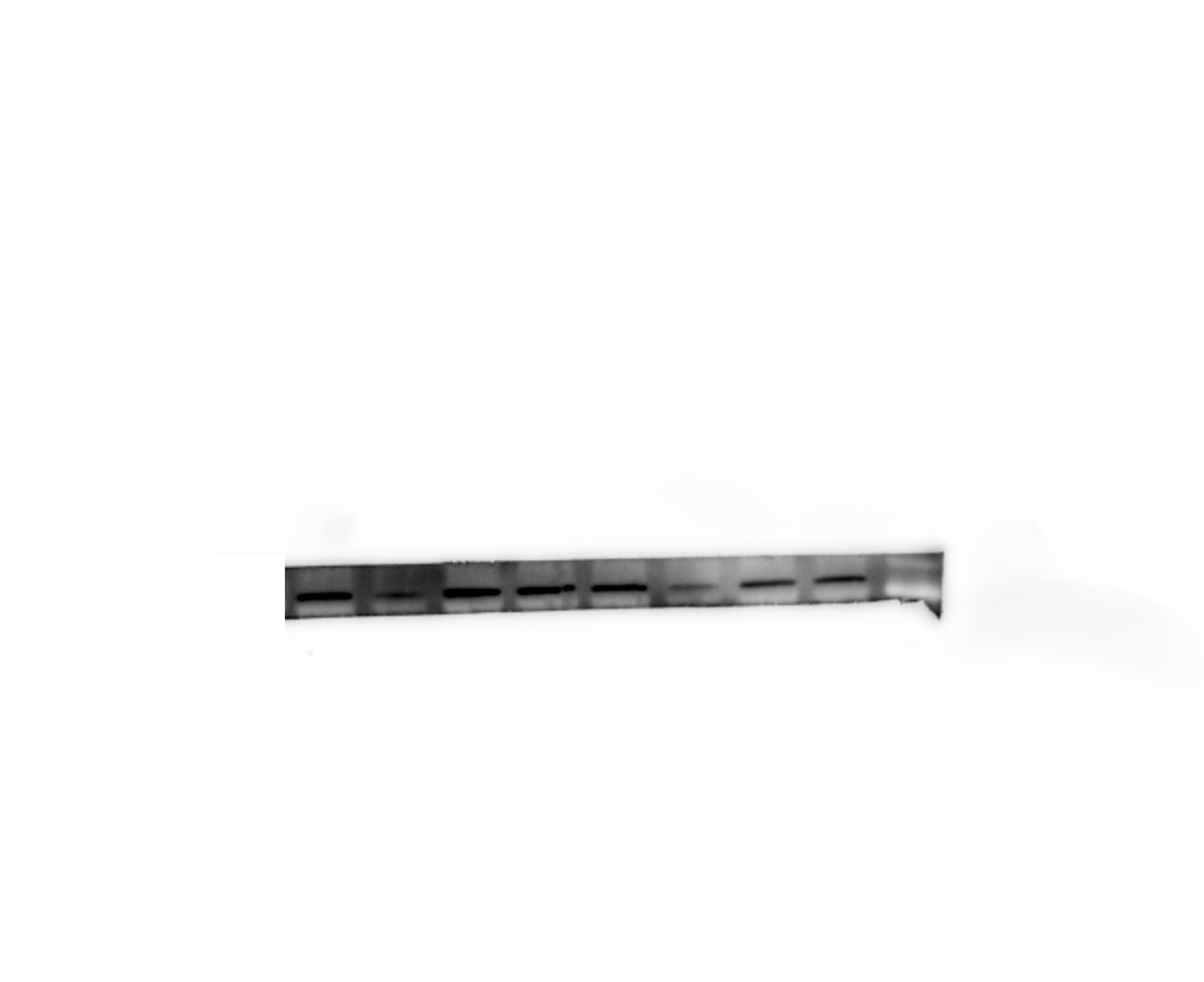

Supplement: Supplementary file 1 — Additional file 1. Raw data. [file 12935_2023_3076_MOESM1_ESM.zip › raw_data/figure7C/p-smad2/p-SMAD2 2∩╝êΓæáSaoS-2+si-NC∩╝¢ΓæíSaoS-2+si-DIO3OS∩╝¢ΓæóSaoS-2+si-NC+TGF-╬▓1∩╝¢ΓæúSaoS-2+si-DIO3OS+TGF-╬▓1∩╝ë.Tif]

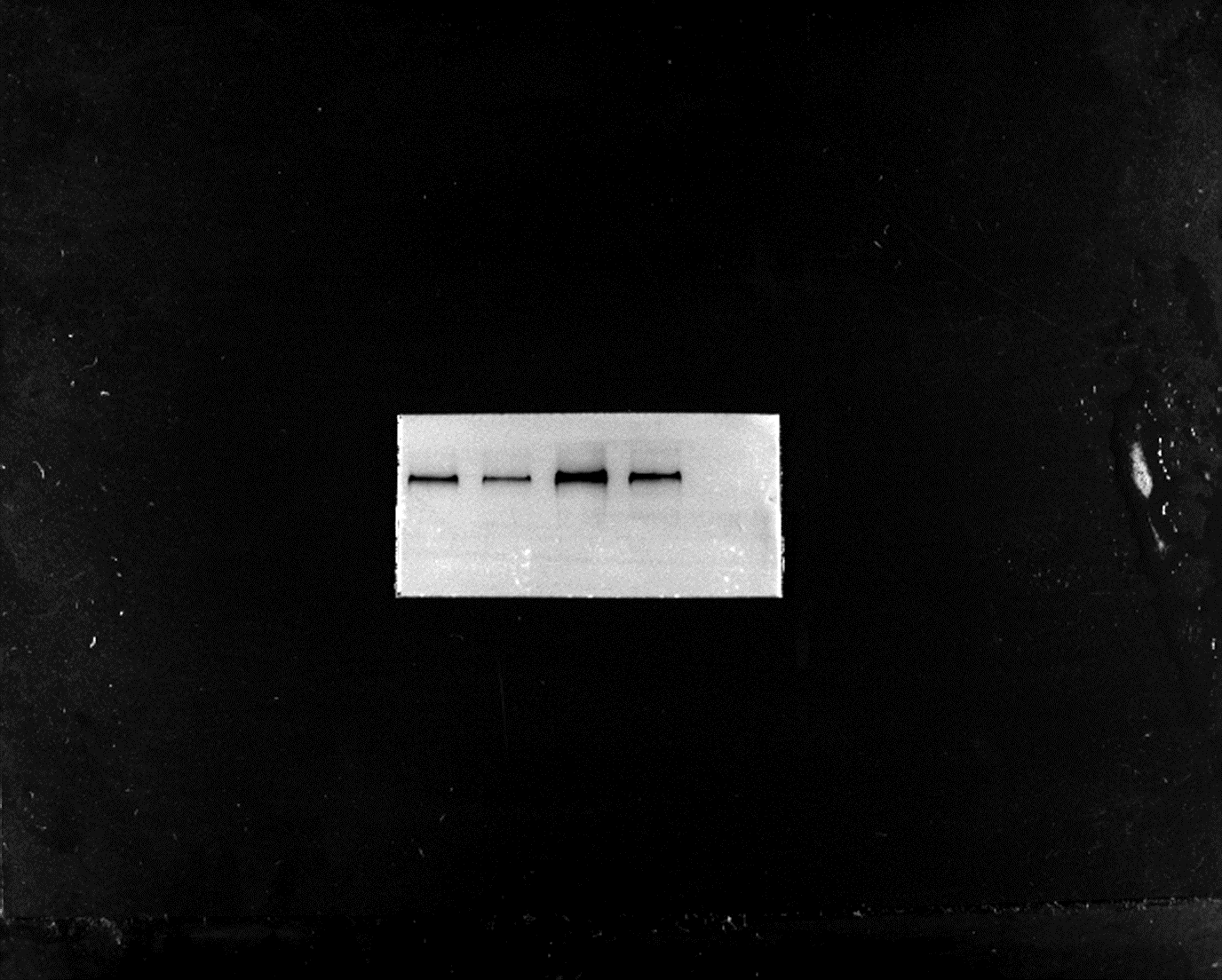

Supplement: Supplementary file 1 — Additional file 1. Raw data. [file 12935_2023_3076_MOESM1_ESM.zip › raw_data/figure7C/p-smad2/p-SMAD2τÖ╜σàë∩╝êΓæáSaoS-2+si-NC∩╝¢ΓæíSaoS-2+si-DIO3OS∩╝¢ΓæóSaoS-2+si-NC+TGF-╬▓1∩╝¢ΓæúSaoS-2+si-DIO3OS+TGF-╬▓1∩╝ë.tif]

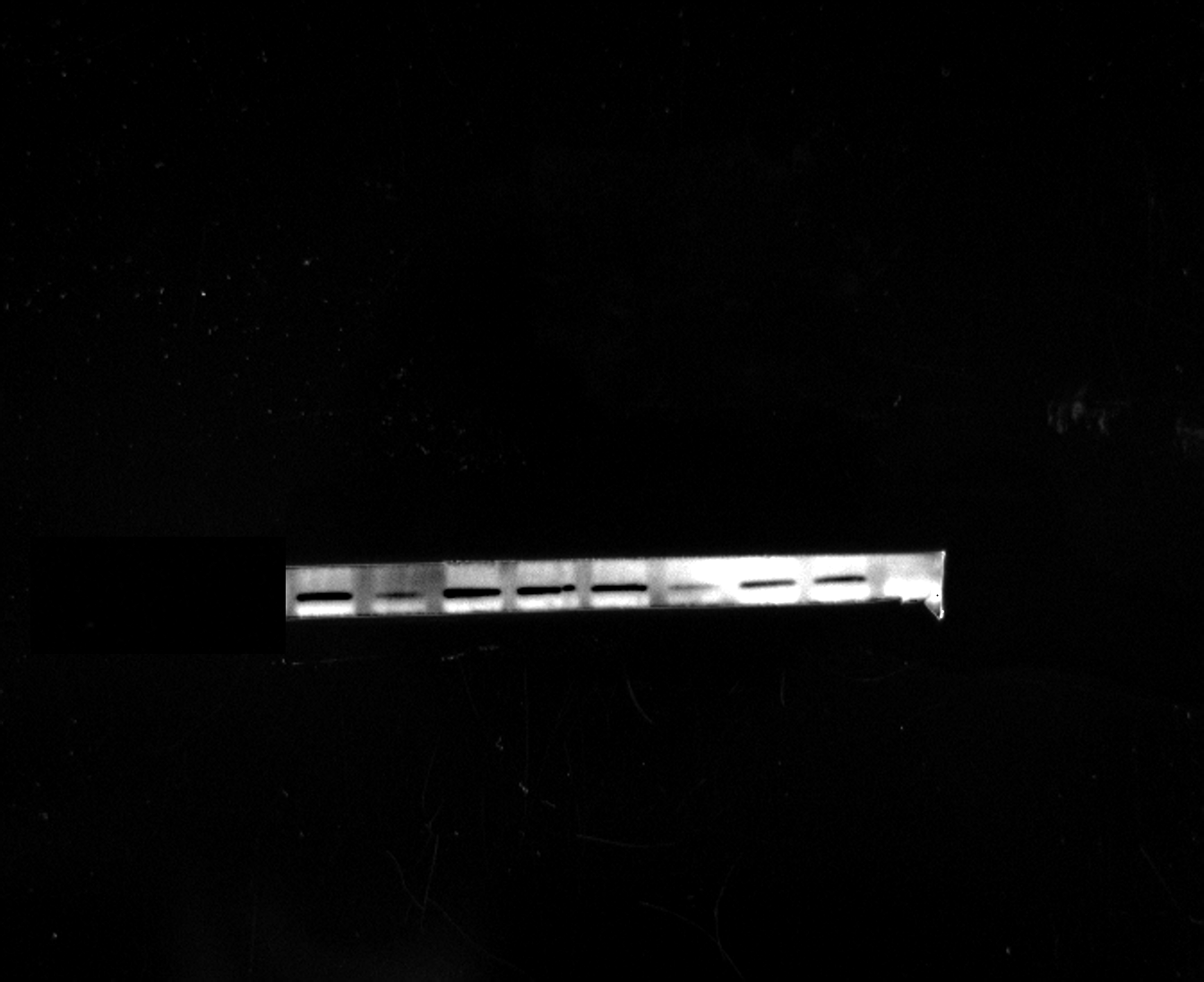

Supplement: Supplementary file 1 — Additional file 1. Raw data. [file 12935_2023_3076_MOESM1_ESM.zip › raw_data/figure7C/p-smad2/p-SMAD2 2τÖ╜σàë∩╝êΓæáSaoS-2+si-NC∩╝¢ΓæíSaoS-2+si-DIO3OS∩╝¢ΓæóSaoS-2+si-NC+TGF-╬▓1∩╝¢ΓæúSaoS-2+si-DIO3OS+TGF-╬▓1∩╝ë.Tif]

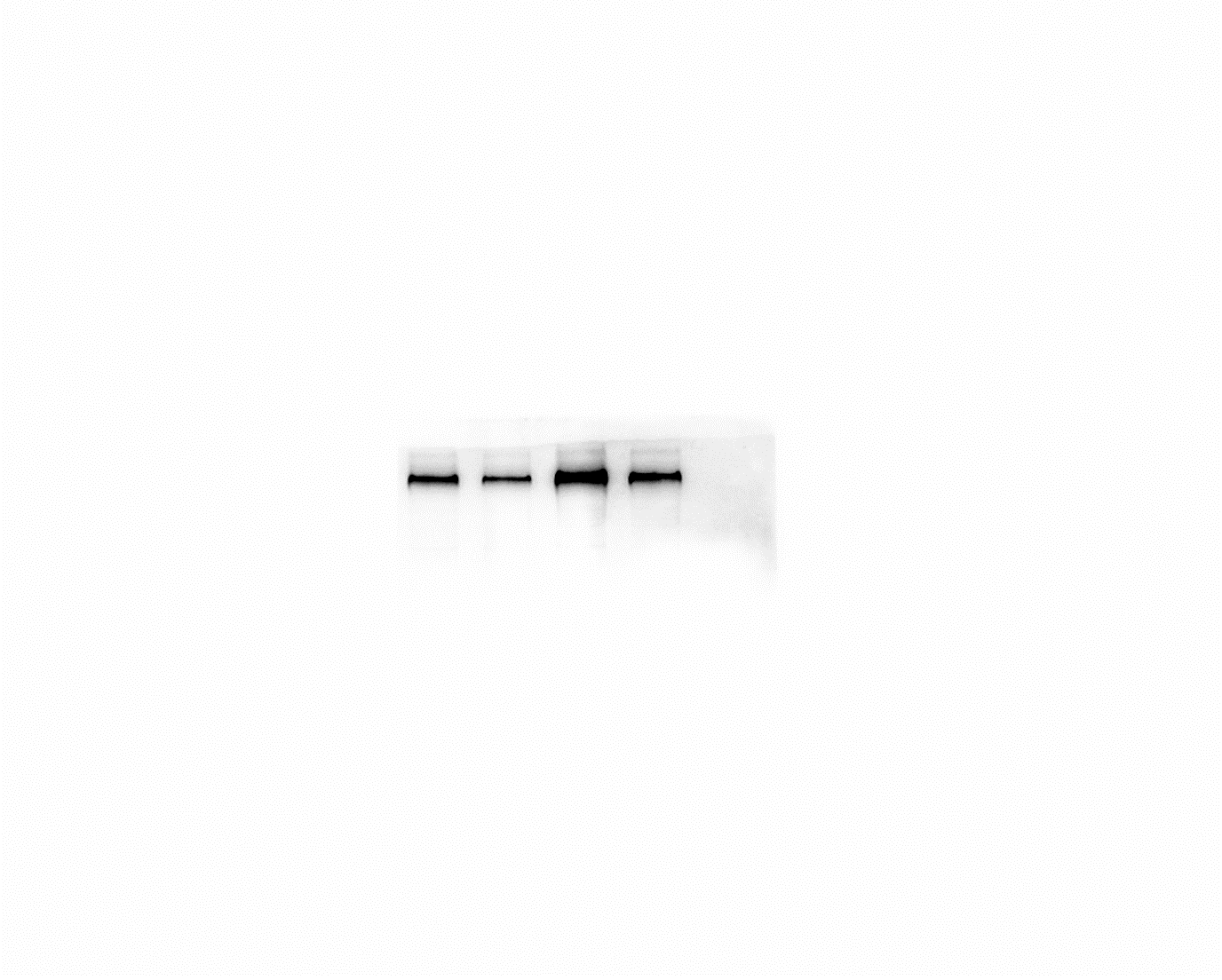

Supplement: Supplementary file 1 — Additional file 1. Raw data. [file 12935_2023_3076_MOESM1_ESM.zip › raw_data/figure7C/p-smad2/p-SMAD2∩╝êΓæáSaoS-2+si-NC∩╝¢ΓæíSaoS-2+si-DIO3OS∩╝¢ΓæóSaoS-2+si-NC+TGF-╬▓1∩╝¢ΓæúSaoS-2+si-DIO3OS+TGF-╬▓1∩╝ë.tif]

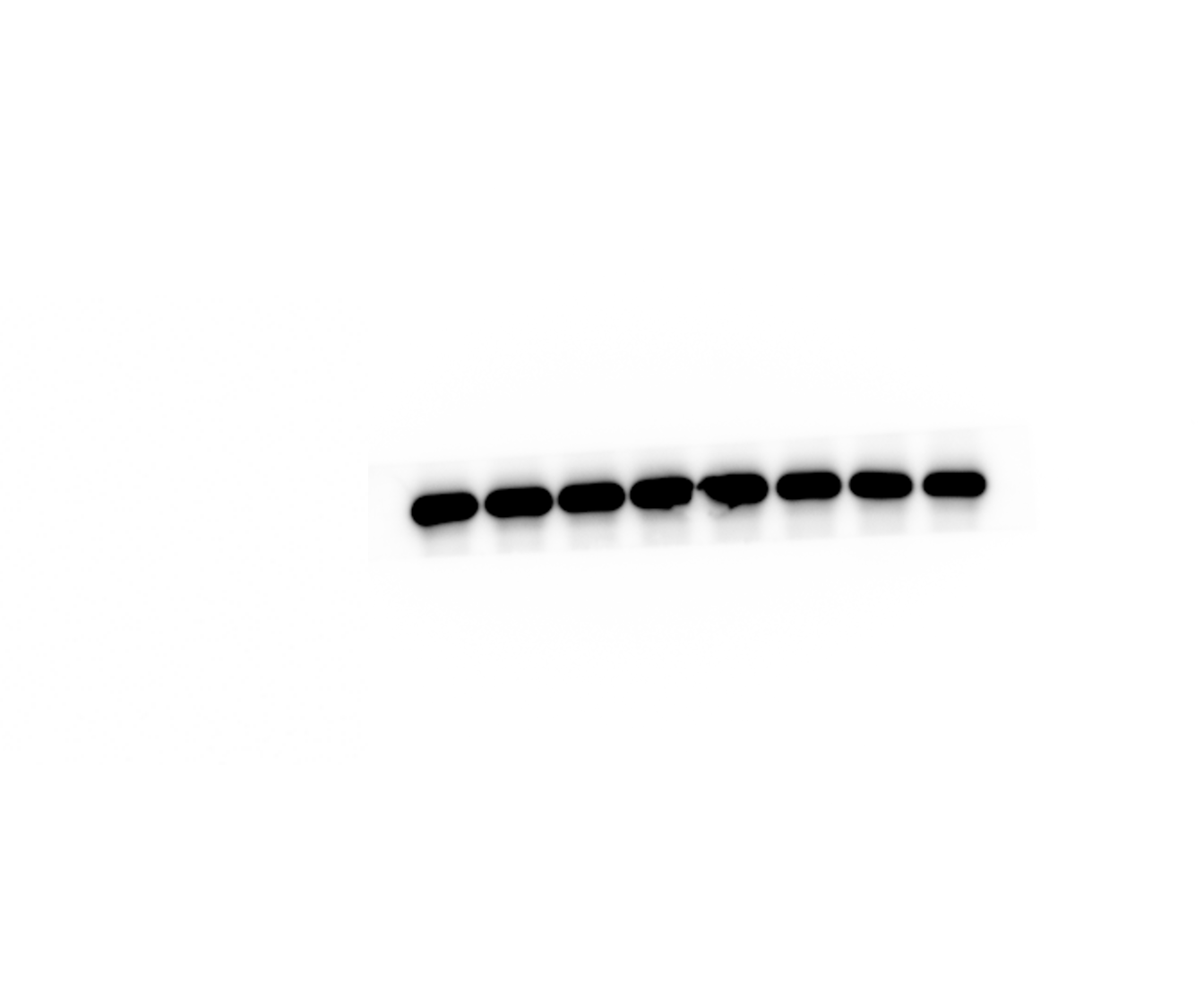

Supplement: Supplementary file 1 — Additional file 1. Raw data. [file 12935_2023_3076_MOESM1_ESM.zip › raw_data/figure7C/gapdh/GAPDH2(ΓæáSaoS-2+si-NC∩╝¢ΓæíSaoS-2+si-DIO3OS∩╝¢ΓæóSaoS-2+si-NC+TGF-╬▓1∩╝¢ΓæúSaoS-2+si-DIO3OS+TGF-╬▓1∩╝ë.Tif]

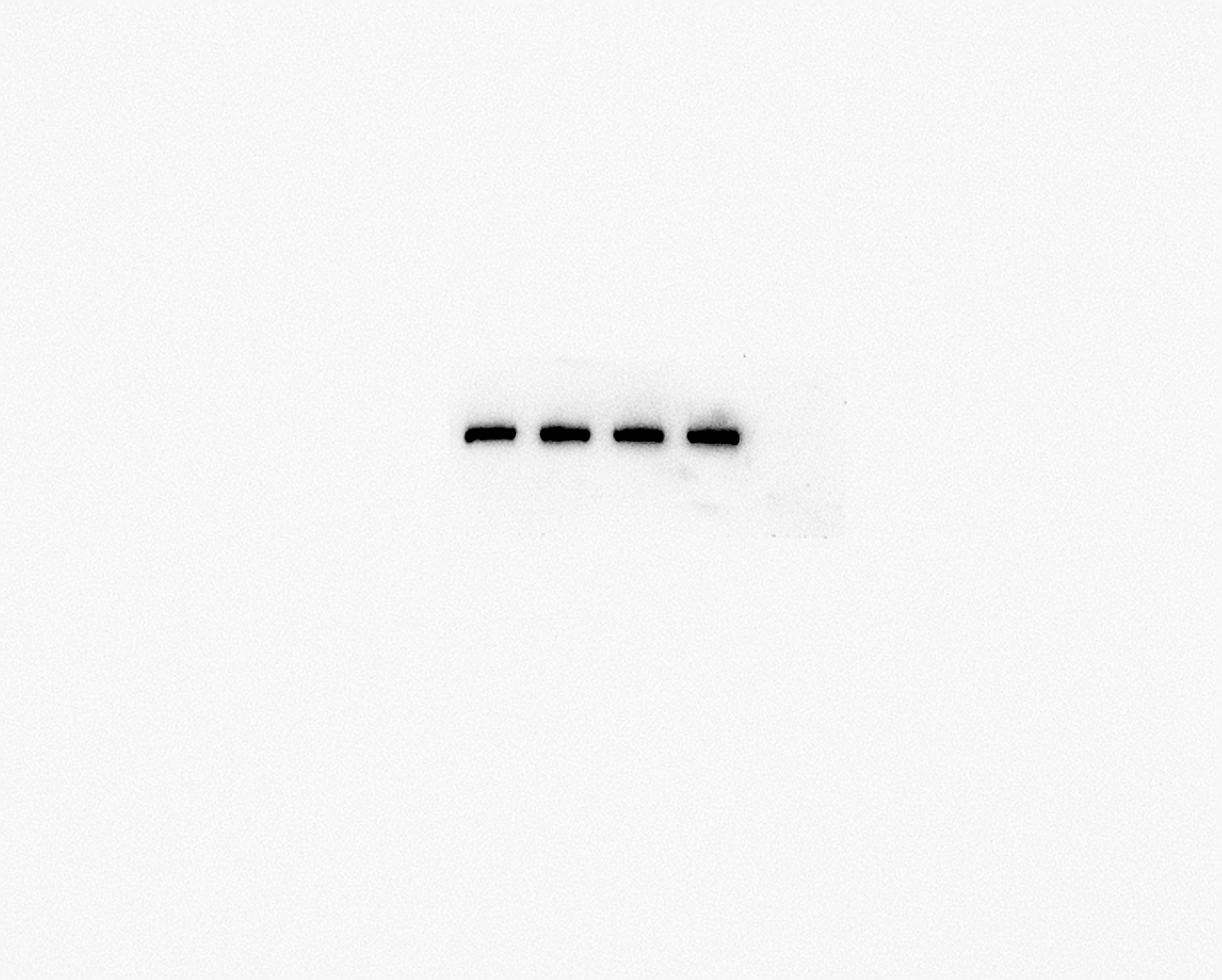

Supplement: Supplementary file 1 — Additional file 1. Raw data. [file 12935_2023_3076_MOESM1_ESM.zip › raw_data/figure7C/gapdh/GAPDH(ΓæáSaoS-2+si-NC∩╝¢ΓæíSaoS-2+si-DIO3OS∩╝¢ΓæóSaoS-2+si-NC+TGF-╬▓1∩╝¢ΓæúSaoS-2+si-DIO3OS+TGF-╬▓1∩╝ë.tif]

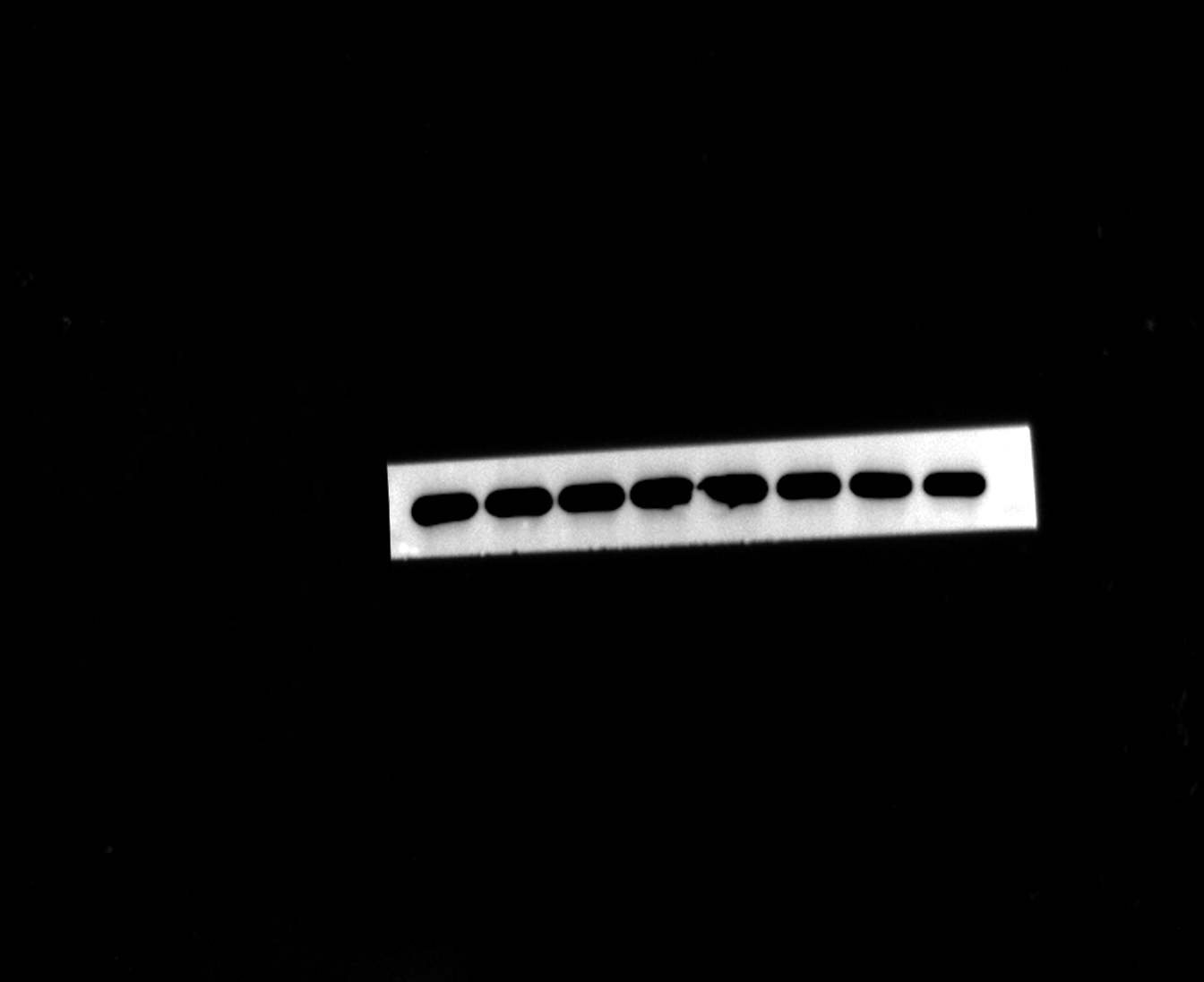

Supplement: Supplementary file 1 — Additional file 1. Raw data. [file 12935_2023_3076_MOESM1_ESM.zip › raw_data/figure7C/gapdh/GAPDH2τÖ╜σàë∩╝êΓæáSaoS-2+si-NC∩╝¢ΓæíSaoS-2+si-DIO3OS∩╝¢ΓæóSaoS-2+si-NC+TGF-╬▓1∩╝¢ΓæúSaoS-2+si-DIO3OS+TGF-╬▓1∩╝ë.Tif]

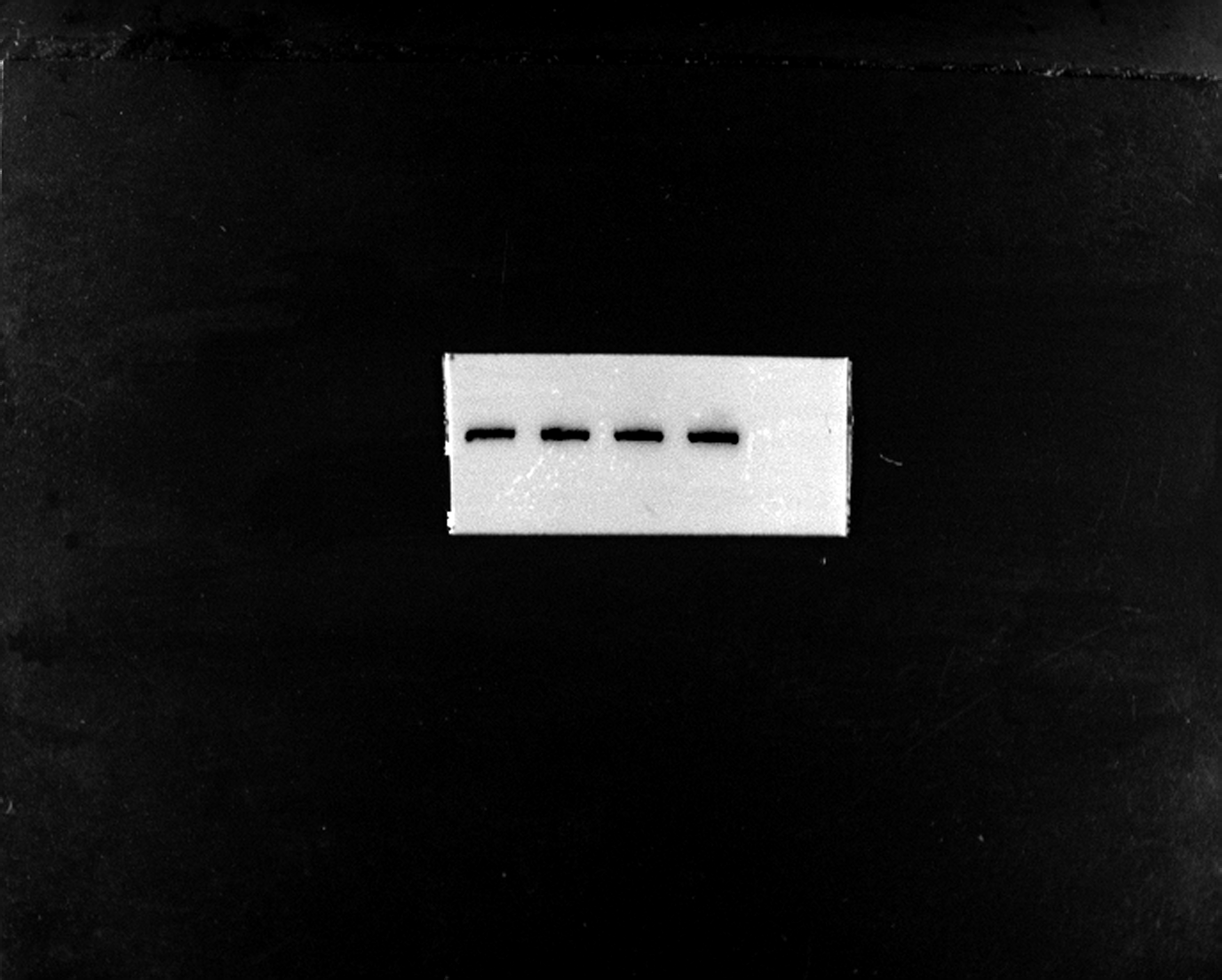

Supplement: Supplementary file 1 — Additional file 1. Raw data. [file 12935_2023_3076_MOESM1_ESM.zip › raw_data/figure7C/gapdh/GAPDHτÖ╜σàë∩╝êΓæáSaoS-2+si-NC∩╝¢ΓæíSaoS-2+si-DIO3OS∩╝¢ΓæóSaoS-2+si-NC+TGF-╬▓1∩╝¢ΓæúSaoS-2+si-DIO3OS+TGF-╬▓1∩╝ë.tif]

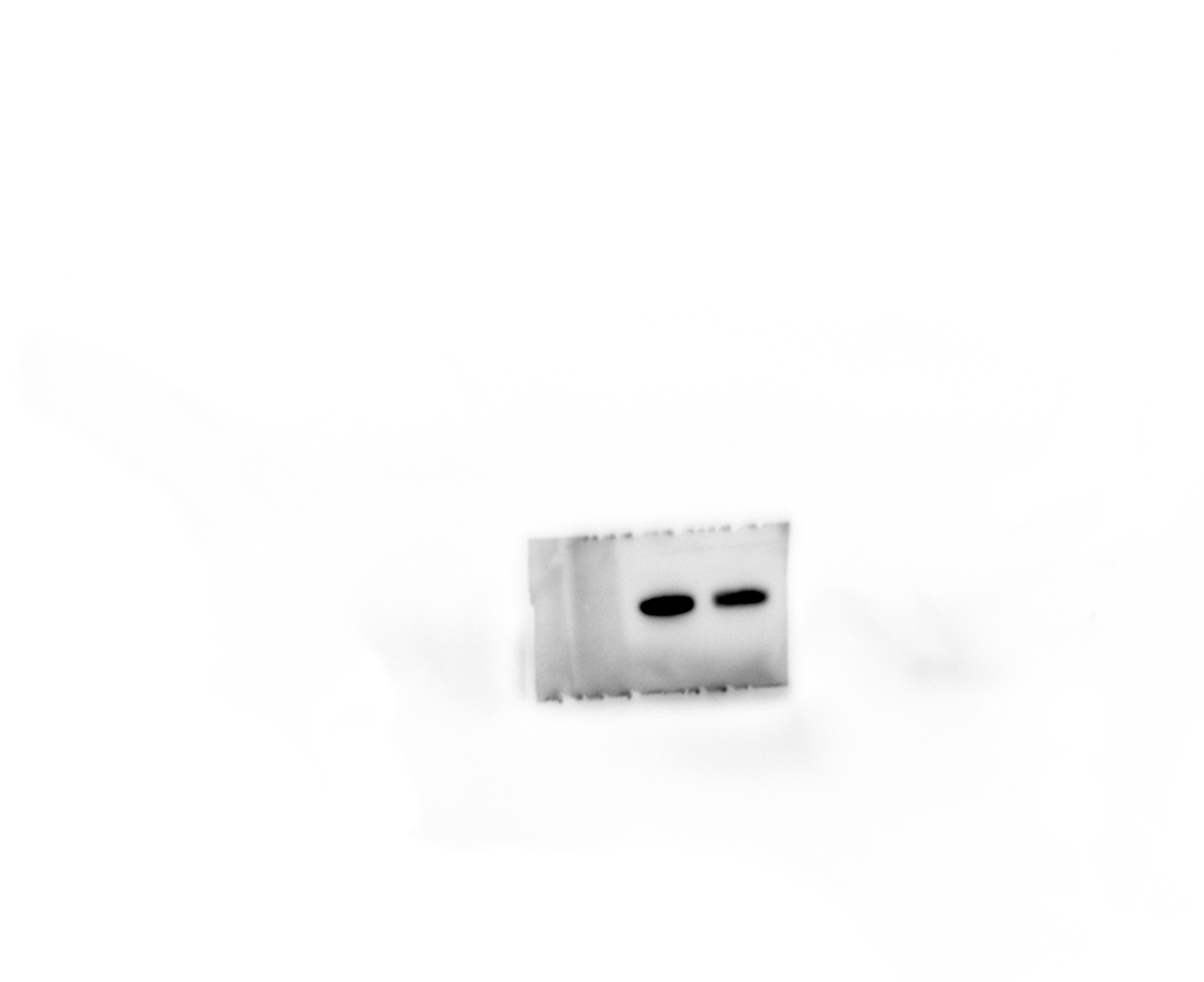

Supplement: Supplementary file 1 — Additional file 1. Raw data. [file 12935_2023_3076_MOESM1_ESM.zip › raw_data/figure6-gh1/Figure.6 Vimentin/raw_H1.Vimentin(ΓæáU2OS; ΓæíU2OS+si-DIO3OS).Tif]

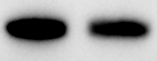

Supplement: Supplementary file 1 — Additional file 1. Raw data. [file 12935_2023_3076_MOESM1_ESM.zip › raw_data/figure6-gh1/Figure.6 Vimentin/final_H Vimentin.tif]

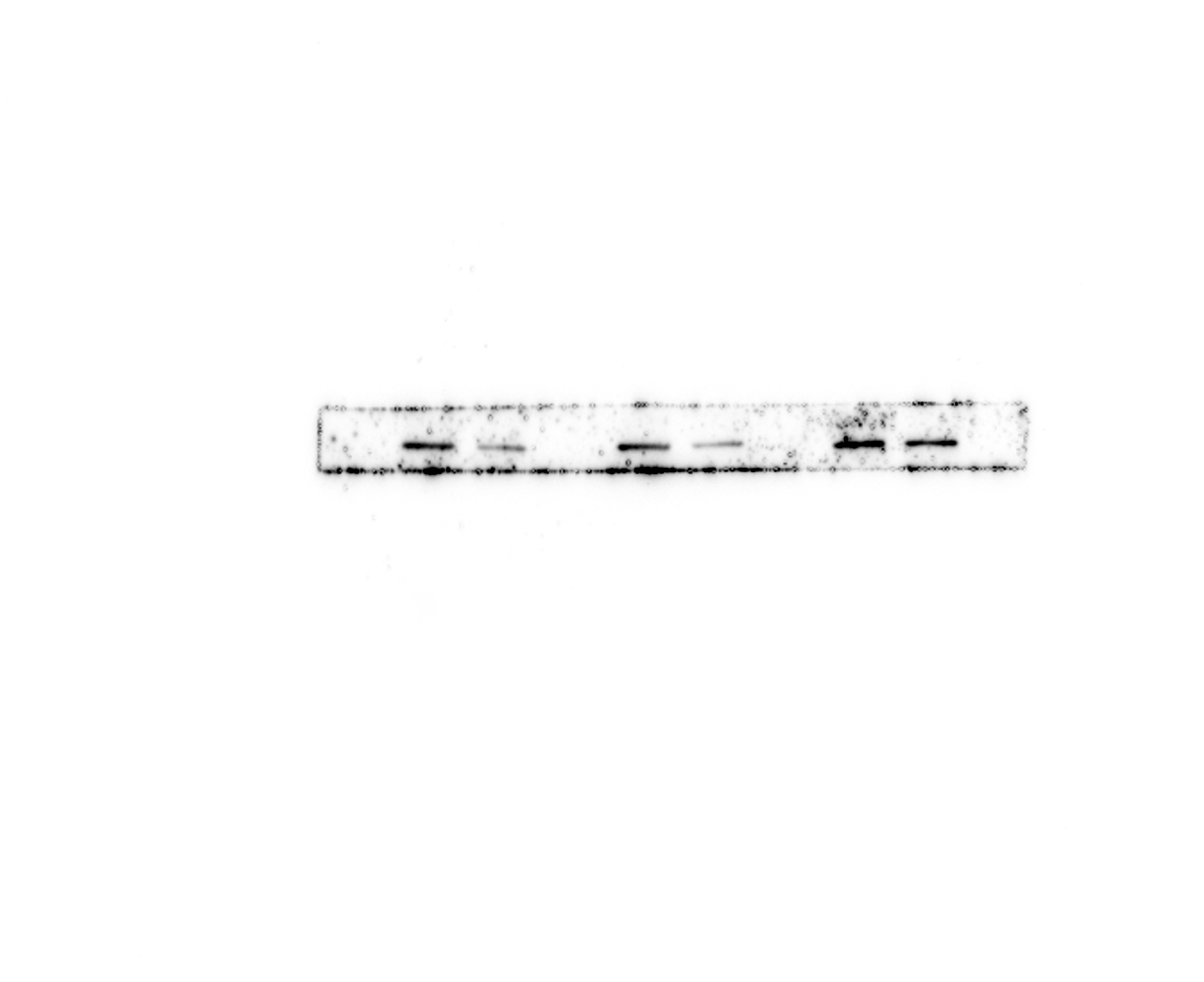

Supplement: Supplementary file 1 — Additional file 1. Raw data. [file 12935_2023_3076_MOESM1_ESM.zip › raw_data/figure6-gh1/Figure.6 Vimentin/raw_G.Vimentin(ΓæáSaoS-2+si-NC; ΓæíSaoS-2+si-DIO3OS).Tif]
