# Supplementary material for: Long intergenic non-coding RNA DIO3OS promotes osteosarcoma metastasis via activation of the TGF-β signaling pathway: a potential diagnostic and immunotherapeutic target for osteosarcoma
Source: Cancer Cell Int. 2023 Sep 26;23:215. doi: 10.1186/s12935-023-03076-5 (PMC10521498; doi:10.1186/s12935-023-03076-5)
Supplement: Supplementary file 3 — Additional file 3: Table S1. Sequences of Ribo TM h-DIO3OS Smart Silencer. Table S2. Primers and sequences used in this study. Table S3. Baseline clinical characteristics of patients with osteosarcoma in validation cohort. [file 12935_2023_3076_MOESM3_ESM.docx]

**Additional file 3**

**Table S1. Sequences of Ribo TM h-DIO3OS Smart Silencer.**

| Cat. No | Name | Target Sequences |
| --- | --- | --- |
| lnc3210428014954 | Ribo TM h-DIO3OS Smart Silencer | ACCTCGGGACTCCATAATAT |
|  |  | CCCAGAGCTGTCCTCAGCAT |
|  |  | GACCCAATAAACCTTTGTGA |
|  |  | CCACAGAATACACTCCACA |
|  |  | CAGTGACTAACCCAAACCA |
|  |  | GATGGATCCTCTACAGTGA |

**Table S2. Primers and sequences used in this study.**

| Primers for qPCR | | |
| --- | --- | --- |
| DIO3OS | F | CTGGAGGAGAGTGCTGGTGAATGG |
|  | R | GGAGGGGACAGAGACAAACGGC |
| GAPDH | F | ACAGCCTCAAGATCATCAGC |
|  | R | GGTCATGAGTCCTTCCACGAT |
| U6 | F | CTCGCTTCGGCAGCACA |
|  | R | AACGCTTCACGAATTTGCGT |

**DIO3OS sequences:**

CCGCTTGCCTCGCCCAGCGCAGCCCCGGCCGCTGGGCGCACCCGTCCCGTTCGTCCCCGGACGTTGCTCTCTACCCCGGGAACGTCGAGACTGGAGCGCCCGAACTGAGCCACCTTCGCGGACCCCGAGAGCGGCGGCGCGACTCACCGCGGAGGCGCCCGGACGCGTCAAGTGCGTACTTTCAACCGTAAATGGGTGCTGAGCGTTTAGGGGTGCGGGGCAACTCCCTAACTCGTGGGGCCCTGGCCGGCCCTGCCGCGGCGTCCGAGACCTTCTGATCCCTCGCTGCGCGCGGCGCGGGGTTGGCGACTTGGGCTCAGGGGCGGCGGGGCGGGGAGGTGCCTTGACCTAGGGACCCAGAGGCCGGGAGCAAGTCTCCTTTCGCCTTGGACTGCAGTGTCCCCCAGGGCAAGGCCTGAACCAGGTCGCGAGGGTCTGTCTCCCGTCTGGGGAGCTGAACTGCCCGAGACACTGTCGCAGTCGCGGGGACTTGTCTGGAGCGGCGGGGTCTGAGCCCAAGTCGGAAGCCGCCGCCGCCGCCAGCGTCCCGTGCGCCTGCCGCCCCGGGACCAGCCGCGAAACTTCTTCTCGGGACGGAGACAGCGCTCGCCGCCCGGGCCGAGGTAGGAGCTGGCTGGGCTCGGTAGAGCGCGGGCGAATCGCTTTCGGCAGCGAAGGAACGTGTCGGGAGAAGCCAACTGCAGGACCAGGGCCCCAGCGGGCGCGCAGAGACGCCTGCCTTGGGCGGAGGTCCCCCCCTCCGGTTTTCGTGGCTCTGGTAGGGCCCTCTGTCCCGTGAGCTCCTCACTGGACCTGAGGCTACAGGCTTCAGCAGGTCAAGCGCCCGAGTGTGCCTGGAGGATTGGCGGCGGGGTGGCATGAGAAGGTGGCCCCTCTTCTCCATCTCCCAGTCAGGCGATTTCTCTGCTGTCTGGGGCAGGGGCGCACACCCCAGGAACTTACTACGTAGAGGGAAGCCAGCCTGGTCTCTCCCCCTTGCGTCAGTGCTCCTAAGCTGCCCCTCACCACTCTGGGTAGGGGTCTTTCAGGTGGGCTAGGGAATTCTCCTTGGAAGTCCCCTTGTTTGCTCCCCTCCATCAGGGCACCTCTGAGTTAGCCAATCATCCTCTTTTCTGGCCTCTTTTTTGCGGGGAGGAGAGTAGAGAGAGAAGCCTCCTACTCGGCAGGTCTCTGAGAGGAGGTTTGGGGGGGCTTCTGAGGTTGCTCCTCCTGGGCAGAGTCAAGGGGTGTTGAGGGTTCCAACTTTTACTGGACAATCTCTCAAGCTCTGATTCCCTGATTTCTGGGAGGTGTCAGTGTAGGGCTTGTGTCCTGCCCCACCCACACAGCATTTGGTGCAGGCACATGGGATGGTTCCCCAAGCCCTTTTCCCACAGTTAGTTTCAGAAAATGCATAAGCCACCTAAGGCTGGGAGAGCCTTTCTGTGCTGGCTGAGATCTGGTGGCATGTGCATGCATATGTGTGCAAGTGTGTGTGTGTGTGTGCAAGTGTGTGTGCGCATGTGCATGTGTGTGGAAAGTATGGTCTGGGTGGCTGTAAGACTGGCCCCTCTTGTCCCCTCTGCAGGCCCAGCCCAATAGGAAGCACCTGATCCACACTCTTTCTAGCCCCAGGGATGCTCAGGAAAGCAGCCTTTCTTTAGGGCTGTCATCTGTTCTGGGGATCTGTTGCTGTTTCTTGGGCCCACAGAATACACTCCACACCTCGGGACTCCATAATATTGGACAAGGCTGGAAGGCCCATTTGGCTCCTGCAGCCTCTTATGCAGGTGAGGACAGACCCAAGGTCCAGGCAATGCTGGCCTTTGGAACACAGAACCTGGCTCACTGACTCCCACTCCAGTTGTCTTTTCCCTCTGCCTCTGGAGCAACCCTCTCTCCTCCAGAGCAACCCCTGTCCCTCCTTCCTACCTCTCTCTAGGGCTGCACCCCCAGCTCACCTTAGACCTGGCCCTTCCTACCTGTTGCCCCTACTGTCTTGTCCCATCCCAGTGGTCAGGAGGGAGAGTACTGGCCAAAGGCCCAGCTCCACCTGTGACTTAGGGGAGTGCCAGGCATCACCCAGAGGTCCCGGCCCTCGGGGGCCTGGCAGGCTCTGCTGTGGGGGAAGCAGAGTTAGAACTGGGGCTGCTTCTCCTGTCCCCCAGCCTGGCTGGGCCTCCCCCACCTCCCTTTCTGCCTGCCCTCTTACAACTCCAGCAGGGGGCGGGCAGGGGAAAGAAAAGGAGGGACAAGTTGGATGGGGTCCATTTGGTGTCCAGGAACAACTTGGAGAGAGGCGGGGAGGGGACAGGCAGCAGGGCCCTGGCAGAGCCAGGGGAGGGCTGGCCTTTGGATGGCCTTCTGATCCCAAGCCCATCCTCCTGGGGCAGTCTTCCCTTGAAAGGGAGGGTTTGGTGAACCCTGGTGCGGACCCCCCCGCCGGACCTCACTGCCCCAGTGCGGGGCGTGGTAGGCGGGTTTGCAATGGGTACAGCCTCTCTCTCCATTCCAGTAGAGCAGCTCCCAGGGCCCAGAGAGGCTGTCACTGAGACTTCCCTCTGGGGTGGGGTGGGTTCTGGTGGCCCTGTGTTCCCCTGCAGGAGGACCTCCTGAGGCTTCGGGCAGGAGCTGGTGCACACCTGGAGGAGAGTGCTGGTGAATGGCAGGTGTGTGCCTCAGTGTCCTTGCTGCCAGGGTGTCAGCCCTGCTTCACCACTGGCCGTTTGTCTCTGTCCCCTCCACTGGCCTCTTGAAGCACAGAGACCCTCCGGGTACTTTCCTTCCTCTGGGCTTCCACTCATGCTATTCCCTGCTCCCTTTTTCTACCTGGAGACCCCCATTCCTCCTCCTCCTCTGCTGCCCTCACCTCCACCTGGGCCCTCCCAGCTCCCGGGAGATGGCTGAACCCCACTCCCCACCCGCTAGTCATCCTCTTCAGCGGTCCTCCCAGAGCTGTCCTCAGCATGGCAGCCTCCACAGCCCCTGGGTGTCACCCCCGGTTTCGTGTCTGCCTCGGACTCCAGACCTGCCCCTCCCAGGCTGGCCCAGATGGATCCTCTACAGTGACTAACCCAAACCAGCACCAGGCAGGGACCCAATAAACCTTTGTGAATGGAGTAAATGAGTGCGGTGTGTACTTACTGAAAACTCCTTGTTTGGGGAGAAACTCAGGGTATTTCACTCTGTCCTTTTGCGCACCTGCAGCAGTGAGCTGGGGCGGCATGTTGGCAGCCAGGGCTGAGCACGGTGGGTCAGGACCTGCCGCTGTGAGCAGACAACACTTCCTGGGAAGTCCCCTTGGGGCGTGGTGTCTGGTGAATGCACCCATCACCTGGTCACTGGGCCAATCCGTTTCCCTTTCTGGGCTAAACAGGTCTTCCCACGGGTACCTGGGCCCTCCCACGGAGGTATTTACCCTTCTCATTTACTTTAAGAATGAATTCTTCTATCCAAACACTTGGCTAGGTTGATCATGGGGACACCCTGCAGGGGATGGGGTGGGAGATGGCTGAGGGACCCAGAGCTCTGGTCTGAGCGGTGTCCTCAACATGGCAGACCTCCATCCCTCTGGGAGTTTGAGGCAGGCACAGGGGAGGTGGCGGGACTCTGGGGTGACTGGGCAGGCTGGGATCCCAGCCAGGACAGCTTGTCAGGGCCCCAGGGCCTGGATCTAGCAGAGGTGTTTGGCAGCATTTGGGGGTTGGAGAAGGTCTGGGGCTCCCGGCCAAGGAGAGGAGAAGGCCTAGGAGGCAGTGCCCTGGCTACTTGCTGTGTGACCCTGTGCATCTCTGGCCCCTTCCTCAGGTCTCGTCTGCATCAGGAGTGGGTTGGGTGGTGACTTTCCAGTGCCTTCCCCATGGGTCATTCTGCACGCCAGAAGGTCCTAGGAAGTTCTAGGAGACGCTATTCTGGCCCTACCTGCTGTTCTGCGGCTGCTGAGAGCCCCACTTGAGGACGAAGGTGCCCCAGTGCTGGTCCTACCTGGCCTCATGCTCTGCCACTTCCACCCAGTACCGCCTTCGCCTGCCCGCTTCCTGCCGCAGCTCAGGGCCCTCCAGCCCGGCCTGGGCTCTGACTTGTTCCTGGCATGAGTGCTACTGTGCTCCCTGGTGCTCAGCCAGACCCCAGGGCACAGGATGATGCCTTGCCAGAGGTTGCCTGTGGGTGCCTGGGTCCTGCCTGGAGCTGGGTGGGGCACTGGGATGCTGGGCTACTTGTGCCAAGACAGATCTTGCTCTGAGCCGGGCCCTGAGGCTGGCTGGAGGGCCACCTTGCGAGTCAGTCCCTCCTTTCCATCTGTGATCCTCTCTGGAAAAAGGTGGGGTTCAACCGACCAGCAGATAGGCATCCCAGGTCCATGTGCTTCTCTGAGCCTCAGTTTCTTCACTGGAGAGGCGGGCCTAATGTTTGAATGTGTTGTGTTCACCATGACATGAGGGGCAAGTGTAAAGTCTATATGTGTGCCTGTGTGAGTTCGTGTGTGTGTGTATGTGTACGTGTGTATATGTGTGTGTGTATGTGTGTGTATGTGTGTATGTGTATGTGTGTGTATGTGTATGTGTGTATGTGTATGTGTGTGTATGTGTGTATGTGTGTATGTGTGTGTACGTGTGTGTACGTGTGTATGTGTGTATGTGTATGTATGTGTGTGTATGTGTGTGTATGTGTGTGTATGTGTATGTGTGTGTGTGTGTGTGTGTGTGTGTGTGTGTGTCTGTTTACTTAAACCCAGCCTTGTTCCAGAGTGGATTTCAGGATTCCTCTAAGAAGCTTTGGAATGCCAGAAGATACCACAAGTGAGAAGAAAAAATAAAACAAGAGGAAGGATATACTTCCCATAAGGGGACAAAGTACAGCCGCAGTGTCCTCCGCTTGGGCCATGGAACAGCTGCAGGTTTTGCCCTGATTACCCTGGTGGTAAAGTCTGATTGGCTATGATTCCCGGTGTCCAGAAGATAAACACGGGCGGTGCAGCGGAGCCACACGCATGCCTGTGCATGGCCACGCCAGGTCGGAAACCCAGCCCCTCTGCTTGGGAGGCACGCAGGGCTCTGGCTGAAGCCTGAAGCTTGGGATCCTCAGAGCACAGATGTCCCAGGGTTGTGTGAAGTTAGTGAGACGAGGTGTACGGAGAGAGCTCGGAGAGGAGAGGGAGTCTCGATTGCTGTGGCTGTGGCTCGGAGATGCCTGCAAGAGGTGACGGTGATGTGCGTGGTGGTGTGTTCACTGATGATGCTACACTGCAGGCCCAGGGCTGTGTTGTGGTGGCAGGTGTCCTCCTGGACCGTTTGACCTTTAGGGAGGCCTCCTCCACTCAAGCACTGGTCCTGGAGGGGCTCTGGGCACACAAGGAGTCAGGCATGTTTTTGCCCCCGAGGAGCCCCCCAGCCTGGCAGGGCCCAGGGGCAGGGCAGGGTGGGGCGGGGTGGGATGGGGGTGCTCCAGGAGGTAGCAGGGACACTTGGTAAGTGGGGCCAAATTGGGAGGCATCTCAGGAGGTGGCCATGGGCAGAGGGACAGCCTTCCAGTTCTTTGCCTCTCCCCTTTGAAGCCCGGCGCCTGGCAACAGACCTGTGGTAGCTGCACCTGCCTGGGTAGGAGACTGTGGGCTCTTTTCTCGTAGGGGGACAAGAGTCATTTGCAGGACCTTCTGAGCAGGTGGGGGACAGCGAAGGACAAGAGGAGGCTGTGCCTGTGCGGCTGTGTCCCGGCACAGGTGCCGCTTCATGCCCACGCCACCACATCCTCACTGGGTGGCTTCGGGCAAGTTACCTGAACTCTGCGCCTCCATCTCGTCTTCTGTAAAATGGGTGGCACATCCGAGCCCACCTCCTAGGGAGGGTGTTGTGAGGACAGGTGGGGGCACGTCCGCACAGCCCCTAGAATAGTGCCCAGGGCACCAGAGTGCTCTGTAGGGAAATAGTAGTGGCTGGCACTGAGCCCCCAACATAAGCCAGCACCGTGGGCCAGAGCCACGACTCCTGCTTCTGGGCACTTCACAAGCTATGGCCAGGGGCCACCACCTTTGGCCACTCCTTTCCCACAGAATTCCTACCAGGGCCCTGCCAGGTCAACAAGTCACAGGGCTTCCCCATCACAGAAGCCCAGCTCTGTGACACCTCTTGGTCCCAAGCACGTTCCTATCCCCCATTCATGTGTTTTGTTAGGACCCTTTAGGGTTGCACATGGCAGAAACTTAATCCACACCAACTTCAGCGAAGAAGGAATGTACTGGCTCCAGTCAGTGAAAAGTCCAGGAGGAGCCATGCCTTCAGGCACGGCTGCATCCAGGCACTCAACAGGTGTATCCAGGCACTCAACAGGTGCAGCCAACACCTTGCCCCGCAGGCTGTCAGCTCCTGCCTTCTGCTGCGTCCACGTCACTGTAGGTGGATTTCTCTGCACATGGGTGAGATGGCCCCAGAAGCTCTGGCTCATGGAGTCCTTGTAGCACCTGTCCTGGCCATCCCCAGATGAGAAAGCATCATTTCCCATGGCAGAAATCTGGTGGAAACAGGAGGGAGGGGGTCGGCTCTCTTTGGTCGGATCTGGGTCTCGTGCCCATCTCTGGGAGAGAAAAGTCCCCATTCACAGAAACTGAGCAGATCCTGGATTGGATGGGGAAGGAGAGATGCTGGCAGATACACACACATTCACTGCACTCCCTGCAAGGATAGTGACAATAGTAGAAGCTGCTCTTTCTTGAGAAGTTATGACGTGTGCCAGAAAATTCTACCTTTTAAGGATATAGAGAGGCTTTTATCATCCCCATTTTACAGATGACAAAACTGAGGCTCAGAGAGGTGAAGTGATTTGCCTATGGATGCATAGCTAAGGTCTGACCCCACAACTCACACTCACAAGTTAGCAAGAGGTGTTTCACCAGGGTAGTCTGGGGGCAGAAGGAGCCTGCCTGGTGAGGGAAGGGAGGTGGAGAGGTGGGGAGAGGGGAGGCCAAGAGGAGGGGACACCAGGACCTGGCCTCTGGCTTGCACAATGGGGAGGGAACCCTGGAGAAGAGGAGGTTTATGGGGGAGGCTGCTGAGTTTTTTGGGCACATATGCTAAGTTGGAGGTGCCCATGGTCCTCAGGAGATGTTCGGGATTTGGGGGTCAGAGGCATGAGCCTCGGCATCCTGGGAATCATGGCTGTTTGCTTTGCTTTGGGAATTTCACAGTGGTGGGGGGAAGGCGGCTGCTGACAGGAAGCACTTCCCATTTGGTGGAGAAGGCAGCCAGAACCTGCATGGCAGAAGCTGCGGCCTGGGCCTGTGACAGTGTCTACAACTGTGGCCACAGGTTCAAGGCCCTTGTAGTTGGCAGACACAGGGCCTGAAGTTCTCTGGCCGTTGTTTTAGGGCAAACACCTTGAGAATGGCATTTCCAAGGCCCTTGGGCTGTGCCAGGGCACCAACGCCAGTCGAGGACCACGCCTTTCTGAGGGATGCTTGGAATCTCACAGACAGGGGTTCACATTCCGACTCTGCTTCCTAGAGGCTATGTGGCCTTGGGCGTGTCTGGAACCTCTCTGGGCCACAGTTTGCTTATCTGTGAAAGAGTCATGGTAGCCTCTCCTTATGGGTTTATGGGGAGCTTCGGATATGGAGGGTGGGGCAGCACACAGGGCTCAAGCTGCAGCTCTTAGTTACCTGCTTTTCCTTTGCAGGTGTCCTACGGGCCTGATGGAAAGGTGGCAGGCGAAATGCACCTGGCCTCAGCGTCAGGGGCTGTGAATGCAGTAGCACTTGTAGCTATGAGGTCAGAGTGGACTAGGATGGTCCTTCCACCAAGAAGCCTGGGCCTTCCTGCTCTTCGTTGTCCAGCTGCAGAAACTGATCTCATCCCTCAGGGCTTGGCACAACTGCCCCTTCCTCCTCACATTCCCAAATATACCTACCCCTCCCCAACTCAATCCTCCTCACCAGACTGTGGCTCCAGGATGACAGGGCAGAGTTGGACTCACCTGTGTCTGCCAGGCCAGCTCACCGTGGGGGCAGGTCCCAAGAGGCCTCTGGATTTGGACCAACTTCCAGTGTGACCTTGAACAAGGCCTTCGCCTCCCTCAGCCTCCGTTGCATGAGGGGTTGTGCTGGCACATCTCAGAGCAGGTAGCGCTACCTGGTGAGTTGAGGGCTCTGTCCATCATCTTTGTCACTGTAATGCTGGGTAACAAATAAACACGAAACAATGAGTGCTCGTT

**Table S3. Baseline clinical characteristics of patients with osteosarcoma in validation cohort.**

| **Characteristic** | **Low-DIO3OS** | **High-DIO3OS** | **P-value** |
| --- | --- | --- | --- |
| **n** | 16 | 16 |  |
| **Gender, n (%)** |  |  | > 0.05 |
| Female | 7 (21.9%) | 2 (6.2%) |  |
| Male | 9 (28.1%) | 14 (43.8%) |  |
| **M/NM, n (%)** |  |  | < 0.05 |
| M | 2 (6.2%) | 9 (28.1%) |  |
| NM | 14 (43.8%) | 7 (21.9%) |  |
| **Survival status, n (%)** |  |  | < 0.05 |
| Alive | 15 (46.9%) | 6 (18.8%) |  |
| Dead | 1 (3.1%) | 10 (31.2%) |  |
| **Age, mean ± SD** | 29.38 ± 16.47 | 30.94 ± 15.94 | 0.787 |
| **Survival time, mean ± SD** | 2505.38 ± 783.16 | 1126.12 ± 866.97 | < 0.001 |
| **DIO3OS, median (IQR)** | 1.29 (1.01, 1.96) | 4.49 (3.42, 6.76) | < 0.001 |
